# Abstracts of the 10th International Conference on Cachexia, Sarcopenia and Muscle Wasting, Rome, Italy, 8–10 December 2017 (Part 1)

**DOI:** 10.1002/jcsm.12255

**Published:** 2017-11-23

**1-01**

**Body composition changes over three years in older adults: a descriptive longitudinal analysis**

Maria Teresa Tomás^1,3^, Alejandro Galán‐Mercant^2,3^ and Beatriz Fernandes^1,3^

^1^ *Escola Superior de Tecnologia da Saúde de Lisboa, Portugal;* ^2^ *Universidade de Jaén, Spain;* ^3^ *2GHRG—Gerontology and Geriatric Health Research Group*

**Introduction:** Many studies analyse body composition changes in older adults. However, few studies analyse body composition in elderly people with functional measures. Studies using Double X‐Ray analysis (DXA) or Bioimpedance analysis proved to be reliable but expensive or only possible in a laboratory environment.

The purpose of our study was to analyse changes in body composition over three years using anthropometric measures in a sample of elderly people in order to perceive functional changes.

**Methods:** Forty‐three participants (12 men; 31 women) aged 60 years and over and independent in activities of daily life were assessed using anthropometric measures in a first moment and past three years. Weight, height, waist and hip circumference were measured, and body mass index (BMI) and waist‐to‐hip ratio (WHR) were also calculated. Skeletal muscle mass (SMM) was also calculated using Al‐Gindan et al. (2014) equations and normalized for height to found skeletal muscle index (SMI) in order to analyze cut‐off points associated with physical disability according to Janssen et al (2004).

**Results:** A significant difference was found over three years in SMM (*p* = 0.007), SMI (*p* = 0.027), BMI (p = 0.041) and WHR (p = 0.003). The majority of the participants has decreased SMM, SMI and BMI and increased WHR, which favors a worst prognostic for comorbidities associated with these variables, and a tendency for sarcopenic obesity seems to be present although more studies are needed. Also, we found that using cut‐off points for disability risk 83.3% of the men and 38.7% of the women of our sample were at moderate or high risk of disability. Three years later this percentage has increased but only for women to 54.8%.

**Conclusions:** Although men are at risk of disability, women quickly lose their functional capacity, making necessary a rapid intervention to reduce the risk of disability in this population.

IPL/2016/SFQ2017_ESTeSL

**1-02**

**Prevalence of cachexia in dogs with congestive heart failure**

Pamela L. Bay, Lisa M. Freeman and John E. Rush

*Cummings School of Veterinary Medicine, Tufts University, North Grafton, MA, USA*

**Background and Aims:** Congestive heart failure (CHF) is a common, naturally occurring disease in pet dogs that is often associated with cardiac cachexia, as defined by a loss of muscle. One study of dogs with dilated cardiomyopathy (DCM) and CHF showed that 54% of dogs were affected by cachexia. No studies have been conducted to confirm these findings in dogs with DCM or to assess prevalence in CHF from other forms of heart disease causing CHF. Therefore, the aim of this study was to determine prevalence of cardiac cachexia in dogs with CHF due to acquired heart disease.

**Methods:** Dogs with CHF evaluated by the Cardiology Service at the Cummings School of Veterinary Medicine between June 2015 and June 2017 were eligible. Dogs with DCM and myxomatous mitral valve disease (MMVD) were enrolled. Data from the medical records were retrospectively reviewed, including body weight, body condition score (BCS), and muscle condition score (MCS). Body condition score, which assesses fat stores, was measured on a 1–9 scale, with 1 = emaciated, 9 = obese, and 4–5 considered ideal. Muscle condition was categorized using the World Small Animal Veterinary Association scoring system as normal muscle, mild muscle loss, moderate muscle loss, or severe muscle loss.

**Results:** Median age of the dogs (*n* = 196) was 10.7 years (range, 1.7–18.0 years). Underlying diseases included MMVD (*n* = 168) and DCM (*n* = 28). Mean body weight was 7.6 kg (range, 2.4–75.8 kg). Only 6.1% of dogs were underweight (BCS < 4/9), and 41.8% of dogs were overweight or obese (BCS > 5/9). However, muscle loss was identified in 48.0% of dogs: Mild muscle loss: 73/196 (37.3%), moderate muscle loss: 14/196 (7.1%), and severe muscle loss: 7/196 (3.6%). 52.0% of dogs were assessed to have normal muscle. Muscle condition score and BCS were not significantly different between dogs with MMVD or DCM.

**Conclusions:** Although many dogs were overweight or obese, cachexia was present in 48% of dogs with CHF.

**1-03**

**Comorbidities and mortality after cachexia hospitalization in Slovenia between 2004 and 2015**

Daniel Omersa^1^, Jerneja Farkas^2^ and Mitja Lainscak^2,3^

^1^ *National Institute of Public Health, Ljubljana, Slovenia;* ^2^ *General Hospital Murska Sobota, Murska Sobota, Slovenia;* ^3^ *Faculty of Medicine, University of Ljubljana, Ljubljana, Slovenia*

**Introduction:** Cachexia is common in several chronic diseases and significantly increases morbidity and mortality. There is a lack of data regarding cachexia hospitalization burden and mortality after cachexia hospitalization. Thus, we aimed to identify all patients that were hospitalized due to cachexia and determine their mortality and prognostic implications of different comorbidities.

**Methods:** The Slovenian National Hospitalization Database has been searched for all individuals with main Cachexia hospitalization (ICD‐10 codes: C80, R64 and B22.2) between 2004 and 2015, and sex, age, length of stay and comorbidities were recorded. For all patients with cachexia hospitalization, date of death was recorded from Slovenian Death Registry. Prevalence of comorbidities during cachexia hospitalization were calculated and hazard ratios (HR) for mortality for sex, age and patients' comorbidities were calculated using multiple Cox proportional hazards model.

**Results:** Overall, we identified 1774 main cachexia hospitalizations in 1406 patients. Main cachexia hospitalizations contributed to 17.7% of all the hospitalizations of an individual during the study period. Cancer, cardiovascular and pulmonary diseases were the most prevalent in cachexia patients (62%, 27% and 10%, respectively). In‐hospital mortality was 29%. Median survival for discharged patients were 103 days (95% confidence intervals, 90–123 days). Older patients, those with cancer and pulmonary disease, had significantly higher HR for mortality (1.16 for 10 year increase, 1.79 and 1.34, respectively).

**Conclusions:** Patients hospitalized due to cachexia have extremely poor prognosis. Cancer, which is the most prevalent comorbidity in patients hospitalized due to cachexia, is associated with the worst prognosis.

**1-04**

**Prevalence of cachexia among COPD cases in the ECLIPSE study**

Merry‐Lynn N. McDonald^1,2^, Erica Rutten^3^, Richard Casaburi^4^, Emiel F.M. Wouters^3^, Stephen I. Rennard^5^, David A. Lomas^6^, Bartolome Celli^7^, Alvar Agusti^8^, Ruth Tal‐Singer^9^, Craig P. Hersh^7,10^ and Edwin K. Silverman^7,10^

^1^ *Division of Pulmonary, Allergy and Critical Care Medicine, University of Alabama at Birmingham, Birmingham, AL, USA;* ^2^ *Department of Genetics, University of Alabama at Birmingham, Birmingham, AL, USA;* ^3^ *Centre of expertise for chronic organ failure, Horn, the Netherlands;* ^4^ *Rehabilitation Clinical Trials Center, Los Angeles Biomedical Research Institute at Harbor Harbor‐UCLA Medical Center, Torrance, CA, USA;* ^5^ *Department of Medicine, Nebraska Medical Center, Omaha, NE, USA;* ^6^ *Wolfson Institute for Biomedical Research, University College London, UK;* ^7^ *Division of Pulmonary and Critical Care, Brigham and Women's Hospital, Boston, MA, USA;* ^8^ *Fundació Investigació Sanitària Illes Balears (FISIB), Ciber Enfermedades Respiratorias (CIBERES), Barcelona, Catalunya, Spain Thorax Institute, Hospital Clinic, IDIBAPS, Univ. Barcelona, Barcelona, Spain;* ^9^ *Respiratory R&D, GSK, Philadelphia, PA, USA;* ^10^ *Channing Division of Network Medicine, Harvard Medical School, Boston, MA, USA*

**Background:** By population prevalence, there are more chronic obstructive pulmonary disease (COPD) cases than cancer cases with cachexia. The consensus definition of cachexia incorporates weight loss (WL) >5% in 12 months in addition to 3 out of 5 of decreased muscle strength, fatigue, anorexia, low fat‐free mass index (FFMI) and abnormal biochemistry (anemia, CRP, IL6, albumin). More recently, cancer cachexia has been classified using WL >5% or, in the presence of low BMI or FFMI, WL >2%. Further, the importance of pre‐cachexia (WL ≤5%, anorexia and inflammation) has been highlighted as more advanced cachexia may indicate a refractory state. Thus, we aimed to examine the prevalence of cachexia using these definitions in a cohort of COPD cases from the ECLIPSE Study.

**Methods:** A total of 1901 COPD cases were assessed for cachexia. Annual weight, muscle strength, FFMI and anemia data were analyzed. Fatigue and anorexia data were available at baseline and end of study. CRP levels were measured at baseline and over the first year. The consensus definition was coded at each annual visit an individual participated in the study. Where data were not available for the specific visit, an aggregate was created (e.g., ever had fatigue). Participants who exhibited WL at an early visit with evidence it was regained were coded as non‐cachectic.

**Results:** The prevalence of cachexia based on the consensus definition ranged from 4.0% (Year 1) to 6.6% (Year 3). Over 3 years of the study, 11% of COPD cases were classified as cachectic at some time point. The prevalence of cachexia using the cancer cachexia definition ranged from 9.7% (Year 1) to 17.7% (Year 3). The prevalence of pre‐cachexia ranged from 2.0% (Year 2) to 3.1% (Year 1).

**Summary:** Based on definition of cachexia and visit used, the prevalence in a large cohort of COPD cases ranged from 4.0% to 17.7%.

**1-05**

**SARA‐data: Integrated, real‐time ICT Platform for the SARA interventional Clinical Trial in Age‐related SARcopenia**

Susanna Del Signore^1,2^, Waly Dioh^2^, Stefania Del Signore^1^ and Gianluca Zia^1^

^1^ *Bluecompanion ltd, London, UK;* ^2^ *Biophytis, Paris, France*

**Introduction:** SARA‐Int(erventional), a randomized, double‐blind clinical trial, will evaluate the safety and efficacy of two oral doses of SARconeos (BIO101) versus placebo over 6 months in 333 sarcopenic or obese sarcopenic patients Aged ≥65 years complaining of loss of strength and muscular function.

**Methods:** We deployed an integrated Information&Communication Technology (ICT) platform, SARA‐data, to monitor on *quas*i real‐time different source data (clinical, imaging (DEXA), laboratory and physical activity). Data can be generated at investigation sites, by the centralised lab and by the patients themselves via wearable devices and auto‐evaluation questionnaires.

Bluecompanion implemented for Biophytis SARA Data, which allows to collect and integrate on one single web‐based portal: an electronic Case Report Form (Clean WEB by Telemedicine), participants row data from DEXA scans, and biochemistry‐haematology results from a centralised laboratory, including sarcopenia‐related biomarkers. Of note, continuous physical activity recording is enabled during the whole clinical trial duration by providing each older participant with a wrist‐worn accelerometer. The device transmits anonymised activity data to SARA platform via a non‐intrusive, unattended, home‐centred machine‐to‐machine technology.

This kind of high volume data, directly generated by the patient during several months, fulfils the definition “Big data in health”, encompassing “high volume, high diversity biological, clinical, environmental, and lifestyle information collected from single individuals to large cohorts, in relation to their health and wellness status, at one or several time points” (Auffray C. *et al*., 2016), and will constitute an important resource for additional, supportive analyses complementing standardised muscular function assessments. These data are generated in a real‐life context and could provide answers to specific questions by regulators and payers.

**Conclusions:** SARA‐data are a single real‐time ICT platform enabling data capture, storage, analysis and retrieval of long‐term clinical data generated during SARA‐Int, a randomized CT evaluating Sarconeos (Bio101) in Age‐related Sarcopenia, including Sarcopenic Obesity.

**1-06**

**Changes of body weight and body composition and cachexia after stroke**

Nadja Scherbakov^1^, Charlotte Pietrock^1^, Nicole Ebner^2,3^, Anja Sandek^2,3^, Miroslava Valentova^2,3^, Jochen B. Fiebach^1^, Joerg C. Schefold^4^, Stephan von Haehling^2,3^, Stefan D. Anker^2,5^, Kristina Norman^6^, Karl Georg Haeusler^1,7^ and Wolfram Doehner^1,5^

^1^ *Center for Stroke Research Berlin CSB, Charité ‐ Universitätsmedizin Berlin, Berlin, Germany;* ^2^ *Innovative Clinical Trials, Department of Cardiology and Pneumology, University Medicine Goettingen (UMG), Goettingen, Germany;* ^3^ *German Centre for Cardiovascular Research (DZHK), partner site Goettingen, Goettingen, Germany;* ^4^ *Department of Intensive Care Medicine, Inselspital, Bern University Hospital, Switzerland;* ^5^ *Department of Cardiology, Charité ‐ Universitätsmedizin Berlin, Berlin, Germany;* ^6^ *Research Group on Geriatrics, Charité ‐ Universitätsmedizin Berlin, Berlin, Germany;* ^7^ *Department of Neurology, Charité ‐ Universitätsmedizin Berlin, Berlin, Germany*

**Background and Purpose:** Body weight loss after stroke has been shown in several clinical trials. Cachexia after stroke has not been studied systematically yet. The purpose of this prospective study was to investigate dynamical changes of body composition and body weight one year after ischemic stroke, and its association with functional outcome.

**Methods:** 67 consecutive patients with acute ischemic stroke (age 69 ± 11 years, BMI 27 ± 4 kg/m^2^) with mild to moderate neurological deficit (mean NIHSS 4.3, range 0–12) were analyzed. Body composition was examined by dual energy X‐ray absorptiometry (DEXA) in acute phase (4 ± 2 days) and at 1‐year follow‐up (389 ± 26 days). Cachexia was defined according to consensus definition by body weight loss ≥5% within one year and clinical symptoms. Functional assessments included Barthel Index (BI), modified Rankin scale (mRS), and muscle strength tests.

**Results:** Cachexia was diagnosed in 21% of the patients at 1‐year follow‐up. Most changes of body composition concerned the fat tissue with the highest fat mass decline of 12.5% in cachectic patients, followed by 5% loss in non‐cachectic patients with weight loss, and fat mass increase by 8% in patients with weight gain. In addition, cachectic patients lost 3% of the lean mass (*P* < 0.05).

At baseline patients who developed cachexia during follow up were older (75 ± 9 years), had moderate neurologic deficit (mean NIHSS 5.8), and the lowest physical and functional capacity. They remained with the worse functional impairment (mRS 2.1 ± 1.6, *P < 0.05*, Barthel Index 74 ± 36, *P = 0.002*) and handgrip strength (22.4 ± 14.9 kg, *P < 0.05*) compared to other patients at 1‐year FU. After adjustment for multiple confounders, patients with higher functional impairment (OR 1.87, 95% CI 1.09–3.20) and neurologic deficit (OR 3.63, 95% CI 0.97–13.6) were at risk for cachexia.

**Conclusions:** The most changes of body composition after stroke with mild‐to‐moderate neurologic deficit concerned the fat tissue. Attention should be focused on identification and targeting of cachexia in the early phase following the stroke.

**2-01**

**Ultrasound: a new strategy to evaluate body composition in crohn's patients undergoing hematopoietic stem cell transplantation (HSCT)**

Andrea Z. Pereira^1^, Sandra E.A. Gonçalves^1^, Bianca L. de Sá^2^, Marister Cocco^3^, Andreza A.F. Ribeiro^1^ and Nelson Hamerschlak^1^

^1^ *Oncology and Hematology Department, Hospital Israelita Albert Einstein, S. Paolo, Brazil;* ^2^ *Nutrition Department, Hospital Israelita Albert Einstein, S.Paulo, Brazil;* ^3^ *Physiotherapy Department, Hospital Israelita Albert Einstein, S.Paulo, Brazil*

**Introduction:** Crohn disease is a chronic inflammatory disorder of the gastrointestinal tract with a strong polygenic immune component. In refractory cases, autologous HSCT can decrease disease activity and mucosal healing and improve quality of life. Reduced muscular mass and excess visceral fat in patients undergoing HSCT are associated with higher mortality, longer hospitalization, longer use of immunosuppressive drugs, graft‐versus‐host disease, shorter disease‐free interval after the HSCT and comorbidities leading to shorter survival time.

**Objectives:** To evaluate muscle thickness and visceral fat by US.

**Methods:** We evaluated 5 HSCT patients (≥18 years) at Hospital Israelita Albert Einstein, São Paulo, Brazil, on their first day of hospitalization, before HSCT and after the engraftment. The thickness of the right femoral quadriceps muscle (RFQ), measured at 6 cm from the top edge of the patella was measured using US in B‐mode. The VF was measured in the abdominal region, by the thickness of the fat layer between the linea alba and the anterior wall of the aorta.

**Results:** Most patients were men (75%) with a mean age of 35 years (±14 years). Most patients were undernutrition, with body mass index (BMI) of 21 kg/m^2^ (±2.5 kg/m^2^). The average time EN was 11 days (±1 day). In the baseline, RFQ was 1.5 cm (±0.2 cm), and the VF was 4.2 cm (±1.3 cm). After engrafment, RFQ was 1.3 cm (±0.2 cm), and the VF was 4.2 cm (±1.2 cm). There wasn't significant difference between baseline and after engraftment, although RFQ had reduced in all patients.

**Conclusions:** In this cohort of patients, we found reduced muscle thickness after engraftment, and VF didn't have any alterations. The US was a practical, economical and effective method to evaluate these patients.

**2–02**

**Elderly patients undergone hematopoietic stem cell transplantation: body composition and engraftment**

Andrea Z. Pereira^1^, Ludmila M. Koch^1^, Polianna M.R. Souza^1^, Bianca L. de Sá^2^, Andreza A.F. Ribeiro^1^ and Nelson Hamerschlak^1^

^1^ *Oncology and Hematology Department, Hospital Israelita Albert Einstein, S.Paulo, Brazil;* ^2^ *Nutrition Department, Hospital Israelita Albert Einstein, S.Paulo, Brazil*

**Introduction:** Hematopoietic Stem Cell Transplantation (HSCT) in elderly is a brand‐new issue. Changes in body composition after HSCT have been the subject of previous studies; however, there aren't many studies in elderly people.

**Objectives:** To evaluate muscle thickness and visceral fat by US; % muscle mass, % fat mass and phase angle by BIA. To correlate body composition with engraftment (EN).

**Methods:** In this prospective study, we evaluated 16 HSCT patients (≥60 years) at Hospital Israelita Albert Einstein, São Paulo, Brazil, on their first day of hospitalization, before HSCT and after the EN. The thickness of the right femoral quadriceps muscle (RFQ), measured at 6 cm from the top edge of the patella was measured using ultrasound (US) in B‐mode, transversal plane. The visceral fat (VF) was measured in the abdominal region, by the thickness of the fat layer between the linea alba and the anterior wall of the aorta. The % muscle mass (MM), % fat mass (FM) and phase angle (PA) were evaluated by Bioimpedanciometry(BIA).

**Results:** Most patients were men (75%) with a mean age of 64(±5.0 years). We had 50% of autologous HSCT and 50% allogenic HSCT. The mean time EN was 13(±4 days). In the baseline, weight was 80(±17 kg), RFQ was 1.8(±0.3 cm) and the VF was 5.5(± 2.0 cm); %MM was 68.5(±11); %FM was 27.5(±7.5); PA was 5.3((±0.7). After EN, weight was 73(±13 kg). RFQ was 1.5(±0.3 cm) and the VF was 5,0(±2.2 cm); %MM was 55.5(±20.5); %FM was 25(±7.0); PA was 7.4(±0.8). There wasn't significant difference between baseline and after engraftment, although all measurements had reduced in all patients, exception for PA and VF had increased. We found the negative correlation between engraftment and RFQ(rp: −0,6), independently of HSCT type by regression. (r_p_: −0,6).

**Conclusions:** In this cohort of patients, muscle thickness and mass was reduced, and visceral fat and phase angle was increased after engraftment. The higher muscle thickness correlated faster engraftment.

**Elderly quilombolas:** Prevalence of sarcopenia using algorithm proposed by the European working group on sarcopenia in older people.

**2-03**

**Prognostic value of psoas muscle area and density in patients undergoing cardiovascular surgery**

Masashi Yamashita^1^, Kentaro Kamiya^1,2^, Atsuhiko Matsunaga^1,2^, Tadashi Kitamura^3^, Nobuaki Hamazaki^4,5^, Ryota Matsuzawa^4^, Kohei Nozaki^4^, Shinya Tanaka^5^, Junya Ako^6^ and Kagami Miyaji^3^

^1^ *Department of Rehabilitation Sciences, Graduate School of Medical Sciences, Kitasato University, Sagamihara, Japan;* ^2^ *Department of Rehabilitation, School of Allied Health Sciences, Kitasato University, Sagamihara, Japan;* ^3^ *Department of Cardiovascular Surgery, Kitasato University School of Medicine, Sagamihara, Japan;* ^4^ *Department of Rehabilitation, Kitasato University Hospital, Sagamihara, Japan;* ^5^ *Department of Cardiovascular Medicine, Kitasato University Graduate School of Medical Sciences, Sagamihara, Japan;* ^6^ *Department of Cardiovascular Medicine, Kitasato University School of Medicine, Sagamihara, Japan*

**Introduction:** Low skeletal muscle area and density, as determined by computed tomography (CT), have yet to be examined and compared in terms of prognostic capability in patients requiring open cardiovascular surgery. This study was performed to examine whether psoas muscle area and density are associated with postoperative mortality and physical performance in patients undergoing cardiovascular surgery.

**Methods:** We reviewed the findings in 773 consecutive patients undergoing preoperative CT imaging including the level of the third lumbar vertebra for clinical purposes. Skeletal muscle area was calculated from psoas muscle cross‐sectional area (CSA) on preoperative CT images at the level of the third lumbar vertebra divided by the square of the patient's height to give the skeletal muscle index (SMI: cm^2^/m^2^). Skeletal muscle density determined by muscle attenuation (MA) was calculated by measuring the average Hounsfield units of the psoas muscle CSA. Quadriceps strength and 6‐minute walking distance were examined as indices of physical performance.

**Results:** The mean age of the study population was 68.6 ± 14.0 years, and 64.7% of the patients were male. Multivariate Cox regression analysis showed that low MA (hazard ratio [HR], 2.18; 95% confidence interval [CI], 1.14–4.16, *P* = 0.018), but not low SMI (HR, 1.35; 95% CI, 0.71–2.56, *P* = 0.361), was significantly associated with all‐cause mortality. Kaplan–Meier analysis showed that low MA, but not low SMI, predicted poor prognosis (*P* = 0.014). Correlation analysis indicated that MA was more strongly associated with quadriceps strength and 6‐minute walking distance than SMI.

**Conclusions:** Low skeletal muscle density, but not skeletal muscle area, predicted survival in patients undergoing cardiac surgery.

**2-04**

**The usefulness of body weight for predicting skeletal muscle mass in congested state of heart failure outpatients**

Shunichi Doi^1^, Norio Suzuki^1^, Keisuke Kida^2^, Chikayuki Ito^3^, Kohei Ashikaga^3^, Kengo Suzuki^2^, Hisao Matsuda^1^, Tomoo Harada^2^ and Yoshihiro J. Akashi^2^

^1^ *Division of Cardiology, Department of Internal Medicine, St. Marianna University School of Medicine Yokohama City Seibu Hospital, Yokohama, Japan;* ^2^ *Division of Cardiology, Department of Internal Medicine, St. Marianna University School of Medicine, Kawasaki, Japan;* ^3^ *Division of Cardiology, Department of Internal Medicine, Kawasaki Municipal Tama Hospital, Kawasaki, Japan*

**Introduction:** Body mass index is cited as an index to recognize significant correlation with skeletal muscle mass. However, body weight changes due to edema are often observed in chronic heart failure (CHF). We investigated the usefulness of body weight for evaluating skeletal muscle mass wasting in congested CHF outpatients.

**Methods:** Totally 45 CHF outpatients with brain natriuretic peptide (BNP) ≥200 pg/ml were enrolled. Total skeletal muscle mass was measured at the level of the third lumbar vertebra using available preoperative computed tomography images (Cutoff value: male 36.2cm^2^/m^2^, female 29.6cm^2^/m^2^). It was investigated on the relationship between skeletal muscle mass and each nutritional indicator.

**Results:** The mean age was 75.6 ± 6.4 years old, body mass index (BMI) was 22.4 ± 2.9 kg/m^2^ and left ventricular ejection fraction was 44.3 ± 18.9%. Median BNP was 417.5 pg/ml (interquartile range, 271.1–590.8). Of the study patients, 53.3% patients were male, 26.7% patients had ischemic heart failure, 57.8% patients had New York Heart Association (NYHA) classification ≥2, and 68.9% patients had Mini Nutritional Assessment Short Form (MNA‐SF) score ≤ 11. Correlation between skeletal muscle mass and each index was BMI (*r* = 0.51, *p* < 0.01), Geriatric Nutritional Risk Index (GNRI; *r* = 0.42, *p* = 0.04), MNA‐SF(*r* = 0.28, *p* = 0.15) and serum albumin value (Alb; *r* = −0.06, *p* = 0.77). The logistic regression analysis indicated that the odds ratio, in BMI, was 0.66 (95% confidence interval; 0.48–0.85, p < 0.01) and area under the receiver operating characteristic curve (AUC) was 0.76, suggesting that BMI might be independent predictors for muscle mass wasting.

**Conclusions:** Though the congested state of CHF outpatients, it was suggested the usefulness of body weight for predicting skeletal muscle mass.

**2-08**

**Comparison of height‐, weight‐, body surface area‐, and body mass index‐adjusted muscle mass indices for prediction of physical performance in Korean hemodialysis patients**

Jun Chul Kim^1^, Jun Young Do^2^, Kyu Hyang Cho^2^ and Seok Hui Kang^2^

^1^ *Division of Nephrology, Department of Internal Medicine, CHA Gumi Medical Center, CHA University, Gumi, Gyeongsangbuk‐do, Republic of Korea;* ^2^ *Division of Nephrology, Department of Internal Medicine, Yeungnam University Hospital, Daegu, Republic of Korea*

**Introduction:** Our study aims to evaluate the association between height‐, weight‐, body surface area‐ (BSA), or body mass index‐ (BMI) adjusted muscle mass indices and physical performance in Korean hemodialysis patients.

**Methods:** Patients were included if they were on HD for ≥6 months (*n* = 84). Each patient's appendicular skeletal muscle mass (ASM, the sum of both upper extremities and lower extremities) was measured by dual X‐ray absorptiometry. ASM was adjusted to body weight (BW, kg), height^2^ (Ht^2^, m^2^), BSA (m^2^), or BMI (kg/m^2^). Low muscle mass was defined as muscle mass of 2SD below sex‐specific means of healthy young adults (20–29 years). Each participant performed a gait speed test (GS), a hand grip strength (HGS) test, a sit‐to‐stand test performed 5 times (5STS), a sit‐to‐stand for 30 second test (STS30), a 6‐minute walk test (6MWT), a timed up and go test (TUG), and an average steps count (AS).

**Results:** In men, Pearson's correlation coefficients for GS, 5STS, STS30, 6‐MWT, TUG, and AS were highest in ASM/Ht^2^. Results from partial correlation or linear regression analyses displayed similar trends to those derived from Pearson's correlation analyses. ASM/Ht^2^ had the greatest negative predictive value for low GS. Patients with low muscle mass had significantly lower physical performances (HGS, 5STS, 6MWT, TUG) than those with normal muscle mass by ASM/Ht^2^. In women, the association between muscle mass indices and physical performance was lack.

**Conclusions:** Height adjusted muscle mass may be the best for predicting physical performance in men on hemodialysis.

**2-12**

**Prevalence and progression of limb contractures amongst long‐term care residents: data from a 5‐year observational study**

Kuen Lam^1^, Joseph Kwan^2^, Chi Wai Kwan^3^ and Iris Chi^4^

^1^ *Cheshire Home, Shati, Hong Kong;* ^2^ *Department of Medicine, The University of Hong Kong, Hong Kong SAR;* ^3^ *Department of Statistics and Actuarial Science, The University of Hong Kong;* ^4^ *Suzanne Dwork‐Peck School of Social Work, University of Southern California, Los Angeles, CA, USA*

**Background:** Limb contractures are associated with poor outcomes and quality of life in long‐term care facility (LTCF) residents. We aimed to study the prevalence and progression of limb contractures over a 5‐year follow‐up period amongst LTCF residents in Hong Kong.

**Methods:** From the Hong Kong Longitudinal Study on LTCF Residents between 2005 and 2015, we analyzed the data for residents who had assessment from the 1st up to 5th year since admission. Trained nurses, social workers and therapists utilized the Minimum Data Set Resident Assessment Instrument (MDS‐RAI 2.0) in 10 residential LTCFs. Limb contractures were defined as functional limitation in the range of motion involving the upper or lower limbs. Primary outcomes were annual prevalence and time trend of limb contractures over 5 years.

**Results:** We analyzed the data for 1,736 older residents (611 men, mean age 83.2 years). During the first 5 years since admission, the annual prevalence of upper limb contractures increased from 30% to 36%, and lower limb contractures increased from 41% to 56%. Time trends were as follows: the proportion of residents who had no contractures on admission remained contracture‐free after 5 years was 59.7% for upper limbs and 39.8% for lower limbs, while the proportion of residents who had developed new contractures after 5 years was 15.1% for upper limbs and 26.5% for lower limbs. The proportion of residents who had unilateral contractures on admission which had improved after 5 years was 4.1% for both upper limbs and lower limbs, and the proportion of residents who had either unilateral or bilateral contractures on admission which did not change after 5 years was 21.2% in upper limbs and 29.6% in lower limbs.

**Conclusions:** Joint contractures are highly prevalent amongst residents admitted to the LTCF, and many residents develop new or worse contractures during the first 5 years of their admission. Further studies are needed to identify the potential strategies to prevent functional decline in this vulnerable group.

**2-13**

**Validating the Care Assessment Need (CAN) tool for frailty screening**

Shivani Priyadarshni^1^, Zubair Rahaman^1^, Kimberly Cabrera^1^, Stuti Dang^1,2^, Willy Valencia^1^, Ramankumar Anam^1^, Michael J. Mintzer^1^ and Jorge G. Ruiz^1,2^

^1^ *Miami VAHS Geriatric Research Education and Clinical Center;* ^2^ *Clinical Center and University of Miami Miller School of Medicine*

**Introduction:** Frailty is a state of vulnerability to stressors resulting in higher morbidity, mortality and healthcare utilization in older adults. Multiple instruments are used to measure frailty; most are time‐consuming. The Care Assessment Need (CAN) score is automatically generated from electronic health record data using a statistical model; it is expressed as a percentile, ranging from 0 (lowest risk) to 99 (highest risk). The CAN score is a known predictor of high risk for hospitalization and mortality at 90 days and one year. The purpose of the study was to validate the CAN score as a screening tool for frailty among older adults in clinical practice.

**Methods:** This cross‐sectional study compared the CAN score with a reference standard. The reference standard, a 40‐item Frailty Index, was generated using retrospective data collected during a Comprehensive Geriatric Assessment (CGA) performed by geriatric medicine physicians. To assess the ability of the CAN score to correctly identify frailty, we calculated the sensitivity, specificity, positive predictive value (PPV), negative predictive value (NPV) and diagnostic accuracy (assessed by the area under the receiver operating characteristic (ROC) curve).

**Results:** 184 patients over age 65 were included in the study: 98% male, 61% White, 80% non‐Hispanic. Our CGA‐based Frailty Index defined 13% as robust, 55% as prefrail and 32% as frail. For the frail, statistical analysis demonstrated that a threshold CAN score of 52.5 provides sensitivity, specificity, PPV and NPV of 91%, 40%, 27%, and 95%, respectively. Area under the ROC curve was 0.749 (SD = 0.038, *p* = 0.0005, 95% CI = 0.674–0.823).

**Conclusions:** CAN score is a potential screening tool for frailty among older adults as it is generated automatically and provides acceptable diagnostic accuracy. Hence, CAN score may offer useful information to Primary Care Providers for early clinical interventions.

**2-14**

**Age‐related variations of skeletal muscle mass and strength among Italian and Taiwanese community‐dwellers: results from the Milan‐EXPO survey and the I‐Lan Longitudinal Aging Study**

Francesco Landi^1^, Matteo Tosato^1^, An‐Chun Hwang^2,3^, Liang‐Kung Chen^2,3^, Li‐Ning Peng^2,3^, Riccardo Calvani^1^, Anna Picca^1^, Roberto Bernabei^1^ and Emanuele Marzetti^1^

^1^ *Department of Geriatrics, Neurosciences and Orthopedics, Catholic University of the Sacred Heart, Rome, Italy;* ^2^ *Aging and Health Research Center, National Yang Ming University, Taipei, Taiwan;* ^3^ *Center for Geriatrics and Gerontology, Taipei Veterans General Hospital, Taipei, Taiwan*

**Background:** Age‐ and gender‐specific curves of muscle mass and strength, using data from large samples of community‐dweller people, need to be better established and so are possible differences among ethnic groups. The aims of the present study were to analyze age‐ and gender‐specific changes in measures of muscle and strength among community‐living persons and to identify differences between Caucasian and Asiatic individuals.

**Methods:** The Italian survey (“Longevity Check‐up”), conducted during EXPO 2015 in Milan, consisted of a population assessment aimed at evaluating the prevalence of specific health metrics in persons outside of a conventional research setting (*n* = 1924), with a special focus on muscle mass and strength. The Taiwanese survey used the first‐wave sampling from the I‐Lan Longitudinal Aging Study (ILAS) collected from August 2011 to August 2013 (*n* = 1839). Muscle mass was estimated by using calf circumference of the dominant side. Muscle strength was determined through handgrip strength testing.

**Results:** The mean age of the 1924 Italian participants was 62.5±8.3 years, of whom 1031 (53.6%) were women. Similarly, the mean age of the Taiwanese sample was 63.9±9.3 years with 966 (52.5%) women. Overall, cross‐sectional observations suggest that calf circumference decline with age in both genders. The calf circumference was significantly greater among Italian participants compared with Taiwanese people in all age groups. A similar effect of age was observed for muscle strength. As for calf circumference, muscle strength was significantly greater among Italian persons relative to Taiwanese participants.

**Conclusions:** Muscle mass and strength curves for Caucasian and Asiatic people may be used to derive reference values for subsequent use in research and clinical settings. In particular, the analyses of trajectories of muscle parameters may help identify cutoffs for estimating risk of adverse events as well as the optimal timing for intervening.

**2-15**

**Associations between lean mass, strength and mortality in the elderly: the EXERNET study**

Lucía Sagarra‐Romero^1,2^, Alejandro González‐Agüero^3,4,5,6^, David Navarrete‐Villanueva^3,5^, Alejandro Gómez‐Bruton^3,4,7^, Angel Matute‐Llorente^3,4^, José A. Casajús^3,4,5,6^, Ignacio Ara^2,8^, Germán Vicente‐Rodriguez^3,4,5,6^ and Alba Gomez‐Cabello^3,4,5,8,9^

^1^ *VALORA Reseach Group, Universidad San Jorge, Spain;* ^2^ *GENUD Toledo Research Group, Universidad de Castilla‐La Mancha, Toledo, Spain;* ^3^ *GENUD (Growth, Exercise, NUtrition and Development) Research Group, Universidad de Zaragoza, Spain;* ^4^ *Centro de Investigación Biomédica en Red de Fisiopatología de la Obesidad y Nutrición (CIBERObn), Spain;* ^5^ *Instituto Agroalimentario de Aragón (IA2);* ^6^ *Faculty of Health and Sport Sciences (FCSD), Department of Physiatry and Nursing, University of Zaragoza, Zaragoza, Spain;* ^7^ *Universidad Isabel I, Burgos, Spain;* ^8^ *Centro de Investigación Biomédica en Red de Fragilidad y Envejecimiento Saludable (CIBERFES), Spain;* ^9^ *Centro Universitario de la Defensa, Zaragoza, Spain*

**Introduction:** Negative changes in lean mass (LM) and muscle strength (MS) have been shown to occur across the aging process 1. These changes are linked with morbidity and have a negative effect on physical ability and independence in older adults and also increase risk of falls. However, the link between LM and mortality in the elderly is not clear.

**Purpose:** The aim of this study was to investigate the relationship between LM, MS and mortality in the elderly.

**Methods:** In this prospective longitudinal study a total of 223 seniors (64 men and 159 women) (age 73 ± 5.8 years) were evaluated in Zaragoza‐Aragon (Spain) during 2008, as part of the *elderly EXERNET* multi‐centre study 2. Whole body muscle mass was measured with dual energy X‐ray absorptiometry (kg). MS of upper and lower extremities was assessed using two tests from the “Senior Fitness Test”: “Chair Stand Test” and “Arm Curl Test”. Access to mortality data was obtained from the Spanish Statistical Office (INE) register in 2017. The Mann–Whitney U test was used to compare differences between groups (deceased vs alive).

**Results:** There were 19 deaths among the original participants**.** The mean total LM was 55.5 kg in men and 39.9 kg in women. No association was found between LM and mortality. However, MS in lower and upper body were statistically lower (*p* < 0.05) in the deceased group (12.7 ± 4.4 and 13.3 ± 4.9, respectively) compared with those still alive (14.3 ± 3.1 and 16.2 ± 3.8, respectively). These differences were maintained after adjustment for daily sitting time.

**Conclusions:** Lean mass is not associated with mortality in elderly people. However, there is an inverse association between muscular strength and mortality suggesting that functionally rather that the size of the muscle seems related to mortality.

**Acknowledgements:** The elderly EXERNET multi‐centre study has been supported by IMSERSO (104/07 and 147/2011), University of Zaragoza (UZ 2008‐BIO‐01), Centro Universitario de la Defensa de Zaragoza (UZCUD2016‐BIO‐01), Ministerio de Economía, Industria y Competitividad (DEP2016–78309‐R), Biomedical Research Networking Center on Frailty and Healthy Aging (CIBERFES) and FEDER funds from the European Union (CB16/10/00477). The authors are also grateful to all the volunteers and to the Community Center for Seniors Pedro Laín Entralgo (Zaragoza), whose cooperation and dedication made this study possible.

**2-16**

**Prevalence of sarcopenia in elderly residents in the urban and rural area of the south region of Brazil**

Letícia Mazocco^1^, Maria Cristina Gonzalez^2^, Thiago G. Barbosa‐Silva^3^ and Patrícia Chagas^1,4^

^1^ *Postgraduate Program in Gerontology at the Universidade Federal de Santa Maria (UFSM), Santa Maria, RS, Brazil;* ^2^ *Postgraduate Program in Health and Behavior at the Universidade Católica de Pelotas (UCPEL), Pelotas, RS, Brazil;* ^3^ *Postgraduate Program in Epidemiology of the Universidade Federal de Pelotas (UFPel), Pelotas, RS, Brazil;* ^4^ *Department of Food and Nutrition at the Universidade Federal de Santa Maria (UFSM), Santa Maria, RS, Brazil*

**Introduction:** The sarcopenia is a syndrome characterized by the progressive and generalized loss of skeletal muscle mass and strength. The aim of this study was to evaluate the prevalence of sarcopenia in a convenience sample of elderly women submitted to bone densitometry and living in the urban and rural area in the South of Brazil.

**Methods:** This is a cross‐sectional study with elderly (over 60 years old) who performed bone densitometry. Sarcopenia was defined according to the criteria recommended by the European Working Group on Sarcopenia in Older People (EWGSOP): muscle mass was evaluated through the Dual‐energy X‐Ray absorptiometry, muscle strength was measured by using the handgrip strength, and muscular performance was assessed through the 4 m gait speed test. Sociodemographic data was evaluated through a specific questionnaire.

**Results:** A total of 205 elderly women with a mean age of 67.3 ± 5.9 years were included in the study, the majority living in rural areas (65.9%), aged 60–69 years (66.3%), Caucasian (71.2%), with 4 to 8 years of schooling (47.3%), with partner (61.5%) and retired (92.2%). The prevalence of sarcopenia was 2.4% of the total sample (5 subjects), with a significant higher prevalence in the urban area (5.1%) when compared to the rural area (0.7%), (*p* = 0.047). There was a significant association with the living area (urban × rural) and schooling (*p* < 0.001), occupation (*p* = 0.010), socioeconomic status (*p* = 0.001) and smoking (*p* = 0.006). The environment in which the elderly women lived was independently associated with sarcopenia OR = 9.561 (95%CI: 1.021–89.523) (*p* = 0.048). The prevalence of sarcopenia was significantly higher in the urban elderly than in rural women. After multivariate analysis, the residence was still independently associated with sarcopenia.

**Conclusions:** In a sample of elderly women of the south region of Brazil, 2.4% of the total sample presented sarcopenia. Residence was independently associated with sarcopenia, showing a greater chance of sarcopenia in the urban elderly.

**2-17**

**Gender difference in anemia association with physical function in community‐dwelling Korean elders: results from the Korean Longitudinal Study on Health and Aging (KLoSHA)**

Hoon Hoon Lee^1^, Seung Yeol Lee^2^, Nam‐Jong Paik^1,3^ and Jae‐Young Lim^1,3^

^1^ *Department of Rehabilitation Medicine, Seoul National University Bundang Hospital, Seongnam, South Korea;* ^2^ *Department of Rehabilitation Medicine, Soonchunhyang University Bucheon Hospital, Soonchunhyang University College of Medicine, Bucheon, South Korea;* ^3^ *Department of Rehabilitation Medicine, Department of Rehabilitation Medicine, Seoul National University College of Medicine, Seoul, South Korea*

**Introduction:** Anemia is common in old age, and the prevalence increases with aging. It is known that the physical performance declines with aging, and anemia is associated with the frailty, but whether gender factor affects functional decline is still controversial. The aim of this study is to find out the different associations of anemia with muscle strength and physical performance between old‐men and old‐women.

**Methods:** We recruited baseline data from a population‐based cohort study on old‐aged Koreans, known as ‘the Korean Longitudinal Study on Health and Aging’ (KLoSHA). A total of 542 people aged 65 years and above were included. Data regarding age, gender, hemoglobin level, body weight, height, body mass index (BMI), physical activity score, muscle strength, muscle mass, pain related dysfunction (WOMAC‐K), depressive symptoms (GDS‐K), global cognition (MMSE), comorbid conditions and physical performance measure with short physical performance battery (SPPB) were included and analyzed.

**Results:** Skeletal muscle mass, strength and SPPB were highly associated with anemia. SPPB of old‐women with anemia was lower than those without anemia (7.76 ± 2.47 vs 8.93 ± 2.53), but there's no definite difference in strength whether anemia presents or not (43.1 ± 19.5 Nm vs 48.4 ± 16.9 Nm). In contrast, old men with anemia and without anemia showed similar SPPB (9.44 ± 2.19 vs 9.95 ± 2.26), but the strength of men with anemia was significantly lower than that without anemia (62.4 ± 22.8 Nm vs 79.8 ± 27.1 Nm). In the multivariate linear regression analysis, SPPB of both men and women with anemia related with only WOMAC‐K, while one without anemia has multiple related variables with such as MMSE, Age and physical activity other than WOMAC‐K.

**Conclusions:** We found out that anemia associated differently with muscle strength and physical performance between old men and old women. Additionally, managing pain along with treatment of anemia in both old men and women may be key source in developing model for promoting better health.

**2-18**

**Sarcopenia, obesity and metabolic syndrome**

Gloria Gabriela Peña Ordóñez^1^, Lilia Patricia Bustamante Montes^2^, Ninfa Ramirez Duran^3^ and Alfonso José Cruz Jentoft^4^

^1^ *Universidad Autonoma del Estado de Mèxico, Toluca, 50180, Mexico;* ^2^ *Universidad Autonoma de Guadalajara;* ^3^ *Universidad Autonoma del Estado de Mexico;* ^4^ *Hospital Universitario Ramon y Cajal*

**Introduction:** Sarcopenia is a geriatric syndrome that increases the risk of falls and severe fractures, physical dependence and death. The relationship between obesity, muscle and its function, as well as the effect on its metabolism are important to understand sarcopenia because the body fat affects the metabolism and muscle. In Mexico over 70% of the adult population is overweight. The objective of this study is to evaluate the association between sarcopenia, obesity and metabolic syndrome.

**Methods:** Observational, analytical, prospective study of cases and controls incident in 2016 and 2017. Sampling is done for convenience in subjects older than 60 years of the Geriatric Care Clinic (ISEM). Clinical records were reviewed to obtain biochemical and sociodemographic data; diagnostic tests for Sarcopenia (percentage of bioelectrical impedance muscle mass, hand grip and short battery of physical tests, SPPB) were performed.

Data were analyzed using non‐conditional multiple logistic regression models to obtain the odds ratios (OR), (confidence intervals calculated at 95%).

**Results:** 97 patients were recruited, of which 62 were cases and 35 controls. The higher weight was found in controls (72.9 kg DE +/− 11.4 vs. to 68.8 kg DE +/− 12.2). However, mean body mass index (BMI) and fat percentage were higher in cases than in controls. In cases with severe sarcopenia, it was observed more fat than muscle (mean of 8.6 kg).

There was a cut of the total patients (35 cases/35 controls) to obtain preliminary results, founding that for each unit that increase the fat percentage, the risk to sarcopenia increase 30%. The rest of the 27 controls will be added to the study in August.

**Conclusions:** Excess body fat reduces the quality of muscle function, especially strength, probably by fat infiltration in the muscle. However sarcorpenia in elderly is related with overweight and desnutrition.

**2-19**

**Elderly quilombolas: prevalence of sarcopenia using algorithm proposed by the European working group on sarcopenia in older people**

Luiz Sinésio Silva Neto, Margô Gomes de Oliveira Karnikowski, Neila Barbosa Osório, Leonardo Costa Pereira, Liana Barbaresco Gomide and João Paulo Chieregato Matheus

*University of Federal Tocantins, Palmas, Brazil*

**Introduction:** Sarcopenia is considered a geriatric syndrome. Currently, there is no agreed definition of sarcopenia, so it is still a challenge to establish the actual prevalence of sarcopenia in the elderly in different races/ethnicities, especially in elderly quilombolas.

**Objective:** Identify sarcopenia in the elderly living in maroon settlement using the algorithm proposed by the European Working Group on Sarcopenia in Older People.

**Patients and Methods**: This is a cross‐sectional study with 70 participants (SD 65.58 ± 6.67 years) men and women living in the Quilombo communities called Malhadinha and Córrego Fundo, located in the city of Brejinho Nazaré‐Tocantins‐Brazil. For the diagnosis of sarcopenia we used the recommendations proposed by the European Working Group on Sarcopenia in Older People. Muscle mass was analyzed by the Dual‐energy X‐ray absorptiometry and the handgrip strength by hand dynamometer. The physical performance was analyzed using the walking speed test. We used the SF‐36 questionnaire to analyze the quality of life.

**Results**: We identified a prevalence of sarcopenia of 10% in the sample. The sarcopenic individuals were classified as low handgrip strength. All individuals in the sample had adequate physical performance.

**Conclusions**: We conclude that the identification of the prevalence of sarcopenia in the elderly maroons was high. The algorithm proposed by the European Working Group on Sarcopenia in Older People had clinical applicability in the study population.

**2-20**

**Association between sarcopenia and quality of life in quilombola elderly in Brazil**

Luiz Sinésio Silva Neto, Margô Gomes de Oliveira Karnikowski, Neila Barbosa Osório, Leonardo Costa Pereira, Liana Barbaresco Gomide and João Paulo Chieregato Matheus

*University of Federal Tocantins, Palmas, Brazil*

Sarcopenia has a direct and indirect impact on the quality of life of elderly populations of different races and ethnicities. No study has yet analyzed these variables in populations of elderly people of the “quilombola” ethnic group.

**Objective:** We aimed to verify the association between sarcopenia and quality of life in quilombola elderly using the Baumgartner and the EWGSOP criteria.

**Methods:** This was a cross‐sectional study of 70 male and female participants. Quality of life was evaluated using the multidimensional SF‐36 of the Medical Outcomes Study. Sarcopenia was diagnosed according to the Baumgartner cutoff for appendicular skeletal muscle mass and the criteria recommended by the EWGSOP. Muscle mass and fat mass percentages were analyzed by DXA, while handgrip strength (HGS) was evaluated using a hand‐held dynamometer. Physical performance was assessed through a gait speed test.

**Results:** The prevalence of sarcopenia was 15% according to the Baumgartner cutoff and 10% according to EWGSOP criteria. Quilombola elderly classified as physically active or very active were at least six times less likely to develop sarcopenia than those classified as irregularly active or sedentary. HGS was negatively associated with a diagnosis of sarcopenia according to both sets of criteria. Subjects with sarcopenia reported lower scores than those without the condition on the physical role functioning and bodily pain domains of the SF‐36.

**Conclusion:** In this sample of quilombola elderly, quality of life was negatively associated with sarcopenia, regardless of the classification criteria used. Additionally, the results showed that diagnostic criteria for sarcopenia should include reductions in lean mass in addition to measures of functioning and physical performance because some subjects showed the former symptom without any alteration of the latter two variables.

**2-21**

**Quantitative assessment of muscle in cats using ultrasound**

Lisa M. Freeman, James Sutherland‐Smith, Charles O. Cummings and John E. Rush

*Cummings School of Veterinary Medicine, Tufts University, North Grafton, MA, USA*

**Background and Aims:** Cachexia occurs in cats with naturally occurring heart failure, kidney disease, cancer, and other chronic diseases. Muscle loss can be assessed subjectively using a muscle condition score, but is difficult to quantify in cats as CT and DEXA require general anesthesia. Therefore, non‐invasive methods for quantifying muscle loss are needed. We previously quantified an ultrasound technique of muscle normalized with vertebral size in dogs. The goal of this study was to assess this technique in cats.

**Methods:** Thirty healthy pet cats between 1 and 6 years of age were enrolled in the study. All cats were neutered and in ideal body condition. Transverse ultrasound images of epaxial muscle height (mean, 3 measurements each side) were obtained at the 13th thoracic vertebra using a 12–5 Mhz linear transducer. Fourth thoracic vertebral (T4) length was measured from a thoracic radiograph and the ratio of the epaxial muscle height to T4 length was calculated and compared to body weight using the Pearson correlation coefficient.

**Results:** One cat was identified to have a cardiac murmur, so 29 cats completed the study. Mean body weight was 5.05 ± 1.40 kg (range, 2.23–8.05 kg). Mean epaxial muscle height = 1.27 ± 0.13 cm which was significantly correlated with body weight (*r* = 0.65; *P* < 0.001). The ratio of epaxial muscle height to T4 length was not correlated with body weight (*r* = −0.18; *P* = 0.34). The range of values for the ratio of epaxial muscle height to T4 length in healthy pet cats was 0.93–01.55.

**Conclusions:** Since the ratio of epaxial muscle height to T4 was independent of body weight, it appears to be a good way of normalizing muscle size across cats of different body weights. Studies assessing this method in cats of different levels of adiposity and in those with cachexia and sarcopenia are warranted.

**2-22**

**Decreased muscle CT attenuation in old dogs**

James Sutherland‐Smith, Dana Hutchinson and Lisa M. Freeman

*Cummings School of Veterinary Medicine, Tufts University, North Grafton, MA, 01536, USA*

**Background and Aims:** Sarcopenia has been documented in aging dogs by CT, DEXA, and ultrasound. In addition to reductions in muscle mass, qualitative changes in muscle also occur during aging in humans, but changes in muscle density have not been documented in dogs. Therefore, the objective of this study was to measure muscle CT attenuation of healthy young and old dogs.

**Methods:** Healthy young (1–5 years old) and old (>8 years old) pet Labrador Retrievers of optimal body weight were eligible for the study. A CT was performed under mild sedation, and the mean muscle attenuation of the left epaxial (paravertebral) muscles over the 13th thoracic vertebral body was measured. To determine the mean values, multiple freehand regions of interest were drawn on the axial images and histogram analysis was performed.

**Results:** Nine young dogs (5 female, 4 male; mean age = 2 ± 1 years) and 11 old dogs (9 female, 2 male; mean age = 9 ± 1 years) were enrolled. Body weight was not different between groups [mean weight for all dogs = 30.7 ± 5.1 kg). Mean CT attenuation of the epaxial muscles was significantly lower in old compared to young dogs (*P* = 0.005). There was a significant negative correlation between CT attenuation and age (*r* = −0.74, *P* < 0.001).

**Conclusions:** Qualitative and quantitative muscle changes occur in aging in both dogs and humans. Additional studies evaluating functional muscle changes in this canine model are warranted, as are studies to evaluate potential benefits of exercise and nutritional modifications.

**2-23**

**Comparative inpatient outcome of fragility fracture integrated rehabilitation management between sarcopenic and non‐sarcopenic patients after hip fracture**

Seung‐Kyu Lim^1^, Sang Yoon Lee^2^, Jae Won Beom^3^ and Jae‐Young Lim^1^

^1^ *Department of Physical Medicine & Rehabilitation, Seoul National University Bundang Hospital, Seoul National University College of Medicine, Bundang, South Korea;* ^2^ *Department of Physical Medicine & Rehabilitation, Seoul Metropolitan Government Seoul National University Boramae Medical Center, Seoul, South Korea;* ^3^ *Department of Physical Medicine & Rehabilitation, Chung‐Ang University College of Medicine, Seoul, South Korea*

**Introduction:** Hip fracture and sarcopenia are critical causes of mortality and loss of function in elderly patients, and comprehensive rehabilitation is important for preventing disability and maintaining health. This study evaluates the effects of Fragility Fracture Integrated Rehabilitation Management (FIRM) on functional outcome in sarcopenic and non‐sarcopenic patients in inpatient care following hip fracture surgery.

**Methods:** Patients over 65 years who underwent hip surgery for fragility hip fracture at two university hospitals in Korea from July 2016 to May 2017 were entered the prospective study. After surgery, all patients received FIRM of advanced physical, occupational therapy and education for 2 weeks. The patients were divided into two groups according to the presence of sarcopenia based on Asian Working Group for Sarcopenia (AWGS) criteria. The demographic and functional characteristics were analyzed before and after FIRM to evaluate the short‐term outcome during inpatient care.

**Results:** Overall, 56 patients were eligible for the study and patients satisfied the criteria of sarcopenia were 18 (32.1%). There were no significant differences in premorbid ambulatory status between the groups. Berg Balance Scale (BBS), Modified Rivermead Mobility Index (MRMI) and Korean Instrumental Activities of Daily Living were significantly different between the groups at baseline. KOVAL, MRMI, BBS, Mini Mental State Examination and Modified Barthel Index were significantly improved, and there were no differences in the change between in the both groups after FIRM, but functional ambulatory category was significantly more improved in non‐sarcopenic group.

**Conclusions:** The functional level of hip fracture patients with sarcopenia was lower than that of the patients those without sarcopenia. FIRM may be effective for short‐term functional recovery in elderly patients who suffered fragility hip fracture with or without sarcopenia.

**2-24**

**Appendicular skeletal muscle mass index adjusted by height correlated more practically with burn size in muscle wasting of post burn**

Yoon Soo Cho, So Young Joo and Chong Hoon Seo

*Department of Rehabilitation Medicine, Hallym University, College of Medicine, Seoul, South Korea*

**Introduction:** Severe burns are inevitably accompanied by a hypermetabolic and hypercatabolic response. These result in a loss of skeletal muscle mass, bone mineral mass and fat mass. Appropriate appendicular skeletal muscle mass indices (ASMIs) is needed in burn patients for proper nutritional intervention. This paper aims to compare diverse ASMIs and to suggest more suitable index for burn survivors.

**Methods:** We conducted retrospective study with 162 burn patients. Appendicular skeletal muscle mass (ASM) measured by dual‐energy X‐ray absorptiometry was divided by height squared (kg/m2), weight (Wt)(%) and body mass index (BMI) to obtain ASMIs. Sarcopenia was defined as the ASMI of 1 SD below the sex‐specific mean for young Korean adults.

**Results:** The mean age of patients was 31.17 ± 5.08 years old. ASM loss was dependent on the burn percentage of total body surface (β = −0.23, *P* = 0.001) with adjusting for age, sex, height (Ht), weight(Wt) and BMI. ASM/Ht^2^ in the group of ≥20% burn surface area (BSA) was significantly lower than in <20% BSA group (*P* = 0.005), while ASM/Wt(%) and ASM/BMI were not different between two groups (*P* = 0.14, *P* = 0.36). Independent association between BSA and sarcopenia was showed in multiple logistic regression analysis after adjustment for sex, age, Ht, Wt and BMI in ≥10% BSA (adjusted ORs (AORs) = 1.23 [95% CI 0.42–3.57]), ≥20% BSA (AORs = 1.41 [0.53–3.76]) and ≥30% BSA group (AORs = 3.47 [1.26–9.06]).

**Conclusions:** This study demonstrates ASM/Ht^2^ is related more closely to burn injury. The burn groups were at higher risk for sarcopenia depending on the size of BSA, especially ≥30% BSA.

**2-25**

**Handgrip strength and falls among community‐dwelling older adults**

Beatriz Fernandes^1,3^, Alejandro Mercant‐Galán^2,3^ and Teresa Tomás^1,3^

^1^ *Escola Superior de Tecnologia da Saúde de Lisboa – Instituto Politécnico de Lisboa, Portugal;* ^2^ *Universidad de Jáen;* ^3^ *2GHRG ‐ Gerontology and Geriatric Health Research Group*

**Introduction:** Falls prevention in older adults includes early screening for fall risk; risk factor assessment and specialized intervention. Several variables such as balance, gait speed and mobility have been used to assess the risk of falling. More recently, handgrip strength has also been identified for this purpose. The aim of this study was to investigate the relationship between handgrip strength and balance, gait speed and mobility in community‐dwelling older adults.

**Methods:** A sample of 45 community‐dwelling older adults (16 M; 30F), aged 76.9 ± 8.6 was enrolled in the study. Inclusion criteria were age 65 and over; ability to walk autonomously (with or without assistive devices) and to understand and perform the tests. Participants were excluded if they had limitations interfering with the performance of tests and medical contraindications for exercise. Balance was assessed with the Berg Balance Scale (BBS), mobility with the 8‐foot‐up‐and‐go‐test, gait speed with the 4 meter walk test and handgrip strength with the hydraulic dynamometer Jamar®.

A Spearman's correlation was run to investigate whether there were associations between variables.

**Results:** A strong positive correlation was found between handgrip strength and balance (rs = 0.645, *p* = 0.000) and handgrip strength and gait speed (rs = 0.593, p = 0.000). Results from the 8‐foot‐up‐and‐go‐test (7.8 ± 3.4 s) did not revealed increased risk of falling; however, there was a strong negative correlation between mobility and balance (rs = −0.758, p = 0.000), gait speed (rs = −0.681, p = 0.000) and handgrip strength (rs = −0.632, p = 0.000).

**Conclusions:** For our participants as handgrip strength increases gait speed and balance also increase. Decreases in balance, gait speed and handgrip strength are related to mobility decline which is related to an increased risk of falling. Our results point‐out handgrip strength as a valuable measure to identify the risk of falling. Further studies with larger samples are needed to confirm these results.

IPL/2016/SFQ2017_ESTeSL

beatriz.fernandes@estesl.ipl.pt

**2-26**

**High risk of fall, fracture and rapid bone microstructure deterioration in men with poor physical performance – the prospective STRAMBO study**

Pawel Szulc, Philippe Wagner and Roland Chapurlat

*INSERM UMR 1033, University of Lyon, Hôpital Edouard Herriot, Lyon, France*

**Introduction:** Poor physical leg capacity is associated with poor bone microarchitecture *(Blaizot, Osteopor Int, 2012)* and may increase the risk of fall and fracture. We assessed the association of physical performance of lower limbs with subsequent changes in distal tibia bone microarchitecture and the risk of fall and fracture in older men.

**Methods:** A cohort of 817 men aged 60–87 was followed up prospectively for 8 yrs. At baseline, physical performance was assessed using a score based on the clinical tests (chair tests, standing with feet in side by side position with eyes open or closed, 10‐step tandem walk forward and backward). The score accounted for the ability and time needed to perform each test *(Szulc, JBMR, 2009)*. Every year, men replied to questionnaires covering incident falls and fractures. Distal tibia bone microarchitecture was assessed by high‐resolution pQCT (XtremeCT Scanco) at baseline and after 4 and 8 yrs. Statistical analyses were adjusted for relevant confounders.

**Results:** Compared with men having good physical performance (best quartile), men with poor performance had greater decrease in cortical thickness (−2.05 ± 0.19 vs. −0.82 ± 0.11%/yr, *p* < 0.001) and cortical density (−0.79 ± 0.06 vs. –0.42 ± 0.03%/year, p < 0.001). Poor physical performance was associated with greater decrease in central trabecular density and trabecular thickness (p < 0.001). Compared with men having good physical performance, men with poor physical performance had higher risk of recurrent falls (≥1 fall/year, *n* = 65, HR = 2.3, 95%CI: 1.1–5.1, *p* < 0.05) and of falls requiring hospitalization (*n* = 71, HR = 3.9, 95%CI: 1.7–9.0, *p* < 0.005). Men having poor physical performance had higher risk of non‐spine fracture (*n* = 61, HR = 2.9, 95%CI: 1.2–7.1, *p* < 0.05).

**Conclusions:** In older home‐dwelling men, poor physical performance of the lower limbs was associated with greater decline in distal tibia bone microarchitecture, higher risk of fall and higher risk of non‐spine fracture.

**2-27**

**Utility of phase angle as marker of low muscle mass, impaired muscle function and muscle quality in colorectal cancer patients**

Nilian Carla Silva Souza^1,2^, Maria Cristina Gonzalez^3^, Renata Brum Martucci^1,2^, Viviane Dias Rodigues^2^, Nivaldo Barroso de Pinho^2^ and Carla Maria Avesani^1^

^1^ *Rio de Janeiro State University – Rio de Janeiro, Brazil;* ^2^ *Brazilian National Cancer Institute – Rio de Janeiro, Brazil;* ^3^ *Catholic University of Pelotas – Pelotas, Brazil*

**Introduction:** Cancer is a catabolic disease that leads to muscle loss, impaired muscle function and quality and, ultimately, to sarcopenia. Phase angle (PA) is a well‐known marker of muscle mass, but its applicability as a marker of impaired muscle function and muscle quality in oncologic patients has not yet been investigated. Therefore, we aimed to investigate the association between PA with measurements of skeletal muscle mass, muscle function and muscle quality in colorectal cancer patients.

**Methods:** This study included 194 colorectal cancer patients (age: 60 ± 11 years; 58% men). The skeletal muscle mass (SMM) and skeletal muscle attenuation were assessed by computed tomography (CT) at the third lumbar vertebra. Muscle quality was assessed by skeletal muscle gauge (SMG) obtained as SMM × skeletal muscle attenuation, where high SMG indicates good muscle quality. Muscle function was assessed by handgrip strength (HGS) and gait speed (GS). The association between PA and the variables studied were assessed by Spearman/Pearson's test. In order to investigate the association between SMM, SMG, HGS and GS with PA, after adjustments for confounding variables (sex, age and BMI), linear regression models were constructed.

**Results:** Most patients (62%) were overweight and obese (BMI ≥ 25 kg/m^2^). The PA was significantly associated with BMI (*r* = 0.36), with SMM (*r* = 0.66), SMG (*r* = 0.68), HGS (*r* = 0.53) and with GS (*r* = 0.32). In the regression models, the PA remained as a significant and independent predictor (p < 0.05) of SMM (r^2^ = 0.75), SMG (r^2^ = 0.70), HGS (r^2^ = 0.62) and GS (r^2^ = 0.16).

**Conclusions:** PA was independently associated with skeletal muscle mass, muscle function and muscle quality in colorectal cancer patients. These results suggest that PA is a marker muscle mass, function and quality in this subset of patients.

**2-28**

**Low muscle radiodensity is associated with pre‐existing comorbidities in early stage colorectal cancer patients**

Jingjie Xiao^1^, Bette J. Caan^2^, Erin Weltzien^2^, Elizabeth M. Cespedes Feliciano^2^, Candyce H. Kroenke^2^, Jeffrey A. Meyerhardt^3^, Vickie E. Baracos^4^, Marilyn L. Kwan^2^, Adrienne L. Castillo^2^ and Carla M. Prado^1^

^1^ *Human Nutrition Research Unit, Department of Agricultural, Food and Nutritional Sciences, University of Alberta, Edmonton, AB, Canada;* ^2^ *Division of Research, Kaiser Permanente Northern California, Oakland, CA;* ^3^ *Department of Medical Oncology, Dana Farber Cancer Institute, Harvard Medical School, Boston, MA;* ^4^ *Department of Oncology, University of Alberta Cross Cancer Institute, Edmonton, AB, Canada*

**Introduction:** Comorbidities and computerized tomography (CT)‐measured muscle abnormalities are both common in cancer patients and evident in diseases such as diabetes and obesity. This is the first study to examine the association between comorbidities and muscle abnormalities in patients with colorectal cancer (CRC).

**Methods:** This cross‐sectional study included 3051 patients with stage I‐III CRC. Muscle abnormalities were defined as low skeletal muscle mass index (SMI) or low skeletal muscle radiodensity (SMD) quantified using diagnostic CT images. Charlson comorbidities were ascertained. Chi‐square tests were used to compare the prevalence of comorbidities by the presence/absence of each muscle abnormality. Logistic regression analyses were performed to evaluate which comorbidities predicted muscle abnormalities adjusting for age, sex, body mass index, weight change, stage, race/ethnicity, and smoking.

**Results:** Mean age was 63 years; 50% of patients were male. The prevalence of low SMI and low SMD were 43.1% and 30.2%, respectively. Comorbidities examined were more prevalent in patients with low SMD than in those with normal SMD (58.6% vs. 34.4%, *p* < 0.001), and most remained significant predictors of low SMD, including myocardial infarction (odds ratio [OR] = 1.82, *p* = 0.017), congestive heart failure (OR = 3.34, p < 0.001), peripheral vascular disease (OR = 2.20, p < 0.001), diabetes with or without complications (OR = 1.61, *p* = 0.008; OR = 1.47, *p* = 0.003, respectively) and renal disease (OR = 2.23, p < 0.001). No comorbidities were associated with low SMI except for diabetes with complication which was associated with a lower likelihood of low SMI (OR = 0.64, *p* = 0.007).

**Conclusions:** Prevalence of muscle abnormalities was high in early stage CRC. Pre‐existing comorbidities were most commonly associated with low SMD, suggestive of a potential shared mechanism between fat infiltration into muscle and each of these comorbidities.

**2-29**

**Relation between cachexia, patient‐generated Subjective Global Assessment and hand grip strength in patients with advanced cancer in palliative care unit in Brazil**

Nathália Masiero Cavalcanti de Albuquerque^1,2^, Juliana Rodrigues^1^, Emanuelly Varea Maria Wiegert^1^, Larissa Calixto‐Lima^1^, Livia Costa de Oliveira^1^, Mayane Marinho Esteves Pereira^1^ and Wilza Arantes Ferreira Peres^2^

^1^ *Palliative Care Unit, National Cancer Institute José Alencar Gomes da Silva (INCA), Rio de Janeiro, RJ, Brazil;* ^2^ *Federal University of Rio de Janeiro (UFRJ), Rio de Janeiro, RJ, Brazil*

Cachexia is frequently observed in advanced cancer patients, and it is associated with muscle mass loss and maybe strength. Therefore, we aimed to correlate cancer cachexia with Subjective Global Assessment Short Form (PG‐SGA SF) and hand grip strength (HGS) in patients with palliative care. This is an observational study comprising 525 patients attended for the first time, presenting different locations of tumor. Cachexia was defined by Fearon *et al*. (2011) criteria: 1) weight loss >2% in 6 months (**A**) + body mass index (BMI) <20 kg/m^2^; or 2) A + Mid‐upper arm muscle area (low muscle mass) <32 cm^2^ for male and <18 cm^2^ for female. Low muscle strength was characterized by HGS <20 kg for female and <30 kg for male. Associations between clinical and nutritional parameters with cachexia were tested by univariate and multivariate regressions.

**Figure 1:**
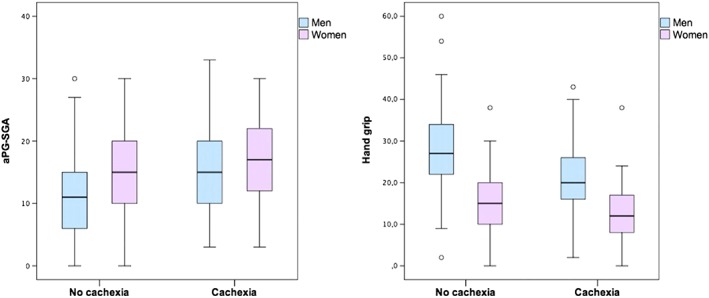
Box‐plots of abridged Patient‐Generated Subjective Global Assessment (aPG‐SGA) and Handgrip Strength compared to cancer cachexia in patients treated at a Palliative Care Unit in the city of Rio de Janeiro‐ Brazil (*n* = 525). 1
**Note: aPG‐SGA: No cachexia ≠ Cachexia (Male, p‐value < 0.001; Female, p‐value = 0.001).**

Patients presented a median age of 63 (54;72, IQR) years, 57.5% were female. Half of then presented *Karnofsky Performance Status* (KPS) < 50% and 60% had albumin <3.5 g/dl and CRP ≥10 mg/dl. Low BMI and cachexia was observed in 37% and 61% of the patients, respectively. Reduced HGS in 79% of women and 74% of men. Patients with cachexia had worst nutritional status by PG‐SGA SF and lower HGS compared with those without (Figure 1). In the univariate regression gastrintestinal tract tumor, CRP and KPS <50%, albumin (<3.5 g/dL), HGS and PG‐SGA SF (≥9) presented an elevated Odds Ratio (OR) for cachexia (*p* < 0.02). In the multivariate analysis PG‐SGA (≥9) presented the highest OR (2.43 [CI 95%: 1.52; 3.90]) against gender, GI tract tumor, and HGS. In conclusion, nutritional risk by PG‐SGA SF, low muscle strength and GI tract tumor, but not KPS were associated with an increased odds to present cachexia.

2Hand grip strength: No cachexia ≠ Cachexia (Male, p‐value <0.001; Female, p‐value = 0.003).

**2-30**

**Association of sarcopenia and survival with different diagnostic methods in patients with advanced cancer in Palliative Care Unit in Brazil**

Nathália Masiero Cavalcanti de Albuquerque^1,2^, Juliana Rodrigues^1^, Emanuelly Varea Maria Wiegert^1^, Larissa Calixto‐Lima^1^, Livia Costa de Oliveira^1^, Mariana dos Santos Campelo Queiroz^1^ and Wilza Arantes Ferreira Peres^2^

^1^ *Palliative Care Unit, National Cancer Institute José Alencar Gomes da Silva (INCA), Rio de Janeiro, RJ, Brazil;* ^2^ *Federal University of Rio de Janeiro (UFRJ), Rio de Janeiro, RJ, Brazil*

Sarcopenia has been evaluated by different methods in patients with advanced cancer. Thus, the objective of this study is to describe the association of sarcopenia with survival by different methods in patients with advanced cancer in palliative care.

This is an observational and prospective study including 342 patients [age: 63 (55; 72, IQR) years; 55.8% female; 65.5% with KPS ≥50%]. Survival time was established by the time of inclusion until 90 days of follow‐up. Sarcopenia was define by patients with reduced muscle mass and strength, where low muscle mass: 1) Mid‐upper arm muscle area (MUAMA) <32 cm^2^ for male and <18 cm^2^ for female; 2) Calf circumference (CC) < 31 cm for both genders; and 3) Appendicular skeletal muscle mass (ASM) described by Baumgartner (1998) <7.26 kg/m^2^ for male and <5.45 kg/m^2^ for female. Low muscle strength was defined by hand grip strength (HGS) <30 kg for male and <20 kg for female. Kaplan–Meier was used to calculate survival and Log‐rank test to evaluate differences between then. The association between sarcopenia and survival was evaluated by hazard ratio (HR) and 95% confidence interval (CI) in a crude univariate analysis and multivariate COX regression adjusted for co‐founders, including parameters with p‐value <0.02 in the previous analysis.

Prevalence of sarcopenia was 86%, 32.2% and 36.8%, for ASM, MUAMA and CC, respectively. Patients with sarcopenia showed shorter survival for those for all three methods. In the univariate analysis, patients with sarcopenia had a significant worse survival for ASM (HR [95%CI]: 1.78 [1.29; 2.46]), MUAMA (1.92 [1.42; 2.60]) and CC (1.89 [1.41; 2.53]). In the Multivariate analysis, MUAMA (1.55 [1.11; 2.15]) and CC (1.50 [1.08; 2.07]) remained significant. In conclusion, our data suggest that patients with sarcopenia have reduced survival, especially, when MUAMA and was used as criteria method.

**2-31**

**Grip strength as an important part of the diagnostic criteria for sarcopenia in advanced cancer**

Guro Brigitte Stene^1,3^, Trude R. Balstad^2,3^, Barry J.A. Laird^2,4^, Neil Jones^7^, Asta Bye^5,6^, Kenneth Fearon^7^, Stein Kaasa^2,3^ and Tora S. Solheim^2,3^

^1^ *Dept. of Neuromedicine and movement science, Faculty of Medicine, Norwegian University of Science and Technology (NTNU), Olav Kyrres gt. 10, Trondheim, N‐7491, Norway;* ^2^ *European Palliative Care Research Centre (PRC), Department of Cancer Research and Molecular Medicine, Faculty of Medicine, Norwegian University of Science and Technology (NTNU), Olav Kyrres gt. 10, Trondheim, N‐7491, Norway;* ^3^ *Cancer Clinic, St. Olavs Hospital, Trondheim University Hospital, Olav Kyrres gt. 10, Trondheim, N‐7491, Norway;* ^4^ *Edinburgh Cancer Research Centre, University of Edinburgh, Crewe Road, Edinburgh, EH4 2XR, UK;* ^5^ *Regional Advisory Unit for Palliative Care, Department of Oncology, Oslo University Hospital, Oslo, Norway;* ^6^ *Department of Nursing and Health Promotion, Faculty of Health Sciences, Oslo and Akershus University College of Applied Sciences, Oslo, Norway;* ^7^ *Department of Surgery, School of Clinical Sciences, University of Edinburgh, Little France Crescent, Edinburgh, EH16 4SA, UK*

**Background**: Patients with advanced cancer are at risk of developing sarcopenia that can impede a positive outcome of chemotherapy treatment. In relation to aging, classification of sarcopenia should include assessment of both muscle mass and muscle strength; however, in cancer, classification is usually based on assessment of muscle mass alone. The addition of muscle strength assessment to classify sarcopenia also in cancer patients could improve precision of diagnosis and better predict clinical outcomes. This study aims to determine prevalence of sarcopenia and examine the association between low skeletal muscle mass and grip strength in patients with advanced cancer.

**Methods:** This is a cross sectional study based on data collected from patients newly diagnosed with advanced stage lung and pancreatic cancer starting chemotherapy. Muscle mass was measured from CT‐images as cross sectional area (cm^2^) of the sum of skeletal muscles at the third lumbar vertebral level. Skeletal muscle index, SMI (cm^2^/height, m^2^) was calculated and cut‐offs used were <52.4 cm^2^/m^2^ for men; <38.2 cm^2^/m^2^ for women. Grip strength (kg) was assessed by a handheld dynamometer using the mean value of three test trials and cut‐offs were <40 kg for men and <30 kg for women.

**Results:** The sample consisted of 45 patients (26 men; 19 women) aged 35–76 years (mean 60 years) with a performance score (Karnofsky) of 80 (range 70–100). SMI (mean 45.5 ± 8.2 cm^2^/m^2^) was strongly correlated to low grip strength in this population (*r* = 0.567; *p* < 0.01). The prevalence of sarcopenia based on SMI alone was 60%. When grip strength was used in combination with SMI to diagnose sarcopenia, the prevalence was 31%.

**Conclusions:** Assessment of grip strength is a simple tool that could be considered an important part of the classification of sarcopenia in patients with advanced cancer. Further studies are warranted to determine cut‐offs for grip strength associated with important clinical outcomes in advanced cancer.

**2-33**

**The impact of changes in skeletal muscle mass (SMM) on changes in quality of life (QoL) in metastatic colorectal cancer (mCRC) patients**

Jeroen W.G. Derksen^1^, Sophie A. Kurk^1^, Petra H.M. Peeters^1^, Bram Dorresteijn^2^, Marion Jourdan^2^, Cornelis J.A. Punt^3^, Miriam Koopman^1^ and Anne M. May^1^

^1^ *University Medical Center Utrecht, Utrecht, The Netherlands;* ^2^ *Nutricia Research, Utrecht, The Netherlands;* ^3^ *Academic Medical Center, Amsterdam, The Netherlands*

**Introduction:** Increasing evidence indicates that low SMM is associated with poor outcomes in various cancers, including mCRC. We recently showed, using data of the randomized phase III CAIRO3 study (Lancet, 2015), that skeletal muscle loss was associated with poor survival during first line maintenance treatment with capecitabine + bevacizumab (CAP‐B) or observation (ASCO, 2017), after induction treatment with capecitabine + oxaliplatin + bevacizumab. The impact of SMM change on QoL is not yet known. Here, we investigate the association between change of SMM and concomitant QoL changes in mCRC patients.

**Methods:** Patients were followed during CAP‐B or observation until first progression of disease (PD1). Routine CT scans were analyzed for SMM (from skeletal muscle cross‐sectional area at L3). Change in SMM was measured continuously and categorized into loss (>2%), stable (≤2% loss‐ ≤ 2% gain), and gain (>2%). Multiple linear regression models were applied to study the association between change in SMM and concomitant change in QoL (EORTC‐QLQ‐C30v.3), adjusted for relevant confounders.

**Results:** 221 patients were included (64% male, mean age 63.5 ± 8.4 years). 24% lost, 27% maintained, and 49% gained SMM, while on average patients gained 0.5 ± 1.7 kg SMM. Mean QoL at randomization and PD1 was 74.7 ± 18.4 and 74.3 ± 16.8, respectively. One kilogram SMM increase was significantly associated with 2.7 points increase (95%CI:1.1;4.4) in QoL. Compared to patients who lost SMM, maintaining or gaining SMM was associated with clinically relevant increase of QoL (9.9 (CI:2.4;17.5) and 14.7 (CI:8.0;21.4), respectively), and role functioning (12.0 (CI:2.2; 21.7) and 17.9 (CI:9.4;26.5)), reduced fatigue (−10.0 (CI: –17.4; –2.5) and −15.0 (CI: –21.6; –8.5)), pain (−7.4 (CI: –16.7;2.0) and −16.3 (CI: –24.6; –8.1)), and appetite loss (−12.3 (CI: –22.2; –2.5) and −17.9 (CI: –26.5; –9.2)).

**Conclusions:** Stable SMM during first line CAP‐B treatment or observation was consistently associated with several clinically relevant increased QoL aspects. In addition, SMM gain was significantly associated with substantially larger positive QoL changes, underlining the importance of SMM in mCRC.

**2-34**

**Early detection of skeletal muscle atrophy using a multiple plasma‐free amino acid index in patients with advanced pancreatic cancer**

Shuichi Mitsunaga^1,2^, Michihiro Takada^3^, Sachiko Nishikawa^3^, Akira Imaizumi^3^, Makoto Ishii^3^, N. Tatematsu^4^, Masafumi Ikeda^1^ and Atsushi Ochiai^2^

^1^ *Department of Hepatobiliary & Pancreatic Oncology, National Cancer Center Hospital East, Kashiwa, Japan;* ^2^ *Research Center for Innovative Oncology, National Cancer Center, Kashiwa, Japan;* ^3^ *Institute for Innovation, Ajinomoto Co., Inc., Kawasaki, Japan;* ^4^ *Department of Rehabilitation, National Cancer Center Hospital East, Kashiwa, Japan*

**Introduction:** Loss of skeletal muscle mass (SMM) is a feature of both aging and cancer cachexia and can influence plasma free amino acid (PFAA) levels via metabolic abnormalities. The early detection of SMM loss using a multivariate index composed of PFAAs might be useful for managing patients with advanced pancreatic cancer (PCa).

**Methods:** Patients with treatment‐naïve advanced PCa were enrolled. The whole body skeletal muscle index (wSMI) and cachexia were measured using bioelectrical impedance analysis and the diagnostic criteria for cachexia of the European Palliative Care Research Collaborative at baseline and one month later. Each patient was assigned to an atrophy or a non‐atrophy group based on the change in wSMI after one month. The concentrations of 19 PFAAs were measured using liquid chromatography–mass spectrometry. An index consisting of the PFAAs at baseline was evaluated for its ability to discriminate atrophy one month later in both younger (<70 years) and older groups.

**Results:** Atrophy was observed in 49% of the 161 PCa patients. The independent risk factors for atrophy were an advanced age (odd ratio [OR]: 2.1, *P* = 0.04) and cachexia at one month (OR: 4.0, *P* < 0.01). The areas under the curves (AUCs) based on a receiver operating characteristic (ROC) curve analysis of the PFAA index for discriminating atrophy from non‐atrophy were calculated using single or multiple PFAAs. The single PFAA analyses revealed that aging affected the diagnostic ability of some PFAAs. The best AUCs for the multiple PFAA indices were 0.85 (95% confidence interval [CI], 0.74–0.96) for the older group and 0.67 (95% CI, 0.56–0.77) for the younger group.

**Conclusions:** SMM atrophy was related to aging and cachexia in patients with advanced.

**2-35**

**Muscle radiodensity is indicative of triglyceride content in skeletal muscle of cancer patients**

Amritpal S. Bhullar^1^, Ana Anoveros‐Barrera^1^, Abha Dunichand‐Hoedl^1^, Karen Martins^1^, David Bigam^2^, Todd McMullen^2^, Charles T. Putman^3,4^, Vickie Baracos^5^ and Vera Mazurak^1^

^1^ *Department of Agricultural, Food & Nutritional Science, Faculty of Agriculture Life and Environmental Sciences, University of Alberta, Edmonton, AB, Canada;* ^2^ *Department of Surgery, Faculty of Medicine and Dentistry, University of Alberta, Edmonton, AB, Canada;* ^3^ *Faculty of Physical Education and Recreation, University of Alberta, Edmonton, AB, Canada;* ^4^ *Faculty of Medicine & Dentistry, University of Alberta, Edmonton, AB, Canada;* ^5^ *Department of Oncology, Faculty of Medicine and Dentistry, University of Alberta, Edmonton, AB, Canada*

**Background:** Computed Tomography (CT) cross‐sectional imaging has been recently applied to evaluate muscle area and radiodensity in cancer patients. Studies of muscle radiodensity, reported in Hounsfield Units (HU)^1^, reveals an association with poor survival in a variety of cancer types.^2^, ^3^, ^4^, ^5^ In healthy adults, muscle radiodensity inversely associates with triglyceride content^6^ but no studies to date have reported this association in cancer patients. It is also not known whether lipid is located inside myocytes as lipid droplets or adjacent to the myocytes within adipocytes.

**Methods:** Rectus abdominis (RA) muscle biopsies were collected during surgery from patients diagnosed with gastrointestinal cancers. Skeletal muscle radiodensity was assessed by analyzing CT images at the 3rd lumbar vertebra, as well as RA specifically. Triglyceride content of muscle was analysed quantitatively using gas chromatography. RA muscles, frozen for histology, were stained for neutral lipid content using Oil Red O (ORO) to determine location of neutral lipids. Percent area of pixels with ORO stain was calculated by Volocity6.3 Software.

**Results:** Mean muscle radiodensity and RA radiodensity were inversely associated with triglyceride content (μg/mg) (*r* = −0.518, *p* = 0.023 and *r* = −0.481, *p* = 0.032, respectively). Of the total percent area of neutral lipid, mean percent area present outside myocytes was 54% compared to 46% mean percent area inside myocytes. There was no significant difference in percent area of neutral lipids present inside (42% vs 54%, *p* = 0.309) and outside (58% vs 46%, p = 0.309) myocytes in muscle with low versus high triglyceride content, respectively.

**Conclusions:** In cancer patients, overall abdominal muscle and rectus abdominis radiodensity is associated with triglyceride content of the muscle indicating that low muscle radiodensity is indicative of fatty infiltration, which appears to occur equally inside and outside of the myocyte. Future studies will enable us to understand effects of high triglyceride content within muscle of cancer patients.

**2-36**

**Characterization of advanced pancreatic cancer patients showing a decrease of the skeletal muscle mass while receiving first‐line chemotherapy**

Noriatsu Tatematsu^1^, Syuichi Mitsunaga^2,3^, Masafumi Ikeda^2^ and Atsushi Ochiai^3^

^1^ *Department of Rehabilitation, National Cancer Center Hospital East, Kashiwa, Japan;* ^2^ *Department of Hepatobiliary & Pancreatic Oncology, National Cancer Center Hospital East, Kashiwa, Japan;* ^3^ *Research Center for Innovative Oncology, National Cancer Center, Kashiwa, Japan*

**Introduction:** Loss of skeletal muscle mass (SMM) represents a clinical feature of cancer cachexia. The goal of this study was to identify the factors associated with the loss of SMM in advanced pancreatic cancer (PCa) patients receiving first‐line chemotherapy.

**Methods:** Advanced PCa patients who were scheduled to receive first‐line chemotherapy were eligible. The SMM was measured using the 3rd lumbar vertebral skeletal muscle index (L3‐SMI). Activity, symptoms, and quality of life (QOL) were prospectively evaluated at baseline and one month after the start of chemotherapy using Karnofsky Performance Status (KPS) and questionnaire (Japanese version of the M.D. Anderson Symptom Inventory). Each patient was assigned to atrophy or non‐atrophy group on the basis of the change of the L3‐SMI over one month. The differences in the assessment results between the two groups were examined at baseline and one month after the start of treatment. Landmark analysis was used for analysis of the overall survival (OS).

**Results:** The data of 159 patients (age in mean: 65.3 years, KPS in mean: 88.7, mean body mass index: 21.7 kg/m^2^, mean L3‐SMI: 41.8 cm^2^/m^2^) were evaluated. There were no differences in the baseline characteristics between the atrophy group (*n* = 78, change of L3‐SMI in mean: −3.2 cm^2^/m^2^) and non‐atrophy group (*n* = 81, +0.7 cm^2^/m^2^). At 1 month, the KPS score was lower in the atrophy group than in the non‐atrophy group (83.4 vs. 89.3, *P* < 0.01). QOL score in atrophy group were worsen to be compared with those in non‐atrophy group (3.3 vs. 2.3, *P* = 0.04). Survival analysis revealed that atrophy group showed poor OS, compared to non‐atrophy group (5.9 vs. 11.9 mo, *P* < 0.01).

**Conclusions:** Loss of skeletal muscle mass occurring during first‐line chemotherapy is related to the deterioration of activity, QOL and poor OS in advanced PCa patients.

**2-37**

**Analysis of body composition by bioimpedance in breast cancer patients at first diagnosis**

Alessio Molfino^1^, Maria Ida Amabile^1,2^, Cesarina Ramaccini^1^, Alessandro De Luca^2^, Federica Maceli^2^, Massimo Monti^2^, Filippo Rossi Fanelli^1^ and Maurizio Muscaritoli^1^

^1^ *Department of Clinical Medicine, Sapienza ‐ University of Rome, Rome, Italy;* ^2^ *Department of Surgical Sciences, Sapienza ‐ University of Rome, Rome, Italy*

**Introduction:** Obesity represents a major under‐recognized preventable risk factor for cancer development and recurrence, including breast cancer (BC). Obesity is highly prevalent in western countries, and it contributes to almost 50% of BC in older women. However, although high body mass index (BMI) may indicate overweight and obesity, often low muscle mass may be present at BC diagnosis, impacting negatively on outcomes.

We aimed at assessing body composition in BC patients at the moment of cancer diagnosis and the association between body composition and clinical parameters.

**Methods:** BC patients were enrolled before undergoing breast surgery and any other therapy. Clinical characteristics were collected, including BMI (weight/height^2^), waist circumference (cm), while muscle mass (MM) (kg) and adiposity (kg) were assessed by bioelectrical impedance analysis (InBody 770^**®**^, InBody Co, Ltd., kindly provided by Caresmed, Italy). Skeletal muscle index (SMI) was calculated as MM/height (m)^2^. Parametric and non‐parametric tests were performed, as appropriate.

**Results:** A total of 19 consecutive BC patients were studied. Age (years) was 54.4 ± 11.2. BMI was 25.2 ± 3.8 and MM was 23.3 ± 3.2. Based on SMI cut‐off values for sarcopenia, we did not find this condition in all patients (9.1 ± 1.2). Eleven patients reported involuntary body weight loss (between 3 and 6 kilograms) in the previous 6 months, whose BMI tended to be lower (23.6 ± 2.4) although not significantly different with respect to the patients without body weight loss (26.3 ± 4.3) (*P* = 0.07). Patients reporting body weight loss had a MM significantly lower with respect to patients without body weight loss (P = 0.04) and a lower phase angle value (*P* = 0.01), as well as lower SMI (*P* = 0.02).

**Conclusions:** Besides the clinical importance of the anthropometric values, body composition analyses revealed at the moment of BC diagnosis lower muscularity in patients presenting involuntary body weight loss in the previous months. These preliminary results suggest that body composition analysis should be included in the evaluation of nutritional status, since BMI and weight loss cannot discriminate the subtle changes occurring in body compartments in early phases of disease.

**2-38**

**Impact of low skeletal muscle mass and density on short and long‐term outcome after resection of stage I‐III colorectal cancer: results from a prospective multicenter observational cohort study**

Jeroen L.A. van Vugt^1^, Robert R.J. Coebergh van den Braak^1^, Zarina S. Lalmahomed^1^, Wietske W. Vrijland^2^, Jan W.T. Dekker^3^, David D.E. Zimmerman^4^, Wouter J. Vles^5^, Peter P.L.O. Coene^6^ and Jan N.M. IJzermans^1^

^1^ *Department of Surgery, Erasmus MC University Medical Center, Rotterdam, the Netherlands;* ^2^ *Department of Surgery, Sint Franciscus – Vlietland Hospital, Rotterdam, the Netherlands;* ^3^ *Department of Surgery, Reinier de Graaf Hospital, Delft, the Netherlands;* ^4^ *Department of Surgery, Elisabeth‐Twee Steden Hospital, Tilburg, the Netherlands;* ^5^ *Department of Surgery, Ikazia Hospital, Rotterdam, the Netherlands;* ^6^ *Department of Surgery, Maasstad Hospital, Rotterdam, the Netherlands*

**Background:** Preoperative low skeletal muscle mass and density are associated with increased postoperative morbidity in patients undergoing curative colorectal cancer surgery. However, the long‐term effects of low skeletal muscle mass and density remain uncertain.

**Methods**: Patients with stage I–III colorectal cancer undergoing surgery, enrolled in a prospective observational cohort study, were included. Skeletal muscle mass and density were measured on CT. Patients with high and low skeletal muscle mass and density were compared regarding postoperative complications and mortality, disease‐free survival (DFS), overall survival (OS), and cancer‐specific survival (CSS).

**Results**: In total, 816 patients (53.9% men, median age 70) were included; 50.4% had low skeletal muscle mass and 64.1% low density. The severe postoperative complication rate was significantly higher in patients with low versus high skeletal muscle and density (20.9% versus 13.6%, *p* = 0.006; 20.0% versus 11.8%, *p* = 0.003). Low skeletal muscle density was independently associated with severe postoperative complications (OR 1.89, 95%CI 1.11–3.23, *p* = 0.020). Ninety‐day mortality was higher in patients with low skeletal muscle mass and density compared with patients with high skeletal muscle mass and density (3.6% versus 1.7%, *p* = 0.091; 3.4% versus 1.0%, *p* = 0.038). No differences in DFS were observed. After adjustment for covariates such as age and comorbidity, univariate differences in OS and CSS diminished.

**Conclusions:** Low skeletal muscle mass and density are associated with short‐term, but not long‐term, outcome in patients undergoing colorectal cancer surgery. These findings recommend putting more emphasis on preoperative management of patients at risk for surgical complications, but do not support benefit for long‐term outcome.

**2-39**

**Correlations of serum creatinine with functional capacity and survival in patients with glioblastoma**

Wenli Liu, Aiham Qdaisat, Jason Yeung, Gabriel Lopez, Jeffrey S. Weinberg, Lorenzo Cohen, Eduardo Bruera and Sai‐Ching J. Yeung

*The University of Texas MD Anderson Cancer Center, Houston, TX, USA*

**Background:** Creatinine is exclusively produced by striated muscle which makes it a reliable surrogate of skeletal muscle mass under stable conditions. Studies reported that low serum creatinine was associated with increased morbidity and mortality across different race, age, and disease groups. We examined the correlations between serum creatinine and physical function as well as serum creatinine and survival in patients with glioblastoma.

**Methods:** 404 consecutive glioblastoma patients who received tumor resection between 1/1/2010 and 12/31/2014 were reviewed. Data about treatments (extent of tumor resection, radiation therapy, and use of temozolamide), medical history, vital signs, weight, height, Karnofsky Performance Status (KPS), and lab tests were collected. Charlson Comorbidity Index (CCI) was calculated with ICD‐9 codes. Bivariate associations between baseline KPS and serum creatinine, and body mass index (BMI) were analyzed using Pearson correlation coefficients. Cox regression for survival analysis was performed.

**Results:** The mean age of the population was 58 (+13) years. 64% were male. Death rate was 78%. The correlation coefficients for KPS and creatinine 0.15 (*P* = 0.003), for KPS and BMI 0.08 (*P* = 0.103). Patients with low serum creatinine (<0.6 mg/dL in female, <0.7 mg/dL in male) had shorter survival (HR = 1.65 [95% CI = 1.07–2.55], *P* = 0.023) when adjusted for known survival predictors including age, extent of tumor resection, and treatment strategies.

**Conclusions:** Serum creatinine positively correlates with functional performance status. Low serum creatinine predicts mortality in patients with glioblastoma. The unique utility of creatinine as a marker of muscle mass deserves more research and clinical attention.

**2-40**

**Identification of immune cells in the muscle of cancer patients**

Ana Anoveros‐Barrera^1^, Amritpal S. Bhullar^1^, Abha Dunichand‐Hoedl^1^, Karen Martins^1^, David Bigam^2^, Vickie Baracos^3^ and Vera Mazurak^1^

^1^ *Department of Agricultural, Food & Nutritional Science, Faculty of Agriculture Life and Environmental Sciences, University of Alberta, Edmonton, AB, Canada;* ^2^ *Department of Surgery, Faculty of Medicine and Dentistry, University of Alberta, Edmonton, AB, Canada;* ^3^ *Department of Oncology, Faculty of Medicine and Dentistry, University of Alberta, Edmonton, AB, Canada*

**Background:** Inflammation is a recognized contributor to muscle wasting^1^. Gene array analysis in human biopsies has revealed that immune cell recruitment is a major event occurring in cancer patients experiencing systemic inflammation^2^. Lymphoid and myeloid cells in the tissue influence muscle atrophy through diverse mechanisms^3^, ^4^; however, little is known about the muscle's immunological environment and its relationship to muscle loss in conditions of malignancy. Therefore, we hypothesized that features of muscle atrophy are related to a higher recruitment of immune cells in the muscle of patients with cancer.

**Methods:** Rectus abdominis biopsies and computed tomography (CT) images were collected from cancer patients (*n* = 22) at the University of Alberta Hospital, Edmonton, Canada, from July 2015 to August 2016. Frozen muscle biopsies cut in transverse sections were stained with immunofluorescence to evaluate immune cells: T cells (CD8+ and CD4+), antigen presenting cells (APCs; CD3‐CD4+), and granulocytes (CD11b+). Features of muscle atrophy were explored by evaluating histopathological components such as mean muscle fiber area (μm^2^) and computed CT images to obtain muscle cross sectional area (CSA; cm^2^). Spearman's coefficient was used to assess correlations.

**Results:** Muscle with higher CSA (cm^2^) (*r* = 0.66, *P* = 0.001) and mean fiber area (μm^2^) (*r* = 0.55, *P* = 0.008) had a larger number of T cells (CD4+ and CD8+ combined). CD8+ T cells (not CD4+ T cells) maintain a significant association with muscle CSA (*r* = 0.53, *P* = 0.012) and mean muscle fiber area (*r* = 0.49, *P* = 0.021). APCs and granulocytes were not related to any muscle feature evaluated; however, APCs were positively correlated to number of CD8+ T cells (*r* = 0.45, *P* = 0.036).

**Conclusions:** Lymphoid and myeloid cells are present in the muscle of cancer patients. In particular, T cells seem to be associated with better muscle condition. Results suggest interaction between CD8+ T cells and APCs. Further exploration will enable understanding the influence of immune cells within the muscle of cancer patients.

**2-41**

**CT‐assessed muscle abnormalities are associated with poor prognosis post colon cancer resection surgery**

Jingjie Xiao^1^, Bette J. Caan^2^, Peter D. Peng^3^, Erin Weltzien^2^, Elizabeth M. Cespedes Feliciano^2^, Candyce H. Kroenke^2^, Jeffrey A. Meyerhardt^4^, Vickie E. Baracos^5^, Marilyn L. Kwan^2^, Adrienne L. Castillo^2^ and Carla M. Prado^1^

^1^ *Human Nutrition Research Unit, Department of Agricultural, Food and Nutritional Sciences, University of Alberta, Edmonton, AB, Canada;* ^2^ *Division of Research, Kaiser Permanente Northern California, Oakland, CA;* ^3^ *Medical Center and Redwood City Medical Center, Kaiser Permanente Northern California, Oakland, CA;* ^4^ *Department of Medical Oncology, Dana Farber Cancer Institute, Harvard Medical School, Boston, MA;* ^5^ *Department of Oncology, University of Alberta Cross Cancer Institute, Edmonton, AB, Canada*

**Introduction:** Despite advanced operative techniques for resection surgery, a large number of patients with colon cancer still suffer from post‐operative morbidity. This leads to delayed subsequent therapy, prolonged hospital stay, and potentially worse prognosis. Computerized tomography (CT)‐measured muscle abnormalities are of emerging interest to surgeons due to their potential value for post‐operative risk stratification. This study investigated the independent prognostic effects of muscle abnormalities on surgical outcomes.

**Methods:** Patients diagnosed with stages I–III colon cancer from 2006 to 2011 were included (*n* = 1,715). Muscle abnormalities were defined as a low skeletal muscle index (SMI) and/or radiodensity (SMD) measured on pre‐operative CT images. Differences in demographic, clinical and surgical outcomes were compared by each muscle abnormality using independent *t*‐tests or Chi‐squared tests. Logistic regression was used to evaluate the associations of muscle abnormalities with post‐surgical length of hospitalization, any complication(s) and readmission up to 30 days post‐surgery or post‐discharge. Cox proportional hazards regression was performed to examine the effect of muscle abnormalities on 30‐day mortality.

**Results:** Mean age was 64.0 ± 11.2 years and 55.5% were female. Patients with low SMI (OR = 1.37, 95% CI 1.10–1.72) or low SMD (OR = 1.50, 95% CI 1.15–1.96) were more likely to remain hospitalized ≥7 days after surgery, and had higher risk of 30‐day mortality (low SMI: HR = 4.65, 95% CI 1.43–15.06; low SMD: HR = 3.34, 95% CI 1.08–10.30). Additionally, patients with low SMI were more likely to have at least one post‐surgical complication (OR = 1.26, 95% CI 1.02–1.55). Readmission rate was not associated with any muscle abnormality.

**Conclusions:** Muscle abnormalities were associated with poorer surgical outcomes, including longer hospitalization and a higher risk of short‐term mortality. Low SMI was associated with a higher risk of post‐surgical complications. Research should evaluate whether targeting potentially modifiable factors preoperatively, such as increasing muscle mass and/or radiodensity may improve post‐operative outcomes.

**2-42**

**Impact of sarcopenia on dose limiting toxicities (DLT) in metastatic colorectal cancer (mCRC) patients receiving palliative systemic treatment**

Sophie Kurk^1,2^, Petra Peeters^2^, Jeroen Derksen^1,2^, Rebecca Stellato^2^, Bram Dorresteijn^3^, Marion Jourdan^3^, Cornelis Punt^4^, Miriam Koopman^1^ and Anne May^2^

^1^ *University Medical Center Utrecht, Utrecht, The Netherlands;* ^2^ *Julius Center for Health Sciences and Primary Care, University Medical Center Utrecht, Utrecht, The Netherlands;* ^3^ *Nutricia Research, Nutricia Advanced Medical Nutrition, Utrecht, The Netherlands;* ^4^ *Academic Medical Center, University of Amsterdam, Amsterdam, The Netherlands*

**Background:** Increasing evidence suggests that severe skeletal muscle (SM) loss (sarcopenia) is associated with reduced overall survival (OS) in mCRC. We recently found that, using data of the randomized phase 3 CAIRO3 study (*Lancet, 2015*), SM loss was related to shorter OS during first‐line maintenance treatment with capecitabine + bevacizumab or observation. Subsequently, sarcopenia at start of more intensive reinduction treatment with oxaliplatin + capecitabine + bevacizumab (CAPOX‐B) was associated with shorter time to progression and OS (*ASCO, 2017*). As a potential risk factor for reduced survival we explored whether sarcopenia was associated with DLT during CAPOX‐B reinduction treatment.

**Methods:** CAIRO3 patients were included who received CAPOX‐B reinduction treatment. DLT were defined as any dose delay/reduction/discontinuation of systemic treatment because of reported CTCAE(v3.0) toxicities at start or during treatment. Poisson regression models, adjusted for confounders, were used to study the association between sarcopenia and DLT.

**Results:** 254 patients received CAPOX‐B reinduction treatment. 39% of patients were sarcopenic and compared to normal SM patients we found no statistically significant differences in age and sex (sarcopenic vs normal SM: mean age 63.6 ± 9.1 vs 61.9 ± 8.5 years, *p* = .20 and 39% vs 31% women, *p =* .31). BMI was significantly lower in sarcopenic patients, but patients were on average still overweight (25.9 ± 3.8 vs 27.2 ± 3.8 *p* = .01). Overall, 67% experienced ≥1 DLT. At start of CAPOX‐B, 25% already received a dose reduction and the risk of dose reduction at start was significantly higher for sarcopenic patients compared to normal SM (RR 1.8 (CI:1.08–2.90)). Despite more frequent dose reductions at start, sarcopenic patients did not have a significantly lower risk of DLT during CAPOX‐B (RR sarcopenia vs normal SM 0.86 (CI: 0.46–1.45)).

**Conclusions:** Sarcopenia was significantly associated with dose reductions at start of CAPOX‐B reinduction treatment, and not with DLT during CAPOX‐B reinduction treatment. Possible explanations for dose reductions at start might be more frequent toxicities during previous treatment including neuropathy.

**2-43**

**Muscle wasting in hemodialysis and lung cancer patients is mediated through down and up‐regulation of several proteins common to both diseases, including actors of the proteasome and the autophagy proteolytic systems**

Julien Aniort^1,2^, Cécile Polge^1^, Agnes Claustre^1^, Lydie Combaret^1^, Daniel Béchet^1^, Didier Attaix^1^, Anne‐Elisabeth Heng^1,2^ and Daniel Taillandier^1^

^1^ *Institut National de la Recherche Agronomique, UMR 1019, Human Nutrition Unit (UNH), 63122, St Genès Champanelle, France;* ^2^ *Service de Néphrologie Réanimation Médicale, Pôle Respiratoire, Endocrinologie‐Diabétologie, Urologie, Néphrologie‐Dialyse, Nutrition Clinique, Infectiologie, Réanimation Médicale, Hygiène Hospitalière (REUNNIRH), 63000, Clermont‐Ferrand, France*

**Introduction:** Muscle atrophy is frequently encountered in diseased patients. It contributes to patient's frailty and is associated with an increased risk of death. Studies using animal models suggest the involvement of the Ubiquitin Proteasome System (UPS) in renal failure‐induced muscle atrophy. However, this remains to be established in humans. Another important goal is to detect markers that may help fighting against muscle atrophy through nutritional or pharmacological strategies. Indeed, it is very difficult to counteract the increased proteolysis when it is established. Our objectives were (i) to identify the proteolytic systems activated in chronic hemodialysis (HD) or lung cancer (LC) patients, i.e. pathologies having a different etiology and (ii) to identify markers specific to the activation of muscle atrophy processes independently of the pathology per se.

**Methods:** Muscle biopsies (*n* = 7 per group) were obtained upon programmed surgery. mRNA and protein levels were determined using qRT‐PCR, immunoblotting and proteomic approaches.

**Results:** We found that the UPS and autophagy were activated in both HD and LC patients. Mass spectrometry analysis identified >1700 proteins. Main component analysis revealed 3 distinct protein expression profiles corresponding to the 3 groups studied. We identified 106 proteins that were significantly modified (decreased or increased) in both HD and LC patients compared to controls (CT). Hierarchical cluster analysis showed that expression levels of these proteins distinguished diseased (HD or LC) vs. CT patients. Orthogonal partial least square discriminant analysis confirmed these results.

**Conclusions:** We demonstrated that the UPS and autophagy were activated during long‐term disease in humans. We also found a set of proteins whose expression levels may be specific of the atrophying process. These proteins constitute potential biomarkers witnessing the activation of muscle atrophy and/or potential therapeutic targets.

**2-44**

**Body composition and sarcopenia before and after surgery for oesophageal cancer**

Ulrika Smedh, Jan Persson, Monika Fagevik Olsen and Britt‐Marie Iresjö

*University of Gothenburg, Gothenburg, Sweden*

**Background:** The occurrence of dysphagia is a well‐known feature of oesophageal cancer that may reduce caloric intake. Body composition alterations and prevalence of sarcopenia in patients with oesophageal cancer before and after surgery are not well known, and their possible consequences have been debated. The aim was to describe biometric measures including body composition, and sarcopenia, physical performance, dysphagia, as well as quality of life (QoL) in a cohort of patients with oesophageal cancer before surgery with curative intent. In addition, we aimed to investigate alterations in body composition as a consequence of the surgery at 1 and 3 months post‐operatively. Moreover, to investigate if pre‐operative biometric measures, or sarcopenia, are correlated to morbidity, length of stay, QoL or mortality.

**Methods:** An observational study was performed in a cohort of 76 patients who had oesophageal or cardia cancer and were planned for open surgery with curative intent. Demographic data and data on body composition measured with bioimpedance, working capacity (cardiac stress test), grip strength and QoL (EORTC QLQ‐C30 version 3.0) were prospectively collected from the patient history database. Data regarding dysphagia was derived from the oesophagus‐related QoL form EORTC QLQ‐OES18. Data on tumour stage and type, complications, length of stay, weight loss and nutritional state were also collected.

**Results:** Pre‐operatively the patients displayed normal BMI despite that almost 20% were sarcopenic, 86% had a lowered physical performance level and 37% of the patients were judged severely malnourished. All body composition variables except fat mass were declined up to 3 months after surgery. No pre‐operative biometric measure or QoL item was correlated with risk for complications. BMI was positively correlated to length of stay at the intensive care unit. Female sex and malnourishment showed significantly fewer ICU days. High physical performance, female sex and high global QoL score positively predicted overall survival.

**Conclusions:** Severe malnourishment was common in patients planned for oesophageal resection due to cancer in spite of normal BMI. Neither pre‐operative malnutrition (SGA C) nor sarcopenia were independent risk factors for morbidity or overall mortality in patients judged suitable for surgery. However, oesophageal resection surgery caused long lasting catabolic effects, highlighting the importance of optimal peri‐ and post‐operative nutrition.

**2-45**

**Patient's skeletal muscle quality and sarcopenic obesity are associated with postoperative morbidity after neoadjuvant chemoradiation and resection for rectal cancer**

Annefleur E.M. Berkel^1^, Joost M. Klaase^1^, Feike de Graaff^2^, Marjolein G.J. Brusse‐Keizer^3^, Bart C. Bongers^4^ and Nico L.U. van Meeteren^4,5^

^1^ *Department of Surgery, Medisch Spectrum Twente, Enschede, The Netherlands;* ^2^ *Faculty of Science and Technology, University of Twente, Enschede, The Netherlands;* ^3^ *Medical School Twente, Medisch Spectrum Twente, Enschede, The Netherlands;* ^4^ *Department of Epidemiology, School for Public Health and Primary Care (CAPHRI), Maastricht University, Maastricht, The Netherlands;* ^5^ *Health~Holland, Topsector Life Sciences and Health, The Hague, The Netherlands*

**Introduction:** This explorative retrospective study investigated the relation between skeletal muscle measurements (muscle mass, muscle quality and sarcopenic obesity), and postoperative morbidity and survival after neoadjuvant chemoradiation followed by non‐laparoscopic resection for rectal cancer.

**Methods:** Ninety‐nine consecutive patients who underwent neoadjuvant chemoradiation and surgery between January 2007 and May 2012 were identified. Skeletal muscle mass was measured as total psoas area and total abdominal muscle area at three anatomical levels using the patient's preoperative computed tomography scan. Skeletal muscle quality was measured using corresponding mean Hounsfield Units for total abdominal muscle area. Sarcopenic obesity was defined as body mass index above 25 kg·m^−2^ combined with skeletal muscle mass measures below the sex‐specific median. Postoperative complications were graded according to the Clavien‐Dindo classification.

**Results:** Twenty‐five patients (25.3%) developed a severe complication. Lower skeletal muscle quality was independently associated with overall (*p* = 0.007) and severe complications (*p* = 0.002). Sarcopenic obesity was associated with overall complications (all *p* < 0.05). No significant relations were found between skeletal muscle measurements and survival.

**Conclusions:** Skeletal muscle quality is associated with overall and severe postoperative morbidity after neoadjuvant chemoradiation and non‐laparoscopic resection for rectal cancer. Sarcopenic obesity is associated with overall complications.

**2-46**

**Functional muscle strength is associated with muscle mass in patients with esophageal cancer awaiting surgery**

Maarten A. van Egmond^1,2,3^, Marike van der Schaaf^1,2^, Eliza R.C. Hagens^5^, Hanneke W.M. van Laarhoven^4,6^, Mark I. van Berge Henegouwen^5,6^, Liesbeth B. Haverkort^7,8,9^, Raoul H.H. Engelbert^1,2^ and Suzanne S. Gisbertz^5,6^

^1^ *Department of Rehabilitation, Academic Medical Center, University of Amsterdam, Amsterdam, The Netherlands;* ^2^ *ACHIEVE, Center of Applied Research, Amsterdam University of Applied Sciences, Faculty of Health, Amsterdam, The Netherlands;* ^3^ *European School of Physiotherapy, Amsterdam University of Applied Sciences, Faculty of Health, Amsterdam, The Netherlands;* ^4^ *Department of Medical Oncology, Academic Medical Center, University of Amsterdam, Amsterdam, The Netherlands;* ^5^ *Department of Surgery, Academic Medical Center, University of Amsterdam, Amsterdam, The Netherlands;* ^6^ *Cancer Center Amsterdam, Amsterdam, The Netherlands;* ^7^ *Research Centre for Innovations in Health Care, University of Applied Sciences, Utrecht, The Netherlands;* ^8^ *Department of Clinical Epidemiology, Biostatistics and Bioinformatics, Academic Medical Center, Amsterdam, The Netherlands;* ^9^ *Department of Dietetics, Academic Medical Center, Amsterdam, The Netherlands*

**Introduction:** Decreased muscle mass and muscle strength are independent predictors of postoperative complications and poor postoperative recovery in patients with esophageal cancer. The association between muscle mass and muscle strength is not self‐evident. If the association between muscle mass and muscle strength in preoperative patients with esophageal cancer is known, physiotherapists are able to measure functional muscle strength as an early predictor for the consequences for functional performance due to decreased muscle mass and eventually sarcopenia.

**Objectives:** To investigate the association between muscle mass and functional muscle strength in patients with esophageal cancer awaiting esophagectomy before neoadjuvant chemoradiation.

**Methods:** In patients with resectable esophageal cancer eligible for surgery between March 2012 and October 2015, Computed Tomography scans were used to assess muscle mass and compared with functional strength measures (hand grip strength, inspiratory‐ and expiratory muscle strength, 30 seconds chair stands test). Pearson correlation coefficients were calculated, and associations were determined by multivariate linear regression analysis.

**Results:** 126 subjects were referred to physiotherapy from a tertiary referral center and were eligible for the study; 94 subjects were finally included for statistical analysis. Multiple backward regression analysis showed that gender (95% CI –32.4, −2.2), weight (95% CI 0.4, 1.0), age (95% CI –0.9, −0.7), non‐dominant handgrip strength (95% CI 0.2, 1.5) and inspiratory muscle strength (95% CI 0.1, 0.4) were all independently associated with muscle surface area at L3. All these variables together explained 65% of the variability (R^2^) in muscle surface area at L3 (*p* < 0.001).

**Conclusions:** This study shows an independent association between aspects of functional muscle strength and muscle mass in patients with esophageal cancer awaiting surgery, and the results could be used by physiotherapists to predict muscle mass based on functional muscle strength in preoperative patients with esophageal cancer.

**2-48**

**The addition of sarcopenia does not surpass the MELD score in predicting waiting list mortality in cirrhotic liver transplant candidates**

Jeroen L.A. van Vugt^1^, Louise J.M. Alferink^2^, Stefan Buettner^1^, Marcia P. Gaspersz^1^, Daphne Bot^3^, Sarwa Darwish Murad^2^, Shirin Feshtali^4^, Peter M.A. van Ooijen^5^, Wojciech G. Polak^1^, Robert J. Porte^6^, Bart van Hoek^7^, Aad P. van den Berg^8^, Herold J. Metselaar^2^ and Jan N.M. IJzermans^1^

^1^ *Department of Surgery, Division of HPB and Transplant Surgery, Erasmus MC University Medical Center, Rotterdam, The Netherlands;* ^2^ *Department of Gastroenterology and Hepatology, Erasmus MC University Medical Center, Rotterdam, The Netherlands;* ^3^ *Department of Dietetics, Leiden University Medical Center, Leiden, The Netherlands;* ^4^ *Department of Radiology, Leiden University Medical Center, Leiden, The Netherlands;* ^5^ *Department of Radiology, University of Groningen, University Medical Centre Groningen, Groningen, The Netherlands;* ^6^ *Department of Surgery, University of Groningen, University Medical Centre Groningen, Groningen, The Netherlands;* ^7^ *Department of Gastroenterology and Hepatology, Leiden University Medical Center, Leiden, The Netherlands;* ^8^ *Department of Gastroenterology and Hepatology, University of Groningen University Medical Center Groningen, Groningen, The Netherlands*

**Background:** Frail patients with low MELD scores may be underprioritized. Recently, low skeletal muscle mass (i.e. sarcopenia) has been identified as a risk factor for waiting list mortality and a study proposed to include sarcopenia in the MELD score (i.e. MELD‐Sarcopenia score). We aimed to investigate the association between sarcopenia and waiting list mortality and to validate the MELD‐Sarcopenia score (MELD + 10.35*Sarcopenia).

**Patients and methods:** Using the Eurotransplant registry, consecutive patients with cirrhosis listed for liver transplantation between 2007 and 2014 were identified. Survival of patients with and without sarcopenia was compared using Fine and Gray Competing Risks Analysis. Performance of the MELD, MELDNa and MELD‐Sarcopenia score was investigated using concordance (c) indices after bootstrapping crossvalidation. Variables in the multivariable analysis were selected based on the Akaike Information Criterion.

**Results:** In total, 588 patients were included with a median MELD of 14 (IQR 9–19), of which 225 (43.4%) were identified as having sarcopenia. Median waiting list survival was 5 months. Both the sarcopenia cut‐offs according to Carey and according to Martin were not significantly associated with survival in univariable analysis. The discriminative performance of the MELD‐Sarcopenia score for 5 months (c‐index 0.668) was comparable to the MELD score alone (c‐index 0.663) and the MELDNa score (c‐index 0.666). Apart from the MELD score with or without sarcopenia, other independent predictive variables were occurrence of hepatic encephalopathy before listing, and recipient albumin at the time of listing.

**Conclusions:** Sarcopenia as an addition to the MELD score did not improve prognostic ability when analysed using competing risks regression in a Dutch national cohort. Other measures of sarcopenia did not reach significance in univariable analysis.

**2-49**

**Resting Energy Expenditure (REE) of children with End Stage Liver Disease (ESLD)**

Eirini Kyrana^1^, Jane E. Williams^2^, Jonathan C.K. Wells^2^ and Anil Dhawan^1^

^1^ *King's College Hospital, London, UK;* ^2^ *Institute of Child Health, London, UK*

**Introduction:** Hypermetabolism has been described in patients with ESLD, as measured REE (mREE) ≥ 120% of predicted REE (pREE).

**Methods:** REE was quantified by indirect calorimetry. Seventeen children (mean age 7.6 yrs/ median 6.9 yrs) with ESLD had their mREE prior to having a liver transplant. Valid results were in sixteen. pREE was calculated from the Henry 2005 equations. REE was measured and predicted for thirteen healthy children (mean age 8.3 yrs/ median 8 yrs). Twelve had valid results. Children over 4 years of age from both groups had fat free mass (FFM) determined with air displacement plethysmography (BOD POD).

**Results:** The differences in mean age, mean mREE/kg of body weight (BW) and RQ between the patients and healthy controls were not significant (NS). Seven of the 16 patients (44%) were hypermetabolic, and 9/16 were normometabolic (56%). Mean RQ for both groups was 0.77.

6 of the 12 (50%) healthy controls were hypermetabolic. The mean RQ for the hypermetabolic healthy children was 0.75 and for the normometabolic 0.79 (NS). Differences of mean SD scores for BW, height and BMI between hypermetabolic and normometabolic groups of patients and healthy children did not achieve statistical significance. 8 patients and 10 healthy controls had FFM measured with BOD POD. Mean mREE/kg of FFM for the patients was 57.53 Kcal/kg and for the healthy controls 59.5 Kcal/kg (NS). Nine of the patients had their liver transplant and had mREE at a median of 10.8 months post‐transplant. The differences in mean REE/kg and RQ before and after liver transplantation were not statistically significant. REE/kg pre transplant correlated significantly with REE/kg post‐transplant (*r* = 0.832, *p* = 0.005).

**Conclusions:** mREE and the presence of hypermetabolism was not different between children with ESLD awaiting liver transplant and healthy controls. mREE/kg after transplant correlated significantly with mREE/kg before transplant.

**2-50**

**Six‐minute walk distance is superior to Karnofsky Performance Status to predict mortality in candidates for liver transplantation**

Elizabeth J. Carey^1^ and Bridgette McNally^2^

^1^ *Mayo Clinic, Phoenix, USA;* ^2^ *Midwestern University Arizona College of Ostepathic Medicine, Phoenix, USA*

**Background:** Poor functional status is associated with increased mortality in cirrhosis patients awaiting liver transplantation; however, the optimal assessment of functional status remains unknown. This study sought to determine the relationship between six‐minute walk distance (6MWD) and Karnofsky Performance Status (KPS) and their association with waitlist mortality (WLM) in liver transplant (LT) candidates.

**Methods:** Two hundred seventy‐eight consecutive patients listed for LT at a single institution were included. KPS and 6MWD were assessed at the time of LT evaluation. KPS scores were recorded as a percentage from 0 to 100, with 0 representing death and 100 representing no presence of disease. Patients were followed from time of listing until transplantation, death, removal from the waitlist or end of the study period (study period: 01/2014–03/2017).

**Results:** The mean KPS and 6MWD were 77.4 ± 13.5 and 323.6 ± 163.9 m, respectively. A mild correlation between 6MWD and KPS was demonstrated (Spearman ρ = 0.4317, *p* < 0.0001). KPS score was significantly lower in patients with 6MWD <250 meters (*p* < 0.0001). In univariate and multivariate logistic regression, there was no significant relationship between KPS score and waitlist mortality. In contrast, the 6MWD was significantly lower in patients who suffered waitlist mortality (266.1 vs 331.8, *p* = 0.05) and was the only significant predictor of WLM in multivariate logistic regression. HCC patients were shown to be less likely to die on the waitlist compared to other liver disease etiologies.

**Conclusions:** In conclusion, 6MWD is a better predictor of waitlist mortality than KPS score in candidates for LT. The addition of 6MWD as a standard assessment may help to identify patients at risk of dying on the waitlist.

**2-51**

**Sarcopenia in liver transplant due to Familial Amyloidotic Polyneuropathy (FAP): the relevance of muscle mass**

Maria Teresa Tomás^1,2^, Xavier Melo^2,3^, Élia Mateus^4^, Eduardo Barroso^4^ and Helena Santa‐Clara^2^

^1^ *Lisbon Higher School of Health Technology (ESTeSL) at Lisbon Polytechnique Institute, Lisbon, Portugl;* ^2^ *Interdisciplinary Centre for the Study of Human Performance (CIPER) at Faculdade de Motricidade Humana – Universidade de Lisboa, Portugal;* ^3^ *Ginásio Clube Português;* ^4^ *Hepatobiliopancreatic and Transplantation Centre at Hospital Curry Cabral, Lisbon, Portugal*

**Introduction:** Loss of muscle mass and function is a common occurrence in liver transplant Familial Amyloidotic Polyneuropathy (FAP) patients. Sarcopenia is associated with morbidity and mortality before and after liver transplantation. However, the ability of skeletal muscle mass to recover after transplant remains questionable and thus the importance of clinical exercise prescription.

**Methods:** Participants were 39 FAP patients aged 23–59 years, who had been submitted to a liver transplant (Tx) (22 men) between 2 and 4 months post‐tx. Sarcopenia was defined according to the International Working Group on Sarcopenia and Society of Sarcopenia, Cachexia and Wasting Disorders. Whole‐body dual x‐ray absorptiometry was used to measure body fat and lean‐soft tissue. Skeletal Muscle Index (SMI) was calculated adjusting the value of appendicular skeletal mass to the squared height. Functional aerobic capacity was assessed using the 6 min walk test (6MWT), and handgrip strength was measured on the dominant hand using a hand dynamometer.

**Results:** The prevalence of sarcopenia using SMI was 45.5% in men and 41.2% in women. A fat mass higher than 16% for men and 26% for women was found in 54.5% of men and 52.9% of women. Also 27.3% of men and 17.6% of the women could be classified as having sarcopenia with low mobility (distance < 400 m).

**Discussion and Conclusions:** Sarcopenia is common in FAP patients and a liver transplant seems to increase the prevalence, also because an aggressive medication with impact on muscle metabolism should be made longtime. More data are needed on the long‐term effects of sarcopenia after transplant, especially in light of the high rate of metabolic syndrome.

A clinical exercise prescription seems to be necessary for these patients but more studies are needed (e.g. longitudinal studies).

**2-52**

**Diagnosing sarcopenia in patients with cirrhosis, ascites and lower limb edema**

Giliane Belarmino^1^, Maria Cristina Gonzalez^2,3^, Priscila Sala^1^, Raquel Susana Torrinhas^1^, Wellington Andraus^1^, Luiz Augusto Carneiro D'Albuquerque^1^, Rosa Maria R. Pereira^4^, Valéria F. Caparbo^4^, Steven B. Heymsfield^3^ and Dan L. Waitzberg^1^

^1^ *Department of Gastroenterology (LIM 35), Surgical Division, Faculdade de Medicina da Universidade de São Paulo, Brazil;* ^2^ *Postgraduate program in Health and Behavior, Universidade Católica de Pelotas, Rio Grande do Sul, Brazil;* ^3^ *Pennington Biomedical Research Center, Baton Rouge, LA, USA;* ^4^ *Laboratory of Bone Metabolism, Rheumatology Division, Faculdade de Medicina da Universidade de São Paulo, Brazil*

**Background:** Ascites limit skeletal muscle mass assessment. We propose that sarcopenia diagnosis in cirrhosis can be improved by using dual‐energy X‐ray absorptiometry to calculate the appendicular skeletal muscle index (DXA‐ASMI), as it bypasses the abdominal compartment.

**Objective:** We evaluated whether DXA‐ASMI is influenced by ascites or lower limb edema (LLE) and can be applied to diagnose sarcopenia with prognostic value for mortality, alone or combined with non‐dominant handgrip strength (ND‐HGS).

**Design:** DXA‐ASMI and ND‐HGS values were obtained and 36 months mortality recorded in 144 male cirrhotic patients. DXA‐ASMI was calculated pre‐ and post‐paracentesis in 20 patients with ascites and compared with data from 20 matched volunteers. DXA‐ASMI values obtained from patients with and without LLE were compared. Prognostic value of DXA‐ASMI and DXA‐ASMI + ND‐HGS was tested in a final survival model adjusted for MELD and age. Survival probabilities were obtained for sarcopenia diagnosed by standard cutoffs for DXA‐ASMI and ND‐HGS values (EWGSOP) and a new cutoff calculated from our DXA‐ASMI + ND‐HGS tertiles.

**Results:** DXA‐ASMI did not differ pre‐ and post‐paracentesis, were lower in cirrhotic patients than in volunteers (*p* < 0.001), and were not influenced by LLE (mean difference = 0.30 kg/m^2^, *p* = 0,068; R^2^ = 2.40%). Mortality was influenced by DXA‐ASMI and ND‐HGS (*p*_interaction_ = 0.028). Sarcopenia diagnosed by EWGSOP was also diagnosed by our new cutoff; both predicted mortality, but the latter was more sensitive for mortality risk prediction (*p* = 0.011).

**Conclusions:** In cirrhosis, DXA‐ASMI estimates are not influenced by ascites or LLE and can identify low skeletal muscle mass for diagnosing sarcopenia with prognostic value for mortality, mainly when combined with ND‐HGS.

**2-54**

**Low skeletal muscle mass is associated with increased hospital costs in patients with cirrhosis listed for liver transplantation**

Jeroen L.A. van Vugt^1^, Stefan Buettner^1^, Louise J.M. Alferink^2^, Niek Bossche^3^, Ron W.F. de Bruin^1^, Sarwa Darwish Murad^2^, Wojciech G. Polak^1^, Herold J. Metselaar^2^ and Jan N.M. IJzermans^1^

^1^ *Department of Surgery, Division of HPB and Transplant Surgery, Erasmus MC University Medical Center, Rotterdam, the Netherlands;* ^2^ *Department of Gastroenterology and Hepatology, Erasmus MC University Medical Center, Rotterdam, the Netherlands;* ^3^ *Department of Control and Compliance, Erasmus MC University Medical Center, Rotterdam, the Netherlands*

**Introduction:** Low skeletal muscle mass (sarcopenia) is associated with increased morbidity and mortality in liver transplant candidates. We investigated the association between sarcopenia and hospital costs in patients listed for liver transplantation.

**Methods:** Consecutive patients with cirrhosis listed for liver transplantation between 2007 and 2014 in a Eurotransplant center were identified. The skeletal muscle index ([SMI], cm^2^/m^2^) was measured on CT performed within 90 days from waiting list placement. The lowest sex‐specific quartile represented patients with sarcopenia.

**Results:** In total, 224 patients were included. Median time on the waiting list was 169 (IQR 46–306) days, and median MELD‐score was 16 (IQR 11–20). The median total hospital costs in patients with sarcopenia were €11,294 (IQR 3,570–46,469) compared with €6,878 (IQR 1,305–20,683) in patients without sarcopenia (*p* = 0.008). In multivariable regression analysis, an incremental increase in SMI was significantly associated with a decrease in total costs (€458 per incremental SMI, 95%CI 14–902, *p* = 0.043), independent of the total time on the waiting list.

**Conclusions:** In conclusion, sarcopenia is independently associated with increased health‐related costs for patients on the waiting list for liver transplantation. Optimizing skeletal muscle mass may therefore lead to a decrease in hospital expenditure, in addition to greater health benefit for the transplant candidate.

**2-55**

**Body composition in extreme small stature adults: the case of mucopolysaccharidosis**

Bhawna Sharma^1,2^, Boyd Strauss^2^, Christian Hendriksz^1,3^ and Gisela Wilcox^1,2^

^1^ *Mark Holland Metabolic Unit, Salford Royal NHS Foundation Trust, UK;* ^2^ *Faculty of Biology, Medicine and Health, University of Manchester, UK;* ^3^ *Steve Biko Academic Unit, University of Pretoria, South Africa*

**Background:** The mucopolysaccharidoses (MPS) are a group of rare inherited metabolic disorders due to the accumulation of glycosaminoglycans (GAGs) within lysosomes. Accumulation of GAGs in different tissues causes significant multi‐systemic complications, associated with, in many cases, extreme short stature. Hydrophilic GAGs may alter body composition, but measurement has not yet been explored in the literature.

**Aims:** We aimed to assess if patients with MPS have a different body composition to that of the general population and to investigate in which body composition compartment GAGs accumulate predominantly.

**Methods:** Eleven MPS patients attending the adult inherited metabolic medicine clinic at Salford Royal Foundation Trust were measured using the multifrequency segmental SECA BIA 515. These data were compared to data from healthy European individuals.

**Results:** In the 11 patients with MPS, weight was significantly correlated with fat mass, but not percentage fat. Weight was also highly correlated with fat‐free mass and skeletal muscle mass, even after correction for height. Height was significantly correlated with fat‐free mass and skeletal muscle mass.

Patients with MPS have a significantly reduced stature, mass and phase angle, and significantly increased resistance to current flow, when compared to the general population.

**Conclusions:** The question of whether or not extreme small stature affects body composition is not yet resolved. The correlation between both weight and height, and skeletal muscle mass, could be accounted for by GAG accumulation in skeletal muscle; other body composition techniques, especially for muscle mass, need to be applied to this group and methods for estimating tissue GAG mass developed. This group challenges conventional definitions of sarcopenia.

**2-56**

**Phase angle and sarcopenia: are they related?**

Jéssica Härter^1^, Silvana Paiva Orlandi^1^ and Maria Cristina Gonzalez^1,2^

^1^ *Post‐graduate Program, Nutrition and Food, Federal University of Pelotas, Pelotas, RS, Brazil;* ^2^ *Post‐graduate Program, Health and Behavioral, Catholic University of Pelotas, Pelotas, RS, Brazil*

**Introduction:** Phase angle (PA) has been considered as a prognostic factor for several clinical conditions, probably because of its relationship to fat‐free mass and function. More recently, decreased muscle mass and functional assessment were used to define sarcopenia. The purpose of this work was to identify the relationship of PA, sarcopenia and its components in cancer patients.

**Methods:** Fifty‐nine cancer patients hospitalized for elective surgery were studied. Bioelectrical impedance analysis (BIA Quantum II, RJL Systems®) was performed in all patients and skeletal muscle index, using Janssen equation, and PA and Standardized Phase Angle (SPA) were estimated, using reference values from this population. The handgrip strength was obtained using Jamar® hydraulic dynamometer, and gait speed was assessed by using 4‐m test. The diagnosis of sarcopenia was performed according the *European Working Group on Sarcopenia in Older People*. Malnutrition was assessed by Patient‐generated Subjective Global Assessment. The outcomes evaluated were postoperative complications (Clavien–Dindo classification) and length of stay (LOS).

**Results:** Sarcopenia was present in 17% of the patients and malnutrition in 30.5%. SPA was significantly lower in malnourished (−0.20 vs. 0.62 *p = 0.028*) and sarcopenic patients (−0.39 vs. 0.59 *p = 0.010*). Patients with a lower handgrip strength (−0.31 vs. 0.69 *p = <0.001*) and slower gait speed (−0.30 vs. 0.60 *p = 0.016*) also showed a lower SPA. SPA and PA from non‐sarcopenic patients with impaired functions also showed a lower SPA and PA than those with normal function. Patients with serious postoperative complications (−0.71 vs. 0.41 *p = 0.007*) and longer LOS (−0.16 vs. 0.64 *p = 0.030*) presented lower SPA. The same results were found when PA (adjusted for sex and age) was used.

**Conclusions:** PA was decreased in all the sarcopenia components, and it has been associated with impaired nutritional and functional status in this sample.

**2-57**

**French translation and validation of the sarcopenia screening tool SARC‐F**

Charlotte Beaudart^1^, Médéa Locquet^1^, Stephen Bornheim^2^, Stéphane Schneider^3^, Jean‐Yves Reginster^1^ and Olivier Bruyère^1^

^1^ *Research Unit in Public Health, Epidemiology and Health Economics (URSAPES), University of Liège, Liège, Belgium;* ^2^ *Unité de Kinésithérapie générale, CHU Liège, Liège, Belgium;* ^3^ *Centre Hospitalier Universitaire de Nice, Pôle Digestif, Université de Nice Sophia‐Antipolis, Faculté de Médecine, Nice, France*

**Introduction:** The purpose of the present study was to translate into French the SARC‐F questionnaire, a simple and easy screening tool for sarcopenia, and then to validate this translated/French version on behalf of the EUGMS Special Interest Group (SIG) on Sarcopenia.

**Methods:** The translation process has been divided into two consecutive parts: 1) the translation of the questionnaire from English to French and its language validation (inter‐rater reliability and test–retest reliability); 2) the clinical validation of the French SARC‐F in order to assess its performance (sensitivity, specificity, predictive positive value and predictive negative value) in a cohort of Belgian elderly subjects, against seven existing definitions of sarcopenia.

**Results:** The translation from English to French has been performed without any difficulties and demonstrated an excellent inter‐rater reliability with an ICC of 0.90 (95% CI: 0.76–0.96) as well as an excellent test–retest reliability with an ICC of 0.86 (95% CI: 0.66–0.94). Afterwards, 306 subjects took part in the clinical validation of the French version of the SARC‐F questionnaire. Results showed that the sensitivity of the tool ranged from 22.1% to 75.0%, depending on the definition used for the diagnosis of sarcopenia, and the specificity ranged from 84.9% to 87.1%. Moreover, all PPVs were below 50%; the lowest PNV was 68.1% and the best one reached around 99%.

**Conclusions:** The results are in line with the performance established in the initial English validation of the SARC‐F and seem to indicate that this screening tool can detect with precision the absence of sarcopenia but seems less precise in affirming the presence of this geriatric syndrome.

**2-58**

**The SarQoL®, a specific quality of life questionnaire for sarcopenia, is adapted to identify 1‐year decrease in quality of life related to muscle function**

Charlotte Beaudart, Médéa Locquet, Laura Delandsheere, Jean‐Yves Reginster and Olivier Bruyère

*Research Unit in Public Health, Epidemiology and Health Economics (URSAPES), University of Liège, Liège, Belgium*

**Background:** Our aim was to assess the impact of 1‐year change in musculoskeletal health on quality of life (QoL) using the SarQoL® questionnaire, a quality of life questionnaire specific for sarcopenia.

**Methods:** Three QoL questionnaires (the SarQoL® and two generic QoL questionnaires, the EQ‐5D and the SF‐36) have been completed by 301 subjects from the SarcoPhAge study (Sarcopenia and Physical impairments with advancing Age, a cohort developed in Belgium). Muscle mass (ALM/h^2^, assessed with DXA), grip strength (assessed with hydraulic dynamometer) and gait speed were evaluated. Sarcopenia was diagnosed according to the EWGSOP algorithm.

**Results:** After one year of follow‐up, the QoL of the general population (75.0 ± 5.97 years, 59% women) decreased (*p* < 0.001 with the SarQoL®, p = 0.03 with the EQVAS, p < 0.001 with the EQ‐5D). The ALM/h^2^ was not significantly modified but a decrease in muscle strength, and gait speed was observed (*p* < 0.001 for both). A significant correlation was found between 1‐year decrease in gait speed and 1‐year decrease in QoL only when using the specific questionnaire SarQoL®, but not when using the generic EQ‐5D or EQVAS tools. Results indicated a correlation of *r* = 0.21 (p < 0.001) for the whole cohort population and *r* = 0.41 (*p* = 0.013) for the sarcopenic elders (*n* = 38). These associations were not observed for muscle mass (*p* = 0.65) or muscle strength (*p* = 0.06). Using a multivariate regression the association between decreased gait speed and decreased QoL, assessed with the SarQoL®, was significant, independently of age, sex, number of comorbidities and number of drugs (p < 0.001 for both whole cohort and sarcopenic subjects).

**Conclusions:** Our findings suggest that a decrease in physical performance and more specifically in gait speed is associated with a decrease in QoL in elders and more specifically in those suffering from sarcopenia. The specific SarQoL® seems better adapted than generic tools to identify decrease in QoL related to muscle function.

**2-59**

**Results from the use of the SARC‐F questionnaire associated with calf circumference as a sarcopenia screening tool in a Brazilian population**

Thiago G. Barbosa‐Silva^1^, Letítia Mazocco^2^, Patrícia Chagas^3^ and Maria Cristina Gonzalez^4^

^1^ *Programa de Pós‐Graduação em Epidemiologia da Universidade Federal de Pelotas (UFPel), Pelotas, RS, Brazil;* ^2^ *Programa de Pós‐graduação em Gerontologia da Universidade Federal de Santa Maria (UFSM), Santa Maria, RS, Brazil;* ^3^ *Departamento de Alimentos e Nutrição e Programa de Pós‐graduação em Gerontologia da Universidade Federal de Santa Maria (UFSM), Santa Maria, RS, Brazil;* ^4^ *Programa de Pós‐Graduação em Saúde e Comportamento da Universidade Católica de Pelotas (UCPEL), Pelotas, RS, Brazil*

**Introduction:** SARC‐F + CC was recently proposed as a variation of the SARC‐F questionnaire, combining its questions (which evaluate muscle function) with an anthropometric measurement (calf circumference (CC), as a surrogate for muscle mass]. However, SARC‐F + CC still lacks validation.

**Objective:** Evaluating SARC‐F + CC's performance as a sarcopenia screening tool.

**Methods:** Cross‐sectional study including elderly women (≥60 years old) who performed bone densitometry for clinical purposes. Sarcopenia was defined by the EWGSOP's recommended criteria: muscle mass evaluation through dual‐energy X‐Ray absorptiometry (DXA), muscle strength evaluation by handgrip strength and muscle performance evaluation through the 4‐m gait speed test. Specific cut‐off reference values for the Brazilian population were used to define low Appendicular Skeletal Muscle Mass Index (ASMI) from DXA (5.62 kg/m^2^) and CC (≤33cm). The Brazilian version of SARC‐F was applied, and 10 points were added to the SARC‐F score if the subjects had a low CC. Subjects with a final score ≥11 were considered as at sarcopenia risk.

**Results:** A total of 205 elderly women (mean age: 67.3 ± 5.9 years) were included in the study. The majority of the sample lived in rural areas (65.9%) were Caucasian (71.2%), had 4–8 years of schooling (47.3%), lived with a partner (61.5%) and were retired (92.2%). Applying EWGSOP criteria, the prevalence of sarcopenia was 2.4% of the total sample (5 subjects). Through SARC‐F + CC, 37 women (18%) were identified as in risk for sarcopenia. Although SARC‐F + CC presented low sensitivity (40%) and positive predictive value (5.4%), it performed well in identifying healthy participants (specificity: 82.5%) and had an excellent negative predictive value (98.2%).

**Conclusions:** This study confirms previous published findings which suggest that the combination of SARC‐F + CC, using specific regional cut‐off values for CC, can be used to rule out healthy subjects from further testing, improving sarcopenia screening in clinical practice.

**2-60**

**A cross‐sectional study on sarcopenia: association between muscle mass and strength and metabolic syndrome in Saudi men**

Shaea Alkahtani^1^, Sobhy Yakout^2^ and Nasser Al‐Daghri^2^

^1^ *Department of Exercise Physiology, College of Sports Science and Physical Activity, King Saud University, Riyadh, Saudi Arabia;* ^2^ *Prince Mutaib bin Abdullah Chair for Biomarkers Research on Osteoporosis, College of Science, King Saud University, Riyadh, Saudi Arabia*

**Introduction:** The mean value of appendicular lean mass (ALM) in Saudi young men has been recently determined and was different from those of other ethnicities. Whether ALM is associated with increased metabolic syndrome among Saudis has not been examined.

**Aim:** The aim was to determine the association between ALM, muscle strength and metabolic syndrome in Saudi men.

**Methods:** Participants included 497 Saudi men aged between 20 and 77 years. Hand grip and thigh strength was measured, and lean muscle mass was assessed using dual‐energy X‐ray absorptiometry (DXA). Venous blood samples were collected to assess metabolic syndrome markers.

**Results:** There were 14.9% of participants who had low ALM/Ht^2^ (2SD below reference value of Saudi young men). Handgrip and thigh strength were significantly lower among sarcopenic group (37.9 ± 7.1 vs 43.4 ± 7.5, p < 0.001 and 63.5 ± 24.3 vs 76.6 ± 23.5, p < 0.001, respectively). Metabolic Syndrome was 17.7% of participants, and 8.1% of them were classified as sarcopenic. There were no significant differences between sarcopenic and normal group in metabolic syndrome factors, but there was correlation between total cholesterol and ALM/Ht^2^ in sarcopenic group (*r* = 0.24). 63.9% of participants were obese (BMI > 30 kg/m^2^). Sarcopenic group had significantly lower level of fat compared with normal group, and the decrease was found in trunk and arms, but not legs (*p* = 0.27).

**Conclusions:** There was no association between low lean mass and metabolic syndrome in Saudi men. Socioeconomic factors that interact with current data have to be examined.

**2-61**

**Prevalence of sarcopenia in patients with cachexia and disease‐related malnutrition**

Igor Khoroshilov^1^ and Sergei Ivanov^2^

^1^ *North‐Western State Medical University named after I.I. Mechnikov, St. Petersburg, Russian Federation;* ^2^ *Pavlov First Saint Petersburg State Medical University, St. Petersburg, Russian Federation*

**Aim:** The sarcopenia prevalence assessment in patients with cachexia and disease‐related malnutrition.

**Methods:** 44 patients (16 men, 28 women) with undernutrition (BMI less than 18.5 kg/m2) held the treating in 2012–2016 years were included into retrospective analyze. Sarcopenia was identified by criteria suggested G. Biolo, T. Cederholm and M. Muscaritoli (2014): fat‐free mass index (FFMI) less than 17 kg/m^2^ for men and less than 15 kg/m^2^ for women. Bioimpedance analysis and dual energy X‐ray absorptiometry were used for FFMI evaluating.

**Results:** It has been found that FFMI in the patients with cachexia and precachexia with inflammation (cancer, Crohn's disease, ulcerative colitis, sepsis etc.) were 13.96 ± 1.4 kg/m^2^, particularly 13.5 ± 0.47 kg/m^2^ in 7 women and 14.3 ± 0.49 kg/m^2^ in 9 men. Sarcopenia was found at all of 16 patients with cachexia and precachexia (100%). At 28 patients with chronic and acute disease‐related malnutrition (without inflammation) FFMI were 13.7 ± 0.4 kg/m^2^, particularly 13.7 ± 0.46 kg/m^2^ in 21 women and 13.7 ± 1.2 kg/m^2^ in 7 men. In patients without inflammation sarcopenia had been observed in 19 persons (68%). Significant differences in FFMI were found between patients with sarcopenia but without inflammation (FFMI was 12.68 ± 0.49 kg/m^2^) and patients with sarcopenia combined with cachexia (*P* < 0.05).

**Conclusions:** Sarcopenia is observed at all of patients with cachexia and precachexia. Among patients with undernutrition without inflammation the sarcopenia was observed at 68% of them.

**2-62**

**Muscle thickness measured by A‐mode ultrasound in prediction of total lean body mass in apparently healthy adults**

Renata M. Bielemann, Mariana O. Xavier, Arele R. Nunes, Rafaela B. Bergmann, Silvana P. Orlandi, Thiago G. Barbosa‐Silva, Maria Cecília Assunção and Maria Cristina Gonzalez

*Federal University of Pelotas, Brazil, Catholic University of Pelotas, Brazil*

**Introduction:** In the last decades, ultrasound (US) has emerged as a promising tool to assess body composition, especially in situations where the assessment of muscle mass should be done at bedside. To evaluate the relationship between muscle thicknesses in various body regions and total lean soft tissue in adults.

**Methods:** Cross‐sectional study with 208 individuals between 20 and 59 years from Pelotas, Brazil. Muscle thicknesses of biceps, triceps, thigh and calf regions were measured by A‐mode ultrasound (BodyMetrix®). Arm, thigh and leg circumference and length were also measured. Lean soft tissue index (LSTI) (kg/m^2^) was obtained by total lean body mass from DXA divided by squared height. The highest muscle thickness from eight ultrasound measurements in each region was used in the analyses. Pearson correlation coefficients were obtained between muscle thickness of regions of the upper and lower limbs and LSTI. Adjusted coefficients of determination (R^2^) were described to show the explained variance in LSTI promoted by addition of each anthropometric information from linear regression.

**Results:** Muscle thickness from thigh showed the highest correlation coefficient with LSTI in both men (*r* = 0.68) and women (*r* = 0.52). Correlation coefficients of sum of muscle thickness from the four regions and LSTI was 0.78 in men and 0.58 in women. Around 62% and 36% of variance in LSTI was explained by the sum of muscle thickness in the four regions. Arm, thigh and leg length did not increase the *R* ^2^, but arm, thigh and leg circumference increased the R^2^ to around 69% and 44% in men and women, respectively. Body weight did not add explanation in LSTI after use of all other anthropometric measurements.

**Conclusions:** The results demonstrated that the A‐mode ultrasonography has shown good predictive value of LSTI in apparently healthy men, mainly in set with other anthropometric information.

**2-63**

**Can phase angle detect differences among nor‐sarcopenic patients with impaired function?**

Maria Cristina Gonzalez^1^, Jaqueline Flores de Oliveira^1^, Inara Regina Frühauf^2^, Eduarda Jaine Facchinello Dall'Aqua^2^, Jean Pierre Oses^1^, Maristela Bohlke^1^ and Rafael Orcy and Thiago Gonzalez Barbosa‐Silva^3^

^1^ *Programa de Pós‐Graduação em Saúde e Comportamento da Universidade Católica de Pelotas (UCPEL), Pelotas, RS, Brazil;* ^2^ *Graduação em Medicina da Universidade Católica de Pelotas (UCPEL), Pelotas, RS, Brazil;* ^3^ *Programa de Pós‐Graduação em Epidemiologia da Universidade Federal de Pelotas (UFPEL), Pelotas, RS, Brazil*

**Introduction:** The European Working Group on Sarcopenia in Older People (EWGSOP) considered muscle mass loss to identify sarcopenic (S) or pre‐sarcopenic (PS) patients. However, loss of function without muscle mass loss, here called “functionally impaired with normal muscle mass” (FINMM), may determine a different prognostic risk. The objective of this study is to use Phase angle (PA), a prognostic marker in several clinical situations, and compare its values between FINMM and patients with normal muscle mass and function in non‐sarcopenic (NS) patients.

**Methods:** Sarcopenia was assessed according to EWGSOP criteria. The muscle mass was evaluated through the calf circumference (CC), using validated cut‐offs for this population. Muscle strength and performance were assessed by handgrip strength and 4‐m gait speed test. PA was assessed with a Bioelectrical impedance analysis device, using resistance and reactance from 50 kHz.

**Results:** A sample of 82 patients with a mean age of 53.9 ± 16.9 years were studied. PS was present in 4.9% of the sample, and sarcopenia in 32.9% (27 patients). There were no significant differences in PA values among NS (5.91° ± 1.31°), PS (6.65° ± 1.54°) and S patients (5.38° ± 1.27 ). Only severe sarcopenic patients had PA values significantly lower than NS and PS patients. From the 51 patients identified as NS, 34 patients were considered FINMM. When normal and FINMM patients were compared, it was found that PA values from FINMM patients were significantly lower than normal patients (5.46° ± 1.11°× 6.82° ± 1.22°, *p* = 0.003), and they were not significantly different from PA values found in PS or S patients.

**Conclusions:** The lower PA values found in FINMM patients may suggest that they should be identified as a special risk group among non‐sarcopenic patients. Longitudinal studies may show the increased morbidity and mortality in this group.

**2-64**

**SARA‐data platform: clinical trials novel methodologies and big data to evaluate SARconeos in Age‐related sarcopenia**

Susanna Del Signore^1,2^, Gianluca Zia^1^, Stefania Del Signore^1^ and Waly Dioh^2^

^1^ *Bluecompanion ltd, London, UK;* ^2^ *Biophytis, Paris, France*

**Introduction:** SARA clinical development program encompasses one observational study and two randomized, placebo controlled clinical trials for evaluating the candidate drug SARconeos (BIO101) in sarcopenic and obese sarcopenic patients Aged ≥65 years.

**Methods:** We deployed an integrated Information & Communication Technology (ICT) platform, SARA‐data, to monitor on real‐time different source clinical, imaging (DEXA), laboratory and physical activity data generated at investigation sites, centralised lab and by the patients via wearable devices and auto‐evaluation questionnaires.

Standard designed clinical trials may be inadequate for collecting informative long‐term clinical data in older adults. Rigid scheduling of visits and investigations negatively affects compliance and retention rate while increasing the risk of biased results. Little is known about intercurrent changes in physical function between protocol scheduled visits at the clinic.

A novel approach, including systematic and continuous use of wearable devices, allows to collect and integrate in one single web‐based portal all different data sources: electronic Case Report Form clinical data, imaging DEXA scans, biochemistry‐haematology results from a centralised laboratory and disease related biomarkers. Of note, continuous physical activity recording is enabled during the whole clinical trial duration by providing each older participant with a wrist‐worn accelerometer. The device transmits anonymised activity data to SARA platform via a non‐intrusive, unattended, home‐centred machine‐to‐machine technology. This kind of high volume data, directly generated by the patient during several months, fulfils the definition of Health‐related Big Data, and will constitute an important resource for additional, supportive analyses complementing standardised muscular function assessments. These data are generated in a real‐life context and could provide answers to specific questions by regulators and payers.

**Conclusions:** SARA‐data is an adapted ICT, real‐time data platform for conducting long‐term clinical trials in older patients. The potential impact of using Big Data in geriatric clinical research represents a further advantage, and enables secondary research.

**2-65**

**CT‐defined muscularity: population distribution and prognostic significance for overall survival**

Dalton Luiz Schiessel^1^, Lisa Martin^2^, Michael B. Sawyer^2^, Oliver Bathe^3^, David Bigam^2^, Aldo Montano‐Loza^2^, Vincent Thai^2^, Pierre Senesse^4^ and Vickie E. Baracos^2^

^1^ *Nutrition Department‐Midwestern State University‐UNICENTRO, Guarapuava, Brazil;* ^2^ *University of Alberta, Edmonton, AB, Canada;* ^3^ *University of Calgary, Calgary, AB, Canada;* ^4^ *Institut du Cancer de Montpellier, Montpellier, France*

**Background:** Computed tomography (CT) is an assessment of body composition offering high precision and specificity. In cancer patients, CT measurements of muscularity are correlated with dose limiting toxicity, hospital length of stay, surgery infections/complications and overall survival (OS). Skeletal muscle compartment is one determinant of these adverse outcomes. Small sample sets to date have hampered our ability to understand the shape of the relationship between muscularity and outcomes. We sought to define the sex‐specific relationships between muscularity and mortality in a large international data set including patients from Canada and Europe.

**Methods:** Skeletal area (cm^2^) (SMA) and skeletal muscle index (SMI, cm^2^/m^2^) were assessed by CT at the 3rd lumbar vertebra (L3) in advanced solid tumor patients. Data were stratified into 10 equal groups (deciles), to explore relationships to OS.

**Results**: Male patients (*n* = 2751) were 64 (±11.5) years, tumor sites were colorectal (29.8%), other GI (22.3%) and lung (17.4%) cancers that were mostly stage 4 (60.9%). Median of OS was 20.7 (95% CI 19.1–22.3) months and mean SMI 49.5 ± 9.2 cm^2^/m^2^ and SMA 150.7 ± 29.27 cm^2^. Survival by deciles of SMI (univariate) are shown (Table 1). SMI was continuously related to OS, with the longest OS in the most muscular individuals. SMI was independently prognostic of OS in a model adjusted for age, cancer site, stage & performance status (*p* = 0.021). Stratification on SMA gave similar results. Women (*n* = 1981) have a smaller range of muscularity than men. Stratification of SMI and SMA by deciles also showed a continuous relationship with OS. In men and women of equal muscularity, survival in women was significantly longer.

**Table 1:** Patient characteristics in male sex: skeletal muscle index and median overall survival

	SMI (cm^2^/m^2^)*	Overall Survival (months)**				
	mean ± sd	Median	95% CI	HR	95% CI	*P*
Male(n = 275)						
Deciles0‐10	34.2 ± 3.7	5.9	4.3–7.5	3.22	2.6–4.0	0.000
Deciles10‐20	40.1 ± 1.1	12.8	10.6–15.0	2.17	1.7–2.7	0.000
Deciles20‐30	43.2 ± 0.7	15.2	12.3–18.1	1.93	1.5–2.4	0.000
Deciles30‐40	45.6 ± 0.7	17.7	13.9–21.5	1.78	1.4–2.2	0.000
Deciles40‐50	47.9 ± 0.7	22.4	16.9–27.9	1.44	1.1–1.8	0.000
Deciles50‐60	50.3 ± 0.6	24.4	18.8–30.0	1.36	1.0–1.7	0.010
Deciles60‐70	52.7 ± 0.9	26.1	22.4–29.8	1.42	1.1–1.8	0.000
Deciles70‐80	55.5 ± 0.8	32.3	27.2–37.5	1.10	0,9–1.4	0.420
Deciles80‐90	58.9 ± 1.2	36.7	29.6–43.7	1.05	0.8–1.3	0.710
Deciles90‐100	66.6 ± 4.9	36.8	28.5–45.0			

* Kruskal–Wallis test * p < 0.000 between all deciles groups.

** Kaplan–Meier method—Log‐rank (Mantel–Cox) analysis and Cox proportional hazards model.

**Conclusions:** Muscularity is continuously related to OS in male and female cancer patients. Prior research used statistical tests to identify threshold values of SMI associated with survival (i.e. “sarcopenia” thresholds); this bivariate approach oversimplifies the relationship with OS.

**3-01**

**Skeletal muscle capillary density predicts insulin sensitivity and muscle morphological adaptation in older adults**

Tatiana Moro^1,3^, Camille.R. Brightwell^1^, Rachel R. Deer^3^, Elena Volpi^2,3^, Blake B. Rasmussen^1,3^ and Christopher S. Fry^1,3^

^1^ *Department of Nutrition & Metabolism, School of Health Professions, University of Texas Medical Branch, Galveston, TX, USA;* ^2^ *Department of Internal Medicine/Geriatrics, University of Texas Medical Branch, Galveston, TX, USA;* ^3^ *Sealy Center on Aging, University of Texas Medical Branch, Galveston, TX, USA*

**Introduction:** Aging induces a substantial decrease in muscle capillarization, reducing the transport of nutrients, oxygen and hormones, such as insulin, into muscle fibers. Thus, muscle capillarization could be critical not only for muscle growth but also for glucose intolerance and insulin resistance. It is known that physical activity can improve muscle perfusion by increasing capillary density in young adults; however, its role in older adults is still controversial.

The aim of this study was to investigate the association between muscle capillary density and indices of muscle hypertrophy and insulin sensitivity before and after 12 weeks of progressive resistance exercise training (RET).

**Methods:** 19 subjects (71.1 ± 4.3 years; 27.6 ± 3.2 BMI) were studied before and after 12 weeks of RET. Pre‐ and post‐training measurements of muscle mass, strength and OGTT were obtained. Insulin sensitivity was assessed with Matsuda index and OGIS index. In addition, pre and post study days also included percutaneous biopsies from the vastus lateralis muscle. Immunohistochemical analysis was used to quantify capillary density, myosin heavy chain (MyHC) isoform expression and cross‐sectional area (CSA).

**Results:** Basal muscle capillarization was highly correlated with lean body mass, sarcopenic index (SMI) and daily physical activity level (*p* < 0.05). Muscle capillary density was also positively associated (p < 0.05) with fiber type I frequency and fiber size (CSA). Moreover, higher basal capillarization was positively correlated (p < 0.05) with a higher improvement in the insulin sensitivity index and muscle fiber CSA. RET increased muscle capillary density (p < 0.05), which was also correlated with an increase in fiber CSA and MyHC type II fiber frequency.

**Conclusions:** Muscle fiber capillarization at baseline may be a predictive factor for improving insulin sensitivity in response to RET in older adults. Increases in muscle fiber perfusion following RET are correlated with muscle growth.

**3-02**

**3 days of human skeletal muscle disuse promote fatty infiltrations development**

Guillaume Py^1^, Allan F. Pagano^1^, Thomas Brioche^1^, Coralie Arc‐Chagnaud^1,2^, Rémi Demangel^1^ and Angèle Chopard^1^

^1^ *Université de Montpellier, INRA, UMR866 Dynamique Musculaire et Métabolisme, Montpellier, France;* ^2^ *Freshage Research Group ‐ Dept. Physiology ‐ University of Valencia, CIBERFES, INCLIVA, Valencia, Spain*

**Introduction:** Fatty infiltrations, or intermuscular adipose tissue (IMAT), are currently well‐recognized components of muscle deconditioning. Although IMAT is present in healthy human skeletal muscle, its increase and accumulation are linked to muscle dysfunction. Although IMAT development has been largely attributable to inactivity, the precise mechanisms of its establishment are still poorly understood. Because the sedentary lifestyle that accompanies age‐related sarcopenia may favor IMAT development, deciphering the early processes of muscle disuse is of great importance before implementing strategies to limit IMAT deposition.

**Methods:** Here, we took advantage of the dry immersion (DI) model of severe muscle inactivity to induce rapid muscle deconditioning during a short period. Skeletal muscle biopsies were obtained from the vastus lateralis of healthy men (*n* = 12; age: 32 ± 6) before and after 3 days of DI.

**Results:** We showed that DI for only 3 days was able to decrease myofiber cross‐sectional areas (−10.6%). Protein expression levels of two key markers commonly used to assess IMAT, perilipin and FAPB4 were upregulated. We observed an increase in the C/EBPα and PPARγ protein expression levels, indicating an increase in late adipogenic processes leading to IMAT development. While many stem cells in the muscle environment can adopt the capacity to differentiate into adipocytes, fibro‐adipogenic progenitors (FAPs) appear to play a major role in IMAT development. In our study, we showed an increase in the protein expression of PDGFRα, the specific cell surface marker of FAPs, in response to 3 days of DI. It is well recognized that an unfavorable muscle environment drives FAPs to ectopic adiposity and/or fibrosis.

**Conclusions:** This study is the first to emphasize that during a short period of severe inactivity, muscle deconditioning is associated with IMAT development. Our study also reveals that FAPs could be the main resident muscle stem cell population implicated in ectopic adiposity development in human skeletal muscle.

**3-03**

**Mitochondrial alterations in pre‐frail/frail elderly women are in part secondary to signals originating in persistently denervated muscle fibers**

Vita Sonjak^1,2^, Madhusudanaro Vuda^2^, Kayla Miguez^1,2^, Carole Spake^1^, Kathryn J. Wright^3^, Anna Perez^2^, Jose A. Morais^3^, Tanja Taivassalo^4^ and RussellT Hepple^5^

^1^ *Department of Kinesiology and Physical Education, McGill University, Montreal, QC, Canada;* ^2^ *Meakins Christie Laboratories and Research Institute of the McGill University Health Centre, Montreal, QC, Canada;* ^3^ *Research Institute of the McGill University Health Centre, Montreal, QC, Canada;* ^4^ *Department of Physiology and Functional Genomics, University of Florida, Gainesville, FL, USA;* ^5^ *Department of Physical Therapy, University of Florida, Gainesville, FL, USA*

**Introduction:** Mitochondrial impairment is implicated in the age‐related decline in skeletal muscle, but whether the mitochondrion represents a therapeutic target depends upon whether the changes in function of this organelle are primary organelle defects or secondary responses to other changes in the aging *milieu*. Whereas we recently showed that mitochondrial function changes in octogenarian men are at least partly secondary to muscle fiber denervation, this has not been addressed in pre‐frail/frail elderly women.

**Methods:** Mitochondrial respiratory capacity by high‐resolution respirometry and reactive oxygen species (ROS) emission by Amplex Red was determined in saponin permeablized myofibers obtained from *Vastus lateralis* muscle biopsies of prefrail/frail elderly (FE; 79.6 ± 4.9y) and healthy young inactive (YI; 23.1 ± 2.5y) women. The effect of denervation on mitochondrial function during the ROS assay was assessed pharmacologically upon inhibition of cPLA_2_ with arachadonyl trifluoromethyl ketone (AACOCF_3_). The presence of denervated myofibers was assessed morphologically by histochemistry.

**Results:** Muscle respiratory capacity was reduced in FE due to lower mitochondrial content, whereas FE exhibited higher ROS emission even after normalizing for mitochondrial content compared to YI group. The reduction in ROS production compared to ethanol vehicle control in FE (Ethanol: 1.3 ± 0.5 pmol/min/mg vs AACOCF_3_: 0.8 ± 0.4 pmol/min/mg; *p* < 0.001) reveals a modulating impact of denervation on mitochondrial function in FE women, as seen previously in octogenarian men. Consistent with this, there was a significant accumulation (15.9%) of very small fibers (defined as a size ≤1505 μm^2^) exhibiting histological evidence of denervation (high non‐specific esterase activity) in FE.

**Conclusions:** We can conclude that denervation plays a modulating role in skeletal muscle mitochondrial function in prefrail/frail elderly women, suggesting therapeutic strategies in advanced age should focus on the causes of persistent denervation.

**3-04**

**Relationships between the branched chain amino acid transporter LAT 3 and Myosin Heavy Chain proteins in human skeletal muscle**

Britt‐Marie Iresjö, Cecilia Engström and Kent Lundholm

*Institute of Clinical Sciences, Dep. of Surgery, Sahlgrenska Academy, University of Gothenburg, Sweden*

**Introduction:** Recent studies indicate participation of amino acid transporter proteins in activation of mTOR signaling and down‐stream increase in muscle protein synthesis. In a previous study we identified amino acid transporter LAT 3, a transporter of BCAA, met, phe, as significantly altered in response to provision of total parenteral nutrition (TPN) (p < 0.01). Here we investigate if alterations in LAT3 mRNA and protein concentrations reflect altered gene transcription of myosin proteins.

**Methods:** 22 patients (mean weight loss 6 ± 2%) scheduled for upper GI‐surgery received either a continuous standard TPN infusion (0.16 gN kg^−1^ day^−1^, 30 kcal kg^−1^ day^−1^) or saline infusion for 12 hours overnight before operation. Biopsies from the rectus abdominis muscle were taken at start of operation. LAT 3 mRNA and mRNA of Myosin heavy chain (MHC) isoforms MHC1, MHC2A and MHC2X were analyzed by real‐time PCR. LAT3 transporter protein content was quantified by western blot in a subgroup of patients (*n* = 18). Linear regression was used for analyses of relationships.

**Results:** MHC2A mRNA concentrations showed a positive regression with LAT 3 mRNA (*r* = 0.56; *p* = 0.007), while there were no relationship between MHC1 or MHC2X and LAT3 mRNA concentrations. There were a negative relationship between MHC2A mRNA and LAT 3 protein content (*r* = 0.62; p = 0.007) while MHC2X mRNA showed a positive linear relationship with LAT3 protein content (*r* = 0.53; *p* = 0.02). No relationship was found between MHC1 mRNA and LAT3 protein content.

**Conclusions:** Both mRNA and protein concentrations of the branched chain amino acid transporter LAT 3 showed a significant relationship with Myosin Heavy Chain 2A mRNA concentrations in muscle cells. The lack of relationship with other Myosin Heavy chain isoforms and LAT3 may suggest specific regulation of individual MHC transcripts. These findings deserve further nutritional evaluations to understand the control of myofibrillar protein translation.

**3-05**

**mtDNA content and mtDNA deletion mutation abundance in skeletal muscle of sedentary high‐ and low‐functioning elderly individuals**

Anna Picca^1^, Andres Gordillo Villegas^2^, Robert Mankowski^2^, Angela Maria Serena Lezza^3^, Riccardo Calvani^1^, Emanuele Marzetti^1^, Roberto Bernabei^1^ and Christiaan Leeuwenburgh^2^

^1^ *Department of Geriatrics, Neurosciences and Orthopedics, Catholic University of the Sacred Heart School of Medicine, Teaching Hospital “Agostino Gemelli”, Rome, Italy;* ^2^ *Department of Aging and Geriatric Research, Institute on Aging, Division of Biology of Aging, University of Florida, Gainesville, FL, USA;* ^3^ *Department of Biosciences, Biotechnology and Biopharmaceutics, University of Bari, Bari, Italy*

**Introduction:** Mitochondrial dysfunction in skeletal myocytes has been proposed as a major factor contributing to the development and progression of sarcopenia. Hence, the quantitation of mitochondrial DNA (mtDNA) abundance and mtDNA deletion mutation load may help clarify the role of mtDNA instability in muscle aging.

**Methods:** We applied real‐time PCR‐based approaches to total DNA purified in muscle samples obtained from young adults, sedentary older adults, classified as high‐ and low‐functioning based on the Short Physical Performance Battery (SPPB), in order to examine the effect of aging on key quantitative alterations of mtDNA and how this relates to physical performance.

**Results:** Muscle volume, as quantified via 3D‐NMR, was decreased by 38% and 30% in low‐ (LFE) and high‐functioning elderly (LFE) participants, respectively, when compared to young and high‐functioning elderly participants, respectively, and positively correlated with physical performance. The content of mtDNA was found to be significantly reduced in both groups of elderly participants, regardless of the SPPB score, relative to their younger counterparts. The age‐associated decrease in mtDNA abundance was paralleled by an increase in the mtDNA deletion in HFE and LFE participants, with no differences between the two groups. Further investigations will probe alterations in mtDNA encoding genes: NADH dehydrogenase 1 (ND1/Complex 1), Cytochrome b (Complex III), and cytochrome c oxidase (COI/Complex IV).

**Conclusions:** This study shows altered mitochondrial homeostasis in muscles of aged human. The decline in myocyte mitochondrial mass and the accumulation of mtDNA deletions may therefore represent critical steps to muscle aging and possible targets for interventions against sarcopenia.

**3-06**

**Genetic variants associated with physical performance and anthropometry in old age: a genome‐wide association study in the *ilSIRENTE* cohort**

Emanuele Marzetti^1^, David Heckerman^2^, Bryan J. Traynor^3^, Anna Picca^1^, Riccardo Calvani^1^, Dena Hernandez^4^, Michael Nalls^5^, Sampath Arepali^4^, Luigi Ferrucci^6^, Roberto Bernabei^1^ and Francesco Landi^1^

^1^ *Center for Geriatric Medicine (CEMI), Department of Geriatrics, Neurosciences and Orthopedics, Catholic University of Sacred Heart, Rome, Italy;* ^2^ *Microsoft Research, Los Angeles, CA, USA;* ^3^ *Neuromuscular Diseases Research Section, Laboratory of Neurogenetics, National Institute on Aging, Bethesda, MD, USA;* ^4^ *Genomics Technology Group, Laboratory of Neurogenetics, National Institute on Aging, Bethesda, MD, USA;* ^5^ *Molecular Genetics Unit, Laboratory of Neurogenetics, National Institute on Aging, Bethesda, MD, USA;* ^6^ *Longitudinal Studies Section, Clinical Research Branch, National Institute on Aging, Baltimore, MD, USA*

**Background:** Unraveling the complexity of aging is crucial for understanding its mechanisms and why aging is the risk factor for most chronic conditions. The advancements marked by genome‐wide association studies (GWASs) have sparked interest in gene cataloguing in the context of aging and age‐related conditions. Here, we used GWAS to explore whether single nucleotide polymorphisms (SNPs) were associated with functional and anthropometric parameters in a cohort of old community‐dwellers enrolled in the *ilSIRENTE* aging study.

**Methods:** Analyses were carried out in men and women aged 80+ years enrolled in the *ilSIRENTE* Study (*n* = 286) and replicated in the *inCHIANTI* Study (*n* = 1055). Genotyping was accomplished on Infinium Human610‐QUAD version 1.

**Results:** In the *ilSIRENTE* population, genetic variants in ZNF295 and C2CD2 (rs928874 and rs1788355) on chromosome 21q22.3 were significantly associated with the 4‐meter gait speed (rs928874, *p* = 5.61 × 10^−8^; rs1788355, *p* = 5.73 × 10^−8^). This association was not replicated in the *inCHIANTI* population.

**Conclusions:** Our findings suggest that specific SNPs may be associated with a key measure of physical performance in older adults. GWASs using larger samples are needed to confirm these preliminary results to enhance our comprehension of complex age‐associated phenomena.

**3-08**

**Skeletal muscle dysfunction in cancer‐associated cachexia characterized by changes in proliferation and differentiation markers in gastrointestinal cancer patients**

Gabriela Salim de Castro^1^, Joanna Darck Carola Correia Lima^1^, Estefanía Simões Fernández^1^, Raquel Galvão Figueredo^1^, Emídio Marques de Matos‐Neto^1^, Paulo Sérgio Alcantara Martins^2^, José Pinhata Otoch^2,3^, Athanassia Sotiropoulos^4^, Dario Coletti^5^ and Marília Cerqueira Leite Seelaender^1,3^

^1^ *Cancer Metabolism Research Group, University of São Paulo, Brazil;* ^2^ *Department of Clinical Surgery, University Hospital, University of São Paulo, Brazil;* ^3^ *Faculdade de Medicina, University of São Paulo, Brazil;* ^4^ *Inserm U1016, Institut Cochin, France;* ^5^ *Department of Biological Adaptation and Ageing B2A, Pierre et Marie Curie University (Paris 6), France*

**Introduction:** Cancer‐associated cachexia is a metabolic syndrome characterized by weight loss and systemic inflammation. Exacerbated inflammation seems to cause protein and energy disorders leading to reduced survival and quality of life. Furthermore, systemic inflammation may be involved in muscle wasting and impaired myogenesis. Therefore, the present study aimed to evaluate satellite cells markers and cell signaling proteins in skeletal muscle of gastrointestinal cancer patients.

**Methods:** Patients with gastrointestinal cancer were recruited after signature of the informed consent form. *Rectus abdominis* muscle biopsies were collected in surgery. Patients were separated into Weight‐Stable Cancer (WSC) and Cachectic Cancer (CC) groups (WSC *n* = 13 and CC *n* = 16) according to criteria described by Evans *et al*. (2008). Muscle mRNA was extracted and real‐time PCR gene expression was analysed using 2^‐ΔΔCT^. Cell signalling proteins and CX3CL1 were quantified with Luminex®xMAP technology.

**Results:** mRNA relative levels of two transcription factors expressed mainly by satellite cells, PAX7 and MYF5, were increased in CC patients (*p* = 0.004 and *p* = 0.003 respectively). Furthermore, CC muscle biopsies showed decreased JNK (*p* = 0.006) and p53 protein content (*p* = 0.03) and an increase in those of ATF‐2 (*p* < 0.03), cJUN (*p* = 0.04) and mitogen‐ and stress‐activated protein kinase (MSK1) (*p* = 0.038) in relation to WSC patients. CX3CL1, a chemokine that may be involved in muscle regeneration, was decreased in CC patients (*p* = 0.0004).

**Conclusions:** Changes in satellite cells markers seems to indicate an attempt towards myoblasts proliferation and differentiation; however, this process may be compromised as suggested by the lower p53 protein content. Sustained systemic inflammation may impair myocyte differentiation and consequently decrease muscle regeneration capability in CC patients. Further analysis of muscle differentiation markers are required to corroborate these findings.

**3-09**

**Evaluation of serum markers for assessing muscle mass in cancer cachexia**

Christina Van Der Borch^1^, Sara Wing^1^, Rachel Murphy^2^, Ami Grunbaum^3^, Sean Eintracht^3^, Elizabeth MacNamara^3^, Vickie Baracos^2^, Vera Mazurak^2^ and R. Thomas Jagoe^1^

^1^ *McGill Cancer Nutrition Rehabilitation Program Clinic, Jewish General Hospital, Montreal, Canada;* ^2^ *Alberta Institute for Human Nutrition, University of Alberta, Edmonton, AB, Canada;* ^3^ *Department of Clinical Biochemistry, Jewish General Hospital, Montreal, Canada*

**Introduction**: Low muscle mass is a defining feature of cancer cachexia. Methods for measuring muscle mass exist, but these are often too time‐consuming and expensive for the routine evaluation of patients in clinic. Several different circulating markers of muscle mass have been proposed but to date, none have been validated.

**Methods**: Repeated measurements of body composition (CT cross‐sectional imaging or DEXA) were performed and simultaneous blood samples were collected, on up to 4 occasions, from patients with cancer. Serum assays for nine different candidate markers of muscle mass were measured. The correlation between each serum marker and muscularity (appendicular skeletal muscle mass index (ASMI (kg/h^2^)) was assessed. In addition, comparison of values of each serum marker in those with and without low muscle mass was performed.

**Results**: 43 patients (67% men), mean (SD) age 62(12) yr were recruited at two sites. 60% had lung cancer and 91% had advanced (stage III/IV) disease. Serum levels of Creatinine Kinase, Myoglobin, Ghrelin, Testosterone (men), Insulin, Growth Hormone, Cortisol and C‐reactive protein did not correlate with ASMI. However, insulin‐like growth factor‐1 (IGF) levels did correlate with ASMI (e.g. visit 1: *R* = 0.38, *P* = 0.02). Furthermore, using sex‐specific cut‐offs for ASMI, IGF levels was the only marker which was significantly different in sarcopenic individuals (Sarcopenia N vs Y: 24.9 vs 17.3 nmol/l, *P* = 0.04). Lower IGF levels were also associated with greater loss of muscle mass (e.g. IGF < 17 nmol/l: 0.8 kg loss vs 0.2 kg gain, P = 0.02).

**Conclusions**: Of the serum markers tested, only IGF appears to be useful to identify advanced cancer patients who have low muscle mass. Furthermore, low circulating IGF may also help predict which patients are most at risk of further muscle loss.

**3-10**

**TNF‐α‐mediated inflammatory apoptosis and angiogenesis in physical inactivity‐induced muscle atrophy**

Xiangke Chen^1^, Chen Zheng^2^ and Joseph Shiu Kwong Kwan^1^

^1^ *Department of Medicine, The University of Hong Kong, Hong Kong;* ^2^ *Department of Sports Science and Physical Education, The Chinese University of Hong Kong, Hong Kong*

**Introduction:** Skeletal muscle atrophy can result from physical inactivity, space flight and chronic bed rest associated with ageing. Hind‐limb unloading (HU) is an accepted rodent model of muscle disuse to simulate physical inactivity. We investigated the roles of tumor necrosis factor‐alpha (TNF‐α) in apoptosis and angiogenesis pathways using HU‐induced muscle atrophy model.

**Methods:** HU was performed in 8‐week‐old ICR mice for 14 consecutive days. We compared muscle mass, muscle strength, morphology, and blood flow, reactive oxygen species (ROS), TNF‐α, vascular endothelial growth factor (VEGF), endothelial NOS (eNOS), cytochrome C (Cyto‐C), B‐cell lymphoma 2 (Bcl‐2), Bcl‐2‐associated X protein (Bax), caspase‐9, and caspase‐3 between HU vs. control mice.

**Results:** As compared to control mice, HU mice were significantly lower in muscle mass and strength (*p* < 0.01), with their muscle fibers arranged more loosely along a different direction. HU mice had significantly higher levels of blood ROS, expressions of TNF‐α, VEGF, and caspase‐3 in the skeletal muscle (*p* < 0.01), and the blood flows in the hind‐limb and whole body were significantly higher (*P* < 0.05). No significant differences were found in the expressions of other biomarkers between the two groups. TNF‐α was highly correlated with both caspase 3 (*r* = 0.725, *P* < 0.01) and VEGF (r = 0.725, *P* < 0.01) in the hind‐limb muscles in response to HU.

**Conclusions:** TNF‐α is involved in both the caspase 3‐mediated apoptosis pathway and the VEGF‐mediated angiogenesis pathway in HU‐induced muscle atrophy. Development of future treatments for physical inactivity‐induced muscle atrophy should take into consideration the findings of this study.

**3-11**

**5‐Fluoroucil's effect on indices of skeletal muscle wasting in Apc^Min/+^ mice**

Brittany R. Counts^1" noteRef="jcsm12255-subcmp-0072-note-0001^, Kristen M. Larsen^2" noteRef="jcsm12255-subcmp-0072-note-0001^, Brandon N. VanderVeen^1^, Justin P. Hardee^1^, Dennis A. Fix^1^, Maydelis K. Minaya^2^, Celestia Davis^2,3^, Maria M. Peña^2,3^ and James A. Carson^1,3^

^1^ *Arnold School of Public Health, University of South Carolina, Columbia, USA;* ^2^ *Department of Biological Sciences, University of South Carolina, Columbia, USA;* ^3^ *Center for Colon Cancer Research, University of South Carolina, Columbia, USA*

^†^Denotes co‐first authors.

**Introduction:** Although colon cancer has the second highest cancer mortality rate, targeted treatments are improving survival. 5‐Fluoroucil (5‐FU) is a common chemotherapeutic used to treat colon cancer. Cancer therapy can adversely affect many tissues and processes including skeletal muscle. However, 5‐FU's effects on skeletal muscle quality and function are not known.

**Purpose:** Therefore, we examined the effect of a 5‐FU treatment regime on indices of skeletal muscle wasting in a pre‐clinical model of colon cancer.

**Methods:** At 12 weeks of age, after intestinal and colon polyp development, male Apc^Min/+^ mice were treated with PBS (*n* = 10) or 5‐FU (n = 10) once every two weeks for six weeks. At 18 weeks of age muscles were examined for inflammation, protein turnover, and mitochondrial signaling associated with wasting.

**Results**: Both PBS (−6 ± 2%) and 5‐FU (−4 ± 1%) mice lost bodyweight and were initiating cachexia. Absolute grip strength and triceps surae muscle mass were higher with 5‐FU treatment. Muscle inflammatory signaling (p‐STAT, p‐65) was suppressed by 5‐FU. Protein turnover regulation was improved by 5‐FU (increased p70S6K, decreased Atrogin‐1), and 5‐FU also increased quadriceps muscle ribosomal capacity. Additionally, 5‐FU treatment rescued muscle metabolic signaling associated with AMPK, DRP‐1, and PGC‐1α. Autophagy‐related signaling (LCB II: I, BNIP3, p62) was decreased by 5‐FU.

**Discussion:** Collectively, we report that the 5‐FU treatment to tumor bearing mice improved indices of muscle quality related to the wasting phenotype. Future studies will need to delineate the potential mechanisms regulating 5‐FU's protective effects on muscle metabolic and functional capacities independent to cancer‐induced body weight loss.

**Acknowledgements:** Supported by NCI R01‐CA121249 and NIGMS 5P30GM103336.

**3-12**

**Purine nucleotide‐mediated enhancement of mitochondrial density and bioenergetics improves skeletal muscle wasting phenotypes via an AMPK/PGC1α/β‐independent pathway**

Cara A. Timpani^1,2^, Craig A. Goodman^1,2,3^, Christos G. Stathis^1,2,3^, Alan Hayes^1,2,3^ and Emma Rybalka^1,2,3^

^1^ *College of Health & Biomedicine, Victoria University, Melbourne, Australia;* ^2^ *Australian Institute for Musculoskeletal Science (AIMSS), Melbourne, Australia;* ^3^ *Institute of Sport, Exercise & Active Living (ISEAL), Victoria University, Melbourne, Australia*

**Introduction:** In the fatal X‐linked skeletal muscle wasting disease, Duchenne Muscular Dystrophy (DMD), the progressive degeneration of skeletal muscle coincides with impaired mitochondrial function, alterations in structural networking, the inability to respond to metabolic stress signaling and the decline of cellular energy stores^1^. Similar metabolic signatures are observed in other skeletal muscle wasting phenotypes such as cachexia^2^, diabetic myopathy^3^, chronic obstructive pulmonary disease^4^ and sarcopenia.^5^ We have investigated whether the purine nucleotide, adenylosuccinic acid (ASA), a mitochondrial and purine nucleotide cycle stimulant, can be used to enhance mitochondrial dynamics, cellular bioenergetics and attenuate skeletal muscle wasting in the *mdx* mouse model of DMD.

**Methods:** Four‐week‐old control (C57Bl/10; CON) and dystrophic *mdx* mice were treated with 3000 μg·mL^−1^ ASA in drinking water (~350 mg·kg^−1^ day^−1^) for 8 weeks. Skeletal muscle mitochondrial density, viability, oxidative function and superoxide (O^2−^) content, alongside metabolic stress response signaling (adenosine monophosphate protein‐activated kinase (AMPK) and peroxisome proliferator‐activated receptor gamma coactivator‐1α/β (PGC1α/β) and histopathology, including fiber cross‐sectional area, damage/regeneration, pseudohypertrophy and fat and fibrotic tissue infiltration, were assessed.

**Results:** ASA treatment increased mitochondrial density (by >200% in isolated *flexor digitorum brevis* (FDB) fibers (p < 0.01) and ~50% in *tibialis anterior* (TA) cross‐sections (*p* < 0.0001) and viability (by ~20% in FDB fibers; *p* < 0.05), as well as the bioenergetical profile of TA muscles, including phosphocreatine (p < 0.01) concentration. ASA also reduced O^2−^ production by ~25% (p < 0.05). These changes were independent of AMPK and PGC1α/β activation in *mdx* quadriceps. ASA treatment significantly attenuated skeletal muscle damage (p < 0.01) and wasting (p < 0.05) in *mdx* TA, as well as downstream histopathological features including pseudohypertrophy (p < 0.05), centronucleated fibers (p < 0.01), lipid accumulation (p < 0.05) and connective tissue infiltrate (*p* < 0.001).

**Conclusions:** Our data highlight a protective effect of ASA on skeletal muscle‐wasting phenotypes by stimulating unknown molecular pathways to enhance mitochondrial density and viability, as well as cellular bioenergetics.

**3-13**

**Chronic tobacco smoke exposure induces aryl hydrocarbon receptor‐dependent neuromuscular junction degeneration**

Kayla Miguez^1^, Madhusudanarao Vuda^2^, Vita Sonjak^1^, Daren Elkrief^1^, Yana Konokhova^3^, Anna Perez^2^, Angela Rico de Souza^2^, Carolyn J. Baglole^2^ and Russell T. Hepple^3^

^1^ *Department of Kinesiology & Physical Education, McGill University, Montreal, QC, Canada;* ^2^ *Meakins Christie Laboratories and Research Institute of the McGill University Health Centre, Montreal, QC, Canada;* ^3^ *Department of Physical Therapy, College of Health & Health Professions, University of Florida, Gainesville, FL, USA*

**Introduction:** Chronic tobacco smoke (TS)‐related diseases, such as cancer, cardiovascular disease and Chronic Obstructive Pulmonary Disease, are associated with common elements of skeletal muscle deterioration (fast fiber shift, erosion of oxidative capacity and atrophy) that worsen clinical outcomes, including increasing the risk of death. Although chronic TS exposure is an exacerbating factor in conditions involving neuromuscular junction degeneration, including aging and amyotrophic lateral sclerosis (ALS), there are as of yet no data addressing the impact of chronic TS exposure on the neuromuscular junction and how this relates to the muscle alterations induced by TS. Furthermore, although chronic activation of the aryl hydrocarbon receptor (AHR) is linked to neurotoxicity, and the AHR responds to multiple compounds in TS, no studies have considered the role of the AHR in mediating the adverse impact of chronic TS exposure on muscle.

**Methods:** In the first set of experiments, we exposed 8‐month‐old C57Bl6 male mice to 60 minutes of TS exposure twice daily, 5 days per week, for 16 weeks. Following the final TS exposure, we examined muscle mass, neuromuscular junction morphology by confocal microscopy, and oxidative capacity by high‐resolution respirometry. In the second set of experiments, we determined whether whole body knockout of the AHR would attenuate the neuromuscular junction impact of 8 weeks of TS exposure in 3‐month‐old male mice.

**Results:** 16 weeks of TS induced mild atrophy of limb muscle that was associated with neuromuscular junction degeneration and mild erosion of oxidative capacity. Strikingly, AHR knockout completely prevented the TS‐induced neuromuscular junction degeneration and reduced indices of stress in homogenates of spinal cord tissue.

**Conclusions:** Chronic TS exposure induces neuromuscular junction degeneration that is dependent upon the AHR, providing novel insights into the mechanisms by which chronic TS adversely impacts skeletal muscle.

**3-14**

**Overview of beneficial effects of beta‐hydroxy‐beta‐methylbutyrate (HMB) supplementation during inactivity: from physical parameters to molecular signaling**

Thomas Brioche, Rémi Roumanille, Allan F. Pagano, Angèle Chopard and Guillaume Py

*Université de Montpellier, INRA, UMR 866 Dynamique Musculaire et Métabolisme, Montpellier, France*

**Introduction:** Inactivity is the main factor during aging leading to physical alterations, such as muscle atrophy, strength reduction, balance and coordination impairment, and aerobic fitness decrease. Muscle atrophy and strength decrease can be explained by an impairment of muscle protein balance characterized by a decreased protein synthesis and an increased proteolysis. Also, a decrease in mitochondria content and functions are involved in the impairment of aerobic fitness observed during aging. These mechanisms are affected by numerous upstream factors including a decrease in the release of anabolic hormones, increased pro‐inflammatory cytokines production and an increase in muscle oxidative stress. Each of these adaptations could turn to serious health deterioration during aging. Beta‐hydroxy‐beta‐methylbutyrate (HMB), a leucine metabolite, has been described in normal condition to increase aerobic fitness and prevents muscle atrophy during cancer through enhanced mitochondria functions, anti‐catabolic and anabolic mechanisms. We hypothesized that HMB could be used as a nutritional countermeasure to prevent physical deconditioning due to inactivity.

**Methods:** Mature C56Black6J male mice were hindlimb‐unloaded (HU) or kept ambulatory for 14 days. Mice were provided either HMB (250 mg/kg body mass per day) or distilled water by oral gavage for 21 days. Aerobic fitness was evaluated and a multiple static rod test assessed coordination and balance. Histological and molecular analyses were done on soleus muscle and plasma.

**Results and Discussion:** HMB treatment counteracted soleus slow and fast‐twitch fibers atrophy. Aerobic fitness, coordination and balance were also counteracted in HU treated mice. These results can be explained by a better protein balance in treated mice compared to placebo mice as shown by higher protein synthesis and lower proteolysis in mice receiving HMB. According to our results, lower oxidative stress and inflammation could explain the better protein balance in treated mice.

**Conclusions:** HMB could thus be envisaged as an efficient nutritional countermeasure.

Our work is supported by grants from the French “Centre National d'Etudes Spatiales” (CNES).

**3-15**

**Overexpression of G6PD delays the onset of frailty in mice acting on muscle mass and metabolism**

Coralie Arc‐Chagnaud^1,2^, Andrea Salvador‐Pascual^1^, Thomas Brioche^2^, Angèle Chopard^2^, Guillaume Py^2^, Mari Carmen Gomez‐Cabrera^1^ and José Viña^1^

^1^ *Freshage Research Group ‐ Dept. Physiology ‐ University of Valencia, CIBERFES, INCLIVA, Valencia, Spain;* ^2^ *University of Montpellier, INRA, UMR866, Dynamique Musculaire et Métabolisme, Montpellier, France*

**Introduction:** Frailty is a clinical syndrome associated with the aging process, which leads to a decreased physical function. The damages induced by reactive oxygen species (ROS) in cells accentuate this process. The antioxidant system is largely based on the reducing power of NADPH, whose levels are mainly determined by the enzyme glucose‐6‐phosphate dehydrogenase (G6PD).

**Material and methods:** Using a specific model of Tg‐mice overexpressing G6PD, we measured oxidative stress parameters, cross‐sectionnal area (CSA) of muscle fibers, markers regulating protein synthesis, mitochondrial dynamics and apoptosis. We used a cohort of WT and G6PD‐Tg old female mice (from 18 to 26 months old) to evaluate the evolution of 5 functional parameters and establish a score for frailty based on the construct described by Linda Fried. CSA and molecular markers were measured on 21 months old mice.

**Results:** Our results relative to functional test demonstrated that 18–20 and 23–24 months old G6PD‐Tg animals performed better in the motor coordination test than the WT. At the age of 23–24 months, 45% of WT‐mice were considered frail for maximal strength vs 4.8% in the G6PD‐Tg group. Taking into account the 5 different parameters, we founded that the percentage of mice considered as frail is higher in the WT than in the G6PD‐Tg group. G6PD‐Tg mice exhibited higher muscle fiber CSA, levels of reduced glutathione (GSH), lower levels of apoptosis and our results suggest a higher protein synthesis in muscle tissue.

**Conclusions:** Finally, overexpression of G6PD in mice prevents frailty in 18 to 26 months old mice acting on muscle mass and metabolism (protein balance, oxidative stress, mitochondria and apoptosis).

**3-16**

**Skeletal muscle alterations in an obese rat model of heart failure with preserved ejection fraction (HFpEF) are not reversed by exercise training**

T. Scott Bowen^1^, Christian Herz^1^, Natale P.L. Rolim^2^, Gustavo di Silva^2^, Ulrik Wisloff^2^ and Volker Adams^1^

^1^ *Department of Internal Medicine and Cardiology, Leipzig University‐Heart Center, Leipzig, Germany;* ^2^ *K.G. Jebsen Center of Exercise in Medicine, Department of Circulation and Medical Imaging, Faculty of Medicine, Norwegian University of Science and Technology, Trondheim, Norway*

**Introduction:** Despite normal left ventricular ejection fraction (LVEF), patients with heart failure and preserved left ventricular ejection fraction (HFpEF) present exercise intolerance which can be attenuated by exercise training. Alterations in skeletal muscle have been described in HFpEF, yet the underlying mechanisms involved as well potential benefits mediated by exercise training remain unknown. The present study, therefore, used a cardiometabolic rat model of HFpEF to further elucidate: 1) the skeletal muscle alterations induced; and 2) the effects of aerobic exercise training during secondary prevention.

**Methods:** After 20 weeks, obese Zucker diabetic fatty/spontaneously hypertensive heart failure F1 hybrid (ZSF1) rats (*n* = 12) were compared to their lean counterparts (*n* = 8), with a further 3 groups of obese ZSF1 rats assessed 8 weeks later following sedentary behavior (*n* = 15), moderate‐continuous training (MCT; *n* = 11) or high‐intensity interval training (HIIT; n = 11) on a treadmill.

**Results:** Obese rats evidenced HFpEF: diastolic dysfunction, LV hypertrophy, and exercise intolerance were present (*P* < 0.05) with LVEF preserved. HFpEF rats demonstrated ~20% reduction (P < 0.05) in both fiber cross‐sectional area and capillary‐to‐fiber ratio in the extensor digitorum longus (EDL) skeletal muscle, which were not reversed by exercise training. These alterations were further associated with a reduction in absolute maximal force by ~30% and greater fatigability by ~20% in all HFpEF groups compared to controls.

**Conclusions:** A cardiometabolic obese rat model of HFpEF was associated with skeletal muscle atrophy, impaired capillarity, and functional deficits, yet these alterations could not be reversed by aerobic exercise training. Whether these findings translate to patients, whereby exercise training has limited efficacy in treating skeletal muscle impairments in HFpEF, requires further investigation.

**3-17**

**Inhibition of TGF‐β pathway protects diaphragm from both muscle atrophy and weakness during chronic sepsis**

Baptiste Jude^1^, Florine Tissier^1^, Mickael Droguet^1^, Karelle Léon^1^, Marie‐Agnès Giroux‐Metges^1,2^ and Jean‐Pierre Pennec^1^

^1^ *EA 4324 ORPHY, IBSAM, UFR Médecine et Sciences de la Santé, Université de Bretagne Occidentale, Brest, France;* ^2^ *Explorations Fonctionnelles Respiratoires, CHRU de Brest, Brest, France*

**Introduction:** Diaphragm weakness is a risk factor leading to prolonged mechanical ventilation, increased morbidity and death for patients in intensive care units. Muscle dysfunction is related to an unbalance between protein synthesis and breakdown. Glucocorticoids and pro‐inflammatory cytokines such as TNF‐α and IL‐6 play an important role in the activation of calpain and ubiquitin proteasome system. Since few years, myostatine, a member of TGF‐β family, seems to strongly trigger activation of protein breakdown. The aim of this study was to investigate the potent benefit effect of a TGF‐ β receptor inhibitor in muscle function during chronic sepsis.

**Methods:** Chronic sepsis was induced by cecal ligation and puncture (CLP) and carried out during 7 days. Three groups were realized: sham group, septic group, and septic group with daily intra peritoneal injection of TGF‐βRI inhibitor (LY364947 at 1 mg/kg) starting 24 h after sepsis induction (LY group). Rats were sacrificed 7 days after sepsis induction, and the diaphragm was removed for muscle contraction recordings then weighted. Mean values are compared to sham group.

**Results:** Chronic sepsis led to a significant decrease of 20% of diaphragm mass (176.7 ± 15 g vs. 221.1 ± 12.4 g in sham group) but with inhibitor treatment, the mass was not significantly modified (218.1 ± 20.8 g). Concerning muscle contraction, absolute force was decreased in septic group by approximatively 35% (75 ± 16 g vs. 115.4 ± 9 g in sham group), and in LY group the maximal force was restored to 109.7 ± 8 g. In the same manner, the specific force was decreased by 32% in septic group (92.3 ± 21 g/cm^2^ vs. 135.2 ± 8 g/cm^2^ in sham group) whereas there was no difference in LY group (140.7 ± 10 g/cm^2^).

**Conclusions:** This work demonstrates that the inhibition of TGF‐β pathway can protect muscle such as diaphragm from weakness and proteolysis during chronic sepsis. This could be of a major importance in the care of septic patients.

**3-19**

**Effects of β hydroxy‐β‐methylbutyrate on liver regeneration, skeletal muscle protein balance, and amino acid concentrations in partially hepatectomized rats**

Milan Holeček and Melita Vodeničarovova

*Department of Physiology, Charles University, Faculty of Medicine in Hradec Kralove, Czech Republic*

**Introduction:** Beta‐hydroxy‐beta‐methylbutyrate (HMB) is a leucine metabolite used as a nutritional supplement for preservation of muscle mass in elderly and muscle wasting disorders, and as an ergogenic aid in exercise. Although most of the HMB is produced in the liver, there are no reports of the effects of HMB in subjects with liver injury. We examined the effects of HMB on liver regeneration, muscle protein balance, and amino acid concentrations in rats after partial (68%) hepatectomy (PH).

**Methods:** HMB (200 mg/kg/day) or saline were administered using osmotic pumps to PH or laparotomized rats for 7 days. The animals were then sacrificed by exsanguination and the liver, m. soleus (SOL, slow‐twitch, red muscle), and m. extensor digitorum longus (EDL, fast‐twitch, white muscle) were quickly removed and weighed. Blood plasma and tissue samples were kept in −80 °C until analyses. ANOVA and Bonferroni multiple comparisons procedure were used for statistical analysis of the results.

**Results:** In PH animals we found lower concentrations of glucose, triglycerides and urea, and higher concentrations of creatinine, bilirubin, alanine, and ketoisovalerate in blood plasma, lower weights and protein contents in EDL, and lower weights and protein and DNA contents in the liver when compared with laparotomized animals. In PH rats treated by HMB we found higher concentrations of urea, triglycerides, branched‐chain amino acids (BCAA; valine, leucine, isoleucine) and branched‐chain keto acids, and lower concentrations of glucose and creatinine in blood plasma, higher BCAA concentrations in muscles and in the liver, and higher DNA contents in the liver than in saline treated animals. The effect of PH on muscle weights and protein contents was in HMB treated rats insignificant.

**Conclusions:** The results indicate that HMB administration affects metabolism of the BCAA and has favourable effects on protein balance in muscles and liver regeneration in PH animals.

**3-20**

**Metabolic reprogramming in order to polarize macrophages and to sustain skeletal muscle regeneration**

Stefania Gorini^1†^, Laura Vitiello^1†^, Maurizia Caruso^2^, Francesca De Santa^2^, Giuseppe Rosano^1,3^ and Elisabetta Ferraro^1^

^1^ *Laboratory of Pathophysiology of Cachexia and Metabolism of Skeletal Muscle, IRCCS San Raffaele Pisana, Rome, Italy;* ^2^ *Institute of Cell Biology and Neurobiology (IBCN), National Research Council of Italy (CNR), Rome, Italy;* ^3^ *Cardiovascular and Cell Sciences Institute, St George's University, London, UK*

^†^These authors have equally contributed to this work

**Introduction:** Macrophages play different roles in tissue homeostasis, as they are involved in defense from pathogens, debris removal as well as in tissue repair. Their effects are related to their activation/polarization state, which depends on the stimuli they receive. Two major MF phenotype can be distinguished; M1, a proinflammatory phenotype, and M2, a pro‐tolerogenic and tissue repair‐favoring profile.

On regenerating skeletal muscle, M1 inhibit myogenic precursor cell fusion while M2 promote satellite cell differentiation to form mature myotubes. It has been demonstrated *in vivo*—included in humans—that macrophages sequentially orchestrate adult myogenesis during regeneration of damaged skeletal muscle where differentiating myogenin‐positive myogenic precursor cells preferentially couple to anti‐inflammatory macrophages.

Interestingly, the activation and polarization of macrophages is associated to a well‐defined metabolic reprogramming and a robust association between the metabolic state and the phenotype of macrophages exists. In particular, M1 obtains energy mainly by glycolysis while M2 relies mostly on the oxidative metabolism. Several drugs, defined metabolic modulators, have been extensively studied in cardiology and their ability to reprogram the metabolism of the myocardium and to optimize the production of energy has been assessed.

**Methods:** Here, we treated un‐stimulated and M2‐stimulated macrophages with metabolic modulators, *in vitro*. Moreover, we administered trimetazidine (TMZ), an MM also used in clinical practice, to mice recovering from skeletal muscle damage due to cardiotoxin.

**Results:** We showed that metabolic modulators are capable to enhance M2 polarization in vitro, inducing the expression of M2‐related genes both in M2‐stimulated macrophages and in un‐stimulated macrophages. We also observed that in injured muscle of TMZ treated mice there is a decrease in the number of muscle infiltrating macrophages.

**Conclusions:** Taken together, these preliminary data suggest that metabolic modulators can be used to modulate macrophage polarization, which might be useful to sustain tissue regeneration.

**3-21**

**Skeletal muscle function might be affected by Mineralocorticoid Receptor activation.**

Alessandra Feraco^1^, Francesca Molinari^1^, Massimiliano Caprio^2^, Andrea Armani^2^, Elisabetta Ferraro^2^ and Giuseppe Rosano^1,3^

^1^ *Laboratory of Pathophysiology of Cachexia and Metabolism of Skeletal Muscle, IRCCS San Raffaele Pisana, Rome, Italy;* ^2^ *Laboratory of Cardiovascular Endocrinology, IRCCS San Raffaele Pisana, Rome, Italy;* ^3^ *Cardiovascular and Cell Sciences Institute, St George's University of London, Cranmer Terrace, London, UK*

**Background and aims:** Mineralocorticoid Receptor (MR) modulation affects adipocyte function, and MR antagonism has been shown to improve glucose metabolism in a mouse model of diet‐induced obesity. Moreover, there is evidence that MR inhibition affects insulin signaling pathway in skeletal muscle. Here, we aim at elucidating the role of MR in regulating skeletal muscle homeostasis.

**Methods:** We analysed MR activation in a murine myoblast cell line (C2C12), and in parallel, we characterized the impact of MR activation on signaling pathways involved in skeletal muscle metabolism in obese mice.

**Results:** We observed an increase in MR protein expression in C2C12 myotubes during differentiation *in vitro*. Treatment of myotubes with aldosterone induced an increase in serum/glucocorticoid‐regulated kinase1 (SGK1) protein phosphorylation. In addition, aldosterone increased activation of AMP‐activated *protein* kinase (AMPK), a key regulator of muscle metabolism, and Acetyl‐CoA carboxylase (*ACC*), a downstream target of AMPK signaling. Importantly, spironolactone was able to revert these effects, suggesting that MR affects muscle fibers energy metabolism.

We also evaluated gastrocnemius expression of MR and metabolic profile in ob/ob mice. Reduction in glucose transporter type 4 (GLUT4), peroxisome proliferator‐activated receptor‐gamma coactivator 1 alpha (PGC1 alpha) and mitochondrial transcription factor A (mtTFA) protein levels in skeletal muscle of obese mice suggested the occurrence of metabolic alterations in glucose uptake and mitochondrial function. Surprisingly, SGK1 and AMPK protein phosphorylation were reduced in obese mice, thus indicating downregulation of MR activity. This hypothesis was confirmed by real‐time qPCR, which revealed a reduction in MR and SGK1 mRNA levels in obese mice gastrocnemius.

**Conclusions:** Our data indicate a downregulation of MR activity in skeletal muscle of obese mice concomitant with impaired muscle metabolism and suggest a causal role of reduced MR activity in metabolic alterations of skeletal muscle fibers in obesity.

**3-23**

**Genes differentially expressed during reversion of androgen‐dependent skeletal muscle atrophy**

Flavia A. Guarnier^1†^, Priscila de O. Coelho^2†^, Leonardo Bruno Figueiredo^3^, Livia S. Zaramela^2^, Rosely O. Godinho^3^ and Marcelo D. Gomes^2^

^1^ *Laboratory of Pathophysiology and Muscle Adaptation, State University of Londrina, Brazil;* ^2^ *Department of Biochemistry and Immunology, Ribeirao Preto Medical School, University of São Paulo, Brazil;* ^3^ *Department of Pharmacology, Escola Paulista de Medicina, Federal University of Sao Paulo, São Paulo, Brazil*

^†^Equal contributed first author

Muscle wasting or atrophy is a condition associated with major human systemic diseases including, diabetes, cancer, and kidney failure, among others. There is accumulating evidence from comparison of transcriptional profiles that a common set of genes, termed atrogenes, are modulated in atrophyi ng muscles. However, the transcriptional changes that trigger reversion or attenuation of muscle atrophy have not been characterized at the molecular level. To identify key factors involved in the recovery of skeletal muscle mass, we have used cDNA microarrays to investigate genes differentially expressed during the atrophy reversion of the androgen‐sensitive levator ani muscle (LA), in the well‐established model of castration and testosterone replacement. The data obtained in microarray assay showed that 310 genes were differentially expressed in LA muscles 24 hours after hormone replacement. The statistical analysis of the p value allowed the selection of some targets to be validated through qPCR. As expected, most of them behave as atrogenes and responded to castration‐induced atrophy. Strikingly, 7 genes (APLN, DUSP5, IGF1, PIK3IP, KLHL38, PI15, and MKL1) did not respond to castration but exclusively to the hormone replacement. We therefore proposed to name these genes antiatrogens. Considering that almost all proteins encoded by these genes may function as regulators modulators of cell proliferation/growth, our results open new perspectives in signaling pathway on atrophy‐related syndromes field, bringing to light also a new perspective for therapeutic approaches.

**3-24**

**In vitro assessment of postnatal myonuclear accretion**

Anita E.M. Kneppers^1^, Lex B. Verdijk^2^, Chiel C. de Theije^1^, Luc J.C. van Loon^2^, Annemie M.W.J. Schols^1^ and Ramon C.J. Langen^1^

^1^ *Departments of Respiratory Medicine;* ^2^ *Human Biology and Movement Sciences; NUTRIM School of Nutrition and Translational Research in Metabolism, Maastricht University Medical Centre, Maastricht, the Netherlands*

**Introduction:** Postnatal myogenesis is essential for skeletal muscle regeneration and relies on satellite cell proliferation, differentiation and subsequent fusion with muscle fibers (i.e., myonuclear accretion). *Ex vivo*, myogenesis is primarily studied using the formation of syncytia during myoblast differentiation, which represents aspects of developmental myogenesis, but may incompletely portray postnatal myogenesis. We aimed to develop an *in vitro* model that better reflects postnatal myonuclear accretion (PNMA) and evaluated the effects of known modulators of muscle plasticity.

**Methods:** Mononuclear C2C12 myoblasts were added to differentiated myotubes and co‐cultured for 3 days. Postnatal myonuclear accretion (PNMA) was assessed by live cell time‐lapse imaging, and cell tracing by cell labelling with Vybrant® DiD and DiO. Furthermore, a Cre/LoxP‐based cell system was developed to quantitatively assess PNMA, by the conditional expression of luciferase upon myoblast–myotube fusion, which was detected using luminometry.

**Results:** Live cell time‐lapse imaging, staining‐based cell tracing, and recombination‐dependent luciferase activity, revealed PNMA *in vitro*. Treatment of co‐cultures with the myogenic factor IGF‐I (*p* < 0.001) and the cytokines IL‐13 (*p* < 0.05) and IL‐4 (p < 0.001) increased PNMA, while the myogenic inhibitors Cytochalasin D (p < 0.001), Myostatin (p < 0.05), and TNFα (p < 0.001) decreased PNMA. Furthermore, PNMA was increased upon recovery from contraction‐induced myotube damage (*p* = 0.052) and LY29004‐induced atrophy (p < 0.001). Moreover, siRNA mediated knockdown of Myomaker in myoblasts (p < 0.001), but not in myotubes, decreased PNMA.

**Conclusions:** We developed a sensitive model system for quantitative assessment of *in vitro* postnatal myonuclear accretion for the study of physiological and pathological modulators of myogenesis, which allows distinction between cell type specific roles of signals and responses in the regulation of postnatal myogenesis.

**3-26**

**The overexpression of PGC‐1α in the skeletal muscle affects myogenesis**

Marc Beltrà, Fabrizio Pin, Riccardo Ballarò, Ambra Iannuzzi, Fabio Penna and Paola Costelli

*Department of Clinical and Biological Sciences, University of Turin, Italy, Interuniversity Institute of Myology, Italy*

**Introduction:** Peroxisome proliferator‐activated receptor γ coactivator 1α (PGC‐1α) is a master regulator of mitochondrial biogenesis. In skeletal muscle, PGC‐1α expression is induced by exercise. Along this line, transgenic MCK‐PGC‐1α mice, which overexpress this transcription factor specifically in the skeletal muscle, are characterized by enhanced exercise performance in comparison with wild‐type animals; this is mainly due to increased myofiber mitochondrial content that results in markedly improved energy metabolism. In addition to an increased proportion of oxidative fibers *vs* glycolytic ones, we found a high number of fibers with centrally located nuclei, which is indicative of muscle regeneration. Moreover, myogenic stem cells are more abundant in transgenic mice compared to wild‐type animals. When cultured in differentiating medium, cells isolated from PGC‐1α mice form myotubes larger than those generated by cells derived from wild‐type animals. Starting from this point, the aim of the study was to investigate if stem cells from MCK‐PGC‐1α mice can improve myogenesis.

**Methods:** Muscles from male wild‐type and MCK‐PGC‐1α mice were subjected to mild digestion, and mononuclear cells were isolated by filtration. These cells were then transplanted into the *tibialis anterior* muscle of female wild‐type mice, either injured (BaCl_2_ i.m. injection 8 hours before cell transplantation) or not. The animals were euthanized 12 days after BaCl2 injection.

**Results:** Hematoxylin/eosin staining of muscles transplanted with WT‐derived cells shows improved regeneration. On the contrary, all the muscles injected with MCK‐PGC‐1α‐derived cells show an increase of centrally located nuclei, altered myofiber cross‐sectional area distribution and marked SDH staining. The expression of molecular markers of regeneration (Pax7, MyoG) and mitochondrial content (Tom20, COX‐IV) is consistent with the histological pattern.

**Conclusions:** The results obtained in the present study suggest that cells isolated from MCK‐PGC‐1α donor mice are able to fuse with recipient muscle myofibers partially inducing a shift towards oxidative metabolism and affecting regeneration.

**3-27**

**Resistance exercise prevents cancer‐induced muscle wasting in Ehrlich tumor‐bearing mice**

Rafael Deminice^1^, Mayra J. Testa^1^, Camila S. Padilha^1^, Fernando T. Frajacomo^1^, Poliana C. Marinello^1,2^, Paola S. Cella^1^, Felipe A. Moura^1^, Jose A. Duarte^3^, Rubens Cecchini^2^ and Flávia A. Guarnier^2^

^1^ *Department of Physical Education, State University of Londrina, Brazil;* ^2^ *Department of Pathology, State University of Londrina, Brazil;* ^3^ *CIAFEL, Faculty of Sport, University of Porto, Porto, Portugal*

**Background and aim:** Resistance exercise training (RET) is known to stimulate protein synthesis and skeletal muscle hypertrophy in health subject. However, RET effects on tumor‐induced cachexia and muscle wasting are poorly known. We aimed to evaluate cachexia and skeletal muscle plasticity responses to RET after Ehrlich tumor cell inoculation.

**Methods:** Swiss mice were divided randomly into 4 groups: control (C), tumor‐bearing (T), exercised (E) and tumor‐bearing exercised (TE). Animals were inoculated with Ehrlich tumor cells (1 × 10^6^ suspension in PBS) before RET that was performed for the following 4 wks. RET protocol consisted of climbing a ladder apparatus with progressive load weights tied to the animal's tail in E and TE groups, while the physical activity of C and T rats was confined to the space of the cage.

**Results:** Ehrlich tumor grew progressively (*P* < 0.05) reaching 15% of animal's body weight after 4 wks. Tumor growth promoted muscle wasting demonstrated by decreased gastrocnemius CSA (−28%, *P* < 0.05), body weight loss (−15%, *P* < 0.05) and decreased fat content. Increased (*P* < 0.05) systemic leukocytes and pro‐inflammatory interleukins were also demonstrated in T animals compared to C. In contrast, RET was able to mitigate the reduced body weight and muscle wasting by the attenuation of systemic inflammatory markers. RET also prevented loss of muscle strength and locomotor capacity associated with tumor development in both experiments. Pro‐inflammatory panel attenuation may down‐regulates protein degradation pathways in skeletal muscle and contributes to the preventive anti‐atrophy effects of RET. In addition, the Erlich tumor microenvironment analysis demonstrated RET reduced the proliferation of tumor cells and increases the necrotic tumor area without change tumor volume.

**Conclusions:** RET prevented muscle wasting by attenuating tumor‐induced systemic pro‐inflammatory. Our data also suggest RET reduces tumor proliferation cells and tumor aggressiveness.

Supported by Capes‐PVE #88881.068035/2014‐01

**3-28**

**Walker‐256 tumour growth affects the ^1^H‐NMR based metabolomic profile of skeletal muscle more severely in young‐host compared to adult rats**

Ophélie Ocean Orvoën^1,2^, Derly Floriano^1^, Bread Leandro Cruz^1^, Lais Rosa Viana^1^, Carla de Moraes Salgado^1^, Rogerio William dos Santos^1^, Luiz Alberto Ferreira Ramos^1^ and Maria Cristina Cintra Gomes‐Marcondes^1^

^1^ *Laboratory of Nutrition and Cancer, Department of Structural and Functional Biology, Biology Institute, University of Campinas (UNICAMP), Brazil;* ^2^ *Université d'Angers, Angers, France*

**Introduction:** Cancer cachexia is an important clinical problem, which reduces the life expectancy, mainly due to the marked body weight loss. The tumour evolution leads to skeletal muscle mass wasting, mediated by the proteolysis and/or reduced protein synthesis. We aimed to evaluate, by a time‐course study, the tumour growth correlation to the cachectic state and also to muscle tissue metabolomic profile in Walker‐256 tumour‐bearing rats.

**Methods:** Adult Wistar rats (A; 90 days) and young (Y; 21 days) were submitted to tumour implant (2 × 106 viable cells). The animals euthanasia occurred at a different time of tumour evolution (7th, 9th and 14th day for young; 7th, 14th and 21st day for adults) and compared to non‐tumour‐bearing rats. The carcass, muscle and tumour weights were measured at each euthanasia day. The metabolomic analysis of skeletal muscle samples were accessed by using high‐resolution ^1^H‐NMR spectroscopy.

**Results:** We observed that young tumour‐bearing rats had an increase in carcass‐to‐initial body weight ratio (58% higher; *P* = 0.0003), associated with a higher liver‐to‐carcass weight (67%; *P* < 0.0001) when compared to A group. The cachexia index was bigger in young than adult group (Y > A, 2.4×; *P* = 0.0001). Even though, the muscle‐to‐carcass weight ratio was similar in Y and A groups, as well as the tumour weight rate. The ^1^H‐NMR analysis of muscle tissue showed differences mainly in the content of methylhistidine (Y > A, 10× higher; *P* = 0.0186), succinate (Y > A, 4.2×; *P* = 0.0255), dimethylglycine (Y > A, 12×, *P* < 0.0001), and other metabolites. These differences showed some impacted metabolic pathway such as nitrogen metabolism (*P* = 0.0128); alanine/aspartate/glutamate metabolism (*P* = 0.0339), and citrate cycle (TCA cycle; *P* = 0.0283) (analysed using http://www.metaboanalyst.ca).

**Conclusions:** The tumour evolution reduced the survival time in young rats (14 days) comparing to the adult group (21 days), leading to severe damage effects mainly related to muscle wasting, showing substantial implications in a young host.

**3-29**

**Akt‐mTOR pathway contribution to the skeletal muscle wasting attenuation effect of resistance exercise in Walker 256 tumor‐bearing rats**

Camila S. Padilha^1^, Fabrício A. Voltarelli^2^, Poliana C. Marinello^1^, Mayra T.J. Testa^1^, Paola S. Cella^1^, Philippe B. Guirro^1^, Kessi C. Iarosz^1^, Lilian E.C.M. Silva^3^, Alceu Afonso Jordão^3^, José A. Duarte^4^, Rubens Cecchini^1^, Flávia A. Guarnier^1^ and Rafael Deminice^1^

^1^ *State University of Londrina, Londrina, PR, Brazil;* ^2^ *Federal University of Mato Grosso, Cuiabá, MT, Brazil;* ^3^ *Faculty of Medicine of Ribeirão Preto, University of São Paulo, Ribeirão Preto, SP, Brazil;* ^4^ *CIAFEL, Faculty of Sport, University of Porto, Porto, Portugal*

**Introduction:** Resistance exercise (RE) is known to up‐regulate Akt‐mTOR signaling for protein synthesis in skeletal muscle; however, RE effect on tumor‐induced muscle wasting is poorly known. We aimed to investigate the Akt‐mTOR pathway contribution to the skeletal muscle wasting prevention promoted by resistance exercise in tumor‐bearing rats.

**Methods:** Thirty‐seven Wistar rats were divided into 4 groups: control (C,n = 9), tumor‐bearing (T,n = 9), exercised (E,n = 9), and tumor‐bearing exercised (TE,n = 10). Walker‐256 tumor cells were implanted in the right flank of T and TE groups. Forty‐eight hours after tumor implantation the animals started a four‐week period of RE. RT protocol consisted of climbing a ladder apparatus with weights tied to the animal's tail in E and TE groups, while the physical activity of C and T rats was confined to the space of the cage.

**Results:** The Walker‐256 tumor grew progressively (*P* < 0.05) reaching 4% of animal's body weight after 4 wks. Tumor growth caused a reduced body weight gain (−9.85%) and muscle weight loss. Tumor growth also decreased muscle strength (−11.09%), promoted skeletal muscle atrophy (P75 = −24%) and increased skeletal muscle collagen deposit (+87.5%). In addition, elevated systemic leukocytes (+150%), TNF‐α (+30.3%) and mRNA muscle levels of Atrogin‐1 (+204%), but not changed mTor skeletal muscle mRNA level were demonstrated in T rats compared to C. In contrast, RE was able to attenuate loss of body weight (+6.35%) and skeletal muscle weight (+12.5%). However, no changes in Akt‐mTOR were demonstrated in tumor‐bearing rats after RE. Exercised tumor‐bearing animals presented attenuated muscle atrophy (P75 = −1.12%) and collagen deposition, probably by attenuating elevated systemic leukocytes (−31.5%) inflammatory interleukins and elevated mRNA Atrogin‐1 levels (−44.6%) caused by tumor growth.

**Conclusions:** RE attenuates cachexia development and muscle wasting, by downregulating tumor‐induced systemic inflammation and proteolysis signaling, but not to enhances Akt‐mTOR signaling.

Supported by Capes‐PVE #88881.068035/2014‐01

**3-30**

**Catwalk XT system (natural gait evaluation) as a skeletal muscle functional measurement in preclinical study of cancer cachexia**

Laís Rosa Viana^1^, William F. Vieira^1^, Natalia Tobar^2^, Gabrielly Machado^1^, Sílvio Roberto Consonni^3^, Bianca Castelucci^3^, Alexandre Leite Rodrigues de Oliveira^1^ and Maria Cristina Cintra Gomes‐Marcondes^1^

^1^ *Laboratory of Nutrition and Cancer, Department of Structural and Functional Biology, Biology Institute, University of Campinas (UNICAMP), Brazil;* ^2^ *Nuclear Medicine Service of the Clinical Hospital of UNICAMP, Brazil;* ^3^ *Department of Biochemystry and Tecidual Biology, Biology Institute, University of Campinas (UNICAMP), Brazil*

**Introduction:** Cancer cachexia is a wasting syndrome characterised by involuntary weight loss caused mainly by skeletal muscle loss associated or not with mobilisation of adipose tissue. Additionally, muscle function could be severely jeopardised leading to a bad quality of life and poor treatment outcomes. Few preclinical studies evaluate the loss of muscle function in experimental models of cancer cachexia. Therefore, the aim of this study was to evaluate whether CatWalk test (natural gait evaluation) could be used to measure muscle function in cancer cachexia preclinical studies.

**Methods:** As preclinical model of cancer cachexia, Wistar rats were subcutaneously injected in the right flank with Walker 256 carcinoma (2.5 × 10^6^ viable cells). Animals performed CatWalk test previously (health) and posterior (pre‐agonic state) of tumour inoculation. For CatWalk analysis, we considered maximal contact area (cm^2^), print area (cm^2^) and maximal intensity of hind limb and forelimb paws. We also evaluate muscle mass (g), muscle fiber cross sectional area (cm^2^) and fat mass using Dual‐Energy X‐Ray Absorptiometry (DEXA).

**Results:** Walker‐256 tumour growth led to cachexia state manifested by significant weight loss (*P* = 0.001) due to loss of skeletal muscle mass (18.7%, *P* = 0.03), decreased muscle fiber cross section area (43.5%, *P* = 0.0001) and also by fat loss (35.8%, *P* < 0.0001). CatWalk measurements at pre‐agonic state showed a significant reduction in the maximal contact area and the print area of hind limb paws (46.9%, *P* = 0.0196 and 46.1%, *P* = 0.0112) and the maximal paw footprint intensity decreased in both fore and hind limb (5.83%, *P* = 0.0024 and 6.8%, *P* = 0.0143).

**Conclusions:** The high sensitivity of the motor dynamic function analysis by the CatWalk method was able to characterise the loss of muscle function in this experimental model of cachexia and also showed that these functional parameters were correlated with the other morphometric parameters analysed.

**3-31**

**Mitochondrial dysfunction promotes cancer‐induced cardiac and respiratory muscle weakness**

Michael P. Wiggs^1^, Brandon M. Roberts^2^, Oh‐Sung Kwon^3^, Jeung‐Ki Yoo^3^, Demetra D. Christou^3^, Andrew R. Judge^2^, David D. Fuller^2^, Hazel H. Szeto^4^ and Ashley J. Smuder^5^

^1^ *Department of Health and Kinesiology, University of Texas at Tyler, Tyler, TX, USA;* ^2^ *Department of Physical Therapy, University of Florida, Gainesville, FL, USA;* ^3^ *Department of Applied Physiology and Kinesiology, University of Florida, Gainesville, FL, USA;* ^4^ *Department of Pharmacology, Weill Cornell Medical College, New York, NY, USA;* ^5^ *Department of Exercise Science, University of South Carolina, Columbia, SC, USA*

**Introduction:** Cancer cachexia is a syndrome characterized by profound cardiac and diaphragm muscle wasting, which increase the risk of morbidity in cachectic patients due to failure of the cardiorespiratory system. In this regard, muscle relies greatly on mitochondria to meet energy requirements for contraction and mitochondrial dysfunction can result in muscle weakness and fatigue. In addition, mitochondria are a major source of reactive oxygen species (ROS) production, which can stimulate increased rates of muscle protein degradation. Therefore, it has been suggested that mitochondrial dysfunction may be an underlying factor that contributes to the pathology of cancer cachexia.

**Methods:** To determine if inhibition of mitochondrial dysfunction is sufficient to prevent cancer‐induced muscle dysfunction, colon 26 (C‐26) tumor‐bearing mice were administered either saline or the mitochondrial peptide SS‐31 daily (3 mg/kg/day). Specifically, SS‐31 is a multifunctional peptide that has been demonstrated to preserve mitochondrial function during atrophic conditions by inhibiting cardiolipin peroxidation and reducing ROS production.

**Results:** C‐26 mice treated with saline demonstrated greater rates of ROS emission and mitochondrial uncoupling compared to C‐26 mice receiving SS‐31 in both the heart and diaphragm muscle. In addition, SS‐31 administration to C‐26 mice attenuated both cardiac and diaphragm muscle dysfunction. Indeed, cancer cachexia‐induced alterations to the myocardial performance index and the percentage fractional shortening were significantly rescued in C‐26 mice treated with SS‐31. In the diaphragm, muscle fiber cross‐sectional area of C‐26 mice treated with saline was significantly reduced, and force production was impaired compared to C‐26, SS‐31 treated animals. Finally, ventilatory deficits were also attenuated in tumor‐bearing animals treated with SS‐31, compared to those treated with saline.

**Conclusions:** These data demonstrate that cancer promotes severe cardiac and respiratory myopathy and that prevention of mitochondrial dysfunction is sufficient to preclude cancer cachexia‐induced cardiorespiratory dysfunction.

**3-32**

**Multitargeted tyrosine kinase inhibitors imatinib, sorafenib and sunitinib perturb energy metabolism and cause cytotoxicity to C2C12 murine skeletal muscle cell line**

Vijaya L. Damaraju^1^, Michelle Kuzma^2^, Carol E. Cass^1^ and Michael B. Sawyer^1,2^

^1^ *Department of Oncology, University of Alberta;* ^2^ *Cross Cancer Institute, Edmonton, AB, Canada*

**Introduction:** Tyrosine kinase inhibitors (TKIs) have improved cancer treatment and prognosis but have also resulted in side effects that include fatigue, diarrhea, hypothyroidism, and other toxicities. Because TKI effects on mitochondrial function are thought to play a role in cardiac myocyte dysfunction, we investigated TKI effects on skeletal muscle as a possible explanation of TKI fatigue. Mitochondrial dysfunction can result from inhibition of oxidative phosphorylation complexes and superoxide generation. It is also likely that inhibition of key transporters involved in uptake of glucose and/or nucleosides could also result in alteration of energy metabolism or mitochondrial mass. In this work, we investigated these processes in cultured C2C12 murine skeletal muscle cells.

**Methods:** We studied imatinib, sorafenib and sunitinib effects in C2C12 cells grown under conditions that result in formation of myotubes by measuring mitochondrial complex activity, ATP production, caspase activation, mitochondrial membrane potential, reactive oxygen species (ROS) generation and nucleoside and glucose uptake.

**Results:** Imatinib was cytotoxic and inhibited complex V activity. Imatinib had negligible effects on ATP levels, ROS generation or membrane potentials, but stimulated glucose uptake. Sunitinib caused cytotoxicity, ATP level depletion, apoptosis activation, increase in ROS, and decrease in mitochondrial membrane potential and nucleoside and glucose uptake. Sorafenib was cytotoxic with rapid activation of caspase 3/7 activity. Sorafenib inhibited complex III and V quite potently, but there was no increase in generation of mitochondrial superoxide, although depolarization of mitochondrial membranes occurred very rapidly with complete loss at 5–10 μM sorafenib.

**Conclusions:** Imatinib, sunitinib and sorafenib through different mechanisms caused changes in mitochondrial complex activities, glucose and nucleoside uptake leading to decreased energy production and decreased mitochondrial function in a skeletal muscle cell model, suggesting that these effects play a role in fatigue, one of the most common TKI side effects.

**3-33**

**Pericyte in the adult muscle satellite cell niche: a key player maintaining the steady state and helping recovery**

Koumaiha Zeynab^1^, Baptiste Périou^2^, Muriel Rigolet^2^, Frederic Relaix^1,3^, Romain Gherardi^2,3^ and Peggy Lafuste^1^

^1^ *INSERM IMRB U955, Team 10 group 1, University of Paris‐Est Creteil, France;* ^2^ *INSERM IMRB U955, Team 10 group 2, University of Paris‐Est Creteil, France;* ^3^ *Henri Mondor Hospital, Department of Pathology, University of Paris‐Est Creteil, France*

**Introduction:** Muscle growth and post‐injury regeneration are supported by muscle satellite cells (mSCs) that reside beneath the myofiber basement membrane in close proximity to capillaries. We previously showed that pericytes play a key role in the microvascular niche of mSCs during post‐natal stages of muscle growth, by promoting post‐natal myogenesis through IGF‐1 and inducing mSC quiescence ending the accretion phase through Angiopoietin‐1. Since 90% of capillaries have pericyte coverage at the end of muscle growth, we investigated the role of pericytes in the adult mSC niche.

**Material et methods:** We used TNAP‐CreERT2 mice crossed with R26RDTR^stoploxP/stoploxP^, Angiopoietin‐1^loxP/loxP^, and R26RmT^loxPmG/loxPmG^ animals to generate respectively conditional models for diphteria toxin‐induced muscle depletion of microvascular cells, ablation of microvascular Angiopoietin‐1 and fluorescent tracing of microvascular cells.

**Results:** Conditional muscle pericyte depletion could not be used due to extensive, presumably ischaemic, muscle necrosis. In contrast, selective Cre recombinase ablation of Angiopoietin‐1 gene in TNAP^+^ pericytes induced release of adult mSCs from quiescence. Following chemical injury, the ablation caused delayed muscle regeneration with persistently cycling Pax7^+^ mSCs. Selective Type 2 fiber hypotrophy was observed consistently with prominent association of TNAP‐expressing microvessels with type 2 fibers observed in TNAP‐GFP reporter mice.

**Conclusions:** We conclude that pericytes associated with endothelial cells exert paracrine effects on adjacent myogenic cells that are essential to maintain adult muscle homeostasis and during muscle repair.

**4-01**

**Circulating hormones and neuropeptides profile in cachectic colorectal cancer patients**

Estefanía Simoes Fernández^1,2^, Joanna Darck Carola Correia Lima^1^, Raquel Galvão Figueredo^1^, Emidio Marques de Matos‐Neto^1^, Gabriela Salim de Castro^1^, Fang Chia Bin^5^, José Pinhata Otoch^1,3,4^, Paulo Sergio Martins de Alcantara^3^, Alessandro Laviano^6^ and Marília Seelaender^1,4^

^1^ *Cancer Metabolism Research Group, University of São Paulo, Brazil;* ^2^ *Department of pathological anatomy, University Hospital, University of São Paulo, Brazil;* ^3^ *Department of Clinical Surgery, University Hospital, University of São Paulo, Brazil;* ^4^ *Faculdade de Medicina, University of São Paulo, Brazil;* ^5^ *Santa Casa de Misericórdia de São Paulo, Brazil;* ^6^ *Department of Clinical Medicine, Sapienza University of Rome, Italy*

**Background:** Cancer Cachexia is a devastating and multifactorial syndrome involving changes in several metabolic pathways, in many tissues and organs. It is defined as a muscle‐wasting syndrome, with or without adipose tissue atrophy. Anorexia, fatigue, asthenia, anaemia, insulin resistance and systemic inflammation are symptoms commonly related to the syndrome, leading to a poor prognosis and reduced survival. Furthermore, circulating hormones and neuropeptides, directly related to peripheral signals from the Central Nervous System, could play a key role contributing in the regulation of energy homeostasis and influencing food intake.

**Aim:** To evaluate circulating hormones and neuropeptides and to elucidate possible modified pathways related to appetite regulation and weight control in cancer cachexia patients.

**Methods:** Patients with colorectal cancer were divided into Weight‐Stable Cancer (**WSC**, *n* = 42) and Cachectic Cancer (**CC**, *n* = 44) groups. Blood sampling was performed after signature of the informed consent form. Hormones and neuropeptides were quantified with Luminex® xMAP technology.

**Results:** Serum samples from cachectic patients presented significant lower levels of circulating amylin and insulin, compared to WSC (*p* < 0.0004 and *p* < 0.0001, respectively). Moreover, Leptin (*p* < 0.0002) and gastric inhibitory polypeptide (GIP) (*p* < 0.0009) were both significantly decreased in CC patients. Finally, no differences were observed between WSC and CC for the other hormones (Glucagon, C‐Peptide, pancreatic polypeptide (PP), Peptide YY (PYY); glucagon‐like peptide‐1 (GLP‐1); Ghrelin). Finally, the neuropeptides Orexin A (*p* < 0.0195) and Oxytocin (*p* < 0.004) were decreased in CC patients, maybe contributing to the behavioural changes generally related to the syndrome.

**Conclusions:** The results show robust modulation by cachexia of patient circulating hormone and neuropeptides, factors directly related to appetite regulation, energy homeostase and food intake. For that reason, the results point out to a possible beneficial effect of hormone therapy. Furthermore, neuropeptide changes might be related with behavioural changes, a major problem in cachexia that remains poorly investigated.

**4-02**

**TAPT: its role in tumoral microenvironment**

Nelson Inácio Pinto Neto^1^, Diego Alexandre Cavalaro^2^, Valter Tadeu Boldarine^1^, Ana Claudia Losinskas Hachul^1^, Joanna Darck Carola Correia Lima^2^, Marilia Seelaender^2^, Claudia Oller do Nascimento^1^ and Lila Missae Oyama^1^

^1^ *Universidade Federal de São Paulo, São Paulo, Brazil;* ^2^ *Universidade de São Paulo, São Paulo, Brazil*

**Introduction:** The adipose tissue is an endocrine organ that secretes a wide range of molecules, including the adipokines. Adipocytes also produce angiogenic and growth factors that contribute to progression of solid tumors such VEGF and TGF‐β, respectively. These adipokines and the peritumoral adipose tissue may have an important role in cancer biology and carcinogenesis, considering the possible crosstalk between the peritumoral adipose tissue and tumour.

**Methods:** 16 patients enrolled in this study was divided as follows: patients with cancer without cachexia‐WSC (*n* = 7) and patient with cancer cachexia‐CC (*n* = 9). The study was approved by the Ethics Research Committee (972.914). Samples of peritumoral adipose tissue were removed for determination of TNF‐α, IL‐1β, STAT‐1, STAT‐3, RANTES, IL‐1Ra, IOp‐10, IL‐15, MCP‐1, IFN‐α, GCSF, FADD, and TGF‐β concentration. Also, correlation between these proteins and cytokines was analyzed.

**Results:** TNF‐α and FADD, factors involved with apoptosis, were significantly higher in CC as compared to WSC. Also, IL‐1β and STAT‐1 were significantly lower in CC as compared to WSC. In the TAPT of CC, a significant positive correlation of RANTES with IL‐1Ra and IP‐10 and a negative correlation (RANTES/IFN‐α) were found. Analysis of correlation of GCSF with the protein content of IL‐1Ra, IP‐10, IL‐15 and MCP‐1 showed a significant negative relationship and a positive correlation with IFN‐α. In TAPT of WSC no significant correlations were detected between RANTES, GCSF, IL‐3, FADD and STAT‐1 and cytokines/chemokines analyzed.

**Conclusions:** These results indicate that inflammatory and tumorigenic pathways are altered in peritumoral adipose tissue during cancer cachexia. Furthermore, we demonstrate that in peritumoral adipose tissue of cachectic patients, inflammatory cytokines are correlated with growth factors, pointing to a modulation of the proliferative environment in close proximity to the tumor by inflammatory cytokines.

**4-03**

**Sexual dimorphism in human cancer cachexia: a preliminary study into miRNA/mRNA expression in human skeletal muscle**

Ashok Narasimhan^1^, Russell Greiner^1^, Oliver F. Bathe^2^, Vickie Baracos^1^ and Sambasivarao Damaraju^1^

^1^ *University of Alberta, Edmonton, Canada;* ^2^ *University of Calgary, Calgary, AB, Canada*

**Introduction:** Cancer cachexia (CC) is a multifactorial syndrome characterized by severe depletion of skeletal muscle, with or without fat loss. Gene expression studies have identified several genes associated with CC; however, sex‐specific expression differences have never been studied.

**Aim:** To profile microRNAs (miRNAs) and differential gene expression at the isoform level (DEI) in human skeletal muscle biopsies to understand the sex‐specific expression differences in CC and mRNA isoform regulation by miRNAs.

**Methods:** 42 cancer patients were classified into cachectic cases based on International diagnostic consensus framework for CC, and non‐cachectic controls—cancer patients who were weight stable for six months compared to pre‐illness weight. While 9 men and 13 women were cachectic cases, 9 men and 11 women were non‐cachectic controls. Next generation sequencing and Affymetrix Human Transcriptome array 2.0 were used to identify miRNAs and DEI, respectively. Representative miRNAs and DEI were validated using qRT‐PCR. Differentially expressed (DE) miRNAs and DEI were identified at 1.4 FC at *p* < 0.05 using Partek genomics suite 6.6.

**Results:** 10 DE miRNAs (9 in autosomes and 1 in X chromosome) were identified in men, and 3 DE miRNAs (autosomal) were identified in women, and none were common between the two sexes. 1324 and 372 DEI were identified in men and women respectively and showed limited overlap (3%). qRT‐PCR results showed cross‐platform concordance for both miRNA and DEI. Validation of DE miRNAs in an independent dataset confirmed these findings. While skeletal muscle atrophy associated pathways were prominent in men, adipogenesis pathways were seen in women. TGFB1 was identified as a common upstream regulator in both sexes. However, their downstream targets were different.

**Conclusions:** The preliminary study identifies sex‐specific expression in CC at the miRNA, DEI and at the pathway levels. Functional characterization of these molecules is warranted to delineate their mechanisms in CC pathophysiology.

**4-04**

**Displaced myonuclei in cancer cachexia suggest altered innervation**

Dario Coletti^1,2^, Hassani Medhi^1,2^, Nissrine Daou^1^, Matos Emidio^3^, Raquel Galvao Figueredo^3^, Gabriela Salim De Castro^3^, Viviana Moresi^2^, Adamo Sergio^2^, Li Zhenlin^1^ and Marilia Seelaender^3^

^1^ *Dept. of Biological Adaptation and Ageing B2A (CNRS UMR 8256 ‐ INSERM ERL U1164 ‐ UPMC P6), Pierre et Marie Curie University Paris 6, France;* ^2^ *DAHFMO Unit of Histology and Medical Embryology, and Interuniversity Institute of Myology, Sapienza University of Rome, Italy;* ^3^ *Institute of Biomedical Sciences, University of Sao Paulo, Brazil*

**Introduction:** Central nuclei are considered a hallmark of skeletal muscle fiber regeneration and a sign of myopathy. We and others have previously observed central myonuclei in both pre‐cachectic patients and animal models of cancer cachexia, in striking contrast with the reduced regenerative potential characterizing cachectic muscles.

**Methods:** To elucidate the mechanisms underlying these divergent observations we further characterised the nature of central nuclei, and the muscle fiber involved, in both pre‐ and cachectic cancer patients, as well as in C26‐tumor‐bearing mice. Muscle with different physiological properties, the *Rectus abdominis* and the *Tibialis anterior*, was analysed in search of denervation and regeneration markers.

**Results:** We observed rare though measurable, non‐peripheral myonuclei. The latter consisted of two distinct population: bona fide central nuclei and nuclei that are neither central nor subsarcolemmal, which we named «displaced» myonuclei. Displaced and central nuclei were detected in all types of muscle fibers. However, displaced myonuclei were the only ones to vary between control and cachectic muscles, suggesting a link with the pathological condition. Non‐peripheral myonuclei were observed in the absence of muscle regeneration molecular markers, such as embryonic or fetal myosin expression. The analysis of longitudinal sections revealed that displaced myonuclei are clustered as it occurs upon denervation, and that the number of nuclear clusters increases in cachexia, coincident with N‐CAM upregulation, another denervation marker. Additional denervation markers were upregulated in both human and murine cachectic muscles.

**Conclusions:** We observed a novel phenomenon in cancer patients and animal models of cachexia: the presence of displaced nuclei consistent with motor neuron loss or NMJ perturbation. This phenomenon could underlay a previously neglected phenomenon of denervation rather that myofiber damage and regeneration in cachexia. Like in aging, denervation‐dependent myofiber atrophy could contribute to the atrophic process leading to muscle wasting.

**4-05**

**Cachexia mechanisms in cancer through the lens of transcriptomics**

Sarah Kolitz, Kevin D. Fowler, Jason M. Funt, Jenny Zhang, Matthew Ung, Renan Escalante‐Chong, Andrew C. Lysaght, Gregory Koytiger, Yoonjeong Cha and Benjamin Zeskind

*Immuneering Corporation, Cambridge, MA, USA*

**Introduction:** Cachexia is a major clinical complication that affects up to 80% of patients with advanced cancer, and by some estimates is responsible for 20% of cancer deaths 1. Despite its role in cancer mortality, no approved therapies are yet available, and the mechanisms underlying cachexia remain to be fully elucidated.

**Methods:** Gene expression profiling provides a quantitative means to systematically assess biological differences between tissue from patients who develop cachexia and from those who do not. Here, we report cachexia‐related findings from computational analysis of publicly accessible gene expression datasets from various biological contexts, including a dataset with adipose tissue from cancer patients with and without cachectic weight loss (E‐GEOD‐51931) 2.

**Results:** Genes and pathways identified by these analyses include currently known cachexia mechanisms, and also emerging mechanisms that have not historically been emphasized in cachexia research. Some of these emerging mechanisms may help to more fully explain the consequences of the well‐known association between inflammation and cachexia and support the previously described importance of Pax7 in cachexia 3.

**Conclusions:** Transcriptomics is a powerful approach for dissecting the molecular underpinnings of cancer cachexia and prioritizing areas for drug development. Our findings suggest new avenues for experimental follow‐up and highlight new possibilities for the treatment of cachexia in cancer patients.

**4-06**

**Blocking adipose tissue lipolysis does not prevent muscle wasting in an experimental cancer cachexia model**

Sílvia Busquets^1,2^, Marta Castillejo^1^, Cristina Moreno^1^, Ecem Tuna^1^, Marina Bantulà^1^, Tessa van Daalen^1^, Francisco J. López‐Soriano^1,2^ and Josep M. Argilés^1,2^

^1^ *Cancer Research Group, Departament de Bioquímica i Biomedicina Molecular, Facultat de Biologia, Universitat de Barcelona, Barcelona, Spain;* ^2^ *Institut de Biomedicina de la Universitat de Barcelona (IBUB), Barcelona, Spain*

**Introduction and Purpose:** A previous studyReference through genetic ablation of adipose triglyceride lipase (ATGL) concluded that blocking the lipolytic machinery prevented the loss of muscle wasting in mouse models of cancer cachexia (Lewis lung carcinoma and B16 melanoma). Bearing this in mind, the objective of the present study was to block all the lipolytic enzimes present in adipose tissue (using chemical inhibitor) to see if muscle wasting could be avoided using this pharmacological strategy.

**Methods:** We used **Acipimox** to block lipolysis, **Atglistatin** to inhibit ATGL, and **Compound 12** to inhibit hormone sensitive lipase (HSL).

**Results:** The results obtained clearly show that, despite the decrease in circulating free fatty acids caused by the inhibition of lipolytic enzymes, no changes in the muscle loss associated with cancer cachexia were observed.

**Conclusions:** It is therefore concluded that preventing adipose tissue wasting does not influence muscle atrophy associated with cancer.

**4-07**

**Long non‐coding RNA profiling in human skeletal muscle and their role in Cancer Cachexia**

Bhumi Bhatt^1^, Ashok Narasimhan^1^, Oliver Bathe^2^, Vickie E. Baracos^1^ and Sambasivarao Damaraju^1^

^1^ *University of Alberta, Edmonton;* ^2^ *University of Calgary, Calgary, AB, Canada*

**Introduction:** lncRNAs (long non‐coding RNAs, >200 bp) regulate gene expression by transcriptional, post‐transcriptional and epigenetic mechanisms. lncRNAs originating in gene regions may regulate the coding genes. lncRNAs act as decoys and scaffolds for miRNAs and transcription factors, respectively, to regulate gene expression. lncRNAs are known to be dysregulated in a tissue‐ and disease‐specific context. Expression and regulation of lncRNAs in human skeletal muscle under conditions of cancer cachexia remains to be elucidated.

**Aim:** i) To profile lncRNAs from human skeletal muscle biopsies from cachectic and non‐cachectic cancer patients; ii) to identify differentially expressed (DE) lncRNAs; iii) to identify muscle‐specific transcription factor gene expression to explain transcriptional regulation of lncRNAs.

**Methods:** Study subjects (*n* = 40) were classified as cachectic cases and non‐cachectic controls based on international consensus diagnostic criteria (high %weight loss with sarcopenia or low BMI). Total RNA was extracted from muscle biopsies and mRNA and lncRNAs were profiled using Human Transcriptome Array‐2.0. Data were analysed using Partek Genomics Suite. DE analysis was done using one‐way ANOVA, and lncRNAs were annotated using Gencode database. Annolnc database was used for functional insights into lncRNAs.

**Results:** 62 lncRNAs were identified as DE (fold change range −1.2 to 1.58 and *p* < 0.05), of which 13 were down‐regulated and 49 were up‐regulated in cachectic cases. Reciprocal expression of the DE representative lncRNA‐nearest mRNA pairs noted were RP11‐420G6.4‐SERPINB9P1; CTA‐292E10.6‐001‐XBP1; RP11‐587D21.4‐001‐BDNF; and RP11‐196G18.22‐HIST2H2BC, suggestive of transcriptional regulatory mechanisms.

We predicted transcription factor binding sites in the lncRNAs and a subset of these also showed DE in muscle: e.g. ZNF143, CHD1/2, SMC3, HDAC2, NFYB, MEF2C; these need to be validated in functional assays.

**Conclusions:** We identified DE lncRNAs associated with cancer cachexia. Our current efforts are also to identify how lncRNAs act as decoys to sequester miRNAs and contribute to the pathophysiology of cachexia.

**4-08**

**Blood cells in cancer cachexia: it is easy to spot the difference**

Ana Flávia Pessoa^1,2,3,4^, Raquel G. Figuerêdo^1^, Paula C. Leme^1^, Katrin Radloff^1^, Luis Henrique Andrade^1^, Fernanda Formiga^5^, Ana Paula Lepique^1^, Alessandro Laviano^6^, Paolo S. Alcantara^5^ and Marilia Seelaender^1^

^1^ *Institute of Biomedical Sciences University of São Paulo, São Paulo, SP, Brazil;* ^2^ *University of São Paulo Medical School (FMUSP), São Paulo, SP, Brazil;* ^3^ *Federal University of Piaui, Teresina, PI, Brazil;* ^4^ *Irmandade da Santa Casa de Misericórdia de São Paulo, São Paulo, SP, Brazil;* ^5^ *University Hospital of the University of São Paulo, São Paulo, SP, Brazil;* ^6^ *Department of Clinical Medicine, Sapienza University of Rome, Rome, Italy*

**Background:** Cachexia is a multifactorial syndrome whose hallmark is weight loss and systemic inflammation. Despite the relevance of cachexia as a health system burden, finding feasible diagnostic methods remains a challenge.

**Aim:** Characterization of blood cellularity and biochemical parameters in cancer cachexia.

**Methods:** Non‐cancer patients (CONTROL), weight stable (WSC) and cachectic gastrointestinal cancer (CC) patients participated in the study approved by the University of São Paulo Biomedical Sciences Institute (CEP 788/07) and University Hospital Ethics Committee (CEP 752/07, SISNEP CAAE: 0031.0.198.019.07). Informed consent was obtained from all participants. Patient group division was based on the criteria proposed by Evans *et al*., 2008. Blood samples were collected to evaluate the protein expression of CCL‐2 and CCL‐3 using Multiplex Magpix® technology. The numbert of lymphocytes, neutrophil, platelets and MDSCs (Myeloid Derived Suppressor Cells) was assessed by flow cytometry.

**Results:** The number of lymphocytes was decreased in CC, whereas that of platelets, neutrophils, the neutrophil‐lymphocyte ratio (NLR) and the PLR (Platelets Lymphocytes Ratio) were increased in CC when compared to CONTROL and WSC. In CC, the concentration of circulating chemokine CCL‐2 was higher than that of WSC patients.

**Conclusions:** Our results demonstrate increased NLR and PLR ratios as systemic inflammatory parameters in cancer cachexia. The easy evaluation of NLR, PLR makes them promising diagnostic tools. The increased platelets levels and the higher number of circulating MDSCs also seem to be specific for the immune phenotype of CC patients.

**5-01**

**The TGF‐b family cytokine MIC‐1/GDF15 has differential effects on lean mass in normal and obese mice**

Samuel N. Breit^1,2^, Hong Ping Zhang^1^, Rakesh Manandhar^1^, Yasmin Husaini^1,2^, Michelle Lee‐Ng^1^, Helene Lebhar^2^, Christopher P. Marquis^2^, Amanda Sainsbury^3^, David A. Brown^1,2^ and Vicky W.W. Tsai^1,2^

^1^ *St Vincent's Centre for Applied Medical Research, St Vincent's Hospital Sydney, NSW, Australia;* ^2^ *The University of New South Wales, Sydney, NSW, Australia;* ^3^ *The University of Sydney, The Boden Institute of Obesity, Nutrition, Exercise & Eating Disorders, Charles Perkins Centre, NSW, Australia*

**Introduction:** MIC‐1/GDF15 is a physiological appetite regulator, which when markedly overproduced in some disease states, such as advanced cancer, causes an anorexia/cachexia syndrome mediated, at least in large part, by its actions on appetite control centres in the brain. Antibodies to MIC‐1/GDF15 have been proposed as therapeutics for patients with some anorexia cachexia syndromes and recombinant MIC‐1/GDF15 protein as an obesity therapeutic.

**Methods:** In order to better understand how prolonged elevation of MIC‐1/GDF15 might impact animals with differing adiposity, we have infused normal chow fed mice and those with diet induced obesity, with either vehicle or highly purified, yeast derived, recombinant murine MIC‐1/GDF15 (0.5 ug/gBW/day), delivered over 28 days, by an osmotic minipump.

**Results:** Steady‐state serum MIC‐1/GDF15 levels in these mice reached an average of about between 10 and 12 ng/ml, levels that are commonly reached in patients with advanced cancer. Over this time, both groups of mice experienced a substantial reduction in body weight in association with a reduction in energy intake (13–15%) that was similar in both groups. The reduction in body weight was significantly greater in obese than in lean mice (15.54 ± 1.5% vs 10.28 ± 1.12%). Moreover, weight loss in obese mice was due exclusively to loss of fat mass, with no significant loss of lean mass. In contrast, the normal mice showed significant reductions in both lean and fat mass. MIC‐1/GDF15 effectively corrected the pro‐inflammatory metabolic abnormalities that frequently accompany obesity in both this mouse model and as is well known to occur in humans.

**Conclusions:** The differential effects of MIC‐1/GDF15 on lean and obese mice may explain the relative protection from the anorexia/cachexia syndrome afforded by having an increased BMI or obesity.

**5-03**

**Identification of tumor‐borne mediators secreted *via* exosomes triggering alterations in fat and muscle tissues in cancer cachexia**

Victor Laurent, Sören Fisker‐Schmidt, Maria Rohm, Stephan Herzig and Mauricio Berriel Diaz

*Institute for Diabetes and Cancer (IDC), Helmholtz Center Munich, Neuherberg, Germany, and Joint Heidelberg‐IDC Translational Diabetes Program, Heidelberg University Hospital, Heidelberg, Germany*

**Introduction:** Cancer cachexia is strongly associated with different types of tumors and negatively affects quality of life, efficacy of tumor treatment and survival of cancer patients. Despite its clinical importance, the identity of tumor‐borne signals inducing wasting and their impact on specific peripheral organ systems remain mostly unknown. Exosomes have been shown to be key mediators of the crosstalk between tumor cells and their microenvironment. Nevertheless, only very few studies have focused on the role of tumor cells derived‐exosomes on cancer cachexia.

**Methods:** In an unbiased differential secretome analysis of colon cancer cells‐derived exosomes combined with high‐throughput adipocytes and myotubes phenotyping, we defined a list of exosomal proteins which potentially had cachexia‐inducing properties.

**Results:** By using overexpression strategy in HEK293 cells, different mouse models for both colorectal and pancreatic cancer and human serum samples, we confirmed that tumor‐derived exosomal proteins contributed to skeletal muscle atrophy and adipose tissue wasting *in vitro and in vivo*.

**Conclusions:** Our study suggests that tumor cell‐derived exosomes are important mediators of different components of cancer cachexia, which could potentially provide novel opportunities for diagnostic and therapeutic measures to alleviate cachexia, thereby contributing to improved disease outcomes.

**5-04**

**Comparison of whole genome gene expression in (cancer) cachectic muscles of animal models and human patients**

Rogier L.C. Plas^1^, Guido J.E.J. Hooiveld^1^, Renger F. Witkamp^1^ and Klaske van Norren^1,2^

^1^ *Division of Human Nutrition, Wageningen University and Research, Wageningen, The Netherlands;* ^2^ *Nutricia Research, Utrecht, The Netherlands*

**Background:** Different animal models are used to study mechanisms and treatment options of (cancer) cachexia. Next to this, experimental conditions may vary for the same model. In some studies, whole genome gene expression of muscle tissue is measured using micro array analysis. Upon publication of these gene expression studies, raw data are often made available via the NCBI Gene Expression Omnibus database. By comparing these datasets, differences and similarities between models and experimental conditions can be examined. Moreover, knowledge can be gathered on underlying processes causing muscle wasting and possible differences between animal models for cancer cachexia and human cancer cachexia. We are currently comparing different datasets containing muscle gene expression of animal models and human tissue (on‐line available) and one of our own new and unpublished datasets containing microarray data of heart muscle and *m. extensor digitorum longus* and *m. soleus* of mice from a C26 model.

**Aims:**

1. Examine differences and commonalities in whole genome gene expression between different muscle tissues in (cancer) cachexia mouse models.
2. Examine differences and commonalities in whole genome gene expression between (cancer) cachexia mouse models and muscle from cachectic colon cancer patients.

**Methods:** All datasets (our own and those online available) are quality checked, normalised and annotated using the latest CDF available. Comparisons are made both on gene and on pathway level.

**Hypotheses:** We hypothesise that main differences exist between different models and that smaller differences occur due to different muscle types analysed for a certain model. Moreover, we hypothesise that these smaller differences are due to different effects of cachexia on specific muscle fibre types. We also hypothesize that some models mimic the human situation better than others.

Currently, analysis is ongoing. At the conference the data on comparison of different animal models and human samples will be available and presented.

**5-05**

**The effect of n‐3 fatty acid‐derived amines and gut‐derived bacterial compounds on excretion of tumour‐derived inflammatory mediators**

Klaske van Norren, Mieke Poland, Rogier Plas, Frouwkje Politiek, Milena Banic, Jocelijn Meijerink and Renger Witkamp

*Nutrition & Pharmacology group, Wageningen University, Wageningen, The Netherlands*

**Aim:** To investigate the role N‐3‐derived‐acyl‐amines on the release of cachexia‐inducing inflammatory‐mediators by colon adenocarcinoma (C26) cells.

**Background:** N‐3 fatty acids have been reported to be able to reduce the cachectic inflammatory response. The effectivity of the anti‐cachectic response on N‐3 fatty acids, however, seems to vary between patients and study populations. We hypothesize that the diversity of the response to N‐3 fatty acids might be due to the ability of an individual to form specific endogenous metabolites (acyl‐amines) from these fatty acids.

**Methods:** C26 cells were incubated for 24 h in the presence and absence of Docosahexaenoic acid (DHA), docosahexaenoyl ethanolamide (DHEA) and docosahexaenoyl serotonin (DHA‐5‐HT). To stimulate IL‐6 release, cells were incubated with lipopolysaccharide (LPS) and Pam3CysSerLys4 (Pam_3_CSK_4_). Interleukin (IL)‐6 and PGE2 release were measured using enzyme‐linked immunosorbent assays (ELISAs). Data represent the average of *n* = 3 independent experiments. Statistical analysis was performed using one‐way analysis of variance (ANOVA) and Dunnett's post hoc testing. A p‐value <0.05 was considered statistically significant.

**Results:** PGE2 excretion was inhibited by both DHEA and DH‐5‐HT, but not significantly by DHA. Incubation with 0.5 μg/ml LPS or Pam_3_CSK_4_ was needed to induce IL‐6 excretion, which was not measurable in unstimulated cells. 1 μM DHA‐5‐HT reduced the LPS‐ and Pam_3_CSK_4_‐induced IL‐6 release. Both DHA and DHEA had no effect on IL‐6 release.

**Conclusions:** Unstimulated cells produced PGE2, but did not produce IL‐6. This is surprisingly because the C26 mouse model shows an IL‐6‐dependent development of cachexia. We therefore hypothesize that passage of bacterial compounds through the gut‐barrier might trigger the excretion of tumour‐derived cachexia‐inducing inflammatory mediators in this model. Acyl‐amine‐derivatives of DHA were far more effective than DHA in inhibiting the excretion of PGE2 and IL6. These results suggest that the metabolic fate of food derived n‐3 fatty acids might influence the anti‐cachectic properties of these fatty acids.

**5-06**

**Tumour growth changed the serum metabolomic profile in ageing rats**

Rafaela Rossi Rosolen, Lais Rosa Viana, Rogerio Willian Santos and Maria Cristina Cintra Gomes‐Marcondes

*Laboratory of Nutrition and Cancer, Department of Structural and Functional Biology, Biology Institute, University of Campinas (UNICAMP), Brazil*

**Introduction:** The neoplasia development promotes host's biochemical and metabolic alterations that culminate in the involuntary weight loss mainly due to spoliation of muscle protein. This process results in a severe syndrome called cachexia, which reduces the quality of life and survival in cancer patients. This study aimed to analyse the tumour evolution and the alterations in metabolic pathways during the ageing process in Walker‐256 tumour‐bearing rats.

**Methods:** Ageing Wistar rats (400 days old) were distributed into six different groups: Ageing control (CS); Walker‐256 tumour‐bearing rats euthanized in various days of tumour evolution: group euthanized at 11th (W11); 14th (W14); 17th (W17); 21st (W21) and 25th (W25) days. The tumour implant occurred by a single inoculation of 2 × 10^6^ viable neoplastic cells in the right flank subcutaneous. These all groups were also compared with adult control (CA; 90 days old). The animals' serum samples were collected, filtered and processed to access the metabolomic profile analysis by using high‐resolution ^1^H‐NMR spectroscopy to evaluate the metabolites/pathways that may be involved in the process of cancer cachexia.

**Results:** The tumour evolution showed an exponential growth curve, associated with marked loss of body weight. The effects of tumour growth led to an crescent increase in concentration of allantoin (W25 > S, 1.8‐fold; *P* = 0.0047), 2‐hydroxybutyrate (W25 > S, 10.7‐fold; *P* = 0.0108), tryptophan (W25 > S, 2.6‐fold; *P* = 0.0011) and n‐methylhistidine (W25 > S, 12.2‐fold; *P* = 0.0027), associated with marked reduction of serum glutamine (W25 80% lower; *P* = 0.0317) and glutamate (W25 34% lower; *P* = 0.0112). These changes led to impacted metabolic pathways such as glutamine/glutamate metabolism (*P* = 0.00001), nitrogen metabolism (*P* = 0.0128) and alanine/aspartate/glutamate metabolism (*P* = 0.0339).

**Conclusions:** These results show that tumour evolution requires energy and structural substrates such as glucose and glutamine and promotes intense host spoliation, such as protein degradation leading to an intense muscle mass waste (higher methylhistidine), inducing a severe weight loss, which culminates in host cachectic state.

**5-07**

**The mitochondrial metabolic reprogramming agent trimetazidine as an ‘exercise mimetic’ and myogenesis promotor in cachectic C26‐bearing mice**

Elisabetta Ferraro^1^, Francesca Molinari^1^, Fabrizio Pin^2,3^, Stefania Gorini^1^, Sergio Chiandotto^4^, Fabio Penna^2,3^, Emanuele Rizzuto^5^, Antonio Musaro^6,7^, Paola Costelli^2,3^ and Giuseppe Rosano^1,8^

^1^ *Laboratory of Pathophysiology of Cachexia and Metabolism of Skeletal Muscle, IRCCS San Raffaele Pisana, 00166, Rome, Italy;* ^2^ *Department of Clinical and Biological Sciences, University of Turin, Italy;* ^3^ *Interuniversity Institute of Myology‐IIM;* ^4^ *DMCM Department of Molecular and Clinical Medicine, c/o Department of Surgery "Pietro Valdoni", Sapienza University of Rome, Italy;* ^5^ *Department of Mechanical and Aerospace Engineering, Sapienza University of Rome, 00184, Rome, Italy;* ^6^ *Institute Pasteur Cenci‐Bolognetti, DAHFMO‐Unit of Histology and Medical Embryology, IIM, Sapienza University of Rome, 00161, Rome, Italy;* ^7^ *Center for Life Nano Science@Sapienza, Istituto Italiano di Tecnologia, 00161, Rome, Italy;* ^8^ *Cardiovascular and Cell Sciences Institute, St George's University of London, Cranmer Terrace, London, UK*

**Introduction:** Mitochondrial metabolism reprogramming might enhance metabolic efficiency. We investigated the ability of the metabolic modulator trimetazidine (TMZ), which partially inhibits mitochondrial free fatty acid β‐oxidation while enhancing glucose oxidation, to counteract skeletal muscle dysfunctions and wasting occurring in cancer cachexia.

**Methods:** For this purpose we used mice bearing the C26 coloncarcinoma as a model of cancer cachexia. Mice received 5 mg/kg TMZ (i.p.) once a day for 12 consecutive days. A forelimb grip strength test was performed, and *tibialis anterior* and *gastrocnemius* muscles were excised for analysis. *Ex vivo* measurement of skeletal muscle contractile properties was also performed. Moreover, satellite cells and C2C12 myoblasts were treated with TMZ during differentiation.

**Results:** Our data showed that TMZ induces some effects achieved through exercise, among which is grip strength increase, an enhanced fast‐to‐slow myofiber phenotype shift, reduced glycemia, PGC1α up‐regulation, oxidative metabolism and mitochondrial biogenesis. TMZ also partially restores the myofiber cross‐sectional area in C26‐bearing mice, while modulation of autophagy and apoptosis were excluded as mediators of TMZ effects. Moreover, TMZ enhances the myogenic potential of skeletal muscle progenitor cells leading to MyoD, Myogenin, Desmin over‐expression and, similarly to exercise, stimulates the phosphorylation of AMPK and up‐regulates PGC1α, both of which enhancing mitochondrial biogenesis necessary for myoblast differentiation. Finally, we found that TMZ is able to stimulate myogenesis *in vivo* both in the C26 mice model of cancer cachexia and upon cardiotoxin damage.

**Conclusions:** In conclusion, our data show that TMZ acts like an “exercise mimetic” and is able to promote myogenesis, enhance regenerative capacity and trigger some mechanisms of adaptation to stress in cancer cachexia. This makes the modulation of the metabolism by TMZ a suitable candidate for a therapeutic rehabilitative protocol design and for regeneration‐impaired disorders, particularly considering that TMZ has already been approved for clinical use.

**5-08**

**Cancer and chemotherapy‐induced cachexia yield distinct metabolic perturbations**

Fabrizio Pin^1,2^, Rafael Barreto^3^, Marion E. Couch^4,5,6^, Thomas M. O'Connell^3,4,5,6^ and Andrea Bonetto^1,2,3,4,5,6^

^1^ *Department of Anatomy and Cell Biology, Indiana University School of Medicine, Indianapolis, IN, USA;* ^2^ *Indiana Center for Musculoskeletal Health, Indiana University School of Medicine, Indianapolis, IN, USA;* ^3^ *Department of Surgery, Indiana University School of Medicine, Indianapolis, IN, USA;* ^4^ *Department of Otolaryngology ‐ Head and Neck Surgery, Indiana University School of Medicine, Indianapolis, IN, USA;* ^5^ *Simon Cancer Center, Indiana University School of Medicine, Indianapolis, IN, USA;* ^6^ *IUPUI Center for Cachexia Research Innovation and Therapy, Indiana University School of Medicine, Indianapolis, IN, USA*

Advanced cancer patients frequently suffer from cachexia, a severe wasting condition characterized by depletion of both muscle and adipose tissue. Work from our laboratory and others have shown that chemotherapy, one of the primary treatment options for cancer can also lead to cachexia. In this study, we employed a comprehensive metabolomics approach to elucidate the similarities and differences in the metabolic perturbations of these two drivers of cachexia.

Our study design included four sets of mice: (1) mice implanted with C26 colorectal tumor cells, (2) mice given chemotherapy Folfiri (5‐FU, leucovorin, irinotecan) for five weeks, (3) C26 tumor bearing mice treated with Folfiri, and (4) vehicle treated mice. The multi‐platform metabolomics approach utilized both NMR and mass spectrometry analyses on serum, muscle and liver tissue. The data highlighted several striking differences between the metabolic phenotypes of the C26 tumor and chemotherapy groups. The C26 group displayed reduced levels of TCA cycle intermediates, branched chain amino acids and short chain acylcarnitines while those same metabolites were unchanged in the Folfiri group. The number of low density lipoprotein particles (LDL‐P) increased by more than five‐fold in the C26 group while a more modest two‐fold increase was observed in the Folfiri group, suggesting differences in lipid and cholesterol metabolism. In muscle, the ATP levels were more significantly reduced in the Folfiri group compared with the C26 perhaps, suggesting a more dramatic impact on oxidative metabolism. In the C26 group treated with Folfiri, the effects of each treatment were sometimes additive and sometimes appeared to be dominated by one condition over the other.

Overall, our results show that cancer‐ and chemotherapy‐induced cachexia are characterized by clearly different metabolic perturbations indicating different pathophysiological mechanisms. New therapeutic interventions to treat cachexia will have to account for these differences in order to provide effective treatment.

**5-09**

**Oxidative alterations in sarcoplasmic reticulum and mitochondria are determinants for loss of mass of fast‐twitch fibers in the first stage of cancer‐induced cachexia**

Flávia Alessandra Guarnier^1^, Fernanda Paschoal Blegniski^1^, Claudia Pecorai^2^, Rubens Cecchini^1^, Feliciano Protasi^2^ and Simona Boncompagni^2^

^1^ *Dept of General Pathology, Univ. Estadual de Londrina, Londrina, PR, Brazil;* ^2^ *CeSI‐MeT, Center for Research on Ageing and Translational Medicine, Univ. G. d' Annunzio, Chieti, Italy*

The current literature correlates the proteolysis‐related atrophy (which usually occurs in later stages of cachexia syndrome) to oxidative damage of proteins. Fast‐twitch fibers are more affected during the muscle waste induced by cancer cachexia. In the present study, we investigated oxidative modifications, ultrastructural modifications in mitochondria and sarcoplasmic reticulum (SR), and the correlation with muscle loss in EDL and Soleus muscles (respectively fast‐ and slow‐twitch muscles) in pre‐cachexia. Male Wistar rats were subcutaneously inoculated with a suspension of Walker‐256 tumor cells and divided into 2 groups: tumor‐bearing rats (T), and tumor‐bearing rats treated with N‐acetylcisteine (T‐NAC; NAC 1% *ad libitum* in drinking water), a drug known to promote increased antioxidant capacity in tissues. After 5 days, pre‐cachexia was characterized in T animals according to the criteria pre‐established by Fearon *et al*. (2011; *Lancet Oncol*). Tumor implantation caused decrease in general body weight (−2.77%), followed by a significant weight loss in EDL muscle (−15.33%), contrasting with the small mass loss of soleus (−5.14%). We measured both protein and membrane oxidation and found that only membrane oxidation was significantly increased in EDL from tumor‐bearing mice. Soleus presented a discrete alteration on redox balance. As a result, we found that the volume of intracellular membrane organelles such as SR and mitochondria was significantly decreased (−18.89% and −22.41%, respectively) compared to group C. In addition, in the EDL of tumor‐bearing rats, the fusion:fission ratio was altered in favor of the former. Interestingly, all differences were significantly rescued in T‐NAC group, suggesting a central role of oxidative stress in the modifications of membrane compartments. These data suggests that in pre‐cachectic stages: 1) glycolytic muscles are already more prone to mass waste, but independently of protein oxidation, and 2) loss of membrane organelles could play an important role early in these initial stages.

This study was supported by the following grants:

- Brazilian Ciências Sem Fronteiras: CNPq 233892/2014‐1 and Brazilian Continuous Flow Program to support special projects (973/2013) to FAG;
- MIUR Future in Research: RBFR13A20K to SB.

**5-10**

**Development of a metabolic biomarker panel for the early detection of cancer cachexia**

Rafael Barreto^1^, Fabrizio Pin^2,3^, Marion E. Couch^3,4,5,6^, Andrea Bonetto^1,2,3,4,5,6^ and Thomas M. O'Connell^3,4,5,6^

^1^ *Department of Surgery, Indiana University School of Medicine, Indianapolis, IN, USA;* ^2^ *Department of Anatomy and Cell Biology, Indiana University School of Medicine, Indianapolis, IN, USA;* ^3^ *Indiana Center for Musculoskeletal Health, Indiana University School of Medicine, Indianapolis, IN, USA;* ^4^ *Department of Otolaryngology ‐ Head and Neck Surgery, Indiana University School of Medicine, Indianapolis, IN, USA;* ^5^ *Simon Cancer Center, Indiana University School of Medicine, Indianapolis, IN, USA;* ^6^ *IUPUI Center for Cachexia Research Innovation and Therapy, Indiana University School of Medicine, Indianapolis, IN, USA*

**Introduction:** Currently, there are no mechanism‐based tools for the early detection & diagnosis of cachexia, a debilitating condition that typically progresses through three phases, namely, pre‐cachexia, cachexia and refractory cachexia. Unfortunately, the classically emaciated patient is often in the refractory stage where the decline is irreversible.

**Methods:** In this study we employed a comprehensive, multi‐platform metabolomics including untargeted high field NMR, targeted LC/MS and NMR‐based lipoprotein analysis. Our study design involved implanting mice s.c. with C26 colorectal tumor cells and sampling serum and muscle tissue every other day for two weeks. In this model the mice generally develop significant weight loss starting day 10.

**Results:** The metabolomics analyses of serum revealed that the trajectories of several biomarkers, among which the branched‐chain amino acids (BCAAs), significantly diverged from controls in advance of detectable weight loss. In particular, leucine levels significantly dropped by day 6, well in advance of detectable weight loss and even in advance of a palpable tumor. Interestingly, previous studies have suggested that reductions in BCAAs are correlated with increased muscle amino acid oxidation. Other metabolic markers that diverged at or before day 10 include low density lipoprotein particles, taurine and GlycA (an inflammatory marker associated with acute phase response).

**Conclusions:** These initial biomarkers represent a set of critically altered pathways in the development of cancer cachexia including protein catabolism, lipolysis/dyslipidemia, oxidative stress and inflammation. This work provides the foundation for the development of a biomarker panel that would provide early detection and stratification of cachexia based upon the unique constellation of metabolic disturbances that may afflict individual patients. Further studies will be conducted on different cancer models and eventually in human cohorts.

**5-12**

**Doxorubicin induces dose‐dependent cachexia in mice**

James C. Sorensen^1,2^, Benjamin M. Butcher^1,2^, Cara A. Timpani^1,2^, Alan Hayes^1,2,3^ and Emma Rybalka^1,2,3^

^1^ *College of Health & Biomedicine, Victoria University, Melbourne, Australia;* ^2^ *Australian Institute for Musculoskeletal Science (AIMSS), Melbourne, Australia;* ^3^ *Institute of Sport, Exercise & Active Living (ISEAL), Victoria University, Melbourne, Australia*

**Introduction:** Doxorubicin (DOX) is a widely used anthracycline chemotherapeutic agent,^1^ which has emerged as a significant and independent promoter of cachexia during anticancer therapy.^2^ To characterise its muscle‐specific effects, DOX has typically been administered in cancer‐free animal models as a single high bolus dose or with prolonged periods between dosages.^2^ However, in the clinical setting, metronomic DOX administration is common since it is generally of greater therapeutic benefit.^3^ Thus, the aim of our study was to assess the cachectic signature of metronomic low, moderate and high dose DOX treatment over either one (short) or two (long) week schedules, in cancer‐free mice.

**Methods:** 6‐week old Balb/C mice were treated with DOX using three different schedules: (1) 6 × 2mg/kg dosages over 14 days (low‐long (LL)); (2) 3 × 4mg/kg dosages over 7 days (moderate‐short (MS)); and (3) 6 × 5mg/kg dosages over 14 days (high‐long (HL)). Mice were weighed and body composition analysis was performed using pre‐ and post‐treatment EchoMRI. Thereafter, muscles and organs were harvested, weighed and functional and structural analyses were performed.

**Results:** DOX treatment induced cachexia in a dose‐dependent and anorexia‐independent manner (LL = −14%; MS = −17%; HL = −28%; *p <* 0.0005), and this was mostly attributable to a loss of fat mass (LL = −47%; MS = −39%; HL = −55%; *p* < 0.0005). Lean mass wasting was only observed for the HL schedule (−11%; *p <* 0.05). Regardless of dosage, cachexia was most severe after day 5 of treatment. On the MS schedule, *extensor digitorum longus* (fast‐twitch muscle) mass was reduced by 28% (*p <* 0.05), while *soleus* (slow‐twitch muscle) mass was unaffected.

**Conclusions:** Our data highlight that cumulative dosage is more impactful than treatment duration, but that dose repetition is a factor in cachexia progression. While overall lean mass was unaffected at lower dosage schedules, the loss of fast‐twitch muscle mass (i.e. EDL) was still observable, suggesting fibre‐type‐specific effects which likely account for the muscle weakness reported by patients.

1. Jamieson & Boddy, Expert Opin Drug Metab Toxicol, 2011; 7(10):1201–10.
2. Sorensen *et al*., Cancer Chemother Pharmacol, 2016; 78(4):673–683.
3. Scharovsky *et al*., Current Oncol, 2009;16(2):7.

**5-13**

**The systemic activin response to pancreatic tumors: implications for effective cancer cachexia therapy**

Xiaoling Zhong, Marianne Pons, Christophe Poirier, Yanlin Jiang, Jianguo Liu, George E. Sandusky, Yunlong Liu, Guanglong Jiang, Marion Couch, Leonidas G. Koniaris and Teresa A. Zimmers

*Indiana University School of Medicine, Indianapolis, USA*

**Introduction:** The majority of patients with pancreatic ductal adenocarcinoma (PDAC) develop cachexia. Serum activin correlates with cachexia and mortality in PDAC, and Activin over‐expression causes cachexia. We studied Activin expression in PDAC cachexia.

**Methods:** Data mining, immunohistochemistry, qRT‐PCR, cell‐based studies, and mouse modeling were used.

**Results:** In meta‐analysis of 9 studies of human PDAC, INHBA, encoding Activin A, was among the top 1–10% of over‐expressed. INHBB, encoding Activin B, was not. In the pancreas TCGA, high INHBA conferred increased mortality (HR 1.218, *P* = 0.003), while INHBB was not significant. Immunohistochemistry detected Activins in human PDAC in both tumor and stromal cells. Next, we characterized three novel orthotopic and the genetic LSL‐**K**rasG12D;LSL‐**p**53R172H;Pdx1‐**C**re (KPC) mouse PDAC models. All developed cachexia, with KPC cell lines expressing more INHBA causing more rapid cachexia. (INHBB was not detected in cell lines.) Tumors from KPC lines expressed both INHBA and INHBB, with expression in both tumor and stromal cells. Notably, in both genetic and orthotopic models, Activin expression was not confined to tumors. Immunohistochemistry detected Activins in muscle, heart, kidney, and liver. RT‐qPCR indicated that tumors expressed mostly INHBA, while organs expressed both INHBA and INHBB, with the latter more dynamically regulated. KPC tumors likely induce Activin and wasting directly, because KPC cell line conditioned medium‐induced INHBA expression and atrophy in C2C12 myotubes. The Activin trap ACVR2B/Fc showed reduced efficacy against cachexia in models expressing higher levels of Activin and did not affect activin expression in organs, suggesting that circulating Activin was not responsible for systemic Activin expression.

**Conclusions:** PDAC induces a systemic Activin response. Given the differential transcriptional regulation of INHBA and INHBB and the differing efficacy of ACVR2B/Fc on endocrine versus autocrine Activin signaling, our results suggest that specific targeting and/or upstream targeting of each tumor‐released and tumor‐induced Activin would offer more effective therapy.

**5-14**

**Enhanced β‐myosine heavy chain gene expression in rat heart during adjuvant arthritis—a model of cachectic rheumatoid arthritis**

Jana Jurcovicova^1^, Andrea Stofkova^2^, Viktoria Lory^1^, Katarina Krskova^1^ and Stefan Zorad^1^

^1^ *Institute of Experimental Endocrinology, Biomedical Centre, Slovak Academy of Sciences, Bratislava, Slovakia and Department of Normal, Pathological, and Clinical Physiology, Third Faculty of Medicine, Charles University, Prague, Czech Republic;* ^2^ *Department of Normal, Pathological, and Clinical Physiology, Third Faculty of Medicine, Charles University, Prague, Czech Republic*

Heart failure is a frequent comorbidity of cachectic rheumatoid arthritis in humans (RA). Adults with RA have about twofold higher risk of cardiovascular disease even without enhanced of traditional risk factors. Inflammatory cytokines are supposed to be implicated in this pathogenesis. Central role in cardiac heart failure plays β‐isoform of myosin heavy chain (β**‐**MHC) by reducing cardiomyocyte contractility. The β**‐**MHC production is regulated by Foxo1 transcription factor which is controlled by insulin signaling cascade, and/or by triiodothyronin‐T3/thyroid hormone receptor (TR) complex which negatively regulates β**‐**MHC transcription.

The aim of our study was to find out whether adjuvant arthritis in rats (AA)—a model of human RA affects cardiac β**‐**MHC expression and to elucidate the mechanisms involved.

AA was induced to male Lewis rats by complete Freund's adjuvant. On day 18 of maximum inflammatory score the experiment was terminated. The hearts were perfused in situ with heparinized saline, and the tissues around left ventricles were dissected and extracted for total RNA. After a reversed transcription the relative cDNA for each transcript was measured by real‐time PCR using the SYBER green mix and the primer sequences for rat TNF‐α, IL‐6, β**‐**MHC, insulin receptor substrates IRS1, IRS2, Foxo1, Foxo4, TRβ1, TRβ2. As reference gene the expression for HPRT was used.

The expression of β**‐**MHC in AA‐rats was enhanced against nontreated controls (*p* < 0.01) and against pair fed rats (*p* < 0.05). The cytokine expressions were low at the detection limit. None of the molecules of the insulin signaling pathway, neither of the Foxo expressions were changed. However, in AA group the significant downregulation of TRβ1, TRβ2 was found, indicating desinhibition of β**‐**MHC and thus contributing to its overexpression.

These results show that chronic inflammation during AA may cause cardiac contractility dysfunction by activating of β**‐**MHC expression whereby downregulation of TRs plays a role.

Supported by: grant P34, Czech Republic and grants VEGA 2/0174/17 and APVV‐15‐0229, Slovak Republic.

**6-01**

**Changes in body composition during first cycle of chemotherapy in metastatic non‐small cell lung cancer (NSCLC) are predictive for poor overall survival**

Juliette H.R.J. Degens^1†^, Karin J.C. Sanders^1^, Evelyn E.C. de Jong^3^, Annemie M.W.J. Schols^1^ and Anne‐Marie C. Dingemans^2^

^1^ *Department of Respiratory Medicine, NUTRIM School of Nutrition and Translational Research in Metabolism, Maastricht University Medical Center, Maastricht, The Netherlands;* ^2^ *Department of Respiratory Medicine, GROW School for Oncology and Developmental Biology, Maastricht University Medical Center, Maastricht, The Netherlands;* ^3^ *Department of Radiation Oncology (MAASTRO Clinic), GROW School for Oncology and Developmental Biology, Maastricht University Medical Center, Maastricht, The Netherlands*

^†^Authors contributed equally

**Background:** Sarcopenia adversely affects prognosis in metastatic NSCLC. However, the pattern of changes and distribution of muscle‐ and adipose tissue mass during chemotherapy and their relation to overall survival (OS) is unclear. Therefore, we analyzed changes in muscle cross‐sectional area (CSA), intramuscular adipose tissue (IMAT), subcutaneous adipose tissue (SAT) and visceral adipose tissue (VAT) on CT‐images after the first cycle of chemotherapy.

**Methods:** In this post‐hoc analysis of the randomized controlled NVALT12 trial (NCT01171170), body composition was characterized in 117 patients at the third lumbar level on CT‐images obtained before start of treatment and after 1 cycle of chemotherapy. Changes in body composition were related to OS with Kaplan Meier and log‐rank test. Cox multivariate analysis assessed the relative contribution of muscle and adipose tissue CSA and distribution to OS.

**Results:** Changes in muscle‐ and IMAT SCA were an independent predictor for OS while change in SAT and VAT were not. Three patterns could be distinguished based on body composition changes; muscle stable or gain (*n* = 36, type 1), muscle loss and IMAT stable or gain (*n* = 47, type 2), muscle‐ and IMAT loss (*n* = 34, type 3). Body composition types were comparable for gender, age, WHO‐PS, TNM‐status and comorbidity index. Type 1 was most predictive for poor OS with a median OS (95% CI) of 8.9 months (5.4 to 12.3) compared to 10.7 months (6.5 to 14.9) in type 2 (HR 2.1 95% CI (1.3–3.4) *p* = 0.003). There was no significant difference in OS between type 2 and type 3.

**Conclusions:** In contrast to previous research in non‐pulmonary malignancies, not only decrease of muscle mass but also IMAT changes are predictive for poor OS in metastatic NSCLC after one cycle of treatment. Early recognition of body composition changes could be useful for more personalised supportive intervention during follow‐up treatment.

**6-02**

**Occult deficits in baseline cardiac function in NSCLC patients eligible for carboplatin‐ based therapy: implications for anti‐cachexia clinical trials**

Seyyed Mohammad Reza Kazemi‐Bajestani^1^, Harald Becher^2^, Charles Butts^3^, Naveen S. Basappa^3^, Michael Smylie^3^, Anil Abraham Joy^3^, Randeep Sangha^3^, Andrea Gallivan^1^, Quincy Chu^3^ and Vickie Baracos^1^

^1^ *Department of Oncology, Division of Palliative Care Medicine, Cross Cancer Institute, University of Alberta, Edmonton, Canada;* ^2^ *Department of Medicine, Division of Cardiology, Alberta Cardiovascular and Stroke Research Centre, Cross Cancer Institute, University of Alberta, Edmonton, Canada;* ^3^ *Department of Oncology, Division of Medical Oncology, Cross Cancer Institute, University of Alberta, Edmonton, Canada*

**Introduction:** According to European and American regulatory agencies, physical function (e.g. stair climbing test) would be an approvable (co)primary endpoint of new anti‐cachexia medications; however, it is unknown whether cardiac function is adequate for physical function tests. We aimed to explore the cardiac status of a group of patients conforming to the criteria for inclusion in cachexia clinical trials (i.e., POWER, ROMANA, and MENAC).

**Methods:** Seventy patients with metastatic non‐small cell lung carcinoma [51.4% male; 96% ECOG 0–1; eligible for carboplatin‐based therapy] were evaluated before start of 1st line chemotherapy. All of the patients were evaluated by echocardiography, FACIT fatigue and MRC dyspnea scales. Computed tomography cross‐sectional images were utilized for body composition analysis.

**Results:** In 9 patients (12.8%), echocardiography revealed previously undiagnosed, clinically relevant cardiac abnormalities. Seven patients had left ventricular ejection fraction (LVEF) 32%–47%; 1 patient had severe right ventricular dilation and pulmonary hypertension, and 1 patient with severe pericardial effusion warranted hospitalization and drainage. Of these 9 patients, only one patient had a recent history of heart failure. Ten (14.3%) additional patients had diastolic dysfunction with preserved LVEF. These cardiac conditions were associated with worse fatigue (*p* < 0.05), dyspnea (p < 0.05) and anemia (*p* = 0.06). Five out of 7 patients with LVEF < 50% were sarcopenic.

**Conclusions:** A detailed evaluation of the cardiac function in our clinical trial—eligible population reveals a heterogeneous cardiac profile and symptom burden that may significantly impact on their ability to perform physical activities (such as stair climbing), which serve as anti‐cachexia trial endpoints. Cardiac‐related inclusion and exclusion criteria of aforementioned trials would fail to identify these patients. Detailed echocardiography might need to be included in the screening procedure of lung cancer patients for clinical trials with anti‐cachexia therapeutics especially those using physical activity as primary endpoints.

**6-03**

**Staging of nutrition disorders in 531 non‐small cell lung cancer (NSCLC) patients: benefit from skeletal muscle mass, anorexia and performance status assessments**

Hugues Morel^1^, Didier Debieuvre^2^, Pierre‐Jean Souquet^3^, Veerle Surmont^4^, David Planchard^5^, Franck Bonnetain^6^, Ivan Krakowski^7^, Hélène Gaudin^8^ and Sami Antoun^9^

^1^ *C.H.R. Orléans ‐ La Source, Department of Pneumology, 45067, Orléans, France;* ^2^ *GHRMSA, Hôpital Emile Muller, Department of Pneumology, Mulhouse, France;* ^3^ *Centre Hospitalier Lyon Sud, Department of Pneumology, Pierre Bénite, France;* ^4^ *Gent University Hospital, Department of Thoracic Oncology, Gent, Belgium;* ^5^ *Institut Gustave Roussy, Department of medical oncology, Villejuif, France;* ^6^ *CHRU Besançon Jean Minjoz, Methodological and quality of life unit in oncology, Besançon, France;* ^7^ *Institut Bergonié, Département Interdisciplinaire de Soins de Support pour le Patient en Oncologie (DISSPO‐CARE), Bordeaux, France;* ^8^ *Chugai Pharma France, Paris, France;* ^9^ *Institut Gustave Roussy, Unité de médecine aigue en oncologie, Villejuif, France*

**Introduction:** An international consensus proposed in 2011 a staging of cancer cachexia (CAX), mainly based on weight loss (WL), skeletal muscle loss (SML), inflammation and anorexia (Fearon). While CAX definition relies on well‐defined criteria, those of pre‐CAX and refractory CAX remain imprecise. We initiated this study to refine CAX stages with quantifiable parameters.

**Methods:** This is a cross‐sectional, prospective, multicentric study conducted in patients with NSCLC. Skeletal muscle mass (SMM) was assessed by analysing L3 CT‐scan images. Patients completed EORTC QLQ‐C30 health‐related quality of life (QoL) questionnaire. For pre‐CAX stage definition we recorded anorexia based on patient answer to QoL question on appetite loss. We defined refractory CAX based on ECOG performance status and WL using the thresholds values associated with low survival (<5.4 months) described by Martin. Endpoints were the frequency and the characteristics of CAX stages.

**Results:** 531 patients recruited in 2016 by 56 sites within 3 months were analysed. 312 had SMM assessment. Median age was 66 years, 79.9% were PS < 2, and the tumor stage was mainly IIIB‐IV (87.3%). 33.8% of patients had pre CAX, 38.7% CAX, and 0.9% refractory CAX. CAX stage progression was associated with lower functional scale score (except cognitive) of the QoL questionnaire (*p* < 0.0001). SMM was the only relevant criteria found in 8.1% of cachectic patients without WL > 5% and in 43.8% of pre‐CAX patients with WL < 2%. Notably, 48.4% of all the patients and 25.8% of pre‐CAX patients had SML with no anorexia (questioning SML mechanisms not secondary to decreased intake).

**Conclusions:** This is the first study defining cancer cachectic stages with quantifiable parameters for pre‐CAX and refractory CAX stages. Stage definitions were supported by associated functional QoL scores. While measuring SMM is of poor interest when WL is >5%, it could however be useful for detecting early nutritional disorders.

**6-04**

**A longitudinal study of muscle strength and function in patients with cancer cachexia**

Nichola Gale^1^, David Wasley^2^, Sioned Roberts^3^, Karianne‐ Backx^2^, Annemarie Nelson^4^, Robert van Deursen^1^ and Anthony Byrne^4^

^1^ *Cardiff University, School of Healthcare Sciences Cardiff, UK;* ^2^ *Cardiff Metropolitan University, Cardiff School of Sport, UK;* ^3^ *Velindre Cancer Centre, Cardiff, UK;* ^4^ *Marie Curie Palliative Care Research Centre (MCPCRC), Cardiff University, Heath Park, Cardiff, UK*

**Introduction:** Patients with advancing cancer frequently suffer a reduction in daily activities with profound effects on function and quality of life (Laviano *et al*. 2005). However, the rate of decline has not been well described in those with established cancer cachexia (CACS). The aim of this study was to assess change in muscle strength and physical function over 8 weeks in this patient group.

**Methods:** Patients with lung and gastrointestinal cancer, with unintentional weight loss of >5% in 6 months or BMI <20 plus 2% weight loss were included. Physical assessments (baseline, 4 weeks, 8 weeks) included: isometric quadriceps and hamstring strength, handgrip, standing balance, 10 m walk time and timed up and go. This was part of a larger study of activity, motivation and exercise preferences in established CACS.

**Results:** Fifty patients (32 male), mean ± SD age 65 ± 10 years and BMI 24.9 ± 4.3 kg/m^2^ were recruited. The majority of patients (*n* = 43) had a BMI over 20 kg/m^2^ with 16% of lung patients and 12% of GI patients being underweight (BMI < 19.9 Kg/m^2^). Lung cancer patients had lower muscle strength and function than those with GI cancer (p < 0.05). Despite notable attrition, in the 19 (8 lung and 12 GI) patients who completed all assessments, there was little change in muscle strength and performance over 8 weeks (*p* > 0.05). Baseline variables did not differ significantly between completers and non‐completers (*p* > 0.05).

**Conclusions:** More than a third of patients with established cancer cachexia in our study were stable over 8 weeks, suggesting a subgroup that may have potential to benefit from targeted interventions. Better understanding the physical performance parameters which characterize and differentiate these patients has important clinical implications for cancer multidisciplinary team practice.

**6-05**

**Proteomic profiling of the tumour microenvironment of cachectic patients: the search for biomarkers**

Joanna Darck Carola Correia Lima^1^, Fernanda Janku Cabral^2^, Estefanía Simões Fernández^1^, Emídio Marques de Matos‐Neto^3^, Rosangela Santos‐Eichler^1^, José Otoch Pinhata^1^, Paulo Sérgio Alcantara Martins^1^, Leo Kei Iwai^4^, Emer Ferro Suavinho^1^ and Marília Cerqueira Leite Seelaender^1^

^1^ *University of São Paulo, Brazil;* ^2^ *University of Campinas;* ^3^ *University of Piauí;* ^4^ *Instituto Butantan*

**Background:** Cachexia is a multiorgan syndrome characterised by progressive and involuntary weight loss and major effects on skeletal muscle. The pathogenicity and origin of this syndrome is multifactorial and strongly associated with systemic inflammation due to a complex interaction of reduced food intake and changes imposed on metabolism by tumour and host factors. To understand the isolated effects of the tumour microenvironment, the aim of the present study was to identify changes in the proteome expression of tumour tissue of cachectic patients and non‐cachectic patients and to investigate possible biological functions related to these proteins.

**Methods:** Patients with colorectal cancer were recruited after signature of the informed consent form and tumour biopsies were collected in surgery. Patients were divided into Weight‐Stable Cancer (WSC *n* = 2) and Cachectic Cancer (CC n = 2) groups. Protein was extracted, *samples incubated with trypsin and* peptides were isotopically marked with light (D0‐TMAB) or heavy (D3‐TMAB). Samples were analyzed by mass spectrometry (LC‐MS/MS), and both quantitation (H/L ratios) and protein/peptide searches in NCBI databank were done using mascot algorithm. Statistics, proteome validation and heatmaps for protein expression visualisation were performed with Perseus. Gene Ontology (GO) analysis was carried out with Furinch.

**Results/Conclusions:** In total, 398 proteins were identified and quantitated. From these, 105 proteins were statistically significantly different between the groups (p value and false Discovery rate (FDR) <0.05). Careful analysis of H/L ratio expression on heatmap was carried out for selection of the proteins that presented expression profile modulation. 22 proteins were found to be up or down‐regulated by cachexia. GO of these modulated proteins showed that their majority consisted of proteins related with energetic and protein metabolism, muscle development and contraction, modulation of immune response and proliferation, growth and cellular maintenance. In conclusion, tumours if cachectic patients show a different pattern of protein expression, implying that one such pattern could be related with the induction of cachexia. Furthermore, the results provide insight on the adoption of tumour sample analysis for precocious diagnosis of the syndrome.

**6-06**

**Fatty acid profile alterations in plasma and subcutaneous adipose tissue of cachectic cancer patients**

Katrin Radloff^1,3^, Daniela Mendes dos Reis Riccardi^1^, Raquel Galvao Figuêredo^1^, Joanna Darck Carola Correia Lima^1^, Joyce de Cassia Rosa Jesus‐Lima^1^, Ariene Murari^1^, José Pinhata Otoch^2^, Gerhard Paul Püschel^3^, Alison Colquhoun^1^ and Marília Seelaender^1^

^1^ *Institute of Biomedical Science, Cancer Metabolism Research Group, University of Sao Paulo, Brazil;* ^2^ *Department of Clinical Surgery, University of Sao Paulo, Brazil;* ^3^ *Institute of Nutritional Science, Department of Biochemistry of Nutrition, University of Potsdam, Germany*

**Background:** Systemic inflammation and loss of fat mass are hallmarks of cancer cachexia. Fatty acids (FA) are involved in inflammatory pathways and the adipose tissue as storage of bulk FA might contribute to promote inflammation.

**Aim:** To assess the FA profile of plasma and adipose tissue and to measure the gene expression of FA modifying enzymes in cancer cachexia.

**Methods:** Volunteers were recruited after signing the informed consent form at the University Hospital. Patients subjected to incisional hernia surgery were included in the control group (Control), while gastrointestinal carcinoma patients were divided into weight‐stable cancer (WSC) group and cachectic cancer group (CC). Cachexia was diagnosed as in Fearon *et al*., 2011. FA were separated and quantified using GC/MS. Gene expression of FA modifying enzymes of subcutaneous adipose tissue (SAT) were measured by real‐time PCR.

**Results:** In plasma there was a shift from unsaturated to saturated FA. The percentage of palmitic acid was higher in WSC and CC compared to Control (*p* = 0.006) and stearic acid content was higher in CC (*p* = 0.025). SAT exhibited slide changes in the bulk fatty acid species. Myristic acid 14:0 was diminished in plasma (*p* = 0.019) as well as in SAT (*p* = 0.06). In plasma, lower contents of different types of n3 and n6‐FA were measured in both cancer groups compared to controls or in CC compared to WSC. In SAT only 20:2n6 was significantly changed in CC compared to the other groups (*p* = 0.04). SAT gene expression of FADS1 and ELOVL5 that modify long chain FA was not altered while stearoyl‐CoA desaturase expression tended to reduce in cachectic patients (*p* = 0.08).

**Conclusions:** Changes in plasma FA profile were different from the changes we observed in SAT. The enzyme gene expression alone cannot explain the FA alterations; hence, further studies are needed to understand the FA profile in cancer cachexia.

**6-07**

**A randomized feasibility study evaluating an exercise intervention for patients with advanced GI malignancies and cancer cachexia: trial in progress**

Richard F. Dunne, Supriya Mohile, Jennifer Peckham, Charles Heckler, Javier Bautista, Michelle Janelsins, Michelle Porto, Po‐Ju Lin, Aram Hezel and Karen M. Mustian

*University of Rochester Medical Center, Rochester, 14620, USA*

**Introduction:** Cancer cachexia (CC) is characterized by progressive loss of weight and lean muscle mass that contributes to impairments in physical performance and a poorer prognosis. Although common in gastrointestinal tract (GI) cancer patients, there are currently no FDA‐approved treatments for CC. Exercise for Cancer Patients (EXCAP©®) is a home‐based, low‐to‐moderate intensity exercise intervention consisting of resistance‐band training and a tailored walking prescription. We are currently conducting a randomized controlled feasibility trial investigating EXCAP©® to improve physical performance and function in patients with advanced GI malignancies and CC.

**Methods:** Incurable GI cancer patients with >5% weight loss or >2% weight loss with signs of inflammation (glucose intolerance, anemia, or hypoalbuminemia) are randomized 1:1 to usual care with chemotherapy (UC) or a 12‐week EXCAP©® regimen beginning on day 1 of chemotherapy (EXCAP). Subjects undergo physical activity tracking and fitness testing at baseline, mid‐intervention and post‐intervention. Cachexia outcome measures include changes in weight and lean mass via bioelectrical impedance and computed tomography (CT), cardiorespiratory and muscle fitness (Short Physical Performance Battery, VO2 max, hand‐grip dynamometry, and muscle activation by EMG), and inflammation and protein synthesis (C‐reactive protein, serum cytokines and branched‐chain amino acids). Since September of 2016, 14 of a planned 40 subjects have enrolled.

**Results:** In total, 74% (14 of 19) of patients offered the study consented to participate. The median age of subjects is 67.4 years. The EXCAP group increased their daily steps on average by 1400 steps, compared to UC whose average activity declined by 1200 steps. So far, 100% of subjects enjoyed participating “very much.” No study‐related adverse events have occurred to date.

**Conclusions:** Preliminarily, EXCAP©® is feasible, well‐liked and safe in patients with CC. The trial is currently enrolling; additional subjects are needed to determine whether exercise can impact physical performance and muscle mass in CC.

**6-08**

**Using body composition from expedient image analysis to predict energy needs in cancer**

Sarah A. Purcell^1^, Vickie E. Baracos^2^, Jessica R. Lieffers^3^, Sunita Ghosh^2^, Marina Mourtzakis^4^ and Carla M. Prado^1^

^1^ *Department of Agricultural, Food, and Nutritional Science, Faculty of Agricultural, Life, and Environmental Sciences, University of Alberta;* ^2^ *Department of Oncology, University of Alberta;* ^3^ *College of Pharmacy and Nutrition, University of Saskatchewan;* ^4^ *Department of Kinesiology, Faculty of Applied Health Sciences, University of Waterloo*

**Introduction:** Resting energy expenditure (REE) is variable in cancer and influences energy needs. We assessed the agreement between measured REE (mREE) and predicted REE (pREE) using anthropometric/demographic variables and skeletal muscle and total adipose cross‐sectional area (CSA, cm^2^).

**Methods:** Patients with advanced colorectal cancer were recruited from the Cross Cancer Institute (Edmonton, Canada). REE was measured by indirect calorimetry. CSAs were quantified via CT images at the 3rd lumbar vertebrae using Slice‐O‐Matic, V4.3. Multiple linear regression identified the contribution of age, sex, height, weight, skeletal muscle and total adipose tissue CSAs to REE and generated equations using significant (*p* < 0.05) variables. Accuracy of new equations was compared to the Harris–Benedict equation. mREE was compared to predicted using Lin's concordance and Bland–Altman analyses.

**Results:** 24 patients were included (BMI 26.1 ± 5.3 kg/m^2^; age 59 ± 13y); mean mREE was 1502 ± 306 kcal/day. Average pREE from the Harris–Benedict equation was 1542 ± 312 kcal/day; bias = 41 kcal/day; limits of agreement = −212 to 293 kcal/day. Linear regression Model 1 included age, sex, height, and body weight; age (*p* < 0.001), height (*p* = 0.001), and weight (p < 0.001) were predictors of REE (p < 0.001 *R^2^* = 0.870, SEE = 118 kcal/day). Mean pREE from this model was 1502 ± 285 kcal/day; bias = 0 kcal/day; limits of agreement = 216 to 216 kcal/day. In Model 2, skeletal muscle and total adipose tissue CSA were added. Skeletal muscle (p = 0.006), age (p < 0.001), weight (*p* = 0.009), and height (*p* = 0.014) were predictors of REE (p < 0.001, *R^2^* = 0.914, SEE = 99 kcal/day). Mean pREE was 1505 ± 290 kcal/day; bias = 3 kcal/day; limits of agreement = −186 to 173 kcal/day. Lin's concordance coefficient was highest for Model 2 equation with a “substantial” strength of agreement (ρ_c_ = 0.958). Model 1 (ρ_c_ = 0.9304) and Harris–Benedict (ρ_c_ = 0.905) equations had “moderate” agreement.

**Conclusions:** Skeletal muscle is a better predictor of REE on an individual and group level compared to anthropometric/demographic data.

**6-09**

**Increased ectopic fat in skeletal muscle and liver is associated with shorter survival in patients with colorectal liver metastases**

David P.J. van Dijk^1,2^, Junfang Zhao^1,2^, Katrin Kemter^1,2^, Vickie E. Baracos^3^, Cornelis H.C. Dejong^1,2,4,5^, Sander S. Rensen^1,2^ and Steven W.M. Olde Damink^1,2,4,5^

^1^ *Department of Surgery, Maastricht University Medical Centre, Maastricht, The Netherlands;* ^2^ *NUTRIM School of Nutrition and Translational Research in Metabolism, Maastricht University, The Netherlands;* ^3^ *Department of Oncology, University of Alberta, Edmonton, Canada;* ^4^ *GROW School for Oncology and Developmental Biology, Maastricht University, Maastricht, The Netherlands;* ^5^ *Department of General, Visceral and Transplantation Surgery, RWTH University Hospital Aachen, Aachen, Germany*

**Background:** Adipose tissue and skeletal muscle mass have been studied extensively in patients with cancer (cachexia). While increased adipose tissue is associated with longer survival, it has been suggested that increased ectopic fat in the muscle and liver is related to poor survival. Using multiple computed tomography scans (CT‐scans) over time and liver biopsies, we assessed the relationship between survival and liver steatosis as well as preoperative changes in muscle fat content and body composition in patients with colorectal liver metastases.

**Methods:** Patients with two available preoperative CT scans were selected from a cohort of 289 consecutive patients with colorectal liver metastases undergoing partial hepatectomy. Scans were analysed using a single CT slice at the L3 level to assess adipose tissue mass and skeletal muscle mass. Muscle fat content was assessed by calculating the average Hounsfield units of the muscle tissue at L3 level (radiation attenuation). Liver biopsies were histologically scored for steatosis using the SAF score.

**Results:** One hundred and thirty‐seven patients had two available preoperative CT scans with a mean time interval of 3.2 months. In multivariate cox‐regression analysis, reduction in muscle radiation attenuation, reflecting fat accumulation, was associated with shorter disease‐free survival (HR 1.98, 95%‐CI: 1.20–3.28; *p* < 0.01) and shorter overall survival (HR 1.79, 95%‐CI: 1.12–2.86; p = 0.01). Liver steatosis was also associated with shorter overall survival (HR 1.77, 95%‐CI: 1.07–2.90, *p* = 0.03). In contrast, high baseline total adipose tissue mass was related to increased disease‐free survival (HR 0.60, 95%‐CI: 0.45–0.80; *p* < 0.01) and overall survival (HR 0.75, 95%‐CI: 0.58–0.98, *p* = 0.03). Changes in skeletal muscle mass were not significantly associated with survival.

**Conclusions:** Ectopic fat accumulation in muscle and liver was associated with shorter disease‐free survival and overall survival, while high total adipose tissue mass seemed to be protective. This indicates that ectopic fat may be an important marker of tumour progression.

**6-10**

**Conditioned medium from cachectic pancreatic tumor organoids inhibits the contractile smooth muscle cell phenotype**

Rianne D.W. Vaes, David P.J. van Dijk, Steven W.M. Olde Damink and Sander S. Rensen

*Department of Surgery and NUTRIM School of Nutrition and Translational Research in Metabolism, Maastricht University, Maastricht, The Netherlands*

**Introduction:** Pancreatic cancer patients often suffer from gastrointestinal‐related symptoms which may be the consequence of underlying gastrointestinal motility problems (e.g. gastroparesis). Although muscle loss in cachectic pancreatic cancer patients is most obvious in skeletal muscle, these clinical symptoms suggest that cachexia manifest itself also in smooth muscle, a tissue responsible for the contractile functionality of the gastrointestinal tract. We therefore aimed to investigate whether factors secreted by pancreatic tumor cells from cachectic or non‐cachectic individuals affect the smooth muscle cell (SMC) contractile phenotype.

**Methods:** 3D pancreatic tumor organoids were established from pancreatic tumor tissue, obtained from pancreatic cancer patients (*n* = 3) of whom the degree of cachexia was thoroughly assessed (weight loss, systemic inflammation, handgrip strength, etc.). Simultaneously, human visceral SMCs were grown to confluency on basement membrane matrix coated surfaces and kept for up to 6 days in the presence of 2% serum allowing SMCs to acquire a contractile phenotype. After 6 days, SMCs were exposed to 25% organoid conditioned medium (CM) for 24 h. mRNA expression of specific contractile SMC markers (*Acta2*, *Actg2*, *Tagln*, *Smtn*) and extracellular matrix components (*Col1a1*, *Col3a1*, *Eln*) was analyzed by RT‐qPCR.

**Results:** CM from cachectic pancreatic tumor organoids provoked decreased expression of *Acta2* (α‐smooth muscle actin; 1.20‐fold) and *Actg2* (γ‐smooth muscle actin; 1.92‐fold)*,* indicating loss of the mature contractile phenotype. Concurrently, we observed increased expression of elastin (*Eln*; 3.4‐fold) supporting a shift from the contractile towards the so‐called synthetic phenotype. Organoids established from non‐cachectic patients (*n* = 2) did not significantly affect expression of these contractile and synthetic markers. Experiments with organoid CM from 8 additional patients are ongoing, as are proteomics analyses to identify potential organoid‐secreted factors involved.

**Conclusions:** These pilot data suggest that pancreatic tumor cells from cachectic patients secrete factors that diminish the contractile SMC phenotype, potentially contributing to gastrointestinal motility problems.

**6-14**

**Role of Electrogastrography (EGG) and effect of ginger (Zingiber Officinale Roscoe) on gastrointestinal symptoms and inflammatory markers in patients with cancer cachexia**

Ravi Bhargava and Martin Chasen

*Bruyère Continuing Care ‐ Bruyère Research Institute, Ottawa, Canada*

**Introduction:** Electrogastrography (EGG) is a non‐invasive method for the measurement of gastric myoelectrical activity (GMA). The aromatic, spasmolytic and carminative properties of ginger suggest it has direct effects on the GMA. Cancer cachexia is associated with a variety of gastrointestinal symptoms such as nausea and vomiting. There are no data on pattern of GMA in response to Ginger in patients with cancer cachexia. Objective of this study is to determine (1) role of electrogastrography in patients with cancer cachexia, (2) effect of oral Ginger administration on GMA in patients with cachexia, and (3) to evaluate GI symptoms using Edmonton symptom assessment scale (ESAS), patient‐generated subjective global assessment form (PGSGA), dyspepsia symptom severity index (DSSI) and correlate levels of inflammatory markers and Ghrelin.

**Methods:** Patients with Cancer cachexia were recruited to document a baseline and post water load EGG after oral ingestion of Ginger capsule (1650 mg) once daily, for 14 days. DSSI, ESAS and PGSGA were completed, and blood test to measure Ghrelin and inflammatory markers was done pre and post intervention.

**Results:** Fifteen participants (8M; 7F) with a median age of 58 and varying cancer diagnoses were enrolled. EGG diagnosis showed that 9 of the 15 patients had a direct improvement in their GMA. All enrolled subjects showed statistically significant improvement in nausea, dysmotility‐like symptoms and reflux‐like symptoms. There was no correlation found for ginger administration and inflammatory factors.

**Conclusions:** Electrogastrography has shown that Gastric myoelectrical activity may get impaired in patients with cancer cachexia. By regulating gastric motility, ginger may improve a range of GI symptoms that can affect oral intake and quality of life.

**6-15**

**Cachexia in lymphoma patients: preliminary results of prevalence and risk factors**

Joris Mallard^1^, Cédric Baudinet^1,2,3^, Manuel Gaviria^1^, Aline Herbinet^1^, Ghislain Quai^1^, Tarik Kanouni^2^, Pierre Louis Bernard^3^ and Guillaume Cartron^2^

^1^ *V@Si SAS, R&D unit, University of Montpellier, 93 Plan de la Prairie, 34270 Saint‐Mathieu‐de‐Tréviers, France, vas‐i.fr;* ^2^ *Montpellier University Hospital, Clinical Hematology clinic, 80 Avenue Augustin Fliche34090, Montpellier, France;* ^3^ *Euromov, University of Montpellier, 700 Avenue du Pic Saint‐Loup 34090, Montpellier, France*

**Introduction:** Cachexia is a major cancer comorbidity defined as a multifactorial syndrome characterised by an ongoing loss of skeletal muscle mass and high mortality. Inflammation with C‐Reactive Protein (CRP) level increase is one of the cachexia mechanisms. Age, gender and Body Mass Index (BMI) represent risk factors of skeletal muscle loss. Lymphoma is a hematological cancer and includes non‐Hodgkin and Hodgkin lymphoma. No recent studies substantiate cachexia prevalence regardless of lymphoma type or analyze risk factors. This retrospective study aims to describe cachexia prevalence in non‐Hodgkin and Hodgkin lymphomas and to analyze association of cachexia with inflammation, age, gender and BMI.

**Methods:** Weight loss was calculated from start of chemotherapy (M0) to 6 months later (M6). Cachexia was diagnosed if weight loss was greater than 5% of total body weight over past 6 months (Fearon's definition, 2011). Correlation between weight loss and inflammation at M6 was investigated using CRP. Risk factors analyzed were age, gender, and BMI.

**Results:** 100 patients were included comprising 59 men and 41 women (mean age 62). The cachexia prevalence was 39%, in both men and women. CRP levels were significantly higher in patients with cachexia (*p* < 0.01). At M6, mean CRP levels of cachectic patients were significantly higher than the 5 mg/L threshold of inflammation in Evan's definition (*p* < 0.01). There was a weak positive correlation between weight loss and respectively CRP level at M6 (*r* ^2^ = 0.42), age (*r* ^2^ = 0.12) and BMI (*r* ^2^ = 0.04).

**Conclusions:** Cachexia prevalence was 39% in lymphoma patients. Inflammation, age, gender and BMI do not seem to be relevant risk factors. Since cachexia prevention concerns all lymphoma patients, exercise could represent one way to prevent it since diagnosis, by increasing protein synthesis and muscle hypertrophy. A study assessing effect of exercise on cachexia prevalence in lymphoma patients will follow these preliminary results.

**6-16**

**Timed up and go test better explains high‐density muscle mass in gynecological cancer patients**

Regielly Candido Silva^1^, Gabriella Villaça Chaves^2^, Anke Bergmann^1^ and Fernando Tadeu Trevisan Frajacomo^1^

^1^ *Program of Molecular Carcinogenesis, Brazilian National Cancer Institute, Rio de Janeiro, Brazil;* ^2^ *Department of Nutrition and Dietetics, Brazilian National Cancer Institute, Rio de Janeiro, Brazil*

**Background and aims:** Current studies recognize muscle quality, means high‐radiodensity skeletal muscle index (HRSMI), as a powerful and independent variable to predict prognosis in cancer survivors. However, it is not clear if muscle quality attenuation on computed tomography (CT) imaging may overcome into lower muscle function. This cross‐sectional observational study investigated whether HRSMI is associated with muscle function in gynecological clinical oncology.

**Methods:** Patients with gynecological cancer prior to treatment were invited to participate. CT images at the third lumbar vertebra (L3) were used to assess overall skeletal muscle index (SMI), calculated in the range −29 to +150 Hounsfield units (HU). The skeletal muscle attenuation in the range +30 to +150 HU was denominated HRSMI. To assess muscle function, we included handgrip dynamometer (HGS/kg), arm curl/30 sec, sit‐to‐stand/30 sec, sit‐and‐reach/cm and Timed‐up and go (TUG, sec) test.

**Results:** Of 73 patients included, mean ages 51.22 (SD ± 14.29), 60.3% had cervical cancer diagnosis. After controlling for BMI, age and cancer stages, we found no evidence of association between SMI and muscle functional impairment. However, considering HRSMI, it was associated with higher values of HGS (*p =* 0.003), arm curl (*p =* 0.002), sit‐to‐stand (*p =* 0.038), and TUG (*p* < 0.001). Specifically, adjusted linear regression model retained TUG test as the most sensitive to decode HRSMI in gynecological cancer (β = −1.45, *p* = 0.003, *R* ^2^ = 38%). Further, ROC analysis indicated an optimal cut‐point value of 6.48 sec to TUG (77% sensitivity and 75% specificity) discriminating those individuals in the 3rd quartile of HRSMI.

**Conclusions:** Our study reinforced relevance of muscle quality, rather than muscle quantity, in the oncology setting. In fact, solely, HRSMI was associated with higher functional capacity. Further, our findings suggest TUG test is eligible as candidate biomarker to elucidate changes in muscle quality in gynecological cancer patients.

**7-01**

**Disease‐related malnutrition factors in patients with ulcerative colitis**

Sergei Ivanov^1^, Igor Khoroshilov^2^, Yury Uspenskiy^1,3^ and Yulia Fominikh^1^

^1^ *Pavlov First Saint Petersburg State Medical University, St. Petersburg, Russian Federation;* ^2^ *North‐Western State Medical University named after I.I. Mechnikov, St. Petersburg, Russian Federation;* ^3^ *Saint‐Petersburg State Pediatric Medical University, St. Petersburg, Russian Federation*

**Background:** Underweight is a frequent complication of ulcerative colitis (UC), but it is not always clear what effect disease‐related malnutrition itself. Our objective is to assess relationship between the disease‐associated factors and the undernutrition occurrence in UC patients.

**Methods:** Cross‐sectional retrospective analysis included data of 103 patients with UC. Demographic characteristics (sex and age), disease behavior (relapse, remission), the extent of colonic involvement (proctitis/left‐sided colitis or pancolitis), the total number of relapses since the onset of the disease were analyzed. Underweight was taken into account when the patient's body mass index was less than 18.5 kg/m^2^, according to WHO criteria. A binary logistic regression was performed to assess the relationship between disease‐related factors and the undernutrition occurrence, taking into account demographic characteristics. The adjusted model included the patient's age, disease behavior, the extent of colonic involvement and the total count of relapses.

**Results:** The general prevalence of underweight among patients with UC was 16.5%. Pancolitis and female sex were associated with a higher incidence of underweight in the adjusted binary logistic model: OR 7.0 (95% CI: 1.3–38.5) and 5.7 (95% CI: 1.3–24.9), respectively. However, there was no reliable relationship between others disease‐related factors and the incidence of undernutrition in patients with UC.

**Conclusions:** The results of this study may be of great importance for screening and preventing of the undernutrition in UC patients with pancolitis.

**7-02**

**High protein, leucine and fish oil‐supplemented nutrition attenuates right ventricular fibrosis and skeletal muscle atrophy in mice with cardiac cachexia**

Paulien Vinke^1,2^, T. Scott Bowen, Renger F. Witkamp^2^ and Volker Adams^1†^ and Klaske van Norren^2†^

^1^ *Department of Internal Medicine and Cardiology, Leipzig University—Heart Center, Leipzig, Germany;* ^2^ *Nutrition and Pharmacology Group, Division of Human Nutrition, Wageningen University, Wageningen, The Netherlands*

^†^Contributed equally

**Introduction:** Cardiac cachexia is characterized by ventricular remodeling that is associated with skeletal muscle wasting. However, therapeutic strategies that target both cardiac and skeletal muscle in order to prevent maladaptations remain sparse. The present study administered a standard chow supplemented with high protein, leucine, fish oil and oligosaccharides, to determine whether cardiac and skeletal muscle remodeling could be attenuated during cardiac cachexia.

**Methods:** Female mice (C57/BL6) were treated for 8 weeks with saline (sham; *n* = 10) or monocrotaline (MCT; 600 mg/kg; n = 10) to induce cardiac cachexia in combination with a control diet (standard chow AIN‐93 M), while a further MCT‐treated group received supplemented nutrition (NS, isocaloric) (MCT + NS; n = 10). Subsequent histological analyses were performed in the right ventricle and tibialis anterior and microarray analysis was performed for the right ventricle.

**Results:** Compared to sham mice, relative body weight was impaired (*p* < 0.05) in MCT and MCT + NS by 5%. Compared to shams, MCT mice also showed an increase in heart weight, right ventricular thickness and fibrosis (all p < 0.05); however, these alterations were attenuated in MCT + NS mice. Subsequent Gene Set Enrichment Analysis of the right ventricle confirmed upregulation in fibrotic pathways in the MCT—compared to sham‐treated mice (*P* < 0.05), which were downregulated in the MCT + NS mice. In addition, skeletal muscle fiber cross‐sectional area was reduced (P < 0.05) by 22% in MCT compared to sham mice, but preserved in the MCT + NS group (1178 vs 1503 vs 1495 μm^2^, respectively), with protein expression of the key E3 ligase MuRF1 also reduced by 30% compared to MCT mice alone (p < 0.05).

**Conclusions:** A multi‐compound supplemented nutrition significantly attenuated both right ventricular and skeletal muscle maladaptations in a mouse model of cardiac cachexia. These data may provide a novel therapeutic strategy for combating pathophysiological alterations in cardiac cachexia.

**7-03**

**Profile of serum amino acids among community dwelling older adults from Hong Kong MrOs and MsOs cohort study: special reference to sarcopenia**

Liu‐Ying Zhu^1^, Ruth Chan^1^, Jason Leung^2^, Timothy Kwok^1,2^ and Jean Woo^1^

^1^ *Department of Medicine and Therapeutics, The Chinese University of Hong Kong, Hong Kong SAR;* ^2^ *Jockey Club Centre for Osteoporosis Care and Control, The Chinese University of Hong Kong, Hong Kong SAR*

**Introduction:** The availability of blood amino acids, especially the essential amino acids (EAAs) is the potent stimulus for muscle protein synthesis. We aimed to compare the profile of serum amino acids among sarcopenic and non‐sarcopenic community dwelling older adults from Hong Kong MrOs and MsOs cohort study.

**Methods:** Baseline data of 2997 Chinese adults aged >65 of Mr and Ms Os (Hong Kong) Cohort Study were analyzed. Sarcopenia was defined using the Asian Working Group Criteria. Serum amino acids levels (EAAs including valine, leucine, isoleucine, methionine, phenylalanine, tryptophan and non‐EAAs including glutamine, arginine, proline, etc.) were measured. Statistical analyses were performed separately for male and female.

**Results:** Mean age of the entire sample was 71.9 years old and 47.5% was male. There were 156 (5.2%) subjects (male *n* = 91, female *n* = 65) being defined to have sarcopenia. Serum levels of all amino acids of both sarcopenic and non‐sarcopenic groups met the requirement for adults above 18‐year‐old. When compared to non‐sarcopenic men, men with sarcopenia showed significantly lower serum levels of branched‐chain amino acids (leucine, isoleucine and valine *p* < 0.0001), methionine (*p* < 0.01), tryptophan (p < 0.01), proline (*p* = 0.039) and tyrosine (*p* = 0.016). In contrast, women with sarcopenia only showed lower levels of glutamine, glutamic acid, leucine and valine (*p* < 0.05) than women without sarcopenia, whereas no significant difference in other amino acids was observed between the two groups. After adjusting for age, body mass index, physical activity scale for the elderly, energy intake and instrumental activities of daily living impairment, only glutamine (*p* = 0.045) and homocysteine (*p* = 0.031) in male and tyrosine (*p* = 0.004) in female showed significant difference between sarcopenic and non‐sarcopenic subjects.

**Conclusions:** The role of amino acid and the effectiveness of AAs supplements may need further exploration with prospective randomized controlled trials for sarcopenic elderly.

**7-04**

**Evidence‐based nutritional and pharmacological interventions targeting chronic low‐grade inflammation in middle‐aged and older adults: a systematic review and meta‐analysis**

Carlo Custodero^1,2^, Robert T. Mankowski^1^, Stephanie A. Lee^1,3^, Todd M. Manini^1^, Marco Pahor^1^ and Stephen D. Anton^1^

^1^ *Department of Aging and Geriatric Research, University of Florida, Gainesville, FL, USA;* ^2^ *U.O.C. Medicina Interna Universitaria “C. Frugoni”, Dipartimento Interdisciplinare di Medicina (DIM), University of Bari “Aldo Moro”, Italy;* ^3^ *Department of Public Health, Social and Behavioral Sciences, Clinical and Translational Science, University of Florida, Gainesville, FL, USA*

**Introduction**: Chronic low‐grade inflammation could be one of the mechanisms underlying the aging process. Interleukin (IL)‐6 and C‐reactive protein (CRP), well‐recognized markers of this condition, are positively associated with impaired physical performance, frailty, sarcopenia and higher mortality. To date, few interventions have been identified to reduce chronic systemic inflammation and improve functional performance in seniors. Thus, the purpose of this review was to examine the state of evidence from relevant randomized controlled trials on the effects of nutritional and pharmaceutical compounds on established markers of inflammation in middle‐aged and older adults.

**Methods**: A systematic literature search was conducted in MEDLINE, PubMed and EMBASE databases using combinations of the target compounds which are promising, safe, affordable, acceptable, and practical (omega‐3, resveratrol, vitamin D, probiotics, Angiotensin Receptor Blockers [ARBs], metformin) with “inflammation” OR “c‐reactive protein” OR “interleukin‐6.” To be included, studies were limited to randomized controlled trials with placebo or control group receiving no treatment. Other key inclusion criteria were participants 45 years and older with chronic low‐grade inflammation as defined by IL‐6 levels between 2.5 and 30 pg/ml and/or CRP levels between 2 and 10 mg/L.

**Results**: Overall, 49 of 858 studies fulfilled the selection criteria. For IL‐6, the following compounds showed a significant effect compared to placebo: probiotics (−0.68 pg/ml), ARBs (−0.37 pg/ml) and omega‐3 (−0.19 pg/ml) (Table 1 and Figure 1). For CRP, a significant reduction was observed with probiotics (−0.43 mg/L), ARBs (−0.2 mg/L), omega‐3 (−0.17 mg/L) and metformin (−0.16 mg/L) (Table 1 and Figure 2).

**Table 1:** 

IL‐6 Summary Table						
Compound	**Total studies**	**N(INT)**	**N(PLA)**	**standardized mean differences (INT‐PLA)**	**95% CI**	**P‐value**
ARBs	4	199	181	**−0.37**	**[−0.59,−0.16]**	**0.0005**
Omega‐3	9	908	841	**−0.19**	**[−0.29,−0.10]**	**<0.0001**
Probiotic	4	89	91	**−0.68**	**[−1.01,−0.35]**	**<0.0001**
Resveratrol	3	144	139	−0.17	[−0.40,0.07]	0.164
Vitamin D	4	276	277	−0.09	[−0.26,0.07]	0.267

**Table 2:** 

CRP Summary Table						
Compound	**Total studies**	**N(INT)**	**N(PLA)**	**standardized mean differences (INT‐PLA)**	**95% CI**	**P‐value**
ARBs	6	250	231	**−0.2**	**[−0.39,−0.02]**	**0.03**
Metformin	7	1617	1630	**−0.16**	**[−0.22,−0.09]**	**<0.0001**
Omega‐3	16	1215	1200	**−0.17**	**[−0.26,−0.09]**	**<0.0001**
Probiotic	4	84	86	**−0.43**	**[−0.75,−0.12]**	**0.0063**
Resveratrol	3	74	73	−0.27	[−0.59,0.06]	0.106
Vitamin D	11	567	568	−0.06	[−0.18,0.06]	0.317

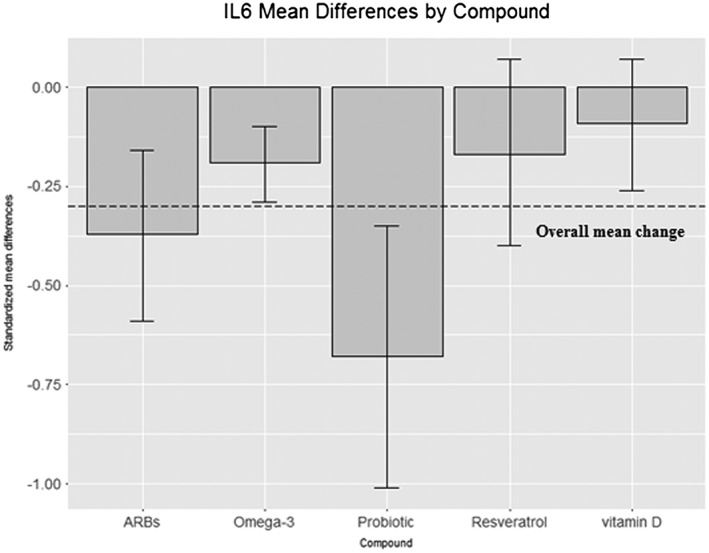

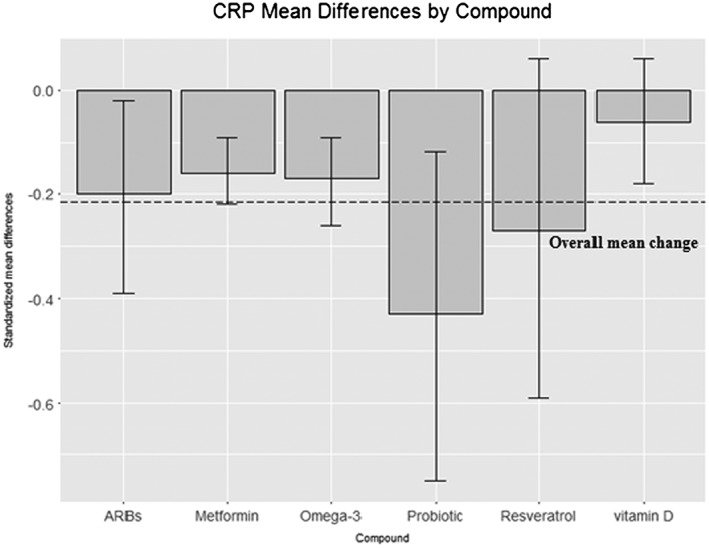

**Conclusions**: These results provide support for the potential of some nutritional and pharmaceutical compounds to significantly reduce established markers of systemic inflammation in middle‐age and older adults. Specifically, interventions utilizing probiotics, ARBs and omega‐3 produced significant reductions in IL‐6 levels, and interventions utilizing probiotics, ARBs, omega‐3 and metformin produced significant reductions in CRP.

 

**7-05**

**Nutritional and functional status in survival of free‐living mild Alzheimer's disease patients**

Odete V. Sousa^1,2,3^ and Teresa F. Amaral^1,4^

^1^ *Faculdade de Ciências da Nutrição e Alimentação, Universidade do Porto, Porto, Portugal;* ^2^ *UNIFAI ‐ Instituo de Ciências Biomédicas Abel Salazar, Universidade do Porto, Porto, Portugal;* ^3^ *Hospital de Magalhães Lemos E.P.E, Porto, Portugal;* ^4^ *UISPA – LAETA / INEGI, Faculdade de Engenharia, Universidade do Porto, Porto, Portugal*

**Background:** Nutritional status may be a modifiable factor in the progression of dementia. Moreover, dementia is an important cause of mortality, and nutritional status on prognosis and survival will be of interest to health and social care. A decline of nutritional and functional status is very common in the end stages of Alzheimer's Disease (AD). The aim of this study was to quantify the association between nutritional and functional status in survival of free‐living mild AD patients`.

**Methods:** A prospective observational study was conducted in 79 free‐living mild AD patients (32 men; age: 78.2 ± 6.6 years) between 2012 and 2017. Nutrition status was evaluated with body mass index (BMI) WHO cutoffs, FFM index ESPEN 2016 cutoffs, MNA full‐form score and bioimpedance analysis. Sarcopenia was defined according to EWGSOP 2010 criteria. Functional status using gait speed (GS) and handgrip strength (HGS) was determined. Kaplan–Meier and adjusted Cox proportional hazard ratio (HR) methods were applied.

**Results:** Among the 79 participants, 20 (25.3%) died during follow‐up (mean follow‐up period was 70.5 months). Forty‐one participants (51.9%) were underweight, 29.1% overweight/obesity and the remaining was at normal weight. Mortality risk was increased in underweight (HR: 3.29; 95%CI: 1.17–9.28; *p* = 0.024) compared to normal‐weight and overweight‐obese participants. HGS tercis (first [lowest]: HR: 4.26; 95%CI: 1.18–15.30; *p* = 0.026), low GS (HR: 2.70; 95%CI: 1.10–6.62; *p* = 0.029), low phase angle (HR: 2.51; 95%CI: 1.02–6.15; *p* = 0.044), undernutrition evaluated by MNA (HR: 4.77; 95%CI: 1.72–13.21; *p* = 0.003) and weight loss (HR: 2.92; 95%CI: 1.12–7.60; *p* = 0.028) increased the mortality risk. Underweight by BMI and, low muscle function by GS, were independently associated with higher probability of non‐survival (HRs of 3.65: 95%CI: 1.29–10.35; *p* = 0.015, and 2.85: 95%CI: 1.14–7.10; *p* = 0.024, respectively).

**Conclusions:** Underweight and low muscle function were associated with lower probability of survival in free‐living AD patients.

**7-06**

**Association between phosphatase and tensin homologue (PTEN) expression, calories supplemented and glucose control in young trauma patients**

Alessio Molfino^1^, Maria Ida Amabile^1^, Francesco Alessandri^2^, Alessio Farcomeni^3^, Paola Mosillo^4^, Donatella Dell'Utri^2^, Maurizio Muscaritoli^1^, Filippo Rossi Fanelli^1^ and Alessandro Laviano^1^

^1^ *Department of Clinical Medicine, Sapienza University of Rome, Rome, Italy;* ^2^ *Department of Cardiovascular, Respiratory, Nephrology, Anesthesiology and Geriatric Sciences, Sapienza University of Rome, Rome, Italy;* ^3^ *Department of Public Health and Infectious Diseases, Sapienza University of Rome, Rome, Italy;* ^4^ *Department of Physiology and Pharmacology "Vittorio Erspamer", Sapienza University of Rome, Rome, Italy*

**Introduction:** PTEN reduces insulin sensitivity and its expression increases during sepsis. Considering that critically ill patients present insulin resistance, we investigated the role of PTEN expression on glucose control and clinical outcome(s) in young adult patients hospitalized for trauma in an intensive care unit (ICU) receiving artificial nutrition.

**Methods:** This was an observational, single‐center study. Plasma glucose levels and its variability were recorded in patients. PTEN expression via western blotting analysis was measured, and the associations between PTEN, plasma glucose levels and glycemic variability, and calories administered were investigated. Parametric and non‐parametric tests were performed, as appropriate, and *P* < 0.05 was considered statistically significant.

**Results:** Twenty patients (13 men, mean age of 37.3 ± 12.7 years) were considered. No correlation between plasma glucose and PTEN was documented (*r* = −0.15, *P* = 0.55), neither between glycemic variability and PTEN expression (*r* = −0.00, *P* = 0.99). However, total kcal/day administered and PTEN expression significantly correlated (*r* = 0.56, *P* = 0.01). Also, patients with PTEN levels below the median value received less kcal/day than those with PTEN above the median (*P* = 0.048). This association was more pronounced when adjusted for body weight (*P* = 0.03) and for the average of insulin daily administered (*P* = 0.02).

**Conclusions:** PTEN expression might contribute to glucose homeostasis and disposal in critically ill patients receiving artificial nutrition.

**7-07**

**Pilot study to identify the modification of performance in sarcopenic nursing home patients after introduction of protein and D vitamin supplementation**

Milko Zanini^1^, Annamaria Bagnasco^1^, Gianluca Catania^1^, Giuseppe Aleo^1^, Stefania Ripamonti^2^, Davide Gonella^1^, Michela Barisone^1^, Nicoletta Dasso^1^ and Loredana Sasso^1^

^1^ *Department of Health Sciences Univeristy of Genova, Italy;* ^2^ *Private Outpatient Nutritional Service, Brugherio, MB, Italy*

**Introduction:** In the population between 60 and 70 years prevalence, ratio is from 5 to 13% and in more than 80 year old people the prevalence is between 11 and 50% 1.

The European Working Group on Sarcopenia in Older People (EWGSOP) defined a conceptual framework that shows three level of sarcopenia: pre‐sarcopenia, sarcopenia and high sarcopenia.

Recent studies indicate that a vitamin D and leucine‐enriched whey protein oral nutritional supplement can improve in muscle mass, and lower‐extremity function among sarcopenic older adults 2.

**Aim:** To improve the management of sarcopenic patients in the province of Genoa (IT) and to measure the prevalence of sarcopenia in elderly institutionalised in Genoa using the EWGSOP‐defined algorithm.

**Methods:** Transversal, prospective observational study in nursing homes.

Exclusion criteria: active tumoral disease; severe cognitive impairment; condition against bioimdenziometric analysis. Sarcopenia has been evaluated with the definition and EWGSOP‐defined algorithm physical performance: gait speed of using the 4‐meter walking; hand grip strength test using Dynax digital dynamometer and evaluation of Skeletal Muscle Index (SMI) using BIA vector analysis.

**Results:** In this pilot, we test 17 people from 2 different nursing homes; we found all 17 patient enrolled at high‐level sarcopenia. During observation, the patients were supplemented with Leucine and Vitamin D with by the nursing home physicians. We provide the same test after three months of therapy, and we found a light modification on EWGSOP indicators as shown in Table 1.

**Table:** 

	**Gait Speed**		**Hand Grip**		**SMI**	
	**t0**	**t3**	**t0**	**t3**	**t0**	**t3**
**media**	0,6	0,7	10,8	12	7,31	7,83
**SD**	0,27	0,29	4,65	5,05	1,52	1,46
**min**	0,36	0,26	3	3,7	4,7	5,7
**max**	1,5	1,39	18,7	18,3	9,8	10,5

**Discussion:** The improvement of EWGSOP parameter, even not significant, allow us to be confident in developing the next RCT to assess parameters in a larger sample of patients.

**7-08**

**Effect of the underlying renal disease on metabolic and nutritional parameters in older adults with reduced renal function**

Alessio Molfino^1^, Silvia Lai^1^, Maria Ida Amabile^1^, Silvia Altieri^2^, Daniela Mastroluca^3^, Carlo Lai^4^, Paola Aceto^5^, Massimiliano Crudo^6^, Filippo Rossi Fanelli^1^ and Maurizio Muscaritoli^1^

^1^ *Department of Clinical Medicine, Sapienza University of Rome, Rome, Italy;* ^2^ *Department of Clinical and Molecular Medicine, Sapienza University of Rome, Sant'Andrea Hospital, Rome, Italy;* ^3^ *Department of Internal Medicine and Medical Specialties, Sapienza University of Rome, Rome, Italy;* ^4^ *Department of Dynamic and Clinic Psychology, Sapienza University of Rome, Rome, Italy;* ^5^ *Department of Anesthesiology and Intensive care, Catholic University of Sacred Heart, Rome, Italy;* ^6^ *Software House INTECS S.p.A, Rome, Italy*

**Introduction:** Chronic kidney disease (CKD) is highly prevalent among older adults. Increased risk of cardiovascular disease is frequent in renal impairment, and metabolic and nutritional derangements are associated with CKD. We compared the metabolic, nutritional, and cardiovascular impact of reduced renal function between patients with and without known renal disease.

**Methods:** Consecutive outpatients aged ≥65 years with reduced renal function were enrolled and divided into 2 groups: Group A (patients with history of renal disease) and Group B (patients with unknown renal disease). Nutritional and metabolic parameters, including involuntary body weight loss (BWL) in the previous 6 months, mineral metabolism, inflammatory indices, and left ventricular mass index (LVMI), were determined. Parametric and non‐parametric tests were performed as appropriate, and *P* < 0.05 was considered statistically significant.

**Results:** Seventy‐six patients were enrolled. Group A (*n* = 39, M: 24) showed greater BWL with a significant reduction of 25‐hydroxyvitamin D, transferrin, cholinesterase, albumin, and greater LVMI with respect to Group B (*P* < 0.01). In addition, Group A showed significantly increased intact parathyroid hormone, total cholesterol, low‐density lipoprotein, triglycerides, and C‐reactive protein when compared to Group B (*P* < 0.05).

**Conclusions:** The positive history of renal disease may negatively impact on several nutritional and metabolic parameters related to increased cardiovascular risk among older adults.

**7-09**

**Sarcopenic obese and malnutrition risk are associated to low survival in a cohort of chronic heart failure patients independently**

Sheila Molinero‐Abad^1,2,3^, María Soto‐Célix^4^, Leticia Sánchez Gómez^1,2^, Ana Riego‐Valledor^1,2^ and Alberto Mijan‐de‐la‐Torre^1,2,3^

^1^ *Internal Medicine, Clinical Nutrition Unit, Hospital Universitario de Burgos, Burgos, Faculty of Medicine, University of Valladolid, Valladolid, Spain;* ^2^ *Clinical Nutrition Unit, Hospital Universitario de Burgos, Burgos;* ^3^ *Faculty of Medicine, University of Valladolid, Valladolid, Spain;* ^4^ *Clinical Nutrition Unit, Universidad Pontificia Católica de Chile, Santiago de Chile, Chile*

**Introduction:** Our previous studies have shown how Sarcopenia is a prevalent syndrome in CHF patients with a lower survival and malnutrition risk modifies survival in this setting too. Sarcopenic Obese account for a significant share. Now we wanted to check if sarcopenic obese modifies survival and his association with malnutrition risk.

**Methods:** Prospective cohort study (*n* = 103) patients with CHF (NYHA III‐IV) were consecutively selected. Nutrition risk screening (NRS‐2002), Mini‐Nutritional assesment (MNA) and Subjective global assessment (VGS) were performed. Muscle function was measured by hand‐grip dynamometry (kg)‐ lower limit <20(f); <30(m)‐. Appendiceal mass index and fat mass index were calculated (DUAL Energy‐X‐RayAbsorciometry (DXA)) and cut‐off values for Sarcopenia were set according to Rosetta Study. Sarcopenic Obese was set according Baumgartner study Patients were followed‐up and survival rate (months) registered. Data were stat analyzed by Kaplan–Meier, Log‐Rank Tests and Chi‐squared test. Statistical significance was reached at *p* < 0.05.

**Results:** One‐hundred three patients (50f, 82.5y; 53m, 82.5y) were selected. Global prevalence of malnutrition was 69.9% NRS‐2002 (67.3% women/71.7% men); 83.3% MNA (83.7% women/83%men) and 58.8% VGS (59.2% women/ 58.5% men). Sarcopenic Obese was detected in 17.5% (12.6% of men; 4.9% of women). Survival rate was not influenced by gender (*p* = 0.56), but was influenced by risk of malnutrition (*p* = 0.017 NRS; *p* = 0.08 MNA; *P* = 0.005 VGS) and of sarcopenic obese (*p* = 0.032). However, no association was shown between sarcopenic obese and risk of malnutrition using the Chi‐squared tests (*p* = 0.095 MNA; *p* = 0.214 NRS; *p* = 0.429 VSG).

Conclusions:

1. The risk of malnutrition is statistically associated with a lower survival.
2. Sarcopenic obese is statistically associated with a lower survival
3. No association has been showed between risk malnutrition and sarcopenic obese in our study
4. Further analysis to check if sarcopenic obese modifies risk malnutrition in this setting.

**7-10**

**Prognostic usefulness of arm circumference and nutritional screening tools in older patients with cardiovascular disease**

Takeshi Nakamura^1^, Kentaro Kamiya^2^, Atsuhiko Matsunaga^2^, Nobuaki Hamazaki^1,3^, Ryota Matsuzawa^3^, Kohei Nozaki^3^, Shinya Tanaka^1^, Emi Maekawa^4^, Takashi Masuda^2^ and Junya Ako^4^

^1^ *Kitasato University Graduate School of Medical Sciences, Sagamihara, Japan;* ^2^ *Department of Rehabilitation, Kitasato University School of Allied Health Sciences, Sagamihara, Japan;* ^3^ *Department of Rehabilitation, Kitasato University Hospital, Sagamihara, Japan;* ^4^ *Department of Cardiovascular Medicine, Kitasato University School of Medicine, Sagamihara, Japan*

**Introduction:** Several studies have shown that arm circumference (AC) and nutritional screening tools have prognostic capability in patients with cardiovascular disease (CVD). However, there have been no direct comparisons between AC and nutritional screening tools. This study was performed to compare the prognostic predictive capabilities of AC and nutritional screening tools in older patients with CVD.

**Methods:** The study population consisted of 1014 admitted patients aged ≥60 years with CVD. Patients underwent AC measurement and nutritional screening before hospital discharge. We used the controlling nutritional status index (CONUT score), the geriatric nutritional risk index (GNRI), and the prognostic nutritional index (PNI) as nutritional screening tools. The endpoint of the study was all‐cause mortality.

**Results:** The mean age was 73.0 ± 7.0 years, and 68.0% of the patients were male. A total of 134 deaths occurred over a median follow‐up period of 2.12 years (interquartile range, 2.62 years). After adjusting for preexisting prognostic factors (including age, gender, body mass index, estimated glomerular filtration rate, and log B‐type natriuretic peptide), AC (hazard ratio [HR]: 0.57; *p* < 0.001), CONUT score (HR: 1.24; *p* = 0.008), GNRI (HR: 0.76; *p* = 0.031), and PNI (HR: 0.78; *p* = 0.006) were significant predictors of mortality. However, adding AC to preexisting prognostic factors (0.733 vs. 0.705, respectively; *p* = 0.033), but not CONUT, GNRI, or PNI (0.717, 0.711, and 0.716 vs. 0.705; *p* = 0.108, *p* = 0.280, and *p* = 0.139, respectively) significantly increased the area under the curve on receiver operating characteristic analysis.

**Conclusions:** AC, but not nutritional screening tools, plays a complementary role to preexisting prognostic factors for predicting prognosis in older patient with CVD.

**7-15**

**Bioelectrical impedance analysis of body composition in obese patients undergoing medical nutrition therapy**

Nadja Vasiljevic, Dragana Davidovic, Branko Jakovljevic, Milos Maksimovic and Jagoda Jorga

*Institute of Hygiene and Medical Ecology, Faculty of Medicine, University of Belgrade, Dr Subotica 8, Belgrade, 11000, Republic of Serbia*

**Background and aim:** Body composition analysis is mandatory in monitoring the effect of dietary therapy. The aim of this study was to evaluate body composition variable before and after MNT in obese patients.

**Methods:** The study involved 714 subjects, 173 men and 541 women, who applied to the Department of Nutrition for MNT for obesity. The body composition analysis was performed using Tanita BC 418 MA bioelectric impedance for all patients. Values of fat mass (FM) and fat free mass (FFM) were obtained, and then the values of FM‐Index (FMI) and FFM‐index (FFMI) were calculated.

**Results:** Body composition analysis showed that FM and FFM values are higher in subjects with higher BMI. FMI and FFMI differed significantly among patients within different BMI categories, as well. These differences are statistically highly significant in persons of both sexes (*p* < 0.001). A statistically significant negative correlation (Pearson correlation −0.222; *p* = 0.003) was observed in age‐related analysis of FFMI in male subjects, while in female subjects this relationship was not statistically significant (*p* = 0.059). After following the hypocaloric diet, there is a decline in FFMI with increasing age (Pearson correlation −0.273; *p* = 0.007). A more detailed analysis shows that the FFMI value is significantly lower in the group of men older than 55 years (*p* = 0.005). In the group of women, this relationship is not observed (*p* = 0.720). FFMI decrease is in correlation with MNT duration. People who have been on regime for more than a month have a lower FFMI (p = 0.032).

**Conclusions:** For the purpose of preserving muscle mass, attention should be paid to reduction of FFM in all patients who are on restrictive regimens for long periods of time. Special attention should be given to men older than 55 years in order to prevent sarcopenia.

**7-16**

**Evaluation of an interdisciplinary Cachexia and Nutrition Support Clinic—the patient and carers perspective**

Vanessa C. Vaughan^1^, Meg Harrison^2^, Anna Dowd^2^, James Goonan^1^ and Peter Martin^1,2^

^1^ *School of Medicine, Deakin University, Waurn Ponds, VIC, Australia;* ^2^ *Barwon Health Cachexia & Nutrition Support Service, Barwon Health, North Geelong, VIC, Australia*

**Background/Aims:** The Barwon Health Cachexia & Nutrition Support Service is an outpatient clinic focused on improving clinical outcomes and quality of life for patients with or at high risk of cancer cachexia. Patients see an interdisciplinary team, incorporating a physician, physiotherapist, dietician and nurse practitioner concurrently. This study aimed to evaluate the patient and carer perspective of the service.

**Methods:** In 2016/17, semi‐structured interviews were conducted with 12 patients and 9 carers. Interviews focused on two broad themes: 1) recounting memories and experience of the Cachexia & Nutrition Support Clinic and 2) describing their ideal experience or expectation of a cachexia‐specific support service. All interviews were recorded and transcribed verbatim. Thematic analysis was supplemented with data from reviews of the patient and carer literature.

**Results:** Analysis generated four superordinate themes that reflected the complex dynamics of the clinic experience. Themes were improved communication regarding health literacy/education for patients and carers, empowerment through person‐centred care, evolution of perception of value, and importance of the interdisciplinary team‐based approach. Generally, patients and carers reported overall positive experiences with the clinic, particularly with regard to improved communication and empowerment of the patient.

**Conclusions:** Findings confirmed that a cachexia‐specific service was viewed as having a positive impact on quality of life and outcomes by patients and carers. A patient‐centred and individualised approach by the interdisciplinary team in particular were of importance to those interviewed. These insights are a critical step in the development of recommendations for future clinical management of cancer cachexia.

**7-17**

**Contribution of reduced food intake to cancer‐associated weight loss: data from the International Cancer Cachexia Data Repository**

Lisa Martin^1^, Cathy Kubrak^2^, Barry Laird^3^, Bruno Gagnon^4^, Martin Chasen^5^ and Vickie Baracos^2^

^1^ *Department of Agricultural, Food & Nutritional Science, University of Alberta, AB, Canada;* ^2^ *Department of Oncology, University of Alberta, AB, Canada;* ^3^ *University of Edinburgh, Edinburgh, UK; and European Palliative Care Research Centre;* ^4^ *Department of Family Medicine and Emergency Medicine, Université Laval, Quebec, Canada;* ^5^ *Division of Palliative Care, University of Ottawa, Ottawa, Canada*

**Background:** In defining diagnostic criteria for cancer cachexia, a first step was achieved with the classification of cancer‐associated weight loss (WL Grades) risk stratified for mortality.^1^, ^2^ Next steps relate to the respective roles of reduced food intake and altered metabolism in driving WL.^3^ Here, we tested the prognostic significance of reduced food intake, using a validated clinical tool designed for patient report.^4^

**Methods:** Canadian and European patients with cancer formed an international data set (*N* = 6301). Included patients had recorded overall survival (OS), WL Grade (0 to 4),^1^ and Patient Generated‐Subjective Global Assessment (PGSGA) categories of current food intake.^4^ Patients relying exclusively on artificial nutrition were excluded. Data were entered into multivariable Cox proportional hazard (outcome = OS) and multinomial logistic regression (MLR; outcome = WL Grade) models, adjusted for age, sex, cancer diagnosis (head and neck, respiratory, colorectal, gastroesophageal, others) and stage, performance status, and WL grade.

**Results:** In the adjusted Cox model, PGSGA food intake categories independently predicted OS (*P* < 0.001). Compared to “*normal food in normal amount*” (*N* = 2190) categories signifying reduced food intake had worse survival:

*“normal food in reduced amount”* (*N* = 2804, HR 1.2; 95% CI 1.1–1.3, *P* < 0.001);

*“little solid food”* (*N* = 600, HR 1.6; 95% CI 1.4–1.8, *P* < 0.001);

*“only liquids”* (*N* = 129, HR 1.8; 95% CI 1.5–2, *P* < 0.001);

*“only nutritional supplements (ONS)”* (*N* = 166, HR 1.9; 95% CI 1.6–2.3, *P* < 0.001);

*“very little of anything”* (*N* = 412, HR 1.8; 95% CI 1.6–2.0, *P* < 0.001).

In the adjusted MLR model, patients with reduced food intake were more likely to have higher WL grades (Grade 1, 2, 3, or 4 vs. Grade 0, P < 0.001). For example, the probability of Grade 4 WL for patients with “*normal food in normal amount*” = 0.09, “*normal food in reduced amount*” = 0.25, “*little solid food*” = 0.37, “*only ONS*” = 0.43, “*only liquids*” = 0.47, and “*very little anything*” = 0.43.

**Conclusions:** Patient‐reported food intake categories provide a practical clinical assessment prognostic of OS and higher WL grades.

**7-18**

**Current use of parenteral and enteral nutrition in the oncological setting of a French University Hospital: adequacy with the recommendations, efficacy, complications and costs**

Lucie Monceau‐Baroux^1^ and Jacques Delarue^2^

^1^ *Brest University Hospital, France;* ^2^ *Brest University Hospital, France*

**Introduction:** International evidence‐based recommendations regarding the prescription of parenteral (PN) and enteral nutrition (EN) in cancer patients are now widely available (ESPEN, ASPEN). However, data from the literature shows that real‐life practice does not always follow those recommendations, especially in the palliative situation. Financial toxicity is a new key parameter in medical oncology because more expensive antineoplastic drugs become available every year and the French Social Security might not be able to take all the costs in charge in the future. Nutritional management being part of the supportive care provided to cancer patients, investigating the costs represented by PN and EN is mandatory.

**Methods:** The aim of this study was to evaluate if the initial prescriptions of artificial nutrition (PN and EN) made to adult patients with solid tumors in one French University Hospital during the year 2015 complied with the international recommendations available at that time. Secondary objectives were to study the evolution of the nutritional status of the patients under nutritional support, overall survival from the prescription time, the rates of septic, metabolic and mechanical complications and the costs.

**Results:** Between the 01/01/2015 and the 12/31/2015, 111 patients (men: 62%, women 38%) received parenteral nutrition for the first time and 137 patients (men: 80%, women: 20%) received enteral nutrition for the first time.

Complete data and analysis will be available in the poster.

**Conclusions:** The end goal of this study is to have a precise look at the current practice inside the institution in order to standardize the prescriptions in the future.

F6‐05

**7-19**

**Evaluation of the nutritional status in patients with incurable cancer**

Eliana De Rosa, Lidia Santarpia, Maurizio Marra, Maria Carmen Pagano, Lucia Alfonsi, Franco Contaldo and Fabrizio Pasanisi

*A.O.U. Federico II, Naples, Italy*

**Introduction:** Patients with advanced cancer frequently experience malnutrition and cachexia which, in turn, may negatively influence morbidity, mortality and quality of life (QoL). The aim of this study was to evaluate the nutritional status of incurable cancer patients and its correlation with survival and performance status.

**Methods:** The nutritional status of 30 consecutive incurable cancer patients (9 men, 21 women, aged 60 ± 9 years), eligible for home parenteral nutrition, was evaluated. The prevalence of cachexia was estimated according to the grading system developed by Martin *et al*. in 2015, whilst sarcopenia was evaluated based on Janssen *et al*.'s 2004 sex‐specific cut‐points for skeletal muscle (SM) normalized for height.

**Results:** According to the applied criteria, 100% of patients were cachectic. The prevalence of moderate and severe sarcopenia was 44.4% in men and 19% in women, 33.3% in men and 28.6% in women, respectively. Karnofsky score was ≤50 in 60% and >50 in 40% of patients. Prealbumin serum levels significantly correlated with survival (*R* ^2^ = 0.430, *p* < 0.05). Mean REE (Resting Energy Expenditure) normalized for fat‐free mass (FFM), (REE/FFM, 30.4 ± 4.5 kcal/kg), was in accordance with age‐ and sex‐specific values for healthy subjects reported in the literature. REE, REE/FFM and phase angle were significantly higher in the subgroup of patients with Karnofsky >50.

**Conclusions:** Prealbumin dosage and body composition evaluation provide useful information on patients' prognosis and performance status, in order to identify incurable cancer patients eligible for nutritional therapy.

**7-20**

**Weight history and nutritional status in advanced cancer patients**

Eliana De Rosa, Lidia Santarpia, Maurizio Marra, Maria Carmen Pagano, Lucia Alfonsi, Franco Contaldo and Fabrizio Pasanisi

*A.O.U. Federico II, Naples, Italy*

**Introduction:** Overweight and obesity are reported in 40–60% of cancer patients. It's not clear if obesity provides a survival advantage in patients with diseases associated with muscle wasting, certainly it could mask the presence of underlying malnutrition. This study compared the nutritional status in cancer patients with and without a history of overweight/obesity.

**Methods:** We evaluated the nutritional status of 25 consecutive advanced cancer patients (6 men, 19 women, aged 59 ± 8 years) candidate to home parenteral nutrition. Anthropometric measures and weight history were collected. Resting energy expenditure (REE) was measured by indirect calorimetry. Body composition and skeletal muscle mass (SM) were assessed by bioimpedance analysis. Janssen *et al*.'s 2004 sex‐specific cut‐points for SM normalized for height (SMI) were used to assess the presence of sarcopenia.

**Results:** Despite mean body mass index (BMI) was 19.6 ± 3.0 kg/m^2^, and no patients resulted to be obese at the visit, mean habitual BMI was 27.4 ± 7.15 kg/m^2^, in particular 32% of patients had a history of obesity and 24% of overweight. Mean REE and fat‐free mass (FFM) were significantly lower (*p* < 0.05) in patients with habitual BMI <25 kg/m^2^ (subgroup 1) than in patients with habitual BMI ≥25 kg/m^2^ (subgroup 2), whilst REE/FFM, SM and SMI showed no significant differences between the two subgroups. The prevalence of sarcopenia was 72.7% in subgroup 1 and 37.7% in subgroup 2. On the other hand, a significantly higher (*p* = 0.001) weight loss, both absolute and in percentage, was reported by subgroup 2; similarly, main laboratory markers of malnutrition (serum albumin, transferrin, prealbumin, pseudocholinesterase) were significantly lower (*p* < 0.05) in subgroup 2 than in subgroup 1.

**Conclusions:** This study highlights the importance of weight history in the nutritional assessment of cancer patients, since it allows to identify patients with a higher nutritional risk, even when body composition seems to be preserved.

**8-01**

**Small‐molecule inhibition of MuRF1 attenuates skeletal muscle atrophy and dysfunction in cardiac cachexia**

Volker Adams^1^, T. Scott Bowen^1^, Tina Fischer^1^, Sarah Werner^1^, Axel Linke^1^, Peter Sehr^2^, Joe Lewis^2^, Dittmar Labeit^3,4^, Alexander Gasch^3^ and Siegfried Labeit^3,4^

^1^ *Department of Internal Medicine and Cardiology, University Leipzig—Heart Center, Leipzig, Germany;* ^2^ *Chemical Biology Core, EMBL Heidelberg, Germany;* ^3^ *IPM, Dept. for Integrative Pathophysiology, Universitätsklinikum Mannheim University of Heidelberg, Theodor‐Kutzer‐Ufer 1‐3, Mannheim, Germany;* ^4^ *Myomedix GmbH, Neckargemünd, Germany*

**Background:** MuRF1 is a muscle‐specific ubiquitin E3 ligase that is activated during clinical conditions associated with skeletal muscle wasting, like cardiac cachexia, heart failure or cancer. Yet there remains a paucity of therapeutic interventions that directly inhibit MuRF1 function, particularly in vivo. The current study, therefore, developed a novel compound targeting the central B‐box‐coiled coil domain of MuRF1 in order to inhibit muscle wasting in cardiac cachexia.

**Methods:** We identified small molecules that interfere with the MuRF1‐titin interaction from a 130,000 compound screen based on Alpha technology. A subset of 9 prioritized compounds were synthesized by Enamine at 10 g scale and administrated during conditions of muscle wasting, i.e. to C2C12 muscle cells treated with dexamethasone and to mice treated with monocrotaline to induce cardiac cachexia.

**Results:** The 9 selected compounds inhibited MuRF1‐titin complexation with IC_50_ values <25 μM; of which 3 were found to also inhibit MuRF1 E3 ligase activity, with 1 further showing low toxicity on cultured myoblasts and myotubes. This last compound, EMBL chemical core ID#704946, also prevented atrophy in myotubes induced by dexamethasone and attenuated fiber atrophy and contractile dysfunction in mice during cardiac cachexia. Proteomic and Western blot analyses showed that stress pathways were attenuated by ID#704946 treatment, including downregulation of MuRF1, and normalization of proteins associated with apoptosis (BAX) and protein synthesis (elF2B‐delta). Furthermore, actin ubiquitinylation and proteasome activity was attenuated.

**Conclusions:** We identified a novel compound directed to MuRF1's central myofibrillar protein recognition domain. This compound attenuated in vivo muscle wasting, and contractile dysfunction in cardiac cachexia by protecting de novo protein synthesis and by downregulating apoptosis and ubiquitin‐proteasome‐dependent proteolysis. Further studies have to show if this newly developed compound is also suitable to treat atrophy and muscle dysfunction in other diseases.

**8-02**

**Dantrolene can protect muscle from toxicity induced by chemotherapy using docetaxel**

Baptiste Jude^1^, Thomas Castel^1^, Florine Tissier^1^, Karelle Léon^1^, Mickael Droguet^1^, Marie‐Agnès Giroux‐Metges^1,2^ and Jean‐Pierre Pennec^1^

^1^ *EA 4324 ORPHY, IBSAM, UFR Médecine et Sciences de la Santé, Université de Bretagne Occidentale, Brest, France;* ^2^ *Explorations Fonctionnelles Respiratoires, CHRU de Brest, Brest, France*

**Introduction:** Many cancers induce cachexia but chemotherapy itself can also induce cachexia associated to muscle atrophy, inflammation and could be a limiting factor of cancer treatment. The aim of this study was firstly to investigate the effects of the docetaxel (Tax), on muscle mass and inflammation. Secondly, an inhibitor of calcium ryanodine channels, the dantrolene (Dant), was tested in order to know if it can reduce the muscular toxicity of docetaxel.

**Methods:** Three groups were realized: Sham, Tax (docetaxel at 10 mg/kg), and DanTax (dantrolene at 10 mg/kg 2 h before and 8 h after docetaxel). In a first series, animals were sacrificed 24 h after treatments for determination of plasma cytokines and for the activated muscle pathways. In a second series, animals were sacrificed 7 days after treatments for muscle contraction recording and atrophy analyses. All values are compared to Sham group.

**Results:** 24 h after treatments, docetaxel‐induced muscle atrophy up to 12% for soleus, 13% for peroneus and 19% for diaphragm whereas in DanTax group it was mitigated with respectively only 7%, 4.2% and 4.5%. For cytokine profile, docetaxel led to an increase of INF‐γ (+280%), IL‐1β (+780%), IL‐6 (+232%) and TNF‐α (+400%), but in DanTax group all concentrations came back to control. 7 days after treatments, there was no significant atrophy in Tax Group (−4.5% for soleus and −17.5% for diaphragm) but in DanTax group the muscle mass was increased by +9% for soleus and +7.6% for diaphragm. For peroneus, atrophy was the same in both groups (−4%). Maximal force of peroneus contraction decreased in Tax group to 179.2 g compared to Sham group (215.3 g), and in DanTax group the force was restored (213.8 g).

**Conclusions:** The results indicated that docetaxel induces muscle atrophy associated to the release of pro‐inflammatory cytokines, and that dantrolene can protect muscle from the deleterious effects induced by docetaxel.

**8-03**

**ACVR2B/Fc counteracts chemotherapy‐induced loss of muscle and bone mass**

Rafael Barreto^1^, Yukiko Kitase^2,3^, Tsutomu Matsumoto^2,3^, Matthew Prideaux^2,3^, Fabrizio Pin^2,3^, Kyra C. Colston^4^, Katherine E. Couch^4^, Marion E. Couch^5,6,7^, Thomas M. O'Connell^3,5,6,7^, Lynda F. Bonewald^2,3,6^ and Andrea Bonetto^1,2,3,5,6,7^

^1^ *Department of Surgery, Indiana University School of Medicine, Indianapolis, IN, USA;* ^2^ *Department of Anatomy and Cell Biology, Indiana University School of Medicine, Indianapolis, IN, USA;* ^3^ *Indiana Center for Musculoskeletal Health, Indiana University School of Medicine, Indianapolis, IN, USA;* ^4^ *Indianapolis Project STEM, Indiana University School of Medicine, Indianapolis, IN, USA;* ^5^ *Department of Otolaryngology—Head and Neck Surgery, Indiana University School of Medicine, Indianapolis, IN, USA;* ^6^ *Simon Cancer Center, Indiana University School of Medicine, Indianapolis, IN, USA;* ^7^ *IUPUI Center for Cachexia Research Innovation and Therapy, Indiana University School of Medicine, Indianapolis, IN, USA*

Despite recent progress, chemo‐ and radiotherapy remain the primary treatment strategies for cancer, whose development is frequently accompanied by the occurrence of cachexia, a debilitating condition mainly characterized by muscle and fat depletion. We recently showed that chemotherapy, among other toxicities, also contributes to the development of cachexia by affecting muscle size and strength. Interestingly, several reports suggest that larger muscle mass correlates with reduced chemotherapy toxicity and better outcomes. Along this line, ACVR2B/Fc, an inhibitor of the Activin Receptor 2B signaling, was shown to preserve muscle mass and prolong survival in tumor hosts, as well as to increase bone tissue in mouse models of osteogenesis imperfecta and muscular dystrophy. Hence, we aimed at investigating the use of ACVR2B/Fc to prevent chemotherapy‐associated toxicities, including muscle and bone depletion.

In order to do so, we compared the effects of ACVR2B/Fc on body composition, as well as muscle and bone mass in mice exposed to Folfiri, a combination of 5‐fluorouracil, leucovorin and irinotecan, for 5 weeks. Here we show that ACVR2B/Fc significantly prevented Folfiri‐induced loss of muscle mass and muscle strength. Moreover, it completely counteracted the loss of trabecular bone in both femurs and vertebrae following Folfiri administration, as suggested by the analysis of trabecular bone volume fraction (BV/TV), thickness (Tb.Th), spacing (Tb.Sp), number (Tb.N) and connectivity density (Conn.Dn). Of interest, Folfiri or ACVR2B/Fc did not affect the cortical bone in the mouse femur tissue, as shown by unchanged cortical bone volume fraction (Ct.BV/TV), cortical thickness (Ct.Th) and porosity.

Overall, our results suggest that ACVR2B/Fc effectively prevents the loss of muscle and bone mass, as well as muscle weakness, in animals chronically exposed to chemotherapy. Future studies will need to validate whether the same protective effects are observed in combination with different anticancer regimens and/or cancer types.

**8-05**

**A novel Antibody‐Oligonucleotide Conjugate (AOC) platform enables efficient regulation of muscle targets in mice**

Beatrice D. Darimont, Rob Burke, Hanhua Huang, Venkata Ramana Doppalapudi, Rachel Johns, Palani Balu, Michael Cochran, Maria Shahmoradgoli, David Chu, Gulin Erdogan, Y. Chen, H.W. Kwon, Y. Shi, M. Hood, M. Moon, A. Cortes, J.D. Arias, Anneke K. Raney and Andrew Geall and Arthur A. Levin

*Avidity Biosciences, 10975 North Torrey Pines Road, Suite150, La Jolla, CA, USA*

Although muscular dystrophy and other diseases of muscle are well suited to treatments using antisense oligonucleotides or siRNAs, efficient delivery remains the key challenge for oligonucleotide‐based therapies to fulfill their full potential. Targeting oligonucleotides to an endocytosing liver‐specific receptor (ASGPR) through conjugation to its ligand GalNAc enhanced delivery of oligonucleotides into hepatocytes and led to various therapies for diseases involving the liver.

Here we demonstrate that conjugation of oligonucleotide payloads to antibodies specific for the transferrin receptor (TfR1) facilitates efficient delivery of siRNAs and single stranded morpholino oligonucleotides to muscle and other organs. A single IV administration of 3 mg/kg of a TfR1 mAb‐myostatin siRNA conjugate produced significant (>70%) reductions in levels of myostatin mRNA/protein in vivo. The knockdown of myostatin lasted for more than 5 weeks and resulted in increased muscle size and grip strength in wild‐type mice. An RISC loading assay demonstrated that these phenotypic effects were mediated through an RNAi mechanism. A single dose (5 mg/kg) of a TfR1 mAb‐morpholino conjugate enabled exon skipping of exon 23 in dystrophin pre‐mRNA in wild‐type mouse skeletal muscle, demonstrating the ability of antibody oligonucleotide conjugates (AOCs) to support delivery of diverse oligonucleotide payloads.

The ability to recruit antibodies and other protein‐based scaffolds to deliver oligonucleotides to specific cell surface receptors for internalization broadens the number of tissues and diseases that can be targeted with oligonucleotide therapeutics. The specificity of these therapies can be enhanced through careful selection of the targeted receptor and/or the oligonucleotide payload. In addition, delivery of multiple oligonucleotide payloads and/or the use of therapeutic antibodies enables potentially greater potency and the regulation of multiple cellular targets. Studies are ongoing to identify additional receptors suitable for oligonucleotide delivery and therapeutic oligonucleotides for the treatment of muscle dystrophies, sarcopenia, cachexia, and other muscle disorders.

**8-06**

**Mechanisms involved in the cross‐talk between humoral and mechanical cues underlying muscle wasting in cachexia**

Alexandra Baccam^1,2^, Medhi Hassani^2^, Alexandra Benoni^1^, Martina Ramella^3^, Francesca Boccafoschi^3^, Ara Parlakian^1^, Zhenlin Li^1^, Zhigang Xue^1^, Sergio Adamo Sergio^2^ and Dario Coletti^1,2^

^1^ *Dept. of Biological Adaptation and Ageing B2A (CNRS UMR 8256 – INSERM ERL U1164 – UPMC P6), Pierre et Marie Curie University Paris 6, France;* ^2^ *DAHFMO Unit of Histology and Medical Embryology, and Interuniversity Institute of Myology, Sapienza University of Rome, Italy;* ^3^ *Dept. Of Health Sciences, University of Eastern Piedmont Avogadro, Novara, Italy*

**Introduction:** Exercise training improves quality of life and survival of cancer patients. In an animal model of cancer cachexia we demonstrated that wheel running counteracts cachexia by releasing the autophagic flux. Exercise pleitropic effects include the alteration of circulating factors in favour of an anti‐inflammatory environment and the activation of mechanotransduction pathways in muscle cells. Our goal is to assess whether mechanostransduciton *per se* is sufficient to elicit exercise effects in the presence of pro‐cachectic factors of tumor origin. Serum response factor (SRF) is a transcription factor of pivotal importance for muscle homeostasis, which is activated with its co‐factor MRTF by mechanostranduction in a way dependent on actin polymerisation.

**Methods:** We use C26 tumor‐bearing mice, in the absence or presence of wheel running, and mixed cultures of C2C12 myotubes and myoblasts treated with C26 conditioned medium (CM) in the absence or presence of cyclic stretch to mimic the mechanical stimulation occurring upon exercise.

**Results:** *In vivo* both SRF expression and activity are differentially modulated by the C26 tumor, i.e. by humoral factors, and by exercise. *In vitro* we showed that CM had a negative effect on muscle cell cultures, both in terms of myotube atrophy and of myoblast recruitment and fusion, and that these effects were counteracted by cyclic stretch. We showed that CM repressed SRF‐MRTF transcriptional activity, while mechanical stretch rescued their transcriptional activity; in addition, loss of function experiments demonstrated that SRF was necessary to mediate the beneficial effects of mechanical stimulation on muscle cells. At least part of the observed effects were mediated by the balance of pro‐ and anti‐myogenic factor of the TGFbeta superfamily.

**Conclusions:** We propose that the positive effects of exercise on cancer patients and mice may be specifically due to a mechanical response of muscle fibers affecting the secretion of myokines.

**8-07**

**Efficacy of a novel selective androgen receptor modulator (TEI‐SARM2) with once weekly dosing in rat unloaded muscle atrophy and Duchenne muscular dystrophy models**

Masanobu Kanou, Kyohei Horie, Katsuyuki Nakamura, Toshie Jimbo, Hiroyuki Sugiyama and Kei Yamana

*Teijin Pharma Ltd, Tokyo, Japan*

**Introduction:** TEI‐SARM2, a non‐steroidal selective androgen receptor modulator (SARM), induces strong anabolism in muscle with minimal effects on reproductive tissues with once‐weekly dosing regimen. In cancer cachexia models, TEI‐SARM2 prevented body weight loss and improved survival rate. In this study, effects of TEI‐SARM2 in animal models of unloaded muscle atrophy (tail‐suspension) and Duchenne muscular dystrophy (DMD) were examined.

**Methods:** Study‐1: Female Wistar rats (12‐wk‐old) were suspended by the tail to avoid a contact of the hindlimb with the ground. On day 14, the rats were released from the suspension to recover for further 14 days. Muscle weight was measured on days 14, 21 and 28. TEI‐SARM2 was administered orally once weekly at 30 mg/kg for 28 days from the day of suspension starting. Study‐2: DMD rats (dystrophin deficient, 5‐wk‐old) were administered orally once‐weekly at 3–30 mg/kg. Grip force was measured once a month. After 6‐month treatment, the rats were monitored for hindlimb force by torque equipment.

**Results:** Study‐1: TEI‐SARM2 prevented rapid loss of muscle weight by tail‐suspension and accelerated recovery of gastrocnemius muscle weight to the level of intact animals within 2 weeks after reloading. Study‐2: TEI‐SARM2 preserved grip force during 6 months in DMD rats. After 6 months, preservation of torque force of hindlimb was also observed. Surprisingly, anabolic effect was not confirmed in appendicular muscles, suggesting that there is novel mechanism of TEI‐SARM2 action to improve muscle function other than anabolism.

**Conclusions:** TEI‐SARM2 prevents muscle atrophy in atrophic condition and promotes recovery by once‐weekly dosing regimen. In addition, TEI‐SARM2 preserves muscle force in DMD rats apart from muscle hypertrophy. Taken together, TEI‐SARM2 could be a promising drug candidate for various muscle disorders such as disuse muscle atrophy after hip fracture, DMD and cancer cachexia.

**8-08**

**IL‐4 counteracts cancer‐induced skeletal muscle atrophy: the double‐edged sword of IL‐4**

Domiziana Costamagna^1,2^, Robin Duelen^1^, Fabio Penna^3^, Paola Costelli^3^ and Maurilio Sampaolesi^1,2^

^1^ *Translational Cardiomyology, Stem Cell Biology and Embryology, Department of Development and Regeneration, University Hospital Gasthuisberg, Leuven, Belgium;* ^2^ *Division of Human Anatomy, Dept. of Public Health, Experimental and Forensic Medicine, University of Pavia, Italy;* ^3^ *Department of Clinical and Biological Sciences, University of Turin, Italy*

**Background and aims:** Terminal cancer patients frequently present cancer cachexia, a syndrome causing fat and skeletal muscle weight loss and interfering with antineoplastic therapies. Interestingly, cancer‐induced muscle depletion seems not only due to hyper‐activation of the main proteolytic systems in muscle tissue but also to an impairment of Satellite Cells (SCs). Indeed, it has already been reported an increase in the number of activated myogenic precursors in cachectic muscle, likely unable to complete the regeneration *in vivo* (Penna *et al*., 2010).

Next to SCs, other cell populations are involved in myogenic differentiation, such as mesoangioblasts (MABs), vessel‐associated muscle progenitors and Fibro Adipogenic Progenitors (FAPs). Recently, we demonstrated that BMP‐SMAD‐signaling blockade improved MAB myogenic differentiation (Costamagna *et al*., 2015). Moreover, Fibro Adipogenic Progenitors (FAPs) have the potential to enhance the differentiation rate of myogenic progenitors after injury and during disease progression (Joe *et al*., 2010; Mozzetta *et al*., 2013).

In this study, we investigated the role of interstitial cells in colon adenocarcinoma‐C26 bearing mice focusing on IL‐4 ability to prevent the detrimental adipocyte differentiation of FAPs in cachectic muscles.

**Results:** Body weight loss and decreased fiber CSA were partially rescued in C26‐bearing mice daily treated with IL‐4 (C26 + IL‐4) with respect to C26 mice. Interestingly, muscle CSA was increased as well in injured muscles from C26 + IL‐4 mice when compared to injured C26, suggesting a faster regeneration ability. Moreover, IL‐4 was able to induce a decrease in FAPs obtained from C26 + IL‐4 muscle explants with respect to C26 ones.

**Conclusions:** The results demonstrated that IL‐4 treatment can partially rescue muscle atrophy in tumor bearing mice undergoing cancer cachexia, probably due to anti‐inflammatory properties. Further experiments are needed to analyze which are the pathways activated by IL‐4 and how this could affect different cell types present in the muscle.

**8-09**

**BIO103 a drug candidate for the treatment of muscle wasting disorders**

Maria Serova^1^, Blaise Didry‐Barca^1^, Sissi On^1^, Anne‐Sophie Foucault^1^, Sophie Raynal^1^, Stanislas Veillet^1^, Pierre Dilda^1^ and René Lafont^2^

^1^ *Biophytis, UMPC – BC9, 4 place Jussieu, Paris, France;* ^2^ *Sorbonne Universités, UPMC Univ Paris 06, CNRS ‐ Institut de Biologie Paris Seine (BIOSIPE), Paris, France*

**Background:** Muscle wasting disorders, including cachexia and sarcopenia, are multifactorial diseases which contribute to overall physical frailty. They represent a worldwide health challenge with limited therapeutic options. The aim of this study was to characterize a new small molecule, BIO103, *in vitro* on myocytes and *in vivo* on a mouse model designed to analyse the effects of aging and muscle disuse.

**Methods:** Mouse C2C12 myoblasts were induced to differentiate, and myotube diameters were measured under fluorescent microscopy. Baseline and phosphorylated protein levels were assessed by Western blot. Relative levels of mRNA expression were evaluated using qRT‐PCR. Oxygen consumption was recorded using a Seahorse XF Analyzer. For *in vivo* studies, 22‐month‐old C57BL/6J mice were employed. Whole animal physical performances, the *in situ* functionality of *tibialis anterior*, and molecular markers of selected muscles were measured after 4 months of treatment.

**Results:** Using C2C12 cells, BIO103 increased myotube diameter consistently with a reduction of myostatin and atrogin gene expression. It led to a rapid activation of AKT/mTOR, MAPK and AMPK signalling pathways and reduced acetyl‐CoA carboxylase activity, suggesting effects on energy homeostasis and fatty acid synthesis. Moreover, BIO103 was shown to increase the oxygen consumption rate in muscle cells. In vivo, BIO103 compensated for the significant aging‐related loss of running velocity (*p* < 0.05). These results were associated with increased IGF‐1 plasma levels (*p* < 0.05), elevated level of p‐AMPKα and p‐AMPKβ and lower myostatin gene expression in the gastrocnemius of BIO103‐treated animals under high‐fat diet.

**Conclusions:** BIO103, a new orally available small molecule, displays both *in vitro* and *in vivo* anabolic properties, activates AKT/mTOR and AMPK which translates into improved functional performance, notably in old animals. These investigations demonstrate the potential of BIO103 in improving skeletal muscle quality and warrant further studies towards its development as a drug candidate for the treatment of muscle wasting disorders.

**8-10**

**The effects of ERK silencing on muscle cell culture phenotype**

Aline R.R. Lima^1^, Luis H. Zucoloto^1^, Paula P. Freire^2^, Mariana J. Gomes^1^, Luana U. Pagan^1^, Thierres H.D. Pontes^1^, Éder A. Rodrigues^1^, Robson F. Carvalho^2^, Katashi Okoshi^1^ and Marina P. Okoshi^1^

^1^ *Botucatu Medical School, Sao Paulo State University – UNESP, Brazil;* ^2^ *Botucatu Bioscience Institute, Sao Paulo State University – UNESP, Brazil*

**Introduction:** The mitogen‐activated protein kinase family, especially ERK, is necessary for skeletal muscle mass maintenance. Although rats with diseases such as heart failure and cancer present muscle atrophy and reduced ERK expression, the role of ERK in muscle differentiation and phenotype is poorly understood.

**Purpose:** To identify phenotypic effects of a decrease in skeletal muscle ERK protein, ERK gene was silenced in C2C12 muscle cell culture.

**Methods:** ERK gene silencing was performed by specific RNA interference (siRNA‐ERK). Post muscle differentiation phase, slides were stained with anti‐miosin heavy chain antibody and analyzed by immunofluorescence. Comparison between groups was performed by Student's t test.

**Results:** The number of nuclei in multinucleated cells (≥3 nuclei) did not differ between groups (Control 77.7 ± 22.6; siRNA‐ERK 64.7 ± 23.2; *p* = 0.10). Other data are shown in the table.

**Conclusions:** ERK has an essential role in myoblast differentiation into myotube and is important to properly myotube formation and development. These results corroborate the fact that muscle atrophy is associated with a reduction in ERK expression.

**Table:** 

	**Control**	**siRNA‐ERK**
**Number of nuclei**	116.2 ± 31.7	167.4 ± 79.0*
**Number of mononucleated cells**	38.6 ± 21.4	102.7 ± 85.1*
**Fusion index (%)**	67.7 ± 12.9	47.8 ± 23.1*
**Myofiber area (μm)**	9478 ± 7164	6871 ± 4604*
**Myofiber diameter (**μm**)**	1.04 ± 0.57	0.89 ± 0.46*
Control: normal C2C12 cells, *n* = 3; siRNA‐ERK: ERK gene silencing C2C12 cells, *n* = 3; *p < 0.05 vs. Control group.		

**8-11**

**Effects of late‐life enalapril administration on cardiac mitochondria of old rats**

Riccardo Calvani^1^, Vito Pesce^2^, Giuseppe Sirago^2^, Anna Picca^1^, Christy S. Carter^3^, Guglielmina Chimienti^2^, Angela Maria Serena Lezza^2^, Roberto Bernabei^1^ and Emanuele Marzetti^1^

^1^ *Department of Geriatrics, Neurosciences and Orthopedics, Catholic University of the Sacred Heart School of Medicine, Teaching Hospital “Agostino Gemelli”, Rome, Italy;* ^2^ *Department of Biosciences, Biotechnologies and Biopharmaceutics, University of Bari, Bari, Italy;* ^3^ *Department of Aging and Geriatric Research, Institute on Aging, Division of Biology of Aging, University of Florida, Gainesville, FL, USA*

**Introduction:** Inhibition of the renin‐angiotensin system ameliorates age‐related mitochondrial alterations in several rat tissues and increases rodent lifespan. Here, we investigated the effect of late‐life enalapril administration on mitochondria biogenesis, mitochondrial dynamics, antioxidant defenses and mitochondrial DNA (mtDNA) content variations in the heart of old rats.

**Methods:** Fischer 344 × Brown Norway rats were randomly assigned to receive enalapril (*n* = 8), or placebo (*n* = 8) from 24 to 27 months of age. After sacrifice, the heart was collected, and total protein and DNA were purified.

**Results:** Enalapril induced a marked increase in mtDNA content as well as citrate synthase activity, a marker of mitochondrial mass. Accordingly, a greater content of mitochondrial biogenesis proteins [i.e., peroxisome proliferator‐activated receptor gamma coactivator 1‐alpha (PGC‐1α), and mitochondrial transcription factor A (TFAM)], mitochondrial fusion protein mitofusin 2 (Mfn2), mitochondrial antioxidant enzymes superoxide dismutase 2 (SOD2) and peroxiredoxin III (PrxIII) was found in the same group. Conversely, the expression of PGC‐1β, dynamin‐related protein 1 (DRP1) and oxidized PrxIII (Prx‐SO_3_) were unchanged between groups.

**Conclusions:** Our data indicate that enalapril enhances mitochondrial biogenesis in the heart of aged rats. As both mtDNA content and mitochondrial biogenesis are crucial for preserving cellular respiratory capacity, and PGC‐1 is protective against ROS production and oxidative damage, our results support the hypothesis that the beneficial effects of enalapril on the heart is mediated at least partly by mitigation of oxidative stress. The shift of mitochondrial dynamics in rats treated with enalapril suggests that enalapril administration may promote the dilution of mitochondrial damage along the network as a further cardioprotective effect.

**8-12**

**Synthetic Ghrelin agonists prevents muscle atrophy in an in vitro model of muscle wasting in GHSR‐independent manner**

Emanuela Agosti^2†^, Sara Clerici^1†^, Michele Ferrara^1^, Elia Angelino^1^, Nicoletta Filigheddu^2^, Claudio Pietra^3^ and Andrea Graziani^1,2^

^1^ *Dept. of Translational Medicine, University of Piemonte Orientale, Novara;* ^2^ *Dept. of Experimental Oncology, University Vita‐Salute San Raffaele, Milano;* ^3^ *Helsinn Healthcare SA, Lugano, Switzerland*

^†^SC and EA contributed equally.

**Introduction:** Acylated and unacylated ghrelin (AG and UnAG, respectively) are circulating peptide hormones generated in the stomach in response to fasting and caloric restriction. AG, through binding to its acylation‐selective receptor, GHSR1, induces strong GH release, and stimulates food intake, adiposity, and positive energy balance. In both patients and animal models, AG ameliorates cachexia induced by several pathological conditions through GHSR‐dependent mechanisms. However, recent evidence indicates that both AG and UnAG directly protect skeletal muscle from experimentally induced atrophy, independently of GHSR‐1a, thus providing the evidence for the existence of an alternative anti‐atrofic ghrelin receptor expressed in the skeletal muscle (Porporato *et al*. J. Clin. Invest. 2013). Anamorelin (ANAM), HM01 are specific GHSR1 agonists, with appetite‐enhancing and anabolic effects. Different clinical studies have highlighted ANAM ability to improve the cachectic state in cancer patients by increasing total body mass and food intake, while HM01 was reported to protect mice in a C26 cancer cachexia model.

**Methods and Results:** In here, we showed that ANAM and HM01 activate anti‐atrophic and hypertrophic signalling, thus inducing hypertrophy and protecting cultured myotubes in an in vitro model of IL6‐induced muscle wasting. In addition, the demonstration that ANAM and HM01 induce hypertrophy in myotubes from GHSR−/− mice provides the genetic evidence that their activity in the skeletal muscle is independent on GHSR. Finally, HM04, a GHSR antagonist, impairs the GHSR‐independent biological activity of both ANAM and HM01, as well as that one of AG and UnAG.

**Conclusions:** Altogether, these observations indicate that i) synthetic Ghrelin agonists, such as Anamorelin and HM01, are dual agonists acting both through GHSR, the canonic AG receptor, and the novel, yet unidentified AG/UnAG receptor, ii) the binding pocket of GHSR and the novel AG/UnAG receptor share some structural features, albeit GHSR binding requires Ghrelin acylation; iii) a direct anti‐atrophic activity in the skeletal muscle may contribute to the biological activity of synthetic Ghrelin agonists in vivo.

**8-13**

**Sildenafil modulates catabolic pathways and improves cardiac function in a model of cancer cachexia‐induced cardiomyopathy**

Vincenzo Musolino^1,2,3^, Cristina Carresi^1,2,3^, Micaela Gliozzi^1,2^, Francesca Bosco^1,2^, Saverio Nucera^1,2,3^, Miriam Scicchitano^1,2^, Delfina Tavano^1,2^, Ernesto Palma^1,2^, Carolina Muscoli^1,2,3^ and Vincenzo Mollace^1,2,3^

^1^ *Institute of Research for Food Safety & Health (IRC‐FSH), University of Catanzaro “Magna Graecia”, Catanzaro, Italy;* ^2^ *NUTRAMED S.c.a.r.l., Roccelletta di Borgia, Catanzaro, Italy;* ^3^ *IRCCS San Raffaele Pisana, Rome, Italy*

**Introduction:** Cachexia is a complex metabolic disorder occurring in late stages of chronic disease including cancer and characterized by involuntary weight loss caused by an ongoing wasting of skeletal muscle with or without loss of adipose tissue. Cachexia also affects the cardiac muscle. Heart atrophy is caused by the hyperactivation of the main cellular catabolic pathways, including autophagy and Ubiquitin Proteasome System (UPS). As a consequence of the atrophy of the heart cardiac function is impaired. Anti‐cachectic therapy in patients with cancer cachexia is so far limited to nutritional support. Sildenafil, a selective inhibitor of the enzyme phosphodiesterase‐5 (PDE5), has been shown to induce myocardial protective effects, to increase muscle protein synthesis and to reduce muscle fatigue. We hypothesized that sildenafil ameliorates the wasting process and the heart function in the Yoshida hepatoma tumor model.

**Study design:** In this study the effects of sildenafil were tested in cachectic tumour‐bearing rats (Yoshida AH‐130). Rats were treated daily with 30 mg/kg of sildenafil or vehicle for a period of 16 days. Body weight and composition were assessed at baseline and at the end of the study. Cardiac function was analyzed by echocardiography at baseline and at day 11.

**Results:** Treatment with 30 mg/kg/d of sildenafil protected the heart from general atrophy. Tumor‐bearing rats displayed cardiac dysfunction, as indicated by the significant impairment of the left ventricular ejection fraction and the left ventricular fractional shortening. In contrast, sildenafil improved cardiac dysfunction. Western blotting analysis showed an upregulation of the autophagy and of the UPS in the hearts of the vehicle‐treated tumour‐bearing rats. Treatment with Sildenafil, however, was able to modulate the UPS markers (e.g. TRAF6 and Atrogin‐1) in the hearts of tumour‐bearing rats. Moreover, cachexia led to an up‐regulation of eNOS and iNOS in the hearts of tumor‐bearing rats. Sildenafil was able to down‐regulate the expression of the enzymes. Although sildenafil did not reduce the loss of lean body mass, it showed a trend to protect from adipose tissue depletion.

**Conclusions:** Sildenafil treatment in the Yoshida hepatoma model showed an attenuation of fat tissue loss in animals with progressive weight loss in cancer cachexia. Moreover, the drug protects the heart from atrophy through a down regulation of the catabolic markers and presumably through a modulation of the NOS/NO pathway. This effect led to a significant improvement of cardiac function. While mounting evidence supports the implication of the NOS/NO pathway in muscle wasting, many questions remain unanswered. Larger studies with longer follow‐up and molecular analysis are required to verify whether sildenafil could ameliorate cardiac wasting and function by modulating inflammation and catabolic pathways.

**8-14**

**Musclin: an exercise‐induced myokine useful to contrast muscle wasting during cancer**

Andrea David Re Cecconi, Giulia Benedetta Martinelli, Sara Previdi, Sergio Marchini, Luca Beltrame and Rosanna Piccirillo

*Oncology Department, IRCCS‐Mario Negri Research Institute for Pharmacological Research, Milan, Italy*

**Introduction:** Physical activity extends life span of patients affected by certain types of cancer, also by contrasting the associated muscle wasting (i.e. cachexia). The most effective type of physical activity against muscle wasting during cancer seems to be aerobic exercise. So we asked whether it promotes secretion of proteins by muscles (i.e. myokines) that may contrast cancer cachexia.

**Methods:** To mimic aerobic exercise, we infected C2C12 myotubes with PGC1α‐expressing adenoviruses, because PGC1α is the main transcriptional coactivator involved in muscle adaptation during aerobic exercise.

**Results:** Our microarray analysis showed musclin as a PGC1α‐induced myokine. We further immunoprecipitated it only from supernatants of PGC1α expressing myotubes. By Q‐PCR, we found musclin expression unchanged in myotubes hypertrophying because of activated AKT (to mimic anaerobic exercise). Among other PGC1α‐induced myokines, we found only musclin strongly downregulated in cachectic muscles and plasma of C26‐bearing mice even at times when their body weights were not lost yet. Thus, we electroporated Tibialis Anterior (TA) of C26‐bearing mice with musclin‐encoding plasmids and found musclin to preserve fiber area.

Dexamethazone‐treated myotubes or FoxO3‐expressing myotubes undergo atrophy as measured by increased rates of proteolysis and MuRF1 induction. Unlike GFP, musclin was able to contrast the dexamethazone induced‐MuRF1 expression in Luciferase assays. Notably, musclin‐containing supernatants of PGC1α expressing myotubes restrained the FoxO3‐induced rates of long‐lived protein degradation.

**Conclusions:** Musclin is a myokine induced specifically by PGC1α, typically increased upon aerobic exercise and musclin overexpression is beneficial against muscle wasting during C26 growth or in atrophying myotubes. Overall, musclin would be a good drug option for cancer patients that cannot exercise and are at risk of developing cachexia.

**8-15**

**Skeletal muscle myotubes show decreased protein synthesis and viability after treatment with chemotherapy**

Francina J. Dijk^1^, Bram Dorresteijn^1^, Yvette Luiking^1,2^, Onno Kranenburg^3^, Marion Jourdan^1^ and Miriam van Dijk^1^

^1^ *Nutricia Research, Nutricia Advanced Medicial Nutrition, Utrecht, the Netherlands;* ^2^ *Center for Translational Research in Aging and Longevity, Department of Health and Kinesiology, Texas A&M University, USA;* ^3^ *Department of Surgical Oncology, Cancer Centre, UMC Utrecht, Utrecht, the Netherlands*

**Introduction:** Chemotherapy is a very non‐specific treatment that targets all highly proliferating cells like cancer cells, but also healthy cells including skeletal muscle cells. Cancer itself can cause muscle wasting (cachexia); however, limited information is available on the direct effect of chemotherapy on skeletal muscle. A combination of capecitabine (a pro‐drug of 5‐fluorouracil) and oxaliplatin is often used as a chemotherapy treatment for colorectal cancer patients. This study aimed at investigating the effects of 5‐fluorouracil and oxaliplatin, either alone or combined, on protein synthesis (MPS), cell‐viability and apoptosis/necrosis in a model for skeletal muscle (C2C12‐myotubes).

**Methods:** C2C12‐myotubes were incubated for 24 h with a concentration range of oxaliplatin (OX; 2–125 μM, *n* = 3), 5‐fluorouracil (5FU; 2–250 μM, *n* = 2), oxaliplatin and 5‐fluorouracil combined 1:1 (OXF; 2–250 μM, *n* = 3) or left untreated (control). Subsequently, after a 4 h serum‐ and leucine‐free period, MPS was measured by puromycin incorporation by the SUnSET method. In parallel, cell viability was measured by CellTiter‐Glo, apoptosis by Caspase‐Glo and necrosis by LDH‐release directly after chemotherapy incubation. Statistical analyses by ANOVA with post‐hoc LSD versus control.

**Results:** MPS was inhibited dose‐dependently by both OXF and OX from 15.6 μM (*P* < 0.0056). In contrast, 5FU only inhibited MPS at the highest dose of 250 μM (*P* < 0.0056). OXF decreased cell viability significantly from 31.3 μM, whereas OX decreased cell viability significantly from 15.6 μM. Both OX and OXF increased apoptosis and necrosis from 15.6 μM to 31.3 μM, respectively (*P* < 0.0056). 5FU decreased cell viability from 62.5 μM (*P* < 0.0056) and apoptosis only at 250 μM (*P* < 0.0056) while necrosis was unchanged.

**Conclusions:** Myotubes are compromised by chemotherapy treatment, with a decrease in protein synthesis and cell viability and increases in apoptosis and necrosis. The impact is chemotherapy and dose dependent. While the compromised effect of 5‐fluorouracil is evident at levels exceeding pharmacological doses, oxaliplatin is more toxic to myotubes than 5‐fluorouracil, even in low doses and given either alone or combined.

**8-16**

**Effects of formoterol on atrophy signaling, autophagy, and muscle phenotype in respiratory and limb muscles of rats with cancer‐induced cachexia**

Anna Salazar‐Degracia^1^, Sílvia Busquets^2,3^, Josep M. Argilés^2,3^, Esther Barreiro^1^ and Francisco J. López‐Soriano^2,3^

^1^ *Pulmonology Department‐Muscle Wasting and Cachexia in Chronic Respiratory Diseases and Lung Cancer Research Group, IMIM‐Hospital del Mar, Parc de Salut Mar, Health and Experimental Sciences Department (CEXS), Universitat Pompeu Fabra (UPF), Barcelona Biomedical Research Park (PRBB), Barcelona, Spain;* ^2^ *Cancer Research Group, Departament de Bioquímica i Biomedicina Molecular, Facultat de Biologia, Universitat de Barcelona, Barcelona, Spain;* ^3^ *Institut de Biomedicina de la Universitat de Barcelona (IBUB), Barcelona, Spain*

**Introduction and purpose:** Muscle mass loss and wasting are characteristic features of patients with chronic conditions including cancer. Beta‐adrenergic receptor agonists attenuate muscle wasting. We hypothesize that muscle atrophy signaling pathways and altered metabolism may be attenuated in cancer cachectic animals receiving a treatment with formoterol.

**Methods:** Rats bearing the Yoshida AH‐130 ascites hepatoma (7 days) were treated daily with formoterol (0.3 mg/kg body weight, subcutaneously).

**Results:** In diaphragm and gastrocnemius of cancer cachectic rats, fiber sizes were reduced, levels of structural alterations, atrophy signaling pathways, proteasome content, protein ubiquitination, autophagy, and myostatin were increased, while those of regenerative and metabolic markers (myoD, mTOR, and PGC‐1alpha) were decreased.

**Conclusions:** Treatment with formoterol attenuates the structural alterations and atrophy signaling, while improving other molecular perturbations. These results have relevant therapeutic implications, showing beneficial effects of formoterol in cachectic muscles through several key biological pathways.

**8-17**

**Fenofibrate prevents skeletal muscle loss in lung cancer‐induced cachexia**

Marcus D. Goncalves^1^, Seo‐Kyoung Hwang^1^, Chantal Pauli^2^, Charles J. Murphy^1^, Zhe Cheng^1^, Guoan Zhang^1^ and Lewis C. Cantley^1^

^1^ *Weill Cornell Medical Center, New York City, USA;* ^2^ *Institute for Pathology and Molecular Pathology*

**Introduction:** Cancer cachexia is a systemic metabolic disorder with high mortality characterized by catabolism of nutrient rich tissues with no effective treatment. This condition is particularly prevalent in non‐small cell lung cancer (NSCLC), where loss of skeletal muscle results in functional impairment and increased mortality. The aim of the current study was to assess the changes in systemic metabolism in a genetically engineered mouse model of NSCLC in an attempt to identify novel therapeutic targets.

**Methods:** NSCLC was induced in adult Kras^G12D/+^Lkb1^flx/flx^ (KL) mice with intranasal adenovirus containing Cre recombinase and the mice were followed for changes in body weight and food intake. Cachexia was a priori defined as a 10% loss in body weight from the peak weight obtained during the study. Serum and tissue metabolites, hormones, and cytokines involved in nutrient homeostasis in tumor‐bearing mice were measured and compared to fed and fasted mice using mass spectrometry and commercially available assays. RNA sequencing of the liver and skeletal muscle was performed to identify differential variables with adjusted p‐values below 0.05 and log‐transformed fold‐change greater than 1 between the subgroups of mRNA expression using DESeq2 (v1.14.1).

**Results:** Sixty percent of KL mice develop loss of skeletal muscle, loss of adipose tissue, and increased inflammatory markers mirroring the human cachexia syndrome. Using non‐cachexic and fasted animals as controls, we report a unique cachexia metabolite phenotype that includes the loss of PPARα‐dependent ketone production by the liver. In this setting, glucocorticoid levels are increased and correlate with reduced type 2 myofiber size and increased markers of hepatic gluconeogenesis. Restoring ketone production using the PPARα agonist, fenofibrate, reversed the metabolic changes in hepatic metabolism and prevented the loss of body weight and skeletal muscle.

**Conclusions:** This study characterized a new model of lung cancer induced cachexia and provides evidence for a novel therapeutic strategy for cachexia focused on hepatic ketone production using the well‐tolerated, generically available drug, fenofibrate.

**8-18**

**BGP‐15 co‐therapy protects against chemotherapy‐induced cachexia**

Dean G. Campelj^1,2^, Cara A. Timpani^1,2^, James C. Sorensen^1,2^, Craig A. Goodman^1,2,3^, Alan Hayes^1,2,3^ and Emma Rybalka^1,2,3^

^1^ *College of Health & Biomedicine, Victoria University, Melbourne, Australia;* ^2^ *Australian Institute for Musculoskeletal Science (AIMSS), Melbourne, Australia;* ^3^ *Institute of Sport, Exercise & Active Living (ISEAL), Victoria University, Melbourne, Australia*

**Introduction:** Chemotherapy‐induced muscle wasting and dysfunction has emerged as a contributing factor to cancer cachexia^1^, ^2^ and increases the risk of morbidity and mortality.^3^ Thus, developing strategies to protect and preserve skeletal muscle during anti‐cancer therapy is paramount. Here, we investigate the effects of irinotecan (IRI), a topoisomerase‐1 inhibitor used in the treatment of colorectal cancer, on skeletal muscle mass and function. Further, we have evaluated the pharmacological small molecule, BGP‐15, a poly‐ADP ribose polymerase‐inhibitor, to ameliorate IRI‐induced skeletal muscle pathology.

**Methods:** 6‐week old male Balb/C mice were treated with 6 intraperitoneal injections of either vehicle (0.1% DMSO), IRI (30 mg/kg) or IRI + BGP‐15 adjunct therapy (15 mg/kg) over two weeks. Through *in vivo* and *ex vivo* experiments, body composition, whole body metabolic and voluntary activity levels, contractile properties and mitochondrial function were investigated.

**Results:** IRI reduced lean mass (*p* < 0.05) and the absolute force production of the *soleus* (SOL; *p* < 0.0005) and *extensor digitorum longus* (EDL; *p* < 0.05) muscles. Remarkably, BGP‐15 co‐therapy was protective, attenuating the IRI‐induced loss of lean mass (*p* < 0.005) and absolute force production of both the SOL (*p* < 0.0005) and EDL (*p* < 0.05). In *flexor digitorum brevis* fibres, IRI (both alone and in combination with BGP‐15) increased the oxidative metabolic potential (*p* < 0.05), a marker of mitochondrial functional capacity, which was achieved by improving coupling efficiency (*p* < 0.05). BGP‐15 co‐therapy also improved the glycolytic metabolic potential (*p* < 0.05), a marker of anaerobic capacity. These metabolic data are consistent with a reduced whole body VO_2_ (ml·min^−1^·g^−1^) at rest (*p* < 0.05) in IRI‐treated mice and suggest induction of an “energy conservation” phenotype in the skeletal muscle.

**Conclusions:** Our study is the first to show that IRI treatment induces skeletal muscle dysfunction and wasting, which was attenuated with BGP‐15 co‐therapy. These findings highlight the potential of BGP‐15 to improve patient quality of life during and following anti‐cancer chemotherapy.

**8-19**

**Creatine supplementation prevents skeletal muscle atrophy by attenuating systemic pro‐inflammatory condition and protein degradation in tumor‐bearing rats**

Rafael Deminice^1^, Paola S. Cella^1^, Mayra J. Testa^1^, Fernando H. Borges^2^, Poliana C. Marinello^1,2^, Patricia C. Perandini^1^, Rubens Cechinni^2^, José A. Duarte^3^ and Flávia A. Guarnier^2^

^1^ *Department of Physical Education, State University of Londrina, Brazil;* ^2^ *Department of Pathology, State University of Londrina, Brazil;* ^3^ *CIAFEL, Faculty of Sport, University of Porto, Porto, Portugal*

**Aim:** To investigate the effects of creatine supplementation on skeletal muscle protein degradation signaling in Walker‐256 tumor‐bearing rats.

**Methods:** Thirty‐two male Wistar rats were assigned to four (*n* = 8) groups: control (C), creatine supplemented (Cr), tumor‐bearing (T) and tumor‐bearing creatine supplemented (TCr) rats. Creatine monohydrate was supplied in drinking water in a total of 21 days (8 g/L). Tumor implantation was performed on the eleventh day of creatine supplementation; rats were euthanized ten days after tumor cells inoculation. Tumor cells inoculation consisted of a suspension of Walker‐256 cells (7.0 × 10^7^cells).

**Results:** The progressive increase in tumor mass (9% of body weight at 10th day) coincided with lower (*P* < 0.05) body mass (−11%) and muscle wasting (−31% soleus CSA) in T compared to C group. In addition, tumor growth promoted elevated (*P* < 0.05) systemic leukocytes (1.2‐fold, *P* < 0.05), increased circulating inflammatory interleukins (TNF‐α 2.6‐fold and IL‐6 4.4‐fold increase, *P* < 0.05) and muscle oxidative stress demonstrated by increased MDA (54%) and decreased GSH/GSSG (−61%) ratio in T compared to C group. Tumor growth also promoted increased protein expression of MuRF1 and Atrogin1. In contrast, creatine supplementation mitigates body weight loss and muscle wasting (6% and 24% of attenuation in TCr group compared to T, respectively, *P* < 0.05). Tumor‐induced systemic pro‐inflammatory condition, muscle oxidative stress and elevated ubiquitin ligases were also attenuated by creatine supplementation. Therefore, decreased expression of MuRF1 and Atrogin1 mediated by the attenuation of pro‐inflammatory condition is thought to be responsible for the preventive effects of creatine supplementation on muscle loss. Mean life span was not different between groups (TCr 26.4 ± 2.2 vs T 22.0 ± 2.7, days).

**Conclusions:** Creatine supplementation prevents cachexia development and muscle wasting by attenuating tumor‐induced systemic pro‐inflammatory condition and muscle oxidative stress; condition that may down‐regulates important regulators of ubiquitin mediated protein degradation Atrogin‐1 and MuRF‐1 in skeletal muscle.

Supported by Capes‐PVE # 88881.068035/2014‐01

**8-20**

**Targeting mitochondria with SS‐31 in experimental cancer and chemotherapy‐induced cachexia**

Riccardo Ballarò^1,2^, Marc Beltrà^1,2^, Paola Costelli^1,2^, Hazel Szeto^3^ and Fabio Penna^1,2^

^1^ *Department of Clinical and Biological Sciences, Experimental Medicine and Clinical Pathology Unit, University of Turin, Italy;* ^2^ *Interuniversity Institute of Myology, Italy;* ^3^ *Mitochondrial Therapeutics Consulting, New York, NY, USA*

Cancer cachexia is a debilitating muscle wasting condition defined also as an energy‐wasting syndrome (1). Mitochondria play a central role, being the main energy source. Indeed, mitochondrial alterations and low intracellular ATP have been found in the skeletal muscle of cachectic animals (2).

The present study aimed at evaluating the effects of a mitochondrial‐targeted compound (SS‐31) on muscle wasting in C26‐bearing mice either under unrestricted tumor growth (C26; 14 days of tumor growth) or treated with chemotherapy (oxaliplatin + 5‐fluorouracil; C26 OXFU; 28 days of tumor growth).

Animals with unrestricted tumor growth exhibited a reduction of muscle mass, muscle strength and food intake. OXFU administration was able to double the lifespan of C26 mice, although resulted in exacerbated muscle wasting. SS‐31 administration counteracted body weight and food intake loss in C26 mice and, while borderline significantly protected from muscle wasting. Regarding mitochondrial markers, both C26 and C26 OXFU animals exhibited a reduction of PGC‐1α, Cytochrome c and SDH protein levels with no effect of SS‐31 administration. In C26 OXFU mice, also the SDH total activity and ATP content were reduced. In C26 mice SS‐31 increased SDH activity and ATP content. Mitochondrial alterations found in C26 mice also associated with a strong reduction in protein synthesis that was improved by SS‐31 treatment.

In conclusion, targeting mitochondria with SS‐31 exerts some beneficial effects on C26‐bearing mice with unrestricted tumor growth, partially protecting from body weight and food intake loss, muscle wasting and counteracting the impairment of muscle oxidative capacity leading to energy wasting. Further investigation and treatment optimization is required in order to demonstrate a potential effectiveness of SS‐31 in the more severe chronic model (C26 OXFU mice).

1. Argilés JM, Busquets S, Stemmler B, López‐Soriano FJ. Cancer cachexia: understanding the molecular basis. Nat Rev Cancer. Nature Publishing Group; 2014;14(11):754–62.
2. Pin F, Busquets S, Toledo M, Camperi A, Lopez‐Soriano FJ, Costelli P, *et al*. Combination of exercise training and erythropoietin prevents cancer‐induced muscle alterations. Oncotarget. 2015;6(41):43202–15.

**9-01**

**A double‐blind placebo controlled clinical trial into the physiological impacts of testosterone administration during resistance exercise training in older men**

Nima Gharahdaghi, Supreeth Rudrappa, Bethan Phillips, Iskandar Idris, Mathew Brook, Daniel J. Wilkinson, Nate Szewczyk, Kenneth Smith and Philip Atherton

*MRC‐ARUK Centre of Excellence and NIHR BRC, School of Medicine, University of Nottingham, UK*

**Introduction:** The andropause is associated with declines in serum testosterone and an associated loss of skeletal muscle mass and function (i.e. sarcopenia), and insulin resistance. Two of the major interventions purported to offset sarcopenia are testosterone (T) therapy and resistance exercise training (RET); however, the benefits of T coupled to RET are poorly defined—especially on the back drop of ‘anabolic resistance’ of ageing. The purpose of this study was to determine the effect of RET plus T (vs. placebo) in relation to body composition, muscle mass, strength and oral glucose tolerance.

**Methods:** Sixteen non‐hypogonadic healthy older men, 65‐75 y, BMI ≤30 kg·m^−2^ (serum total *T* > 8.3 nmol·l^−1^) were randomly assigned in a double‐blinded fashion to receive bi‐weekly: either placebo (*P*, *n* = 8) or testosterone (T; Sustanon 250‐mg, *n* = 8) injections over 6‐week whole‐body RET. RET consisted of leg extension, leg press, leg curl, lat pull down, shoulder press and bench press (3 sets, 8–10 reps at 80% 1‐RM). Dual energy x‐ray absorptiometry (DXA), muscle ultrasound, isokinetic dynamometry and oral glucose tolerance tests (OGTT) were used to quantify body composition, muscle architecture, maximal voluntary contraction (MVC) and glycaemic control, respectively.

**Results:** T adjuvant to RET, augmented total lean mass (5.29 ± 2.67% vs 1.18 ± 2.84%, *P* = 0.01, ES = 0.79), appendicular lean mass (24520 ± 2739 g to 26133 ± 3075 g vs. 24978 ± 3813 g to 25166 ± 3438 g, *P* = 0.023, ES = 0.42), m.vastus lateralis thickness (VL) (*P* < 0.0001, ES = 0.38), 1‐RM leg extension (*P* < 0.0002, ES = 0.61) and leg curl (*P* = 0.005, ES = 0.39). However, no group differences (*P* > 0.05) were observed in MVC or fascicle length/pennation angle. Additionally, T decreased body fat 6.40 ± 3.66% (*P* = 0.017, ES = 0.25) vs. P. There was no significant impact of T/RET in relation to OGTT (*P* > 0.05).

**Conclusions:** T therapy, adjuvant to RET, enhanced muscle mass (i.e. thus “overcoming” anabolic resistance), whilst simultaneously decreasing body fat without impacting glycaemic control. T therapy coupled to RET is an effective short‐term intervention to improve muscle mass/function in older age.

**9-02**

**SARA Clinical program for evaluating safety and efficacy of Sarconeos in a Phase 2b clinical trial**

Waly Dioh^1^, Carole Margalef^1^, Philippe Dupont^1^, Pierre Dilda^1^, René Lafont^2^, Stanislas Veillet^1^ and Susanna Del Signore^1^

^1^ *Biophytis, UMPC – BC9, 4 place Jussieu75005, Paris, France;* ^2^ *Sorbonne Universités, UPMC Univ Paris 06, CNRS ‐ Institut de Biologie Paris Seine (BIOSIPE), 75005, Paris, France*

**Introduction:** Sarcopenia is a key underlying cause of physical frailty, a reversible condition in older subjects, which may lead to mobility disability and dependency. Sarcopenia is characterized by the loss of muscle mass and function. Biophytis has developed the drug candidate Sarconeos (BIO101) acting via Mas, the Angiotensin1–7 receptor for prevention and treatment of Sarcopenia. Mas receptor is one of the components of the Renin‐Angiotensin System.

**Methods:** Specifically, the SARA clinical program was developed with Sarconeos (BIO101) in age‐related sarcopenia and is composed of three main studies:

- The completed SARA‐PK Phase 1 study that evaluated Sarconeos safety profile in young and elderly volunteers up to 1400 mg/day in single administration and up to 900 mg/day after 14‐day multiple oral administration.
- The ongoing SARA‐OBS observational study that aims to validate applicability of inclusion criteria based on sarcopenia definition according to the Foundation of NIH (Studenski *et al*., 2014) and a very low Short Physical Performance Battery (SPPB; Guralnik J *et al*., 1994). This study will also evaluate functional (6 minutes walking distance: 6MWD and 400 meters test) and three Patient Reported Outcomes (SF‐36; SarQoL, TSD‐OC) parameters.
- The SARA‐INT interventional phase 2 study that will evaluate safety and efficacy of two oral doses of Sarconeos versus placebo in older sarcopenic patients (scheduled for second semester 2017).

All data are hosted within the SARA dedicated platform, allowing real‐time monitoring.

**Results:** Our presentation will focus on 1) Sarconeos effects on a battery of pharmacodynamic plasma biomarkers (Myoglobin, PIIINP etc.) as observed in SARA‐PK. 2) Preliminary baseline data of SARA‐OBS on target population and primary (6 MWD/400 meters and Patient Reported Outcomes) and secondary endpoints. And, 3) SARA‐INT methodology for investigating Sarconeos safety and efficacy in sarcopenic older patients.

**Conclusions:** The SARA clinical program will allow to outline the effects of Sarconeos on age‐related sarcopenia.

**9-03**

**Neuromuscular electrical stimulation (NMES) during hemodialysis improves muscle mass and function in chronic renal disease patients**

Ana C. Marini^1^, Reika D. Motobu^1^, Bruna M. Giglio^1^, Renata C. Fernandes^1^, João F. Mota^1^, Fabio S. Lira^2^, Ana T.V.S. Freitas^1^, Alessandro Laviano^3^, Benjamin T. Wall^4^ and Gustavo D. Pimentel^1^

^1^ *Clinical and Sports Nutrition Research Laboratory (Labince), Nutrition Faculty (FANUT) – Federal University of Goias (UFG), Goiânia, GO, Brazil;* ^2^ *Immunometabolism Research Group, Department of Physical Education, São Paulo State University (UNESP), Presidente Prudente, SP, Brazil;* ^3^ *Department of Clinical Medicine, Sapienza University, Rome, Italy;* ^4^ *Department of Sport and Health Sciences, College of Life and Environmental Sciences, University of Exeter, Exeter, UK*

**Background and aims:** Renal failure patients undergoing hemodialysis experience significant loss of muscle mass and function, which in turn contributes to poor clinical outcome. Physical inactivity contributes to loss of muscle mass and function. NMES elicits muscle contraction and has been shown to attenuate muscle atrophy when physical activity is not feasible or tolerated. Therefore, we hypothesized that regular NMES during dialysis sessions may improve muscle mass and function.

**Methods**: Adult renal failure patients, receiving hemodialysis 3 times/week, were considered for the study. Patients were randomly assigned to usual care (control group) or to NMES. NMES was applied bilaterally to both thighs and calves, at the origin and insertion points of muscle for 40 min during each hemodialysis session for 1 month. Skeletal muscle mass index (SMI; g/m^2^), muscle function and performance were assessed at the beginning and at the end of the study period by anthropometry, BIA, handgrip strength (Kg) and gait speed (m/sec). Results were statistically analyzed by Student's t‐test, and data are reported as M ± SD.

**Results**: Twenty‐one patients were studied (*x* = 8 women, *x* = 13 male; 45.8 ± 10.6 yr). In NMES‐treated patients (*n* = 10; 6 M/4F), SMI increased significantly at the end of the study when compared to control group (+0.35 kg/m^2^ vs. −0.41 kg/m^2^, respectively; *p* = 0.007). Gait speed improved in NMES‐treated patients at the end of the study (+0.07 m/s; *p* = 0.04), whereas it remained unaltered in the control group (−0.02 m/s; *p* = 0.70). At the end of the study period, no significant change in handgrip strength was observed in NMES and control groups (*p* = 0.28 and *p* = 0.08, respectively).

**Conclusions**: Thirty days of NMES targeting muscles of the legs may improve muscle mass and function in patients with chronic renal failure and undergoing hemodialysis.

**10-01**

**Changes in muscle quality following 12 weeks of power training in mobility‐limited older adults: a randomized controlled trial**

Jonathan Vaarst^1^, Mikkel Kolind^1^, Lars N. Hvid^2^, Marianne Andersen^3^ and Paolo Caserotti^1^

^1^ *Department of Sports Science and Clinical Biomechanics, SDU Muscle Research Cluster (SMRC), University of Southern Denmark, Odense, Denmark;* ^2^ *Section for Sport Science, Department of Public Health, Aarhus University, Aarhus C, Denmark;* ^3^ *Department of Endocrinology, Odense University Hospital, Odense, Denmark*

**Introduction:** Aging is associated with a loss of muscle strength that exceeds the loss of muscle mass, which ultimately translates into reduced muscle quality (MQ: strength per unit of muscle mass). The decline in muscle strength is greater in mobility‐limited older adults compared to well‐functioning older adults, and MQ may be even poorer in this group. There is evidence that power training counteracts the loss of function, although it is unclear to which extent this exercise regime affects muscle mass, strength and quality, especially in mobility‐limited older adults. This study investigated the effect of 12 weeks of progressive high‐intensity (70–80% of 1 RM) power training on muscle quality in mobility‐limited participants aged 75+.

**Methods:** Sixty‐five older adults with usual gait speed <0.9 m/s (age: 82.4 ± 4.8(mean ± SD), 66.2% female) were randomized to a control group (CG) or training group (TG). Fifty‐one participants had complete cases (CG: *n* = 27, age 82.8 ± 5.2, 77.8% women); (TG: *n* = 24, age 82.6 ± 4.5, 50% women). Muscle strength was assessed as an isometric single‐leg maximum voluntary contraction (MVC) normalized to body weight (N/kg). Leg muscle mass (LMM) of the dominant leg was determined using dual‐energy X‐ray absorption (DXA – Hologic Inc, Bedford, MA). MQ was calculated as MVC divided by LMM.

**Results:** Muscle strength increased by 2.6 ± 2.9 N/kg (27.2%) in the TG with no changes in the CG (TG vs CG; *p* < 0.01). LMM did not change in either group. MQ increased by 28.6 ± 32.0 N/kg_LMM_ (27.9%) in the TG with no changes in the CG (TG vs. CG; p < 0.01).

**Conclusions:** The results indicate that progressive power training is effective in improving MQ in mobility‐limited older adults and that these changes primarily are mediated by increases in muscle strength with no changes in muscle mass.

**10-02**

**Physical mobility and survival in older Chileans**

Cecilia Albala^1^, Lydia Lera^1^, Barbara Angel^1^, Carlos Marquez^1^, Rodrigo Saguez^1^, Florencia Danioni^3^, Patricia Arroyo^2^ and Felipe Salech^2^

^1^ *INTA, Universidad de Chile, Santiago, 7640765, Chile;* ^2^ *Clinical Hospital Faculty of Medicine, Universidad de Chile, Chile;* ^3^ *Medical student Faculty of Medicine, Universidad de Chile, Chile*

**Background:** Physical Mobility is a key issue for maintaining independence in older people.

**Objective:** To study the association of self‐reported physical mobility, defined as ability to walk eight blocks and leisure time physical activity (LTPA) practice, with survival in older Chileans.

**Methods:** Follow up of the Alexandros cohorts designed to study disability associated with obesity in community‐dwelling people 60 y and older living in Santiago/Chile. At baseline 2395 participants, mean age 69.6 ± 7.1 y (min 60; max 100), 67.1% women, underwent home interviews including history of chronic diseases, self‐reported disability/functional limitations, ability to walk 8 blocks and LTPA were performed. Anthropometry, dynamometry and physical performance were measured. Information about deaths was available for the whole sample. Cox proportional hazard models and Survival probabilities according ability to walk eight blocks and LTPA at baseline were built.

**Results:** At baseline 62% of the participants reported no limitations in walking eight blocks, 30.3% some limitations and 7.7% inability. Only 10.8% reported LTPA at least 3 times per week with 74.2% of the sample never practicing. After 10–14 years of follow up and 24459 person/years at risk 469 people were died. After adjusting by age, sex, BMI, smoking and education, the hazard ratios for death associated with limitation for walking 8 blocks was HR = 1.84 (95%CI:1.49–2.28) and the protective effect of practicing physical activity ≥3 times/week was HR = 0.04 (95%CI:0.006–0.305).

The probability of survival at 12–14 y according ability to walk eight blocks was 0.84 (95%CI 0.82–0.86) for people with no limitations and 0.62(95%CI 0.58–0.66) for people with limitations/inability.

For people practicing LTPA ≥3 times/week the probability of survival at 12–14 y was 0.988 (0.9633–0.9961) vs. 0.748 (0.6987–0.7907) for people practicing LTPA 1–2 times/week or never.

**Conclusions:** Ability to walk 8 blocks and physical activity ≥3 times per week are independently associated with higher survival in older Chileans.

**Funding:** Fondecyt Grants 1080589 & 1130947

**10-03**

**Concurrent training does not impair myonuclei addition in elderly**

Miguel Conceição^1,3^, Claudia Cavaglieri^1^, Cleiton Libardi^2^, Felipe Vechin^3^, Mara Patricia Chacon‐Mikahil^1^, Aline Bacurau^3^, Patricia Chakur Brum^3^ and Carlos Ugrinowitsch^3^

^1^ *University of Campinas, SP, Brazil;* ^2^ *Federal University of São Carlos, SP, Brazil;* ^3^ *University of São Paulo, SP, Brazil*

**Introduction:** It is well known that aging is associated with sarcopenia and cardiovascular problems. To reduce the burden, elderly should perform both resistance training (RT) and endurance training (ET), called concurrent training (CT). However, CT can hamper muscle strength and hypertrophy gains compared to RT alone, phenomenon known as interference effect. Interestingly, the physiological mechanisms behind interference effect are uncertain. Recently, it was showed the importance of satellite cells (SC) to muscle hypertrophy. After activation, SC enter the cell cycle, proliferate and may fuse and donate their nuclei to existing muscle fibres. The donation of myonuclei is nedeed when increased transcription capacity is required, such as to promote protein accretion. Nevertheless, no study has investigated changes in myonuclei number after CT, in elderly.

**Objective:** To compare myonuclei number after 12 weeks of CT and RT in elderly.

**Methods:** 24 healthy subjects (64 ± 4 years, 64 ± 4 kg, 1.64 ± 0.11 m) were randomly allocated in one of the following groups: concurrent training (CT, *n* = 8), resistance training (RT, *n* = 9) or control group (GC, *n* = 7). A muscle biopsy was undertaken before and 48 after the last training session. Maximum strength test (1‐RM), maximal oxygen uptake (VO2max) test and magnetic resonance imaging (MRI) were also assessed pre‐ and post‐training. RT was performed twice a week in the leg press 45° (4 × 10 at 70–80% of 1‐RM). CT performed the same RT protocol followed by walking/running at 60–85% of VO2max during 40–50 min.

**Results:** CT and RT significantly increased muscle strength and hypertrophy after intervention with no difference between them. There were no changes in myonuclei number in all groups.

**Conclusions:** 12 weeks of CT increase muscle strength and hypertrophy similarly to RT. Also, neither CT nor RT showed myonuclei addition suggesting that pre‐existing myonuclei induced muscle growth.

**10-04**

**Leisure‐time physical activity at moderate and high intensity is associated with parameters of body composition, muscle strength and sarcopenia in aged population from the PREDIMED‐PLUS study**

Nuria Rosique‐Esteban^1,2^, Nancy Babio^1,2^, Andrés Díaz‐López^1,2^, Dora Romaguera^2,3^, J. Alfredo Martínez^2,4^, Vicente Martin Sanchez^5^, Helmut Schröder^2,6^, Ramón Estruch^2,7^, Josep Vidal^8^, Pilar Buil‐Cosiales^2,9^, Jadwiga Konieczna^2,3^, Itziar Abete^2,4^ and Jordi Salas‐Salvadó^1,2^

^1^ *Human Nutrition Unit, University Hospital of Sant Joan de Reus, Department of Biochemistry and Biotechnology, Pere Virgili Institute for Health Research, Rovira i Virgili University, Reus, Spain;* ^2^ *CIBER de Fisiopatología de la Obesidad y la Nutrición (CIBEROBN), Instituto de Salud Carlos III, Madrid, Spain;* ^3^ *Instituto de Investigación Sanitaria de Illes Balears (IdISBa), University Hospital of Son Espases, Palma de Mallorca, Spain;* ^4^ *Department of Nutrition, Food Sciences, and Physiology, Center for Nutrition Research, University of Navarra, Pamplona, Spain. IDISNA Navarra's Health Research Institute, Pamplona, Spain. IMDEA Food Institute, Madrid, Spain;* ^5^ *Grupo de Investigación en Interacciones Gen‐Ambiente y Salud, Universidad de León, León, Spain; CIBER Epidemiología y Salud Pública (CIBERESP), Madrid, Spain;* ^6^ *Cardiovascular Risk and Nutrition Research Group (CARIN), IMIM (Hospital del Mar Medical Research Institute), Barcelona, Spain. CIBER Epidemiology and Public Health (CIBERESP), Instituto de Salud Carlos III, Madrid, Spain;* ^7^ *Department of Internal Medicine, Hospital Clinic, Institut d'Investigació Biomèdica August Pi i Sunyer (IDIBAPS), University of Barcelona, Barcelona, Spain;* ^8^ *Department of Endocrinology and Nutrition, Hospital Clínic, Barcelona, Spain; CIBER de Diabetes y Enfermedades Metabólicas Asociadas (CIBERDEM), Instituto de Salud Carlos III, Madrid, Spain;* ^9^ *Servicio Navarro de Salud‐Osasunbidea Primary Health Care, Navarra, Spain; Navarra Institute for Health Research, Pamplona, Spain*

**Introduction:** Aging‐related changes in body composition and muscle strength, together with sarcopenia prevalence, are greatly influenced by physical activity (PA) and sedentary behaviors (SB). However, these associations have been barely explored in elder adults at high cardiovascular risk. We examined the associations of leisure‐time PA and SB with sarcopenia prevalence, body composition and muscle strength in elder adults with overweight/obesity and metabolic syndrome from the PREDIMED‐PLUS trial.

**Methods:** Cross‐sectional baseline analysis including 1,539 men and women (65 ± 5y). Sarcopenia was defined as low muscle mass (lowest sex‐specific tertile for skeletal muscle mass index) plus low muscle strength (lowest sex‐specific tertile for 30‐second chair‐stand test). We applied multivariable‐logistic regression for the associations of self‐reported leisure‐time PA and SB with sarcopenia; and multivariable‐linear regression for the associations with DXA‐derived bone mass, fat mass, lean mass and lower‐limb muscle strength.

**Results:** Inverse associations were observed between sarcopenia and each hourly increment in total [odds ratio 0.76 (95% confidence interval, 0.63, 0.94)], moderate [0.74 (0.57, 0.97)], vigorous [0.44 (0.23, 0.85)], and moderate‐vigorous PA (MVPA) [0.68 (0.53, 0.87)]. Incrementing 1 h/day total‐PA and MVPA was inversely associated with BMI, waist circumference (WC), fat mass, and positively associated with bone mass and lower‐limb muscle strength (all *P* < .05). One h/day increase in total SB, screen‐based SB and TV‐viewing was positively associated with BMI, WC and fat mass. Light‐PA was not significantly associated with any outcome.

**Conclusions:** Total‐PA and PA at moderate and high intensities may protect against the prevalence of sarcopenia, have a beneficial role on body composition and prevent loss of muscle strength. SB, particularly TV‐viewing, may have detrimental effects on body composition in elderly adults at high cardiovascular risk.

**10-05**

**Lean mass is better predictor of bone mineral density than physical fitness in elderly people**

Alba Gómez‐Cabello^1,2,3,4,5^, Alejandro Gómez‐Bruton^2,3,6^, Alejandro González‐Agüero^2,3,4,7^, Lucía Sagarra‐Romero^8,9^, José A. Casajús^2,3,4,7^, Ignacio Ara^5,9^ and Germán Vicente‐Rodríguez^2,3,4,7^

^1^ *Centro Universitario de la Defensa, Zaragoza, Spain;* ^2^ *GENUD “Growth, Exercise, NUtrition and Development” Research Group, University of Zaragoza, Zaragoza, Spain;* ^3^ *Centro de Investigación Biomédica en Red de Fisiopatología de la Obesidad y Nutrición (CIBERObn), Spain;* ^4^ *Instituto Agroalimentario de Aragón (IA2);* ^5^ *Centro de Investigación Biomédica en Red de Fragilidad y Envejecimiento Saludable (CIBERFES), Spain;* ^6^ *Universidad Isabel I, Burgos, Spain;* ^7^ *Faculty of Health and Sport Science (FCSD), Department of Physiatry and Nursing, University of Zaragoza, Ronda Misericordia 522001, Huesca, Spain;* ^8^ *VALORA Research Group, Universidad San Jorge, Spain;* ^9^ *GENUD Toledo Research Group, Universidad de Castilla‐La Mancha, Toledo, Spain*

**Introduction:** Within the older population, osteoporosis is a critical age‐related disorder because of the bone loss that occurs during the ageing process (1). A better understanding of the factors underlying increased bone fragility in elderly will lead to the definition of appropriate screening and diagnostic strategies.

**Purpose:** The aims of this study were to examine the association of bone mineral density (BMD) with some of the most widely used physical fitness tests and lean mass and to determine the differences in bone mass by fitness status in elderly.

**Methods:** Lean mass and BMD (subtotal body, lumbar spine and femoral neck) were measured with dual energy x‐ray absorptiometry in 204 elderly (144 women) aged 73.4 ± 5.6 years from Zaragoza (Spain). Physical fitness was evaluated with the Senior Fitness Test battery and 2 additional tests (static balance and 30‐meter walking speed). The association between physical fitness‐related variables and lean mass with BMD variables was tested by linear regression adjusting by age and sex. Finally, physical fitness of osteoporotic participants was compared to that of non‐osteoporotic with age and sex adjusted ANCOVAs.

**Results:** Physical fitness‐related variables did not significantly predict BMD, while lean mass significantly predicted BMD in all measured sites (4% for the femoral neck, 9% for the lumbar spine and 6% for the subtotal body; all *p* < 0.05). Moreover, no differences were found for physical fitness variables between osteoporotic and non‐osteoporotic participants. Nevertheless, non‐osteoporotic participants presented higher subtotal lean (39.30 ± 4.33 vs. 36.73 ± 4.27 kg), arms lean (3.91 ± 0.57 vs. 3.82 ± 0.56 kg) and legs lean (12.91 ± 1.59 vs. 12.05 ± 1.56 kg) masses than osteoporotic participants (all *p* < 0.05).

**Conclusions:** BMD is associated to lean mass and not physical fitness in elderly people. Therefore, BMD may be more susceptible to changes in lean mass than to changes in physical fitness.

**Acknowledgements:** The elderly EXERNET multi‐centre study has been supported by IMSERSO (104/07 and 147/2011), University of Zaragoza (UZ 2008‐BIO‐01), Centro Universitario de la Defensa de Zaragoza (UZCUD2016‐BIO‐01), Ministerio de Economía, Industria y Competitividad (DEP2016‐78309‐R), Biomedical Research Networking Center on Frailty and Healthy Aging (CIBERFES) and FEDER funds from the European Union (CB16/10/00477). The authors are also grateful to all the volunteers and to the Community Center for Seniors Pedro Laín Entralgo (Zaragoza), whose cooperation and dedication made this study possible.

References:1

Gomez‐Cabello
A
, 
Vicente Rodriguez, G
, 
Vila‐Maldonado, S
, 
Casajus, JA
, 
Ara, I
. [Aging and body composition: the sarcopenic obesity in Spain]. Nutr Hosp
2012;27: 22–30.2256630110.1590/S0212-16112012000100004

**10-06**

**Patients with established cancer cachexia lack the motivation and self‐efficacy to undertake regular structured exercise**

David Wasley^1^, Nichola Gale^2^, Sioned Roberts^2^, Karianne Backx^1,3^, Annmarie Nelson^4^, Robert van Deursen^2^ and Anthony Byrned^4^

^1^ *Cardiff Metropolitan University, Cardiff School of Sport, Cyncoed Campus, Cardiff, UK;* ^2^ *Cardiff University, School of Healthcare Sciences, College of Biomedical and Life Sciences, Heath Park, Cardiff, UK;* ^3^ *Cardiff Centre for Exercise & Health, Cardiff Metropolitan University, Cardiff School of Sport, Cyncoed Campus, Cardiff, UK;* ^4^ *Marie Curie Palliative Care Research Centre (MCPCRC), Division of Population Medicine, School of Medicine, Cardiff University, Heath Park, Cardiff, UK*

**Background:** Patients with advanced cancer frequently suffer a decline in activities associated with involuntary loss of weight and muscle mass (cachexia). This has profound effects on function and quality of life. Although exercise participation can maintain physical and psychological function in patients with cancer, uptake is low in cachectic patients who are underrepresented in exercise studies. To understand how such patients' experiences influence exercise participation, we investigated exercise history, self‐confidence, and exercise motivations in patients with established cancer cachexia, and relationships between relevant variables.

**Methods:** Lung and gastrointestinal cancer outpatients with established cancer cachexia (*n* = 196) completed a questionnaire set exploring exercise history and key constructs of the Theory of Planned Behaviour relating to perceived control, psychological adjustment and motivational attitudes.

**Results:** Patients reported low physical activity levels and few undertook regular structured exercise. Exercise self‐efficacy was very low with concerns it could worsen symptoms and do harm. Patients showed poor perceived control and a strong need for approval but received little advice from healthcare professionals. Preferences were for low intensity activities, on their own, in the home setting. Logistical regression analysis revealed no significant factors related to the independent variables.

**Conclusions:** Frequently employed higher intensity, group exercise models do not address the motivational and behavioural concerns of cachectic cancer patients in this study. Developing exercise interventions which match perceived abilities and skills are required to address challenges of self‐efficacy and perceived control identified here. Greater engagement of health professionals with this group in exploring potential benefits of exercise is required.

**10-07**

**Sleep characteristics are associated with physical functioning during acute hospitalization and predict functional recovery following discharge in older adults**

Rachel R. Deer^1^, Elena Volpi^1^ and Sara Nowakowski^2^

^1^ *Sealy Center on Aging, University of Texas Medical Branch, Galveston, TX, USA;* ^2^ *Department of Obstetrics & Gynecology, University of Texas Medical Branch, Galveston, TX, USA*

**Introduction:** Poor sleep quality, frequent in older adults, is associated with reduced physical function and well‐being. However, little is known about the relationship between sleep quality and the recovery of physical function after hospitalization. Thus, we conducted this study to examine the association between sleep quality and functional recovery after an acute hospitalization in community dwelling older adults.

**Methods:** Older adult patients (*N* = 27, mean age = 74 ± 8 years) were recruited during hospitalization (average length of stay 3.9 days) with Cardiovascular (56%), Pulmonary (22%), or Metabolic (13%) admission diagnosis. Testing was performed prior to discharge (baseline) and 1‐month post‐discharge. Functional performance was measured using the Short Physical Performance Battery (SPPB) consisting of three parts: gait, balance and chair rise. Sleep quality was measured using Pittsburgh Sleep Quality Index (PSQI) global score. Sleep efficiency and minutes awake after sleep onset was assessed using actigraphy (Actiwatch 2).

**Results:** Pearson correlations revealed significant associations between PSQI and baseline SPPB total or baseline gait scores. Separate regression models revealed baseline PSQI score predicted change scores from baseline to 1‐month post‐discharge for balance or gait; with a trend for SPPB total scores. Pearson correlations revealed significant associations between sleep efficiency and baseline balance, baseline SPPB total, SPPB total change, SPPB total percent change, balance change, balance percent change, or gait percent change. Minutes awake after sleep onset was negatively associated with SPPB percent change score or gait percent change. Regression found that higher sleep efficiency predicted greater percent change in SPPB score and higher minutes awake after sleep onset predicts lower SPPB percent change score.

**Conclusions:** For older adults, actigraphy‐assessed sleep efficiency and subjectively measured poorer sleep quality is associated with worse physical functioning during acute hospitalization. Sleep efficiency, minutes awake after sleep onset and baseline sleep quality also predicted recovery of physical functioning following hospitalization. These results suggest that interventions to improve sleep might help enhance functional recovery from hospitalization and increase physical function levels.

**10-08**

**Resistance training improves lean mass and function in hemodialysis patients**

Lorena Cristina Curado Lopes^1^, João Felipe Mota^1^, Jonato Prestes^2^, Raquel Machado Schincaglia^1^, Débora Mendes Silva^1^, Nayara Pedatella Queiroz^1^, Ana Tereza Vaz de Souza Freitas^1^ and Maria do Rosário Gondim Peixoto^1^

^1^ *Clinical Nutrition and Sports Research Laboratory (LABINCE), Faculty of Nutrition, Federal University of Goias, Brazil;* ^2^ *Post Graduation Program on Physical Education, Catholic University of Brazilia (UCB), Brazil*

**Introduction:** Chronic kidney disease (CKD) patients on hemodialysis present a reduction in muscle mass and functional capacity. These reductions are associated with greater mortality risk and impairments in quality of life (QoL). Resistance training (RT) is a viable strategy to attenuate muscle wasting and loss of strength. The aim of this study was to investigate the effect of two distinct intradialytic RT protocols on body composition, sarcopenia prevalence, functional capacity and QoL in CKD patients.

**Methods:** The study consisted of a 12‐week randomized clinical trial that enrolled 50 participants (age 54.6 ± 11.6) divided into three groups: high‐intensity intradialytic RT (HIG, *n* = 14); moderate‐intensity intradialytic RT (MIG, *n* = 16) and control (CG, *n* = 20). Body composition was assessed by dual‐energy X‐ray absorptiometry, functional capacity by the Short Physical Performance Battery score, and QoL by Kidney Disease Quality of Life Instrument.

**Results:** After the training period leg lean mass was higher in HIG compared with CG [*P* = 0.040; large effect size (ES) = 0.92]. HIG also displayed improvements in QoL in pain and physical function domains (P = 0.04 for both). Skeletal muscle index was increased in both RT protocols (*P* = 0.007; ES = 1.16 and 0.81 for HIG and MIG, respectively), and functional capacity (*P* = 0.002; ES = 0.33 and 0.52 for HIG and MIG, respectively) when compared to CG.

**Conclusions:** High‐intensity intradialytic RT was associated with gains in lean mass and QoL compared with lower‐intensity while functional capacity and appendicular muscle mass were improved, regardless of RT intensity.

**10-09**

**Intramyocellular and extramyocellular lipid, and rVO2 changes following vitamin D repletion and aerobic training in aged individuals**

D. Travis Thomas, Maja Redzic, Mingjun Zhao, David M. Schnell, Hideat Abraha and Guoqiang Yu

*University of Kentucky, Lexington, KY, USA*

**Introduction:** Intramyocellular (IMCL) and extramyocellular lipid (EMCL) is associated with muscle metabolic dysfunction. The combined effect of vitamin D and exercise on muscle lipid has not been investigated. We compared the magnitude of changes in myocellular lipid stores and local muscle oxygen consumption following combined treatment of vitamin D and aerobic training (DAT) compared with vitamin D alone (D), aerobic training alone (AT), and control (Ctl).

**Methods:** Subjects (>60 YO) with vitamin D insufficiency (25(OH)D < 32 ng/mL) were randomized to a double blinded design. Vitamin D3 (10,000 IU × 5 days/week) or placebo was provided for 12 weeks with 1 additional week (7 consecutive days) of aerobic training or no training. Gastrocnemius IMCL and EMCL were measured with magnetic resonance spectroscopy and fat segmentation. Hybrid near‐infrared and diffuse correlative spectroscopy measured local tissue blood flow, oxygen saturation, and VO2 during and following (recovery) a gastrocnemius fatiguing protocol. All measurements were completed at week 0 and at end‐study.

**Results:** Fifteen men and 16 women completed all measures. Mean age and BMI were 68 ± 6 YO and 25.5 ± 3 kg/m2, respectively. Mean 25(OH)D increased significantly in subjects receiving vitamin D (41 ± 4 ng/mL) vs. placebo (7 ± 6 ng/mL) (*p* < 0.05). No group or group by time differences were observed with EMCL (*p* > 0.05). DAT trended (*p* = 0.103) toward the greatest change in IMCL and corresponded to a 18% increase in rVO2 during full exercise recovery in DAT compared to a 2%, 11%, and 18% reduction in AT, D, and Ctl, respectively.

**Conclusions:** Vitamin D, when combined with AT, may potentiate the metabolic benefits of exercise by reducing IMCL and altering tissue‐level VO2. Future work will test the hypothesis that vitamin D promotes muscle lipid availability for β‐oxidation in response to exercise, thereby preventing lipotoxicity, and improving muscle anabolic sensitivity in populations at risk for sarcopenia and cachexia.

Supported by NIH grants R21AG046762, UL1TR000117, and T32DK007778‐16

**10-10**

**Influence of serum testosterone, insulin‐like growth factor 1 (IGF‐1), insulin‐like growth factor‐binding protein‐1 (IGFBP‐1) and 3 (IGFBP3) on muscle strength gain after 6 months of progressive resistance training in older subjects**

Antonio Paoli^1^, Tatiana Moro^1,2,3^, Antonino Bianco^4^, Mario Plebani^5^ and Giuseppe Marcolin^1^

^1^ *Department of Biomedical Sciences, University of Padova, Padova, Italy;* ^2^ *Department of Nutrition and Metabolism, University of Texas Medical Branch, Galveston, TX, USA;* ^3^ *Sealy Center on Aging, University of Texas Medical Branch, Galveston, TX, USA;* ^4^ *Sport and Exercise Sciences Research Unit, University of Palermo, Palermo, Italy;* ^5^ *Department of Medicine (DiMed), University of Padova, Padova, Italy*

 

**Introduction:** Loss of strength is an important contributor to disability and poor quality of life in the elderly. Resistance training (RT) is one of the main treatments suggested to reduce strength and muscle loss during aging process. Even though basal hormonal levels could play a pivotal role in determining elderly's muscle mass (Stilling et al., 2017) there is no agreement about the real role of basal hormonal levels on RT exercise's response.

**Methods:** We sough to investigate if basal hormonal levels influence strength improvement after 6 months of progressive RT in a group of elderly subjects. We analysed 23 subjects (14 men M and 9 women F), 62.4 ± 3.1 years. We tested lower limb strength on leg extension before after 6 months of training. We analysed free fat mass, total testosterone (tT), free testosterone (fT), IGF‐1, IGFBP‐1, and IGFBP3 before and after the training period.

**Results:** No correlation was found between tT, fT, IGF‐1, IGFBP‐1 and IGFB3 and free fat mass considering M and F or merged. Moreover, no correlation was found between fT, tT and strength increase considering men and women. No correlation was found between IGF‐1 and strength. We found a positive, significant correlation between strength improvement (p < 0.05, *r* = 0.435) and basal IGFBP1 concentration considering men and women merged and a significant negative correlation between IGFBP3 and strength gains in female (*P* = 0.006; *r* = 0.791).

**Conclusions:** Our results are suggestive of a more complex relationship between androgens, IGF1, IGFBPs and the response to 6 months of RT. Whilst a negative correlation between IGFBP1 and muscle mass was found in older woman (Stilling *et al*., 2017) our results suggest instead that higher serum IGFBP1 is related to a better RT response in the elderly (irrespective to gender) whilst higher IGFBP3 is a predictive factor for a poor strength increase in older women.

**10-11**

**Low‐intensity blood flow restriction training in combination with protein supplementation: a new approach for the prevention and treatment of sarcopenia**

Christoph Centner^1^, Denise Zdzieblik^1^, Steffen Oesser^2^, Albert Gollhofer^1^ and Daniel Koenig^1^

^1^ *Department of Sport Science, University of Freiburg, Germany;* ^2^ *CRI, Collagen Research Institute GmbH, Kiel, Germany*

Preservation of skeletal muscle mass and strength is of vital importance in older age for maintaining musculoskeletal and functional capacity and thus independence. Recent research has indicated that high‐intensity resistance training (RT) is an effective strategy to counteract the age‐related decline of muscle mass and function. According to the American College of Sports Medicine training loads of 70–85% of each individual's one repetition maximum (1RM) are necessary to produce substantial muscle growth. However, mainly due to comorbidities such as coronary heart diseases, diabetes and joint or muscle impairments, many elderlies are not capable of lifting heavy loads. Hence, there is an increasing need of identifying more suitable training modalities that combat sarcopenia and other causes of muscular atrophy, particularly in aged people with serious concomitant diseases. Recently, several studies have demonstrated that low‐intensity exercise (20–40% 1RM) combined with partial vascular occlusion elicits muscular hypertrophy to a similar extent to what is typically seen after high‐intensity resistance training. Although the effectiveness blood flow restriction (BFR) training has been well investigated, no study to date has combined this training method with an additional nutritional intervention. Besides an optimal training stimulus, an adequate protein intake has been shown to stimulate protein synthesis and thus increasing muscle mass and strength. Previous studies from our research group have demonstrated that collagen peptides enhance the effects of a 12‐week RT regarding its influence on muscle mass and fat reduction. Therefore, we hypothesize that BFR training in combination with a protein supplementation is a promising alternative in order to prevent and treat sarcopenia, especially in populations for whom high‐intensity exercise programs are contraindicated. We will present the method of BFR training and additionally discuss possible explanations for improved muscle mass and function by this concept.

**10-12**

**Circulatory levels of oxidative stress markers and BMD in postmenopausal women**

Fawaz Azizieh^1^, Khaled Al Jarallah^2^, Dia Shehab^2^, Renu Gupta^3^ and Raj Raghupathy^4^

^1^ *Department of Mathematics and Natural Sciences, International Centre for Applied Mathematics and Computational Bioengineering, Gulf University for Science and Technology, Kuwait;* ^2^ *Departments of Medicine, Kuwait;* ^3^ *Radiology, Faculty of Medicine, Kuwait University, Kuwait;* ^4^ *Microbiology, Faculty of Medicine, Kuwait University, Kuwait*

**Objective:** Osteoporosis is a significant health issue which is set to rise alarmingly worldwide. In addition to some well‐characterized factors, oxidative stress is also suggested to contribute to bone loss in osteoporosis seen in menopause. Oxidative stress is the result of an imbalance between the production of reactive oxygen species (ROS) and the removal of their reactive intermediates. There is much controversy regarding the possible pathological role of oxidative stress on bone mineral density (BMD). This study was aimed at measuring serum oxidative stress parameters i.e., Catalase, Peroxiredoxin 2 (PRX2), Superoxide dismutase 1 (SOD1), Superoxide dismutase 2 (SOD2) and Thioredoxin (TRx1) in postmenopausal women with normal, osteopenic and osteoporotic bone density.

**Methods:** The study population included 64 post‐menopausal women of whom 23 had normal BMD, 38 had osteopenia and 13 had osteoporosis. Serum oxidative stress parameters were measured using the Multiplex system (Millipore) and read on the Magpex ELISA platform.

**Results:** Serum levels of Catalase, SOD2 and PRX2 were found significantly lower in postmenopausal women with low BMD group as compared to women with normal BMD (*p* = 0.031, 0.044 and 0.041 respectively). These findings support that oxidative stress plays an important role in pathogenesis of postmenopausal osteoporosis. However, levels were not significantly different in women with osteopenia as compared to those with osteoporosis. Furthermore, levels did not correlate significantly with BMD of the hip or spine.

**Conclusions:** These data provide insights into the possible role of oxidative stress on the pathogenesis of postmenopausal osteoporosis. However, the lack of association between studied oxidative stress markers and BMD may indicate that osteoporosis may be rather multivariate and complex.

**Acknowledgment:** This study is supported by Kuwait Foundation of Advancement of Science (KFAS) projects no. 2013–1302‐02 and PR17‐18SL‐01.

**10-13**

**Anthropometric and physical changes in normal weight, overweight and obese community dwelling old adults during a 12 week resistance exercise program**

O.G. Geirsdottir^1,2^, P.V. Jonsson^1,3^, I. Thorsdottir^4^ and Alfons Ramel^1,2^

^1^ *The Icelandic Gerontological Research Center, Reykjavik, Iceland;* ^2^ *Faculty of Food Science and Nutrition, University of Iceland, Reykjavik, Iceland;* ^3^ *Department of Geriatrics, National University Hospital of Iceland, Reykjavik, Iceland;* ^4^ *School of Health Sciences, University of Iceland, Reykjavik, Iceland*

**Introduction:** Resistance exercise is important for the prevention of sarcopenia and physical dependence of old adults. However, little is known whether overweight/obesity affect the outcomes of a resistance exercise program.

**Methods:** Community dwelling Icelandic old adults (*N* = 236, 73.7 ± 5.7 years) participated in a 12‐week resistance exercise program. Anthropometrics, muscular strength and physical function were measured at baseline and endpoint. Participants were grouped retrospectively into normal‐, overweight and obese according to international BMI cut off values. Statistical analyses were corrected for age and gender.

**Results:** Of the participants, 22.0% were normal, 41.4% were overweight and 36.6% were obese. BMI categories were neither related to drop‐out (11.9%) nor to attendance (88.4%). All groups experienced improvements in outcome measurements, but improvements in obese individuals were less pronounced compared to normal weight individuals. There was similar weight gain in the groups (+0.48 kg, *P* < 0.001), however, normal weight individuals gained more lean mass (+0.70 kg, *P* = 0.015), appendicular muscle mass (+0.42 kg, *P* = 0.007) but tended to loose more fat mass (−0.75 kg, *P* = 0.081) as compared to obese. This resulted in a greater change in body fat percent in normal weight subjects as compared to obese (−1.5%, *P* = 0.014).

Absolute gains in quadriceps strength were similar between groups, but relative to body weight, normal weight subject gained more than obese subjects (+0.31 N/kg, *P* = 0.017). This translated into a greater improvement in 6‐minute walk‐for‐distance (+24. m, *P* < 0.001) compared to obese subjects. Interestingly, grip strength increased more in obese subjects (2.4 lb, *P* = 0.020). Timed‐up‐and‐go improved similarly in all groups (−0.64 sec, *P* < 0.001).

**Discussion:** Independently from their body weight status, old adults benefited from a resistance exercise program in terms of body composition and physical function. However, obese individuals improved significantly less than normal weight participants although there was similar attendance to the exercise classes. Great care has to be taken of obese elderly in order to maintain their physical independence as long as possible.
